# Development and pre-clinical testing of a novel hypoxia-activated KDAC inhibitor

**DOI:** 10.1016/j.chembiol.2021.04.004

**Published:** 2021-09-16

**Authors:** Anna Skwarska, Ewen D.D. Calder, Deborah Sneddon, Hannah Bolland, Maria L. Odyniec, Ishna N. Mistry, Jennifer Martin, Lisa K. Folkes, Stuart J. Conway, Ester M. Hammond

**Affiliations:** 1 Department of Chemistry, Chemistry Research Laboratory, University of Oxford, Mansfield Road, Oxford OX1 3TA, UK; 2 Oxford Institute for Radiation Oncology, Department of Oncology, University of Oxford, Old Road Campus Research Building, Oxford OX3 7DQ, UK

**Keywords:** hypoxia, KDAC, HDAC, HAP

## Abstract

Tumor hypoxia is associated with therapy resistance and poor patient prognosis. Hypoxia-activated prodrugs, designed to selectively target hypoxic cells while sparing normal tissue, represent a promising treatment strategy. We report the pre-clinical efficacy of 1-methyl-2-nitroimidazole panobinostat (NI-Pano, CH-03), a novel bioreductive version of the clinically used lysine deacetylase inhibitor, panobinostat. NI-Pano was stable in normoxic (21% O_2_) conditions and underwent NADPH-CYP-mediated enzymatic bioreduction to release panobinostat in hypoxia (<0.1% O_2_). Treatment of cells grown in both 2D and 3D with NI-Pano increased acetylation of histone H3 at lysine 9, induced apoptosis, and decreased clonogenic survival. Importantly, NI-Pano exhibited growth delay effects as a single agent in tumor xenografts. Pharmacokinetic analysis confirmed the presence of sub-micromolar concentrations of panobinostat in hypoxic mouse xenografts, but not in circulating plasma or kidneys. Together, our pre-clinical results provide a strong mechanistic rationale for the clinical development of NI-Pano for selective targeting of hypoxic tumors.

## Highlights

• NI-Pano (CH-03) is reduced in an O_2_-dependent manner to release panobinostat
• Treatment with NI-Pano in hypoxia leads to an accumulation of acetylated histones
• NI-Pano increases cell death in hypoxic conditions but not normoxia
• Treatment with NI-Pano led to tumor growth delay in a xenograft model

## Graphical abstract

**Figure:**
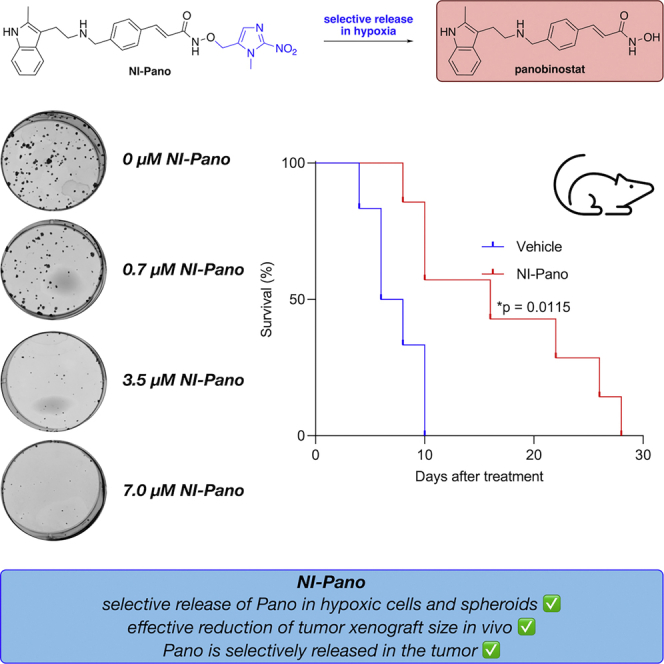

## Teaser

Skwarska, Calder et al. describe the design, synthesis, and biological testing of a hypoxia-activated KDAC inhibitor (NI-Pano, CH-03). NI-Pano is bioreduced in hypoxic conditions to release panobinostat, which leads to an accumulation in histone acetylation, increased cell death, and tumor growth delay.

## Introduction

Insufficient O_2_, or hypoxia, is a common feature of the tumor microenvironment arising from abnormal tumor vasculature and high metabolic demand. Clinically, hypoxia is associated with resistance to chemotherapy, radiotherapy, and poor patient prognosis across a broad range of tumor types (Hammond et al., 2014). O_2_is a radiosensitizer and possesses a high affinity for radicals generated during ionizing radiation. Specifically, O_2_binds covalently to radiation-induced DNA radicals fixing the damage which can lead to DNA double-strand breaks (Bristow and Hill, 2008; Gatti and Zunino, 2005). Therefore, there is a critical, and largely unmet, need for combination approaches to improve radiotherapy response. The mechanisms that underlie resistance to anti-cancer drugs in hypoxic cells are complex and include drug efflux, autophagy, metabolic reprogramming, DNA damage, and mitochondrial activity (Bristow and Hill, 2008; Hammond et al., 2014). In addition, hypoxia leads to extracellular changes in the tumor microenvironment such as acidosis, which can lead to extracellular ion trapping of weakly basic drugs such as doxorubicin (Gatti and Zunino, 2005). The aberrant vasculature found in hypoxic tumors also leads to inefficient drug distribution (Gottesman, 2002). Furthermore, hypoxia leads to cell-cycle arrest and inhibition of proliferation, and as anti-cancer drugs often preferentially target rapidly dividing cells, hypoxia also results in resistance to chemotherapy (Tredan et al., 2007).

As hypoxia represents a major obstacle to therapy, a number of strategies have been developed to overcome tumor hypoxia. These include directly targeting hypoxia inducible factor (HIF), inhibition of HIF downstream target genes, increasing tissue oxygenation, drugs that enhance the diffusion of O_2_, O_2_transport agents (hemoglobin/fluorocarbon based), hemoglobin modifiers, and hypoxia-activated prodrugs (HAPs) (Graham and Unger, 2018; Harris, 2002; Minchinton and Tannock, 2006; Mistry et al., 2017; O'Connor et al., 2015; Spiegelberg et al., 2019a). HAPS are inactive in normoxic cells and undergo selective reduction via endogenous oxidoreductases, which are enhanced under hypoxic conditions, yielding the active drug. Traditionally, HAPs were designed to release a DNA-damaging cytotoxic agent, and as such have limited use in combination with chemotherapies due to overlapping toxicities (Mistry et al., 2017). This led to the development of a generation of molecularly targeted HAPs. Key examples include the hypoxia-activated Chk1 inhibitor (CH-01), DNA-PK inhibitor (BCCA621C), and HER2 inhibitor (tarloxotinib) (Baran and Konopleva, 2017; Cazares-Korner et al., 2013; Portwood et al., 2013).

We have previously synthesized a prototype hypoxia-activated prodrug of the lysine deacetylase (KDAC) inhibitor, vorinostat (suberanilohydroxamic acid [SAHA]). However, while this compound, NI-SAHA, demonstrated effective bioreduction and release of SAHA, it was not suitable for cellular testing due to the instability of SAHA (Calder et al., 2020). KDACs contribute to malignant progression via removal of acetyl groups from histone and non-histone proteins, and their action is reversed by histone acetyltransferases (HATs) (Li and Seto, 2016). An aberrant global histone acetylation profile has been identified in oncogenesis, with loss of acetylation of H4K16 regarded as a common feature across a broad range of cancer types (Fraga et al., 2005). Furthermore, increased expression of class I KDACs has been observed across cancer types. For example, overexpression of KDAC1 is observed in lung, breast, pancreatic, and gastric cancers and is associated with poor patient prognosis (Cao et al., 2017; Feng et al., 2014; Sudo et al., 2011; Zhang et al., 2005). Increased expression or activity of KDACs leads to inappropriate silencing of tumor suppressor genes, contributing to tumorigenesis (Kazanets et al., 2016). Together, this has led to the development of KDAC inhibitors as anti-cancer therapeutics, with SAHA, belinostat, romidepsin, and panobinostat (Pano) gaining FDA approval for the treatment of hematological malignancies.

In hypoxic conditions, cells undergo global changes in histone modifications, induction of KDAC activity, and alterations in KDAC protein interactions. A number of reports have indicated that inhibition of KDACs in hypoxia leads to a reduction in HIF-1α expression and activity, although the mechanism remains unclear (Kim et al., 2001, 2007; Kong et al., 2006). Therefore, the development of HAPs that selectively inhibit KDAC function in hypoxia would allow manipulation of the histone acetylation profile in tumors promoting a favorable clinical outcome, while leaving that of non-cancerous cells unaltered.

## Results

**Figure 1:**
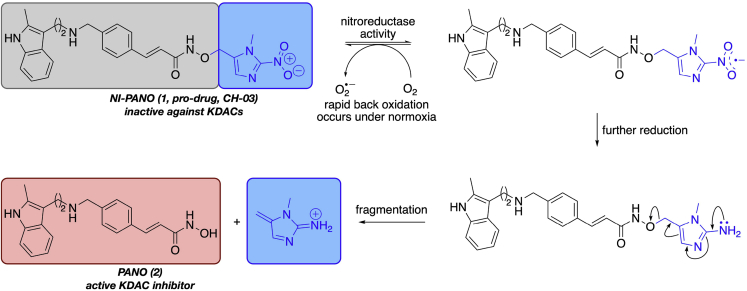
The concept of a hypoxia-activated prodrug of the KDAC inhibitor Pano The hydroxamic acid is protected with a bioreductive group to prevent binding to the KDAC enzymes. The nitroimidazole group undergoes reduction and fragmentation in hypoxia to release the active KDAC inhibitor Pano.

**Figure 2:**
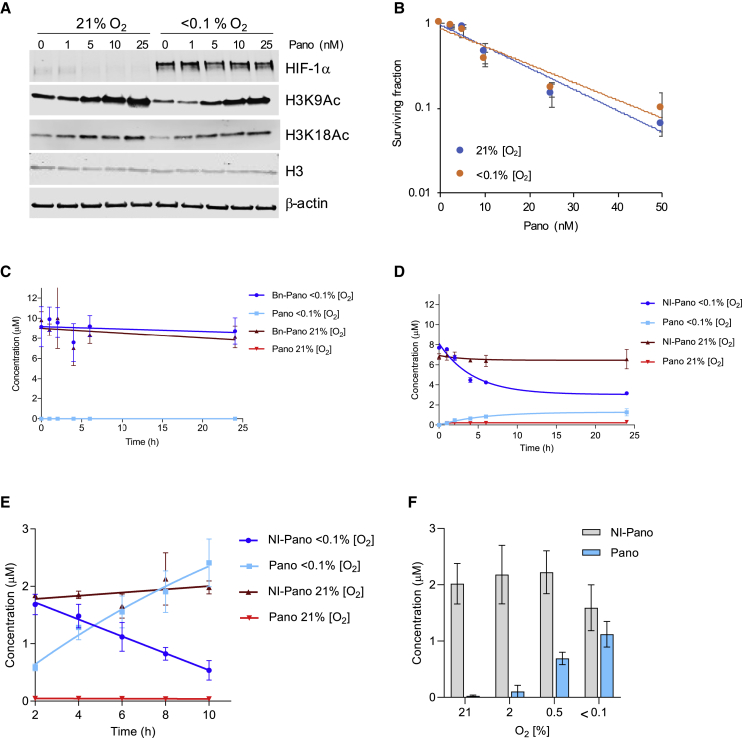
NI-Pano is reduced in an O_2_-dependent manner in OE21 cells and leads to increased histone acetylation (A) OE21 cells were exposed to Pano (0–25 nM) for 24 h in the O_2_ concentrations shown. Western blotting was then carried out as indicated. (B) OE21 cells were treated with Pano (0–50 nM) for 24 h at the O_2_ concentration shown. Pano was removed and cells allowed to form colonies in normoxic conditions. Data are mean ± SD (n = 3). (C) Bn-Pano (10 μM) and (B) NI-Pano (10 μM) were incubated with 9.2 pmol/mL of bactosomal NADPH-CYP reductase (CYP004) in normoxic (21% O_2_) or hypoxic (<0.1% O_2_) conditions for 0–24 h and analyzed using LCMS. Data are mean ± SD, n = 3 except (C), which is n = 2. (E) OE21 cells were treated with NI-Pano (5 μM) for the indicated times and the reduction of NI-Pano to Pano was determined by HPLC. Data are mean ± SD (n = 3). (F) OE21 cells were treated with NI-Pano (5 μM) for 6 h at different O_2_ concentrations and analyzed by HPLC. Data are mean ± SD (n = 3).

We selected Pano (**2**) as the basis of our hypoxia-activated prodrug (Figure 1). Pano has favorable metabolic properties compared with SAHA(Clive et al., 2012), and shows effective inhibition of KDAC1–6 and KDAC9–11. Pano also inhibits KDAC 7 and 8, but with higher half maximal inhibitory concentration (IC_50_) values than the other KDAC enzymes (Arts et al., 2009; Bradner et al., 2010). Before developing a hypoxia-activated form of Pano, we tested Pano under hypoxic conditions in human esophageal OE21 cancer cells. We found that Pano led to increased acetylation of histone 3 lysine 9 (H3K9Ac) and histone 3 lysine 18 (H3K18Ac) in both normoxic (21% O_2_) and hypoxic (<0.1% O_2_) conditions (Figures 2A and S1A). In contrast to previous reports, Pano did not significantly affect HIF-1α stabilization (Kim et al., 2001, 2007; Kong et al., 2006). Importantly, as determined by colony survival assay, hypoxic OE21 cells and those grown under normoxic conditions were equally sensitive to Pano (Figure 2B), supporting the rationale for use of Pano to target hypoxic tumors.

We, and others, have previously demonstrated that blocking the O_2_atom of the hydroxamic acid disrupts binding to the KDAC enzymes (Calder et al., 2020). This ensures that the prodrug is inactive until the drug is released at the desired time and/or location. Four nitroaromatic groups were chosen as the bioreductive moieties to attach to the hydroxamic acid. The nitrobenzyl (NB), nitrothiophene (NT), and nitroimidazole (NI) groups have previously been used by us (Calder et al., 2020; Cazares-Korner et al., 2013; Collins et al., 2018; O'Connor et al., 2016) and others (Baran and Konopleva, 2017; Hunter et al., 2016; Mistry et al., 2017; Phillips, 2016; Sharma et al., 2019; Spiegelberg et al., 2019a; Zeng et al., 2018) as bioreductive groups in HAPs, and have a range of bioreduction potentials (O'Connor et al., 2016). We also investigated the use of the nitroquinoline (NQ) group, which we predicted would have bioreduction properties similar to the NI group. A negative control compound was designed, in which the bioreductive group was replaced with a simple benzyl moiety, which is inert to reduction and consequently fragmentation. We initially proposed that the synthesis of the nitroaromatic-functionalized hydroxylamines would allow a convergent route to all of these molecules.

Pano (**2**) was synthesized using a route that has previously been reported in the literature with minor modification made (Scheme **S1** and supplemental information for details) (Chen et al., 2018). A Grandberg reaction of phenylhydrazine with the chloroketone **S1** gave 2-methyltryptamine (**S2**) (Grandberg, 1974; Grandberg and Zuyanova, 1971; Slade et al., 2007). A Mizoroki-Heck reaction of 4-bromobenzaldehyde (**S3**) with methyl acylate yielded the aldehyde **S4**, which subsequently underwent a reductive amination with the primary amine of **S2** to give **3**. Displacement of the methyl ester with hydroxylamine gave Pano (**2**).

While the *O*-(benzyl)hydroxylamine is commercially available, the remaining functionalized hydroxylamines were synthesized as shown in Scheme S2. Briefly, reaction of *N*-hydroxyphthalimide with 4-nitrobenzyl chloride (**S5**) followed by deprotection with hydrazine gave *O*-(nitrobenzyl)hydroxylamine **S7**. To form the thiophene derivative (**S10**), the commercially available 5-nitrothiophene-2-carboxaldehyde (**S8**) was first reduced to the alcohol, and then brominated with phosphorus tribromide to give **S9**. The bromide was displaced with *N*-hydroxyphthalimide, and hydrazine deprotection gave **S10**. The nitroquinoline bromide has previously been synthesized by our group; however, an alternative route was used here (Conway et al., 2016). Sandmeyer iodination of 5-amino-6-nitroquinoline (**S11**) gave the iodide **S12**. Grignard exchange with phenyl magnesium bromide, followed by addition of paraformaldehyde yielded the primary alcohol, which was brominated with hydrobromic acid to give the bromide **S13**. Following the same steps used for the nitrobenzyl and nitrothiophene analogues gave the nitroquinoline-functionalized hydroxylamine **S14**. The nitroimidazole chloride (**S17**) was synthesized in six steps using a route similar to that we have previously reported (Calder et al., 2020; O'Connor et al., 2016). However, the nitroimidazole-containing hydroxylamine derivative was unstable, meaning that the chloronitroimidazole (**S17**) was coupled to Pano using an alternative procedure (*vide infra*).

**Scheme 1:**
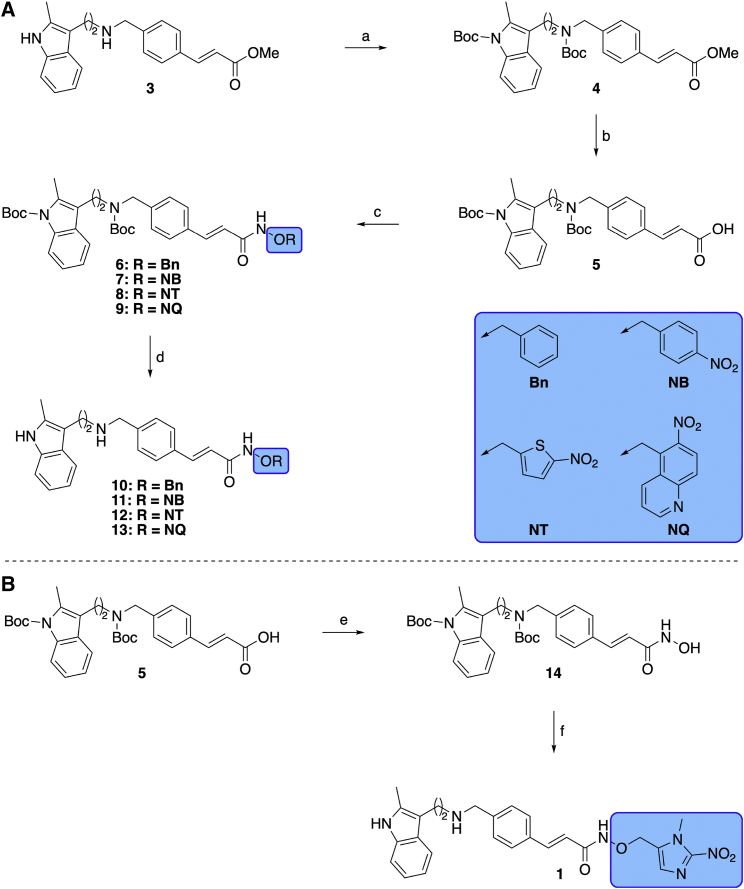
General synthesis of HAP analogues of Pano (1, 11-13) and the negative control compound (**10**) Reagents and conditions: (A) (a) Boc_2_O, DMAP, THF, room temperature (rt), 18 h, 67%–82%, n = 5; (b) LiOH, THF, MeOH, H_2_O, rt, 6 h, 88%–83%, n = 5; (c) RONH_2_, PyBOP, NEt_3_, THF, rt, 18 h, *O*-(benzyl)hydroxylamine, R = Bn, 73%, n = 1, RONH_2_, PyBOP, NEt_3_, THF, rt, 18 h, **S7**, R = NB, 71%–92%, n = 2, RONH_2_, PyBOP, NEt_3_, THF, rt, 18 h, **S10**, R = NT, 62%–84%, n = 2, RONH_2_, PyBOP, NEt_3_, THF, rt, 18 h, **S14**, R = NQ, 60%, n = 1; (d) TFA, TIPS-H, CH_2_Cl_2_, rt, 1 h, **6**, R = Bn, 79%, n = 1, TFA, TIPS-H, CH_2_Cl_2_, rt, 1 h, **7**, R = NB, 68%–69%, n = 2, TFA, TIPS-H, CH_2_Cl_2_, rt, 1 h, **8**, R = NT, 52–64%, n = 2, TFA, TIPS-H, CH_2_Cl_2_, rt, 1 h, **9**, R = NQ, 40%, n = 1. (B) (e) CDI, THF, rt, 1 h then HONH_3_Cl, rt, 18 h, 51%–63%, n = 4; (f) (i) NaH, DMF, −5°C, 20 min then **S17**, −5°C to rt, 18 h, 71%–86%, n = 4, (ii) TFA, TIPS-H, CH_2_Cl_2_, rt, 1 h, 51%–61%, n = 4.

To synthesize the Pano prodrugs, protection of both nitrogen atoms in the core molecule was necessary (Scheme 1). Boc protection of the methyl ester **3** (Scheme 1A), followed by lithium hydroxide-catalyzed hydrolysis, gave the carboxylic acid **5**. Coupling with the functionalized hydroxylamines **S7**, **S10**, **S14**, and *O*-(benzyl)hydroxylamine, using (benzotriazol-1-yloxy)tripyrrolidinophosphonium hexafluorophosphate (PyBOP), followed by deprotection with trifluoroacetic acid (TFA), yielded the inactive control **10** and three of the prodrugs **11–13**. 1,1′-Carbonyldiimidazole (CDI)-mediated coupling of the carboxylic acid **5** with hydroxylamine hydrochloride yielded di-Boc-protected Pano, **14**. This compound was alkylated with the nitroimidazole halide **S17**, and deprotected using TFA and triisopropylsilane (TIPS-H) to give NI-Pano **1** (Scheme 1B).

To determine if the prodrugs **1** and **11–13** underwent hypoxia-dependent reduction and fragmentation, the four HAP analogues, Pano (**2**), and the negative control Bn-Pano (**10**), were incubated with NADPH-CYP reductase (CYP004) in normoxic and hypoxic conditions for up to 24 h. We have previously used this procedure as an initial stage of prodrug validation, and it shows good correlation with cellular activity (O'Connor et al., 2016). The prodrug reduction and release of Pano was monitored using liquid chromatography mass spectrometry (LCMS), as described previously (Calder et al., 2020). As expected, neither Bn-Pano (**10**) nor Pano (**2**) were reduced in normoxic or hypoxic conditions (Figures 2C and S1B). NB-Pano (**11**) appeared stable in both normoxia and hypoxia with no apparent production of Pano (Figure S1C). In previous studies, a higher concentration of enzyme (92 pmol/mL, 10-fold) has been used to reduce the NB group, as this moiety is more stable to bioreduction than other groups (Calder et al., 2020; Cazares-Korner et al., 2013; Collins et al., 2018). With the increased enzyme concentration, Pano was produced in hypoxia (Figure S1D). We have found, however, that the lower enzyme concentration translates well to levels and rates of cellular reduction for this class of compounds, indicating that NB-Pano might not be optimum for cellular and *in vivo* studies.

Pano release from NT-Pano (**12**) was observed in both normoxic and hypoxic conditions (Figure S1E). Although more Pano was released in hypoxia, to be suitable for cellular and *in vivo* studies, there should be no released of Pano in this assay. The levels of NQ-Pano (**13**) were depleted in both normoxic and hypoxic conditions. However, unlike NT-Pano, NQ-Pano did not release Pano under normoxic conditions (Figure S1F). This result suggests that some metabolism of NQ-Pano can occur in normoxia but that fragmentation to release the free drug does not occur. It is possible that NQ-Pano undergoes partial reduction to one of the intermediate reduced states, without fragmentation to release Pano. Partial reduction to intermediates including the nitroso, and full reduction to the amine without fragmentation has been observed with a nitrobenzyl group in hypoxia (Cazares-Korner et al., 2013). However, in this case these intermediates could not be observed using LCMS.

NI-Pano (**1**) was stable in normoxic conditions and did not release detectable Pano; however, in hypoxic conditions, a clear increase in Pano was observed (Figure 2D). This observation is in line with our previous work in which we demonstrated an NI-based HAP of SAHA can undergo bioreduction and fragmentation in the presence of NADPH-CYP reductase (CYP004) (Calder et al., 2020). Therefore, of the Pano-based HAPs synthesized and tested, NQ-Pano (**13**) and NI-Pano (**1**) were found to have the most encouraging and selective reduction and release profiles and were progressed for further evaluation.

We have previously shown that neither NB-SAHA nor NI-SAHA showed any inhibition of a panel of 10 Zn^2+^-dependent KDAC enzymes. We used the same enzyme assay (Reaction Biology) to determine whether the addition of the NQ and NI groups would prevent Pano inhibiting these enzymes. While NQ-Pano retained some inhibitory activity against KDAC1–3 and KDAC6, NI-Pano showed weak or no inhibitory activity against any of the KDAC enzymes tested (Table 1).

**Table 1:** NI-Pano shows little KDAC inhibition *in vitro*

IC_50_ values (nM) for Pano, NI-Pano, and NQ-Pano against KDAC1–9 and KDAC11. The color scale represents a heatmap, with hot’ colors showing effective enzyme inhibition.

^a^Data obtained by Reaction Biology Corporation, NI = no inhibition observed at concentrations up to 10 μM.

^b^Data taken from Arts et al. (2009).

^c^n = 2 biological repeats for all enzymes, value shown is the mean ± SEM.

It initially appears surprising that the NQ group is tolerated by these enzymes, while the NI group is not. As the activity shown by NQ-Pano was not observed for all enzymes, it is reasonable to assume that these data are meaningful, and not a result of assay interference. Analysis of the X-ray crystal structures of KDAC1–3 and KDAC6 (Bergman et al., 2012; Butler et al., 2010; Ho et al., 2018; Huang et al., 2019; Lee et al., 2013; Wagner et al., 2013) shows that they have larger active site pockets than the other KDACs. KDAC1–3 have been shown to accommodate aromatic groups into the active site pocket of, for example, the selective biaryl benzamide KDAC inhibitors (Gao et al., 2017; Witter et al., 2008). The nitro moieties of the NI and NQ groups have different vectors, meaning that they will extend into different areas of the enzyme active site. Our current hypothesis is that the vector of the nitro group of the NI group is particularly unfavorable for enzyme binding, making it an effective bioreductive group for HAPs targeting the KDAC enzymes. The modest residual inhibition activity against KDAC6 might be explained by Pano exhibiting a monodentate coordination to the Zn^2+^ ion, perhaps allowing accommodation of the NI group (Hai and Christianson, 2016). It is also possible that the nitro group is able to weakly coordinate the Zn^2+^ ion in KDAC1–3 and KDAC6; nitro group coordination of Zn^2+^ has previously been observed in carboxypeptidase A inhibitors (Wang et al., 2008).

We continued to test NI-Pano in human esophageal cancer cell lines because it is a tumor type associated with hypoxia (Spiegelberg et al., 2019b). OE21 cells were incubated with NI-Pano (5 μM) for up to 10 h in either normoxia or hypoxia (<0.1% O_2_) followed by high-performance liquid chromatography (HPLC) analysis to determine the Pano release. In normoxia, NI-Pano did not release Pano. However, in hypoxic conditions NI-Pano was quickly reduced (within 2 h) and Pano accumulated within cells (Figures 2E and S1G). As the hypoxic tumor microenvironment includes a gradient of O_2_tensions, we investigated the O_2_dependency of Pano release from NI-Pano. OE21 cells were treated with NI-Pano (5 or 10 μM) and exposed to a range of O_2_ concentrations (<0.1%–2% O_2_). As expected, Pano was barely detectable in the cells incubated at 21% O_2_; however, Pano was readily detectable in the cells exposed to either 0.5% or <0.1% O_2_ demonstrating an O_2_-dependent reduction of NI-Pano to Pano (Figures 2F and S1H).

**Figure 3:**
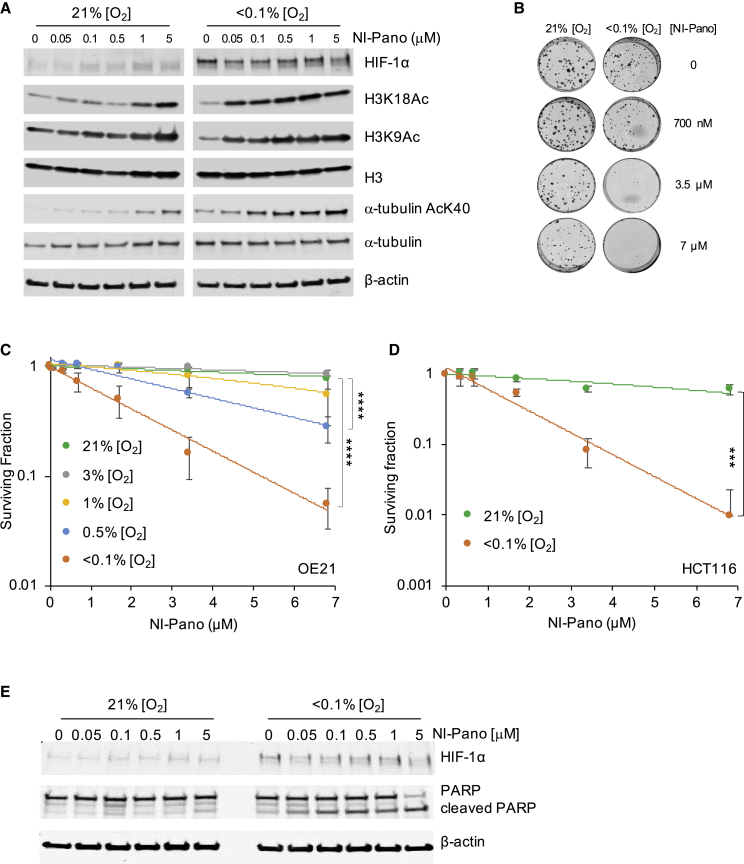
NI-Pano decreases cancer cell survival in hypoxia (A) OE21 cells were treated with a range of doses of NI-Pano as indicated in 21% or <0.1% O_2_ for 6 h followed by western blot analysis using the antibodies indicated. Normoxic and hypoxic samples were run on the same gels but are split for clarity. Additional loading controls are shown in Figure S2A. (B and C) OE21 cells were treated with NI-Pano (0–7 mM) for 24 h at the O_2_ concentration shown. NI-Pano was removed and cells allowed to form colonies for 7 days in normoxic conditions. Data are mean ± SD (n = 3). Significance: two-way ANOVA test, ^∗∗∗∗^p < 0.0001. Representative images are shown in (B). (D) HCT116 cells were treated with NI-Pano (0–7 μM) for 24 h at the O_2_ concentration shown. NI-Pano was removed and cells allowed to form colonies in normoxic conditions. Data are mean ± SD (n = 3). Significance: two-way ANOVA test, ^∗∗∗^p < 0.001. (E) OE21 cells were treated with NI-Pano (0–5 μM) in 21% or <0.1% O_2_ for 6 h followed by western blotting as indicated.

Having determined that NI-Pano was converted to Pano in human cell lines, we asked if this was sufficient to inhibit KDAC activity. OE21 cells were treated with a range of doses of NI-Pano (0.05–5 μM) in normoxic and hypoxic (<0.1% O_2_) conditions. Western blotting demonstrated that only those cells exposed to hypoxia (<0.1% O_2_) showed an increase in H3K18 and H3K9 acetylation at doses as low as 0.05 μM. As HDAC6 has been shown to deacetylate α-tubulin, we also investigated the effect of NI-Pano on α-tubulin K40Ac (Skultetyova et al., 2017). Again, as expected, the levels of α-tubulin K40Ac increased when NI-Pano was added to cells in hypoxia. In comparison, cells in normoxic conditions showed no change in any of the acetylation points investigated until doses of 1–5 μM were reached (Figures 3A and S2A). In agreement with the data shown (Figure 2A), the Pano released from NI-Pano in hypoxic conditions did not appear to significantly affect HIF-1α levels. Next, to determine the NI-Pano-dependent impact on cell viability, colony survival assays were carried out in a range of O_2_concentrations (<0.1%–21% O_2_). OE21 cells were treated with NI-Pano (0–7 μM) for a period of 24 h in the conditions indicated. Significant loss of viability was observed in the cells incubated at <0.1 and 0.5% O_2_ (Figures 3B and 3C). We verified that this was not cell line dependent using the HCT116 cell line, which also showed NI-Pano-dependent loss of viability specifically in hypoxic conditions (Figures 3D and S2B). In addition, cytotoxicity was determined by MTT assay and again demonstrated a hypoxia-dependent effect of NI-Pano (Figures S2C and S2D). We investigated a possible Pano-dependent mitotic arrest by determining the levels of H3 serine 10 phosphorylation. As expected, the mitotic fraction decreased significantly in response to hypoxia and we found that this was not altered by the presence of NI-Pano (Figure S2E). KDAC inhibition has been reported to lead to an increase in apoptosis (Zhang and Zhong, 2014). Here, NI-Pano treatment in hypoxic conditions (<0.1% O_2_) led to apoptosis as determined by increased PARP cleavage (Figure 3E). Little change in PARP cleavage was observed in normoxic conditions, further demonstrating that NI-Pano does not induce apoptosis in normoxic conditions. Bioreduction of NI-Pano generates a quinone-methide-like by-product (Figure S3A). To determine if this intermediate contributed to the NI-Pano mediated toxicity, we synthesized (*E*)-2-(2-(4-((1-methyl-2-nitro-1*H*-imidazol-5-yl)methoxy)styryl)-4*H*-chromen-4-ylidene)malononitrile IOD (Figure S3A). This compound will release the same quinone-methide-like by-product as NI-Pano, providing an ideal control compound to determine the toxicity of this product. We therefore carried out an MTT assay on treated OE21 cells treated with IOD (Figures S3B–S3D). As expected, we saw no impact on cell viability of IOD-treated OE21 cells with 21% O_2_ or <0.1% O_2_, providing strong evidence that the quinone-methide-like intermediate produced by bioreduction is not toxic, and the source of cell death caused by bioreduction of NI-Pano results from the released Pano. The toxicity of the same nitroimidazole-based quinone methide-like by-product has also recently been shown to be non-toxic in in H460, HeLa, or A549 cell lines (10 μM concentration over 24 h) (Jin et al., 2017).

**Figure 4:**
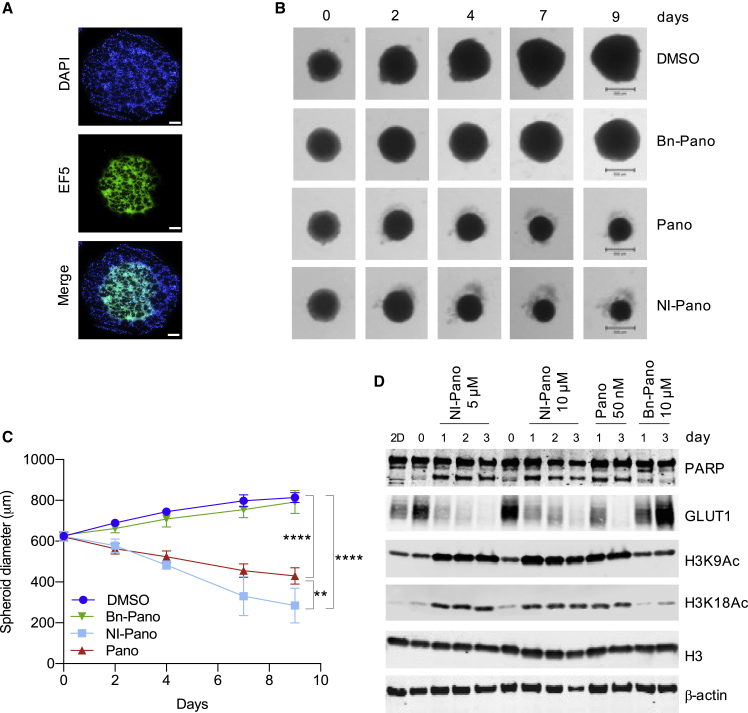
NI-pano inhibits growth of HCT116 cells grown as 3D spheroids (A) Representative images of HCT116 cells grown as spheroids and treated with EF-5 to visualize the hypoxic core. DAPI was used as a nuclear marker. Scale bar, 100 μM. (B) HCT116 cell spheroids were treated with Bn-Pano (10 μM), Pano (50 nM), or NI-Pano (2.5 μM) in normoxic (21% O_2_) conditions for 9 days, representative images of live spheroids are shown. Scale bar, 500 μM. (C) The change in spheroid diameter following treatment as in (B) was quantified using GelCount software. At least four spheroids were analyzed per condition. Data are mean ± SD (n = 4). Significance: two-way ANOVA test, ^∗∗^p < 0.01, ^∗∗∗∗^p < 0.0001. (D) HCT116 spheroids were grown and treated with NI-Pano (5 or 10 mM), Pano (50 nM), or Bn-Pano (10 μM) followed by western blotting. HCT116 cells grown in normoxic (21% O_2_) 2D culture were used for comparison (labeled as 2D). Two lanes with samples from day 0 are shown to aid comparison.

The presence of hypoxic regions in solid tumors is, in part, the result of abnormal vasculature, which results in a reduced supply of O_2_, nutrients, and also chemotherapeutic drugs (Sharma et al., 2019; Tannock et al., 2002). Before moving to *in vivo* testing, we made use of cells grown as 3D spheroids to assess whether NI-Pano could diffuse through multiple cell layers to reach the hypoxic core. HCT116 cells were utilized for this assay as OE21 cells failed to form large spheroids. HCT116 cells were grown as spheroids, reaching sizes of up to 500–600 μm. The presence of hypoxic cores was demonstrated using EF5 staining (Figure 4A). HCT116 cells grown as spheroids were then allowed to continue growing in the presence of Bn-Pano, Pano, NI-Pano, or DMSO. The diameter of the spheroids was measured, and representative spheroids are shown (Figure 4B). While the diameter of the spheroids treated with either DMSO or Bn-Pano increased over time, those treated with Pano or NI-Pano decreased significantly (Figure 4C). These data suggest that NI-Pano was able to penetrate the hypoxic core of the spheroid and was reduced to release Pano. HPLC analysis of lysates from treated spheroids confirmed the presence of Pano in spheroids treated with NI-Pano (Figure S4A). Protein lysates were then prepared from treated spheroids and markers of hypoxia, apoptosis, and histone acetylation were investigated. An increase in H3K9 and H3K18 acetylation was observed when the spheroids were treated with NI-Pano (5 or 10 μM) and Pano but not with the negative control, Bn-Pano (Figure 4D). Changes in the acetylation of H3K56 and α-tubulin were also observed consistent with NI-Pano being active in treated spheroids (Figure S4B). In support of our previous findings demonstrating increased PARP cleavage and therefore apoptosis, we found that this was also evident in the treated spheroids. In addition, we determined the levels of GLUT1, a well-characterized hypoxia marker and HIF target (Chen et al., 2001), and found that, firstly, the untreated spheroids (day 0) had high expression of GLUT1, again confirming the presence of hypoxia, and, secondly, that the level of GLUT1 decreased with treatment with either NI-Pano or Pano. Given that we did not observe a marked Pano-dependent effect on HIF-1α stabilization, these data suggest that GLUT1 expression is decreased in spheroids treated with NI-Pano as a result of a reduction of the hypoxic fraction through increased Pano-dependent apoptosis.

**Figure 5:**
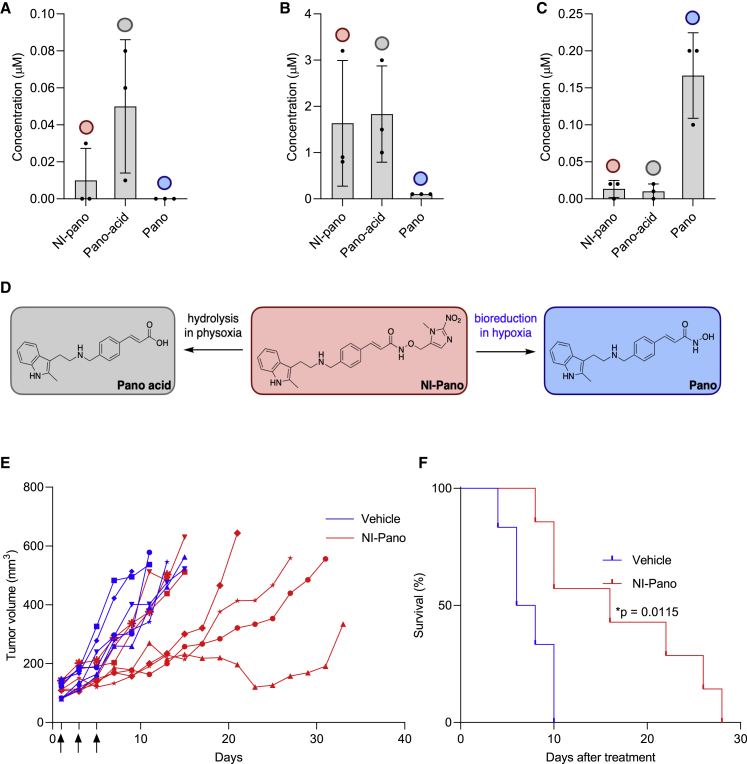
NI-Pano is reduced to Pano *in vivo* and significantly inhibits tumor growth rate OE21 xenografts were treated with three doses of 50 mg/kg NI-Pano on days 1, 3, 5. Treatment was initiated when the mean size of the tumor reached approximately 100 mm^3^. (A–C) (A) plasma, (B) kidney, and (C) tumors were harvested from three mice 24 h after the last dose of NI-Pano and analyzed by LCMS. (D) The chemical structures of the Pano-acid, Pano, and NI-Pano. (E) Tumor growth rates in individual vehicle- (n = 6) and NI-Pano (n = 7)-treated mice. Arrows indicate days of treatment. Tumor size was measured every second day and volumes exceeding 500 mm^3^ were used as a study endpoint. (F) Kaplan-Meier survival analysis. The differences between vehicle and NI-Pano were statistically significant by log rank examination.

We next tested the efficacy of NI-Pano in a xenograft model. OE21 cells were grown as xenograft tumors and were found to contain regions of hypoxia as determined by pimonidazole staining (Figure S5A). Tumor-bearing mice (n = 7) were injected intraperitoneally with NI-Pano (50 mg/kg) on days 1, 3, and 5 after randomization. Mouse plasma, kidney, and tumors were collected 24 h after the last dose of NI-Pano (day 6) and analyzed by LCMS. The concentration of NI-Pano in plasma was significantly lower than in kidney, suggesting renal clearance of NI-Pano but no significant accumulation in plasma (Figures 5A and 5B). In addition, we detected Pano-acid, a product of NI-Pano hydrolysis in both the plasma and kidney. To determine the likely impact of Pano-acid on cell viability, we synthesized Pano-acid and carried out a colony survival assay using OE21 cells. Pano-acid was not toxic and had no effect on the viability of OE21 cells (Figure S5B). To verify that Pano-acid was cell penetrant HPLC analysis was carried out on OE21 cells and determined that Pano-acid accumulated inside the cells (Figure S5C). Interestingly, although NI-Pano was hydrolyzed to Pano-acid in mouse plasma, this did not occur in rat or human plasma (Figures S5D and S5E). This difference is consistent with the high level of nonspecific esterases found in mouse plasma compared with rat and human (Rudakova et al., 2011). While Pano was barely detectable in plasma and kidney, tumor samples had sub-micromolar levels of Pano (Figures 5C and 5D). Importantly, the level of Pano in tumor xenografts exceeded the nanomolar concentrations required to inhibit survival of OE21 cells *in vitro*. Finally, we asked whether the level of Pano released from NI-Pano in tumor xenografts was sufficient to translate into antitumor activity by measuring the growth of the tumors over time. Tumors in untreated mice grew rapidly and reached the endpoint size in less than 2 weeks (range 4–10 days) (Figure 5E and S6). NI-Pano treatment resulted in a clear tumor growth delay and significantly increased survival (range 8–28 days) (Figure 5F).

## Discussion

We have designed and synthesized a new bioreductive prodrug of the KDAC inhibitor, Pano. Of the four prodrugs investigated, NI-Pano showed the most attractive HAP characteristics as shown by little or no inhibition of KDACs in the non-reduced form, stability in normoxic conditions, and being reduced to release Pano in hypoxic conditions. We demonstrated increased histone acetylation in an NI-Pano- and hypoxia-dependent manner. Most importantly, treatment of hypoxic tumors led to a significant growth delay in an esophageal xenograft mouse model, therefore demonstrating the translational potential of this molecule. It is likely that the growth-inhibitory effects of NI-Pano were due to the release of Pano in the hypoxic cells and subsequent diffusion to the surrounding, more oxygenated cells.

Previously, we developed NI-SAHA, a hypoxia-activated prodrug of KDAC inhibitor SAHA. NI-SAHA is enzymatically reduced under hypoxic conditions to release SAHA. NI-SAHA, while achieving the characteristics of an HAP in enzyme assays, was not suitable for further testing as the released drug SAHA has a short half-life *in vivo* (Calder et al., 2020). In contrast, further development of NI-Pano could be beneficial for a wide range of cancer types, such as esophageal and colorectal cancers, as both tumor types have been found to develop significant levels of hypoxia. A clear potential use of NI-Pano is in combination with radiotherapy. Selective elimination of hypoxic tumors may be achieved by the combination of NI-Pano with lower doses of radiation, simultaneously sparing normal tissues from side effects of high-dose radiotherapy and off-target effects associated with pan-KDAC inhibition. In addition, the standard of care for myeloma in the UK is a combination of proteasome inhibitor (bortezomib) and KDAC inhibitor (Pano). However, this combination is associated with severe gastrointestinal toxicity in patients and particularly the elderly group (San-Miguel et al., 2013). Myelomas have been described as hypoxic and form in the hypoxic areas of bones, suggesting that the use of NI-Pano would not only reduce the observed toxicity but would also be efficacious (Hu et al., 2010). NI-Pano could affect the treatment of pediatric gliomas, including highly hypoxic diffuse intrinsic pontine gliomas (DIPGs), which occur in the brainstem and are the most lethal types of cancer in children (Meel et al., 2018). The majority of DIPGs have oncogenic mutations in histone H3 (Weinberg et al., 2017), and Pano has shown therapeutic efficacy *in vitro* and in DIPG xenografts models, raising the possibility for new treatment options (Hennika et al., 2017). While clinical trials of Pano alone or in combination with proteasome blockade have only recently been launched in children with DIPG (ClinicalTrials.gov NCT02717455 and NCT04341311), it is possible that NI-Pano could be a less toxic alternative for these patients.

The recent development and use of checkpoint inhibitors is significantly changing cancer therapy and leading to these agents being approved as standard of care for a number of tumor types (Darvin et al., 2018; Fritz and Lenardo, 2019). The hypoxic regions of tumors have been described as immune cold and therefore resistant to immune therapy (Chouaib et al., 2017). Thus, there is an opportunity to use HAPs in combination with immune therapy as the HAP should decrease the hypoxic fraction of tumors and restore sensitivity to immune therapy. In support of this hypothesis, a recent report described the use of TH-302 in prostate cancer models and showed that, by using the HAP to target hypoxia, T cell infiltration was increased and sensitivity to CTLA-4 and PD-1 blockade was restored (Jayaprakash et al., 2018).

In summary, the novel KDAC inhibitor prodrug described here, NI-Pano, has favorable biochemical and pharmacologic properties with the potential to selectively eliminate hypoxic cells. The O_2_ dependency of Pano release from NI-Pano suggests that organs that experience the lowest physiological levels of O_2_ should be shielded from KDAC inhibition. Our pre-clinical results provide a strong mechanistic rationale for the further development of NI-Pano as a means to selectively target hypoxic tumors.

## Significance

**Although bioreductive prodrugs have been described previously, these have predominantly focused on agents that release non-targeted toxic agents in response to hypoxia. A more recent strategy is to design molecularly targeted prodrugs, which release active inhibitors of therapeutic targets in hypoxic conditions. This approach has significant advantages over the agents previously described, none of which have been adopted into routine clinical use. A key advantage to creating a bioreductive of a previously known and clinically used drug is that the side effects are already known and can be minimized by targeting to hypoxic areas. This study describes the pre-clinical testing of a novel bioreductive prodrug of the KDAC inhibitor Pano, which we have called NI-Pano (CH-03). We demonstrate that NI-Pano has little or no inhibitory activity on KDACs but is reduced to release active Pano in hypoxic conditions. Our analysis demonstrates the O**_**2**_ **dependency of this reduction in increasingly complex biological systems, including purified enzymes, cancer cell lines, 3D spheroids, and, importantly, mouse xenografts.**

## STAR★Methods

### Key resources table

**Table:** 

REAGENT or RESOURCE	SOURCE	IDENTIFIER
**Antibodies**		

Hif-1α	Novus Biologicals	Cat#NB100-122; RRID: AB_10002593
H3K9Ac	Cell Signaling	Cat#9649S; RRID: AB_823528
H3K18Ac	Cell Signaling	Cat#13998S; RRID: AB_2783723
H3K56Ac	Cell Signaling	Cat#4243S; RRID: AB_10548193
H3	Cell Signaling	Cat#3638S; RRID: AB_1642229
PARP	Cell Signaling	Cat#9542S; RRID: AB_2160739
GLUT1	Abcam	Cat#ab652; RRID: AB_305540
α-tubulin	Santa Cruz	Cat#sc-5286; RRID: AB_628411
α-tubulin K40Ac	Cell Signaling	Cat#5335; RRID: AB_10544694
β-actin	Santa Cruz	Cat#sc-69876; RRID: AB_1126280
Goat anti-Mouse IgG (H+L)	LI-COR Biosciences	IRDye® 680RD
Donkey anti-Rabbit IgG (H+L)	LI-COR Biosciences	IRDye® 800CW
EF5	Millipore	Clone ELK3-51
Hypoxyprobe 1 antibody	Hypoxyprobe	Clone 4.3.11.3

**Chemicals, peptides, and recombinant proteins**		

Bactosomal human NADPH-CYP reductase	Cypex	CYP004
NADPH-regenerating system	Corning	A 451220 and B 451200

**Critical commercial assays**		

Mycoplasma testing - PlasmaTest	InvivoGen	rep-pt1

**Experimental models: cell lines**		

Human OE21 (male)	PHE culture collections	96062201
Human HCT116 (male)	ATCC	ATCC® CCL-247

**Experimental models: organisms/strains**		

Mouse: 6-week old female athymic CD-1 nude mice	Charles River	Crl:CD1-*Foxn1*^*nu*^

**Software and algorithms**		

GraphPad Version 8.4.3 (471)	Prism	https://www.graphpad.com/scientific-software/prism/

### Resource availability

#### Lead contact

Further information and requests for resources and reagents should be directed to and will be fulfilled by the lead contact, Stuart Conway (stuart.conway@chem.ox.ac.uk).

#### Materials availability

There are restrictions to the availability of NI-Pano and IOD due to limited supply.

#### Data and code availability

This study did not generate/analyze datasets/code.

### Experimental model and subject details

#### Animal studies

Animal studies were approved by the University of Oxford Biomedical Services Ethical Review Committee, Oxford, UK. OE21 cells were grown as xenograft tumors as previously described (Leszczynska et al., 2016). Briefly, cells were prepared in phenol red-free Matrigel (BD Biosciences) and RPMI medium 1:1, and 100 μL (5 x 10^6^ cells) injected subcutaneously into the flank of 6-week old female athymic CD-1 nude mice (Charles River). Xenograft volumes were determined every second day using [length x width x height x π/6] formula. Mice were randomized when the mean xenograft volume reached 100 mm^3^. Animal groups received either vehicle (5% DMSO, 5% sulfobutylether-β-cyclodextrin in sterile water, (*n*= 6) or three doses of 50 mg/kg NI-Pano intraperitoneally (*n* = 7) every second day (D1, D3, D5). Tumor volumes exceeding 500 mm^3^ was used as a study end point. To confirm hypoxic regions in OE21 xenografts, additional control mice (*n* = 3) were injected (ip) with 60 mg/kg of pimonidazole and 2 h later tumors were harvested, fixed in 4% paraformaldehyde, embedded in paraffin and sectioned. Samples, after dewaxing and antigen retrieval, were stained with primary hypoxyprobe 1 antibody (clone 4.3.11.3, Hypoxyprobe) followed by secondary HRP-conjugated antibody. HRP was developed with 3,3′-diaminobenzidine (DAB, Vector Labs), and samples were counterstained with hematoxylin and eosin (H&E). Images were obtained using an Aperio Scanner (Leica Biosystems). Study approval: *in vivo* experiments were performed under UK Home Office-approved project Licence PPL30/3395 and personal licenses PILIBBFCF7C6 and I2117011B.

#### Cell lines

OE21 esophageal squamous cancer cells (male) were obtained from PHE culture collections and were grown in RPMI medium supplemented with 10% FBS, penicillin (100 U/mL) and streptomycin (100 μg/mL). The HCT116 colorectal cancer cell line (male) (ATCC) was cultured in DMEM medium supplemented with 10% FBS, penicillin (100 U/ mL) and streptomycin (100 μg/mL). Cells were cultured in a humidified incubator at 37°C and 5% CO_2_ unless otherwise stated. Cell lines were not authenticated in our hands. All cell lines were routinely mycoplasma tested and found to be negative.

### Method details

#### Hypoxia treatment

Hypoxic experiments at 0.5-3% O_2_ were carried out in a Whitley H35 Hypoxystation (Don Whitley). For hypoxic experiments at <0.1% O_2_, cells were plated on glass dishes and experiments carried out in a Bactron II Anaerobic chamber (Shell Laboratories).

#### Immunoblotting

Cells were collected and lysed in UTB (9 M urea, 75 mM Tris-HCl pH 7.5, 0.15 M β-mercaptoethanol) and briefly sonicated. Primary antibodies were HIF-1α (NB100-122 Novus Biologicals), H3K9Ac (9649S Cell Signaling), H3K18Ac (13998S Cell Signaling), H3K56Ac (4243S Cell Signaling), H3 total (3638S Cell Signaling), PARP (9542S Cell Signaling), GLUT1 (ab652 Abcam), α-tubulin (sc-5286 Santa Cruz), α-tubulin K40Ac (5335 Cell Signaling) and β−actin (sc-69876 Santa-Cruz biotechnology). Secondary antibodies were IRDye® 680RD Goat anti-Mouse IgG (H+L) and IRDye® 800CW Donkey anti-Rabbit IgG (H+L) from LI-COR Biosciences. Odyssey IR imaging technology (LI-COR Biosciences) was used for imaging.

#### Colony survival assay

Cells were seeded at low density in 6-well plates and incubated for 4 h at 37°C to adhere. Cells were treated in a range of oxygen tensions; media was then changed, and cells incubated under normoxic (21% O_2_) conditions for 7-10 days. Colonies were stained with 2% crystal violet diluted in 50% methanol and 20% ethanol and counted manually. Plating efficiency was calculated by dividing the numbers of colonies by the number of cells seeded and the surviving fraction was determined by dividing plating efficiency for treatment by the plating efficiency for respective control.

#### MTT assay

Cells were incubated with 0.5 mg/mL MTT reagent in complete media for 3 hours at 37°C protected from light. MTT was removed and formazan crystals were solubilized with 100 μl DMSO for 15 mins at 37°C protected from light. Absorbance was read immediately at 570 nm. MTT assays for hypoxic samples were performed inside the hypoxia chamber. Data expressed as percentage viability relative to vehicle normoxic control.

#### NADPH reductase/CYP450 assay

Bactosomal human NADPH-CYP reductase (9.2 or 92 pmol/mL, CYP004, Cypex) were used in combination with NADPH-regenerating system (A 451220 and B 451200, Corning) as previously described (O'Connor et al., 2016). Enzymatic reactions were carried out in glass vials under normoxic (21% O_2_) or hypoxic (<0.1% O_2_) conditions. Aliquots (50 μL) were taken at different time points and immediately quenched with MeCN (50 μL). After centrifugation samples were analyzed by LCMS. LCMS was performed on a Waters 2695 system using an RPB column (5 μm, 100 mm × 3.2 mm, 35°C). Separation was achieved at a flow rate of 0.5 mL/min with a gradient of 5–95% acetonitrile in 0.1% aqueous trifluoroacetic acid over 12 minutes, returning to starting conditions over 0.1 minute. Detection used a photodiode array spectrophotometer (Waters 2996) and a mass spectrometer (Waters Micromass ZQ mass spectrometer).

#### Cellular reduction of NI-Pano

Cells were incubated with NI-Pano under normoxic or hypoxic condition, washed twice with PBS, collected by scraping and centrifugation. The cell pellet was mixed with 100 μL MeOH/MeCN (1:1), briefly sonicated and centrifuged to remove cell debris. Resulting supernatants were analyzed by HPLC. HPLC analysis was performed on a Waters 2695 system using an RPB column (5 μm, 100 mm × 3.2 mm, 35°C). Separation was achieved at a flow rate of 0.5 mL/min with a gradient of 5–95% acetonitrile in 0.1% aqueous trifluoroacetic acid over 12 minutes, returning to starting conditions over 0.1 minute. Detection used a photodiode array spectrophotometer (Waters 2996). Injections of 10 μL were made.

#### Cellular permeability of pano acid

Cells were incubated with Pano acid under normoxic conditions, washed twice with PBS, collected by scraping and centrifugation. The cell pellet was mixed with 100 μL MeOH/MeCN (1:1), briefly sonicated and centrifuged to remove cell debris. Resulting supernatants were analyzed by HPLC.

#### Spheroid growth

HCT116 cells (5x10^3^) were seeded in 200 μL of DMEM medium in 96-well U-bottom ultra-low attachment plates (Corning). The plates were incubated at 37°C in 5% CO_2_ for 5 days until the mean diameter of spheroids reached approximately 600 μm. Then, 100 μL of medium was replaced with fresh medium containing drugs and spheroid diameter was measured over 9 days. Spheroids were imaged and analyzed using GelCount Tumor Colony Counter (Oxford Optronix). At least 4 spheroids were measured per each treatment condition.

#### Spheroid immunofluorescence

Spheroids were incubated with 200 μM EF5 (2-(2-nitro-1*H*-imidazol-1-yl)-*N*-(2,2,3,3,3-pentafluoropropyl) acetamide) for 6 h, fixed in 4% paraformaldehyde at 4°C overnight and treated with 30% sucrose (*w/v*, PBS) for 3 h before mounting in OCT embedding medium (ThermoScientific) as described previously (Leszczynska et al., 2016). Spheroids were sectioned, rehydrated in 0.1% Tween (*v/v*, PBS), and blocked with 0.5% (*w/v*) FP1020 blocking reagent (PerkinElmer) in 0.1 M Tris-HCl, pH 7.5 and 0.15 M NaCl for 2 h at room temperature. Sections were washed with 0.1% Tween before overnight treatment at 4°C with anti-EF5 (clone ELK3-51) Alexa Fluor 488 conjugate antibody (Millipore). Next, sections were washed with 0.1% Tween and stained with DAPI solution (1 μg/mL) for 10 min, and the slides were mounted using ProLong Diamond mounting medium (Invitrogen/Life Technologies).

#### *Ex vivo* studies on NI-Pano stability in rat and human plasma

Plasma extracted from healthy rats and human donors was incubated with 20 μM NI-Pano at 37°C. Samples were collected at 30 min internals and extracted into acetonitrile and the dried extract reconstituted in 70% 0.1% TFA/30% acetonitrile. Metabolism was measured using a Waters 2996 separations module with an incubation temperature of 15°C. Separation was achieved on an ACE Amide (C18, 3 μm, 100 x 2.1 mm) column maintained at 35°C Samples were eluted in 70% 0.1 % TFA/30% acetonitrile with a flow rate of 0.25 mL/min with a run time of 9 min. Standards were prepared in surrogate matrix (5% BSA *w/v* in PBS). Analytes were detected by mass spectrometry with a Waters Acquity QDa detector with electrospray ionization in a positive ionization. Pano was detected at SIR of *m/z* 350.5, Pano-acid at *m/z* 335.0 and NI-Pano at *m/z* 489.2 (M+H).

#### Analysis of NI-Pano, pano-acid and pano levels in xenografts and tissue samples

Mice were sacrificed 24 h after receiving third dose of NI-Pano. Blood was collected by cardiac puncture into sodium citrate vials (Sarstedt Ltd, UK), centrifuged for 10 minutes at 1500 *g* and the resulting plasma was immediately frozen in dry ice. Xenografts and organs were collected, snap frozen in dry ice before analysis by LCMS-MS. For LCMS-MS, tumor xenograft or tissue was homogenized in ice-cold water with a 5-fold dilution. Homogenates were mixed with internal standard (carbamazepine) and extracted into acetonitrile. Samples were reconstituted in 35%. HPLC analysis was carried out on a Waters Acquity H-Class Quarternary Solvent Manager. Separation was achieved on an ACE Excel amide (C18, 3 μm, 100 x 2.1 mm) column maintained at 35°C with eluents A: 10 mM formic acid, B: acetonitrile. For the analysis of NI-pano and pano-acid samples were eluted with a gradient of 30% B 3min, 30-70% 0.6 min, hold 1.4 min -80% acetonitrile over 5 min with a run time of 9 min. For the analysis of pano samples were re-injected and eluted in 65% 0.1% formic acid/35% acetonitrile with a flow rate of 0.25 mL/min and a run time of 8 min. Analytes were detected by mass spectrometry with a Waters Acquity TQ detector with electrospray ionization in a positive ionization mode. Pano was detected with multiple reaction monitoring (MRM) of 350.5 > 158.0, Pano-acid *m/z* 335.0 > 158 and IS *m/z* 236.9 > 194.1. NI-Pano was detected with SIR *m/z* 489.2.

#### Chemistry experimental section

Chemicals were purchased from Acros Organics, Alfa Aesar, Apollo Scientific, Fisher Scientific, Fluka, Fluorochem, Merck or Sigma Aldrich and were used without further purification. Where appropriate and if not otherwise stated, all non-aqueous reactions were carried out under an inert atmosphere of argon, using flame-dried glassware. Anhydrous solvents were obtained under the following conditions: THF, acetonitrile, dichloromethane, diethyl ether, and DMF were dried by passing them through a column of active basic alumina according to Grubbs’ procedure and stored over activated 3 Å molecular sieves under argon (Pangborn et al., 1996). Anhydrous methanol and ethanol was purchased from Sigma Aldrich UK in SureSeal™ bottles and used without further purification. Analytical thin layer chromatography (TLC) was performed on normal phase Merck silica gel 60 F254 aluminium-supported thin layer chromatography sheets. Spots were visualized by either absorption under UV light (254 nm), exposure to iodine vapor or thermal development after dipping into a solution of ammonium molybdate in sulfuric acid, an aqueous solution of potassium permanganate or an ethanolic solution of ninhydrin. Reaction progress was monitored at appropriate times using TLC analysis. Normal phase silica gel flash column chromatography was performed manually using Geduran Silicagel 60 (40–63 μm) under a positive pressure of compressed nitrogen or on a Biotage SP1 automated column chromatography system using KP-Sil® SNAP Flash Silica Cartridges. ^1^H NMR spectra were recorded on a Bruker AVIIIHD 400 (400 MHz) a Bruker AVII 500 with dual ^13^C(^1^H) cryoprobe (500 MHz) or a Bruker AVIIIHD 500 (500 MHz) spectrometer with the stated solvents as a reference for the internal deuterium lock. Chemical shifts are reported as δ_H_ in parts per million (ppm) relative to tetramethylsilane (TMS) where δ_H_ (TMS) = 0.00 ppm. The spectra are calibrated using the solvent peak with the data provided by Fulmer et al. (Fulmer et al., 2010). The multiplicity of each signal is indicated by: s (singlet); br s (broad singlet); d (doublet); t (triplet); q (quartet); dd (doublet of doublets); ddd (doublet of doublet of doublets); m (multiplet). The number of protons (*n*) for a given resonance signal is indicated by *n*H. Identical proton coupling constants are averaged in each spectrum and reported to the nearest 0.1 Hz. The coupling constants were determined by analysis using Bruker TopSpin software (versions 3.2 and 4.0) or Mestrenova software (version 11). ^1^H spectra were assigned using 2D NMR experiments including ^1^H-^1^H COSY, ^13^C-^1^H HSQC and ^13^C-^1^H HMBC. ^13^C NMR spectra were recorded on a Bruker AVIIIHD 400 (101 MHz) or a Bruker AVII 500 with dual ^13^C(^1^H) cryoprobe (126 MHz) spectrometer in the stated solvents with broadband proton decoupling and an internal deuterium lock. Chemical shifts are reported as δ_C_ in parts per million (ppm) relative to tetramethylsilane (TMS) where δ_C_ (TMS) = 0.00 ppm. The spectra are calibrated using the solvent peak with the data provided by Fulmer et al. (Fulmer et al., 2010) The shift values of resonances are quoted to 1 decimal place unless peaks have similar chemical shifts, in which case 2 decimal places are used. ^13^C spectra were assigned using 2D NMR experiments including HSQC and ^13^C-^1^H HMBC. Electrospray ionization (ESI) mass spectra were acquired using an Agilent 6120 Quadrupole spectrometer or Waters LCT Premier spectrometer, operating in positive or negative mode, as indicated, from solutions of MeOH or MeCN. Chemical ionization (CI) mass spectra were acquired using a Waters GCT spectrometer. MS data was processed using Mestrenova software (version 11). *m/z* values are reported in Daltons and followed by their percentage abundance in parentheses. Accurate mass spectra were obtained using Bruker μTOF spectrometer. *m/z* values are reported in Daltons. When a compound was not observed by LRMS, only HRMS is quoted. Melting points were determined using either a Griffin capillary tube melting point apparatus or a Kofler hot stage and are uncorrected. The solvent(s) from which the sample was crystallized is given in parentheses. Infrared (IR) spectra were obtained either from neat samples, either as liquids or solids, or as a thin film using a diamond ATR module. The spectra were recorded on a Bruker Tensor 27 spectrometer. Absorption maxima are reported in wavenumbers (cm^−1^). Only the main, relevant peaks have been assigned. Semi-preparative HPLC purification of NI-Pano was carried out on Waters Autopurification system, equipped with a Waters Atlantis T3 column (19 mm × 100 mm, 5 μm), with an injection loop of 1 mL, eluting with H_2_O+0.1% TFA/MeOH +0.1% TFA. The crude samples (in MeOH, DMSO <10%) were filtered (nylon, 0.2 μm) and injected in 750 μL aliquots, with mass-directed purification with an ACQUITY QDa performance mass spectrometer. The gradient profile is as shown in Table S1.

#### Compound purity

The purity of Pano (**2**), Pano acid (**S18**), and NB-Pano (**11**) was determined using analytical high-performance liquid chromatography (HPLC) on a PerkinElmer Flexar system with a Binary LC Pump and UV/Vis LC Detector. Purity of NI-Pano (**1**) was determined using either the instrument described or by analytical high-performance liquid chromatography (HPLC) on an Agilent 1260 Infinity II fitted with a quaternary pump, vial sampler, DADWR, column chamber and an Agilent Infinity fraction collector or by liquid chromatography mass spectrometry (LCMS) on a Waters 2695 autosampler with a Waters QDa detector. All batches used for biological testing were >95% purity. All batches used for animal studies were >99% purity.

**Table:** Table S1. Semi-preparative HPLC gradient profile.

Time (mins)	Flow (mL/min)	% H_2_O + 0.1% TFA	% MeOH + 0.1% TFA	Curve
0	20	95	5	6
1	20	95	5	6
17	20	5	95	6
18	20	5	95	6
20	20	95	5	6

**Purity** of all novel compounds except NI-Pano was determined using analytical high-performance liquid chromatography (HPLC) on a PerkinElmer Flexar system with a Binary LC Pump and UV/Vis LC Detector. All biologically tested compounds were of >95% purity as determined by HPLC. For determination of compound purity on reversed phase (RP) 2-10 μL of sample was injected a Dionex Acclaim® 120 column (C18, 5 μm, 12 Å, 4.6 × 150 mm). The gradient profile, unless otherwise stated is shown in Table S2. **Method A** refers to the solvents shown without modifiers. **Method B** refers to the solvents shown with the addition of 0.1% *v/v* TFA. **Method C** refers to the solvents shown, without modifiers, with an additional 10-minute hold at 5% H_2_O, 95% MeCN at the end of the gradient profile.

**Table:** Table S2. Analytical HPLC gradient profile for Methods A, B and C.

Time (min)	Flow (mL/min)	% H_2_O	% MeCN
0	1.5	95	5
10	1.5	5	95
15	1.5	5	95

**Purity of NI-Pano** was determined using analytical HPLC as described above (Method B) or by one of the methods described below. **Method D**: Purity was determined using analytical HPLC on an Agilent 1260 Infinity II fitted with a quaternary pump, vial sampler, DADWR, column chamber and an Agilent Infinity fraction collector fitted with a XBridge BEH C18, 4.6 × 150 mm, 5 μm column. Samples were prepared in 10% DMSO in MeOH and injected at 10 μL. The gradient profile is shown in Table S3.

**Table:** Table S3. Gradient profile for Agilent Infinity.

Time (mins)	Flow (mL/min)	% H_2_O + 0.1% TFA	% MeCN + 0.1% TFA
0	1	95	5
1	1	95	5
11	1	5	95
13	1	5	95
14	1	95	5
15	1	95	5

**Method E**: Purity was determined using analytical HPLC on a Waters 2695 autosampler with Waters QDa detector fitted with an ACE Excel 3 C18 Amide column (100 × 3mm, 3 μm). Samples were prepared at 10 μM in 0.1% DMSO in H_2_O and injected at 10 μL. Samples were run on an isocratic method H_2_O + 0.1% TFA/Methanol + 0.1% TFA (70:30) at 3 mL/min at 35°C.

#### Experimental methods

##### 2-Methyltryptamine **(S2)**

5-Chloropentan-1-one (**S1**) (3.55 mL, 27.4 mmol, 1.4 eq, 85% purity) was added dropwise to a rapidly stirred solution of phenylhydrazine (2.00 mL, 19.6 mmol, 1.0 eq) in absolute ethanol (200 mL) at rt. The solution was then slowly heated to 80°C (WARNING: uncontrolled exotherm can occur if heated too fast) and stirred at this temperature for 18 h, cooled to rt and concentrated *in vacuo*. The residue was partitioned between water (100 mL) and dichloromethane (100 mL), the aqueous layer was separated and extracted with dichloromethane (2 × 100 mL). A saturated aqueous solution of sodium hydrogen carbonate (20 mL) was added to the aqueous components, which were then extracted with dichloromethane (2 × 100 mL). A 2 M aqueous solution of sodium hydroxide (20 mL) was added to the aqueous components, which were then extracted with ethyl acetate (3 × 50 mL). The ethyl acetate components were then washed with water (100 mL), brine (100 mL), dried (Na_2_SO_4_), filtered, and concentrated *in vacuo* to yield the title compound (**S2**) (3.02 g, 89%) as a yellow solid. *R*_*f*_ 0.29 (10% MeOH/CH_2_Cl_2_+1% Et_3_N); mp 91–93°C (from methanol) [lit. (Righi et al., 2012) 95°C] [lit. (Slade et al., 2007) 90°C from toluene]; ^1^H NMR (400 MHz, CDCl_3_) δ_H_ 8.20 (1H, br s), 7.51 (1 H, dd, *J* 7.0, 1.5), 7.29–7.21 (1H, m), 7.17–7.04 (2H, m), 2.98 (2H, td, *J* 6.7, 0.7), 2.86 (2H, t, *J* 6.7), 2.37 (3H, s); LRMS *m/z* (ESI^+^) 277 (38%), 175 (100%, [M+H]^+^), 158 (68%, [M–NH_2_]^+^). The spectroscopic data are consistent with literature (Righi et al., 2012).

##### (*E*)-Methyl-3-(4-formylphenyl)prop-2-enoate **(S4)**

Methyl acrylate (387 μL, 4.32 mmol, 4.0 eq) was added to a solution of 4-bromobenzaldehyde (**S3**) (200 mg, 1.08 mmol, 1.0 eq), palladium (II) acetate (12.0 mg, 0.0533 mmol, 0.05 eq) and potassium acetate (212 mg, 2.16 mmol, 2.0 eq) in *N,N-*dimethylformamide (11 mL) and heated to 110°C for 24 h. The reaction was cooled to rt and diluted with diethyl ether (110 mL) then filtered through a plug of silica and concentrated *in vacuo* to yield the title compound (**S4**) (203 mg, 99%) as a colorless solid. *R*_*f*_ 0.58 (50% ethyl acetate/petroleum ether); mp 84–86°C (from EtOH), [lit. (Hajipour et al., 2010) 81–84°C from EtOH]; ^1^H NMR (400 MHz, CDCl_3_) δ_H_ 10.02 (1 H, s), 7.89 (2H, d, *J* 8.0), 7.71 (1H, d, *J* 16.1), 7.66 (2H, d, *J* 8.0), 6.54 (1H, d, *J* 16.1), 3.82 (3H, s); LRMS (ESI^+^) 381 ([2M+H]^+^, 100%), 205 (61%), 108 (76%). The spectroscopic data are consistent with literature (Hajipour et al., 2010).

##### (*E*)-Methyl-3-(4-[(2-[2-methyl-1*H*-indol-3-yl]ethylamino)methyl]phenyl)prop-2-enoate **(3)**

(*E*)-Methyl-3-(4-formylphenyl)prop-2-enoate (**S4**) (322 mg, 1.70 mmol, 1.0 eq), 2-methyltryptamine (**S2**) (590 mg, 3.39 mmol, 2.0 eq) and 3 Å molecular sieves (1.00 g) were combined in a solution of 1,2-dichloroethane (21 mL) and acetic acid (100 μL, 1.70 mmol, 1.0 eq) (on scales >1.0 g, 2.0 eq of acetic acid was required to maintain the yield) and stirred at rt for 1 h. Sodium triacetoxyborohydride (719 mg, 3.39 mmol, 2.0 eq) was added to the solution and stirred for 18 h. The reaction mixture was filtered through a pad of Celite® then quenched with saturated aqueous sodium hydrogen carbonate solution (50 mL) and extracted with chloroform (2 × 100 mL). The organic components were combined and washed with water (200 mL), brine (200 mL) then dried (Na_2_SO_4_), filtered, and concentrated *in vacuo*. Purification using column chromatography (elution with 3–70% ethanol:chloroform) yielded the title compound (**3**) (509 mg, 86%) as a colorless solid. *R*_*f*_ 0.32 (10% EtOH/CHCl_3_); mp 82–84°C (from dichloromethane/hexane); ${\bar{\nu }}_{\max}$ (thin film)/cm^−1^ 3401, 3055, 2918, 2851, 1718, 1608, 1462, 1434, 1325, 1206, 1170; ^1^H NMR (400 MHz, CDCl_3_) δ_H_ 7.91 (1H, s), 7.67 (1H, d, *J* 16.1), 7.51 (1H, d, *J* 7.7), 7.44 (2H, d, *J* 8.2), 7.28 (2H, d, *J* 8.2), 7.26–7.24 (1H, m), 7.15–7.05 (2H, m), 6.41 (1H, d, *J* 16.1), 3.81 (5H, s), 2.99–2.88 (4H, m), 2.37 (3H, s); ^13^C NMR (101 MHz, CDCl_3_) δ_C_ 167.7, 144.8, 143.1, 135.4, 133.1, 131.8, 128.8, 128.6, 128.2, 121.1, 119.3, 118.1, 117.3, 110.3, 109.3, 53.6, 51.8, 49.7, 24.9, 11.9; HRMS *m/z* (ESI^+^) Found: 349.1907, C_22_H_25_N_2_O_2_ requires [M+H]^+^ 349.1911; LRMS (ESI^+^) 349 ([M+H]^+^,100%), 332 (7%), 158 (9%); HPLC Method A, Retention time - 8.0 min, 97%.

##### (*E*)-*N*-Hydroxy-3-(4-(((2-(2-methyl-1*H*-indol-3-yl)ethyl)amino)methyl)phenyl)acrylamide (panobinostat, 2)

A solution of potassium hydroxide (2.8 g, 50 mmol, 168 eq) in dry methanol (7 mL) was added dropwise with rapid stirring to a solution of hydroxylamine hydrochloride (2.3 g, 34 mmol, 114 eq), in dry methanol (12 mL). The precipitate was quickly removed by filtration and the resulting solution was added to a solution of **3** (0.10 g, 0.30 mmol, 1.0 eq) in dry methanol (1 mL). The reaction mixture was stirred at rt for 20 h then quenched to pH 7 with 1 M aqueous hydrochloric acid then extracted with ethyl acetate (3 × 50 mL). The organic components were washed with brine (150 mL), dried (Na_2_SO_4_), filtered, and concentrated *in vacuo*. Purification using column chromatography (elution with 1:2:3 water:ethanol:ethyl acetate) yielded the title compound (**2**) (63 mg, 63%) as a colorless solid. *R*_*f*_ 0.54 (1:2:3 water:isopropanol:ethyl acetate); mp 100–104°C (from methanol) [lit. (Xu, 2017) 109–115°C]; ${\bar{\nu }}_{\max}$ (thin film)/cm^−1^ 3195, 3055, 2921, 1659, 1623, 1462, 1340, 1049, 977; ^1^H NMR (500 MHz, CD_3_OD) δ_H_ 7.54 (2H, d, *J* 15.8), 7.49 (2H, d, *J* 7.9), 7.38 (1H, d, *J* 7.9), 7.29 (2H, d, *J* 7.9), 7.23 (1H, d, *J* 8.1), 7.00 (1H, ddd, *J* 8.1, 7.0, 1.2), 6.92 (1H, ddd, *J* 7.9, 7.0, 1.0), 6.45 (1H, d, *J* 15.8), 3.83 (2H, s), 2.95 (2H, t, *J* 7.2), 2.87 (2H, t, *J* 7.2), 2.35 (3H, s); ^13^C NMR (126 MHz, CD_3_OD) δ_C_ 166.3, 141.4, 141.1, 137.2, 135.5, 133.2, 130.2, 129.8, 128.9, 121.4, 119.5, 118.5, 118.3, 111.4, 108.4, 53.6, 50.1, 24.6, 11.4; HRMS *m/z* (ESI^+^) Found 350.18630, C_21_H_24_O_2_N_3_ requires [M+H]^+^ 350.18625; LRMS (ESI^+^) 350 ([M+H]+,100%), 333 (10%), 242 (8%), 158 (40%); HPLC Method B, Retention time - 6.5 min, 94–99% purity at three wavelengths, 97% mean. All batches used for biological assays were >95% purity. The spectroscopic data are consistent with the literature (Chen et al., 2018).

##### *N*-Phthalimido-*O*-(4’-nitrobenzyl)-hydroxylamine (S6)

*N*,*N*-Diisopropylethylamine (5.78 mL, 33.1 mmol, 1.8 eq) was added to a stirred solution of *N*-hydroxyphthalimide (3.00 g, 18.4 mmol, 1.0 eq) in *N*,*N*-dimethylformamide (18 mL). 4-Nitrobenzyl chloride (**S5**) (4.12 g, 23.9 mmol, 1.3 eq) was added the solution was heated to 70°C for 2 h. The reaction was cooled to rt, diluted with ethyl acetate (200 mL) and washed with aqueous 0.5 M lithium chloride (4 × 200 mL), dried (MgSO_4_), filtered, and concentrated *in vacuo* then crystallized from hot ethanol to yield the title compound (**4**) (4.61 g, 84%) as a colorless solid. *R*_*f*_ 0.12 (20% ethyl acetate: petroleum ether); mp 191–193°C (from EtOH) [lit. (Wang et al., 2011) 191–193°C]; ^1^H NMR (400 MHz, CDCl_3_) δ_H_ 8.30–8.21 (2H, m), 7.87–7.80 (2H, m), 7.79–7.76 (2H, m), 7.76–7.72 (2H, m), 5.31 (2H, s); LRMS *m/z* (ESI^+^) 321 (100%, [M+Na]^+^). The spectroscopic data are consistent with literature (Wang et al., 2011).

##### *O*-(4-Nitrobenzyl)-hydroxylamine **(S7)**

A 65% *w/v* aqueous solution of hydrazine monohydrate (501 μL, 6.71 mmol, 4.0 eq) was added to a solution of *N*-phthalimido-*O*-(4-nitrobenzyl)-hydroxylamine (**S6**) (500 mg, 1.68 mmol, 1.0 eq) in methanol (9 mL) and dichloromethane (9 mL) and stirred at rt for 4 h. The suspension was diluted with dichloromethane (20 mL), filtered, and concentrated *in vacuo*. The residue was dissolved in diethyl ether (40 mL), washed with water (2 × 40 mL) then brine (40 mL), dried (Na_2_SO_4_), filtered, and concentrated *in vacuo*. The residue was dried by azeotroping from toluene, the title compound (**S7**) (202 mg, 72%) crystallized on cooling to give a yellow solid. *R*_*f*_ 0.44 (100% ethyl acetate); mp 47–50°C (from dichloromethane:hexane) [lit. (Brady and Klein, 1927) 56°C from light petroleum]; ^1^H NMR (400 MHz, CDCl_3_) δ_H_ 8.53–8.02 (2H, m), 7.59–7.40 (2H, m), 5.53 (2H, br s), 4.77 (2H, s).; LRMS *m/z* (ESI^+^) 393 (60%), 209 (100%), 152 ([M−NH_2_]^+^, 42%). The spectroscopic data are consistent with literature (Wang et al., 2011).

##### (5-Nitrothiophen-2-yl)methanol

Sodium borohydride (0.484 g, 12.7 mmol, 2.0 eq) was added portion-wise to a stirred solution of 2-formyl-5-nitrothiophene (**S8**) (1.00 g, 6.37 mmol, 1.0 eq) in methanol (64 mL) at 0°C. The reaction mixture was warmed to rt over 2 h. After this time, it was cooled to 0°C and the pH carefully adjusted to pH 7 with a 1 M aqueous solution of hydrochloric acid. The solution was diluted with methanol (30 mL) and concentrated *in vacuo*. Purification using column chromatography (10–60% ethyl acetate:petroleum ether) yielded (5-nitrothiophen-2-yl)methanol (1.00 g, 99%) as a pale yellow oil. *Rf* 0.48 (50% ethyl acetate:petroleum ether); ^1^H NMR (400 MHz, CDCl_3_) δ_H_ 7.82 (1H, d, *J* 4.1), 6.94 (1H, dt, *J* 4.1, 1.0), 4.88 (2H, br s), 2.18 (1H, t, *J* 5.6); LRMS *m/z* (ESI^−^) 687 (67%), 659 (39%), 643 (74%), 320 (34%), 275 (37%), 643 (74%), 204 (57%), 158 ([M−H]^−^, 100%). The spectroscopic data are consistent with literature (Wiles et al., 2006; Winn et al., 2017).

##### (5-Nitrothiophen-2-yl)methyl bromide (S9)

Phosphorus tribromide (1.41 mL, 12.3 mmol, 2.0 eq) was added dropwise to a solution of (5-nitrothiophen-2-yl)methanol (979 mg, 6.16 mmol, 1.0 eq) in dichloromethane (123 mL) at 0°C. The solution was warmed to rt over 5 h, quenched with a saturated aqueous solution of sodium hydrogen carbonate (50 mL), and extracted with dichloromethane (100 mL). The organic components were washed with brine (100 mL), dried (Na_2_SO_4_), filtered, and concentrated *in vacuo*. Purification by filtration through a pad of silica gel (elution with 4:1 petroleum ether:ethyl acetate) yielded the title compound (**S9**) (652 mg, 48%) as a brown oil. *Rf* 0.53 (20% ethyl acetate:petroleum ether); ${\bar{\nu }}_{\max}$ (thin film)/cm^−1^ 3106 (CH), 1496 (NO_2_), 1332 (NO_2_), 1237, 1212, 1028; ^1^H NMR (500 MHz, CDCl_3_) δ_H_ 7.78 (1H, d, *J* 4.2), 7.06 (1H, dt, *J* 4.2, 0.7), 4.62 (2H, d, *J* 0.7); ^13^C NMR (126 MHz, CDCl_3_) δ_C_ 152.0^∗^, 148.4, 128.5, 127.2, 24.7; HRMS *m/z* (EI^+^) Found: 220.9151, C_5_H_4_BrNO_2_S requires [M]^+^ 220.9141; LRMS No ion observed; HPLC Method B, Retention time - 9.6 min, 94%. ^∗^Signal at 152.0 confirmed by ^1^H-^13^C HMBC correlation see supplemental information for details. The spectroscopic data are consistent with literature (Sohda et al., 1998).

##### *N*-Phthalimido-*O*-(5-nitrothiophen-2-yl)-hydroxylamine

A solution of *N*-hydroxyphthalimide (388 mg, 2.38 mmol, 1.1 eq) in *N,N*-dimethylformamide (1.1 mL) was added to a solution of (5-nitrothiophen-2-yl)methyl bromide (**S9**) (480 mg, 2.16 mmol, 1.0 eq) in *N,N*-dimethylformamide (1.1 mL) and stirred at rt for 2 h. The reaction mixture was diluted with ethyl acetate (20 mL), quenched to pH 7 with a 1 M aqueous solution of hydrochloric acid then extracted with ethyl acetate (3 × 20 mL). The organic components were washed with a 0.5 M aqueous solution of lithium chloride (5 × 20 mL), brine (20 mL), dried (MgSO_4_), filtered, and concentrated *in vacuo*. Purification using column chromatography (elution with 0–5% ethanol:chloroform) removed some impurities. Trituration with ice cold chloroform yielded the title compound (413 mg, 63%) as a tan solid. *R*_*f*_ 0.54 (100% chloroform); mp 185–187°C (from chloroform); ${\bar{\nu }}_{\max}$ (thin film)/cm^−1^ 1733, 1503, 1350; ^1^H NMR (500 MHz, CDCl_3_) δ_H_ 7.84 (2H, dd, *J* 5.5, 3.1), 7.82 (1H, d, *J* 4.1), 7.78 (2H, dd, *J* 5.5, 3.1), 7.18 (1H, d, *J* 4.1), 5.34 (2H, s); ^13^C NMR (126 MHz, CDCl_3_) δ_C_ 163.4, 153.4, 143.1, 135.0, 128.9, 128.8, 128.3, 124.0, 73.2; HRMS No ion observed; LRMS No ion observed; HPLC Method A, Retention time - 9.5 min, 97%.

##### *O*-(5-Nitrothiophen-2-yl)methylene)hydroxylamine (S10)

A 65% *w/v* aqueous solution of hydrazine monohydrate (0.21 mL, 2.6 mmol, 4.0 eq), was added to a solution of *N*-phthalimido-*O*-(5-nitrothiophen-2-yl)-hydroxylamine (0.20 g, 0.66 mmol, 1.0 eq) in dichloromethane (13 mL) and stirred for 2 h at rt. The resulting suspension was filtered through a pad of silica gel, eluting with chloroform, and concentrated in *vacuo* to yield the title compound (**S10**) (77 mg, 68%) as an orange solid that decomposed rapidly. *R*_*f*_ 0.34 (100% chloroform); mp 40–42°C (from dichloromethane); ${\bar{\nu }}_{\max}$ (thin film)/cm^−1^ 3322, 3106, 2920, 1537, 1497, 1334, 1153; ^1^H NMR (500 MHz, CDCl_3_) δ_H_ 7.81 (1H, d, *J* 4.1), 6.96 (1H, dd, *J* 4.1, 0.9), 5.63 (2H, s), 4.80 (2H, d, *J* 0.9); ^13^C NMR (126 MHz, CDCl_3_) δ_C_ 151.9, 149.2, 128.4, 125.7, 72.1; HRMS No ion observed; LRMS No ion observed; HPLC Method A, Retention time - 6.8 min, 97%.

##### 5-Iodo-6-nitroquinoline (S12)

5-Amino-6-nitroquinoline (**S11**) (500 mg, 2.65 mmol, 1.0 eq), copper(I) iodide (503 mg, 2.65 mmol, 1.0 eq) and sodium nitrite (402 mg, 5.82 mmol, 2.2 eq) were combined and dissolved in dimethyl sulfoxide (26 mL) then heated to 60°C. A 50% aqueous solution of hydroiodic acid (2.20 mL, 28.6 mmol, 5.4 eq) was added dropwise at 60°C and heating was continued for 30 min. The reaction mixture was cooled to 0°C and quenched with a saturated aqueous solution of potassium carbonate. The quenched reaction mixture was extracted with ethyl acetate (5 × 50 mL) and the combined organic fractions were washed with brine (200 mL), dried (Na_2_SO_4_), filtered, and concentrated *in vacuo*. Purification using column chromatography, eluting with 0–100% ethyl acetate:petroleum ether, yielded the title compound (**S12**) (475 mg, 60%) as an off-white solid. *R*_*f*_ 0.53 (50% ethyl acetate:petroleum ether); mp 134–136°C (from chloroform), [lit. (Sapountzis et al., 2005) 160–162°C]; ^1^H NMR (500 MHz, CDCl_3_) δ_H_ 9.02 (1H, dd, *J* 4.2, 1.6), 8.68 (1H, ddd, *J* 8.7, 1.6, 0.8), 8.22 (1H, dd, *J* 9.0, 0.8), 7.94 (1H, d, *J* 9.0), 7.63 (1H, dd, *J* 8.7, 4.2); LRMS *m/z* (ESI^+^) 301 ([M+H]^+^, 23%), 190 (64%), 181 (100%), 144 (29%). The spectroscopic data are consistent with literature (Sapountzis et al., 2005).

##### 5-(Hydroxymethyl)-6-nitroquinoline

5-Iodo-6-nitroquinoline (**S12**) (0.15 g, 0.50 mmol, 1.0 eq) was dissolved in dry tetrahydrofuran (2.5 mL) and cooled to −40°C. A 1 M solution of phenyl magnesium chloride (0.55 mL, 0.55 mmol, 1.1 eq) in 2-methyltetrahydrofuran was added dropwise and the solution was stirred for 10 min. Paraformaldehyde (60 mg, 2.0 mmol, 4.0 eq) was added to the solution and the reaction mixture was warmed to rt over 1 h then heated to 40°C for 18 h. The reaction mixture was cooled to rt, quenched with water (20 mL) and extracted with ethyl acetate (3 × 20 mL). The combined organic fractions were washed with brine (50 mL), dried (Na_2_SO_4_), filtered, and concentrated *in vacuo*. Purification by column chromatography, eluting with 0–100% ethyl acetate:petroleum ether, yielded 5-(hydroxymethyl)-6-nitroquinoline (88 mg, 86%) as an off-white solid. *R*_*f*_ 0.30 (ethyl acetate); mp 139–141°C (from ethyl acetate); ^1^H NMR (400 MHz, CDCl_3_) δ_H_ 9.09 (1H, dd, *J* 4.2, 1.6), 8.78 (1H, ddd, *J* 8.7, 1.6, 0.9), 8.22 (1H, dd, *J* 9.2, 0.9), 8.10 (1H, d, *J* 9.2), 7.65 (1H, dd, *J* 8.7, 4.2), 5.14 (2H, s), 2.79 (1H, s); LRMS *m/z* (ESI^+^) 205 ([M+H]^+^, 100%). The spectroscopic data are consistent with literature (Conway et al., 2016).

##### 5-(Bromomethyl)-6-nitroquinoline **(S13)**

5-(Hydroxymethyl)-6-nitroquinoline (63 mg, 0.31 mmol, 1.0 eq) was dissolved in a 47% aqueous solution of hydrobromic acid (0.62 mL) and warmed to 75°C for 18 h. The reaction mixture was cooled to rt and the pH was adjusted to 7 with solid potassium carbonate. The solution was extracted with ethyl acetate (3 × 20 mL), dried (Na_2_SO_4_), filtered, and concentrated *in vacuo* to yield the title compound (**S13**) (82 mg, 99%) as a yellow solid. *R*_*f*_ 0.37 (50% ethyl acetate:petroleum ether), [lit., (Conway et al., 2016) 0.33, 50% ethyl acetate: petroleum ether]; mp 96–98°C (dec.; from chloroform), [lit., (Conway et al., 2016) 112–114°C from ethyl acetate]; ^1^H NMR (400 MHz, CDCl_3_) δ_H_ 9.11 (1H, dd, *J* 4.2, 1.6), 8.67 (1H, ddd, *J* 8.7, 1.6, 0.9), 8.24 (1H, d, *J* 9.2), 8.14 (1H, d, *J* 9.2), 7.70 (1H, dd, *J* 8.7, 4.2), 5.11 (2H, s); LRMS *m/z* (ESI^+^) 407 (100%), 377 (29%), 269 (^81^Br [M+H]^+^, 12%), 267 (^79^Br [M+H]^+^, 11%), 221 (16%). The spectroscopic data are consistent with literature (Conway et al., 2016).

##### *N*-Phthalimido-*O*-(6-nitroquinolin-5-yl)-hydroxylamine

*N*,*N*-Diisopropylethylamine (75 μL, 0.43 mmol, 1.4 eq) was added to a stirred solution of *N*-hydroxyphthalimide (60 mg, 0.37 mmol, 1.2 eq) in *N*,*N*-dimethylformamide (1 mL). 6-Nitroquinolin-5-yl-methyl bromide (**S13**) (82 mg, 0.31 mmol, 1.0 eq) was added and the solution was stirred at 70°C for 2 h. The reaction was cooled to rt, diluted with ethyl acetate (20 mL), washed with 0.5 M lithium chloride solution (4 × 20 mL) then dried (Na_2_SO_4_), filtered, and concentrated *in vacuo.* Purification using column chromatography, eluting with 0–60% ethyl acetate:petroleum ether, then crystallization from hot chloroform yielded *N*-phthalimido-*O*-(6-nitroquinolin-5-yl)-hydroxylamine (94 mg, 88%) as a colorless solid. *Rf* 0.21 (50% ethyl acetate:petroleum ether); mp 225–227°C (dec.; from DMSO); ${\bar{\nu }}_{\max}$ (thin film)/cm^−1^ 1721, 1527, 1392, 1138; ^1^H NMR (500 MHz, (D_6_-DMSO) δ_H_ 9.16 (1H, dd, *J* 4.1, 1.6), 9.14–9.08 (1H, m), 8.30 (1H, d, *J* 9.1), 8.16 (1H, d, *J* 9.1), 7.97–7.77 (6H, m), 5.81 (2H, s); ^13^C NMR (126 MHz, (D_6_-DMSO) δ_C_ 162.9, 153.3, 148.7, 148.2, 135.1, 134.9, 132.9, 128.4, 127.4, 125.0, 123.44, 123.42, 123.3, 69.6; HRMS *m/z* (ESI^+^) Found: 350.07711, C_18_H_12_O_5_N_3_ requires [M+H]^+^ 350.07715; LRMS *m/z* (ESI^+^) 372 ([M+Na]^+^, 21%), 350 ([M+H]^+^, 17%), 107 (100%); HPLC Method A, Retention time - 9.1 min, 95%.

##### *O*-(6-Nitroquinolin-5-yl)-hydroxylamine **(S14)**

A 65% *w/v* aqueous solution of hydrazine monohydrate (0.14 mL, 1.9 mmol, 8.0 eq) was added to a solution of *N*-phthalimido-*O*-(6-nitroquinolin-5-yl)-hydroxylamine (84 mg, 0.24 mmol, 1.0 eq) in dichloromethane (3.2 mL) and methanol (1.2 mL) and stirred at rt for 5 h. The suspension was diluted with dichloromethane (20 mL), filtered then concentrated *in vacuo*. The residue was triturated with minimal chloroform to yield the title compound (**S14**) (49 mg, 92%) as an orange solid that decomposed rapidly. *R*_*f*_ 0.26 (50% ethyl acetate petroleum ether); mp 96–98°C (from chloroform); ${\bar{\nu }}_{\max}$ (thin film)/cm^−1^ 1531, 1499, 1414, 1008, 904; ^1^H NMR (500 MHz, CDCl_3_) δ_H_ 9.06 (1H, dd, *J* 4.2, 1.7), 8.71 (1H, dd, *J* 8.8, 1.7), 8.21 (1H, d, *J* 9.1), 8.01 (1H, d, *J* 9.1), 7.60 (1H, dd, *J* 8.8, 4.2), 5.51 (2H, br s), 5.33 (2H, s); ^13^C NMR (126 MHz, CDCl_3_) δ_C_ 152.8, 149.1, 149.0, 134.8, 132.0, 128.6, 127.6, 123.6, 122.9, 68.6; HRMS *m/z* (ESI^+^) Found: 220.07168, C_10_H_10_O_3_N_3_ requires [M+H]^+^ 220.07167; LRMS *m/z* (ESI^−^) 256 ([M+^37^Cl]^−^, 26%), 254 ([M+^35^Cl]^−^, 100%), 218 ([M−H]^−^, 93%), 216 (59%), 185 (29%), 169 (46%), 113 (37%); HPLC Method A, Retention time - 6.6 min, 90%.

##### Ethyl (2-amino-1-methyl-imidazol-5-yl)carboxylate **(S16)**

Ethyl *N*-methylglycine hydrochloride (**S15**) (6.00 g, 39.0 mmol, 1.0 eq) was dried using lyophilization then suspended in a combination of dry tetrahydrofuran (37 mL), dry absolute ethanol (4.2 mL), and ethyl formate (22 mL) and cooled to 0°C under a stream of argon. Sodium hydride (3.74 g, 156 mmol, 4.0 eq) was added in small portions to the cooled suspension and, once gas evolution had ceased, the reaction mixture was warmed to rt and stirred for 18 h. The reaction was quenched by the addition of wet diethyl ether (200 mL) and filtered. The collected solids were washed with diethyl ether (2 × 100 mL) then dried under vacuum. The solids were then suspended in ethanol (130 mL), and concentrated hydrochloric acid (26 mL) was slowly added to the suspension. The suspension was stirred at rt for 2 h then filtered to remove salt. The resulting solution was concentrated *in vacuo* then dissolved in ethanol (210 mL) and water (90 mL), and the pH adjusted to 3 with aqueous 6 M sodium hydroxide (∼40 mL). Cyanamide (3.27 g, 77.9 mmol, 2.0 eq) was added to the solution. The reaction solution was heated to 100°C for 2 h, then cooled to rt and concentrated *in vacuo*. The residue was dissolved in ethyl acetate (200 mL), and saturated aqueous potassium carbonate solution (100 mL) was added. This mixture was extracted with ethyl acetate (3 × 100 mL). The combined organic fractions were washed with brine (300 mL), dried (Na_2_SO_4_), filtered, and concentrated *in vacuo* to yield the title compound (**S16**) (5.18 g, 79%) as a pale-yellow solid which slowly decomposes at rt. Further purification or re-purification could be achieved by trituration with minimal chloroform but was usually unnecessary. *R*_*f*_ 0.27 (5% ethanol: chloroform); mp 153–155°C (from methanol) [lit. (O’Connor et al., 2015) 130–133°C (from water)]; ^1^H NMR (500 MHz, CDCl_3_) δ_H_ 7.44 (1H, s), 4.39 (2H, br s), 4.26 (2H, q, *J* 7.1), 3.67 (3H, s), 1.33 (3H, t, *J* 7.1); LRMS *m/z* (ESI^+^) 170 ([M+H]^+^, 100%), 142 (30%). The spectroscopic data are consistent with literature (Calder et al., 2020; O'Connor et al., 2015; Parveen et al. 1999).

##### Ethyl (1-methyl-2-nitro-imidazol-5-yl)carboxylate

A solution of ethyl (2-amino-1-methyl-imidazol-5-yl)carboxylate (**S16**) (1.73 g, 10.2 mmol, 1.0 eq) in glacial acetic acid (18 mL) was added, dropwise at 0°C, to a solution of sodium nitrite (7.06 g, 102 mmol, 10 eq) in water (9 mL). The solution was stirred at 0°C for 1 h then warmed to rt over 3 h. The solution was extracted with dichloromethane (3 × 50 mL). The combined organic fractions were washed with a saturated aqueous solution of sodium sulfite (100 mL), and brine (100 mL) then dried (MgSO_4_), filtered, and concentrated *in vacuo*. The residue was dissolved in dichloromethane (100 mL) and filtered through a short pad of silica to yield ethyl (1-methyl-2-nitro-imidazol-5-yl)carboxylate (1.46 g, 72%) as an off-white solid. *R*_*f*_ 0.27 (100% dichloromethane); mp 51–53°C (from dichloromethane) [lit. (O’Connor et al., 2015) 56–58°C (from dichloromethane)]; ^1^H NMR (400 MHz, CDCl_3_) δ_H_ 7.71 (1H, s), 4.37 (2H, q, *J* 7.2), 4.32 (3H, s), 1.38 (3H, t, *J* 7.2); LRMS *m/z* (ESI^+^) 200 ([M+H]^+^, 100%), 172 (65%). The spectroscopic data are consistent with literature (Calder et al., 2020; O'Connor et al., 2015; Parveen et al., 1999).

##### (1-Methyl-2-nitroimidazol-5-yl)methanol

A solution of sodium borohydride (0.160 g, 4.22 mmol, 3.0 eq) in dry ethanol (5.3 mL) was added dropwise to a solution of ethyl 1-methyl-2-nitroimidazole-5-carboxylate (0.280 g, 1.41 mmol, 1.0 eq) in dry tetrahydrofuran (7.0 mL) with vigorous stirring at 0°C. The reaction was stirred at 0°C for 3 h then slowly added to a stirred mixture of diethyl ether (100 mL) and wet methanol (100 mL) at 0°C. The resulting solution was stirred at 0°C for 30 min then gradually acidified to pH 5 with aqueous 2 M hydrochloric acid. The solution was concentrated *in vacuo* to give a mostly aqueous solution. The residue was then extracted with ethyl acetate (5 × 50 mL), the combined organic components were dried (Na_2_SO_4_), filtered, and concentrated *in vacuo* to yield (1-methyl-2-nitro-imidazol-5-yl)methanol (0.176 g, 80%) as a pale-yellow solid. *R*_*f*_ 0.37 (5% ethanol:chloroform); mp 126–130°C (from chloroform) [lit. (O'Connor et al., 2015) 141–143°C (from ethyl acetate)]; ^1^H NMR (400 MHz, CD_3_OD) δ_H_ 7.10 (1H, s), 4.66 (2H, s), 4.04 (3H, s); LRMS *m/z* (ESI^+^) 180 ([M+Na]^+^, 45%), 170 (80%), 158 ([M+H]^+^, 100%), 113 (31%). The spectroscopic data are consistent with literature (Calder et al., 2020; O'Connor et al., 2015; Parveen et al., 1999).

##### (1-Methyl-2-nitro-imidazol-5-yl)methyl chloride (S17)

Methanesulfonyl chloride (74 μL, 0.96 mmol, 1.5 eq) was added dropwise to a stirred solution of (1-methyl-2-nitro-imidazol-5-yl)methanol (0.10 g, 0.64 mmol, 1.0 eq) in pyridine (1.3 mL) and stirred at rt for 3 h then concentrated *in vacuo*. Purification by filtration through a short pad of silica, eluting with 50% ethyl acetate:petroleum ether, yielded the title compound (**S17**) (75 mg, 67%) as a colorless solid. *R*_*f*_ 0.43 (50% ethyl acetate:petroleum ether). mp 68–77°C (from dichloromethane) [lit. (O'Connor et al., 2015) 87–90°C (from ethyl acetate)]; ^1^H NMR (400 MHz, CDCl_3_) δ_H_ 7.19 (1H, s), 4.62 (2H, s), 4.07 (3H, s); LRMS *m/z* (ESI^+^) 176 ([M+H]^+^, 100%), 140 (43%).The spectroscopic data are consistent with literature (Calder et al., 2020; O'Connor et al., 2015; Parveen et al., 1999).

##### (*E*)-Methyl-3-(4-[*tert*-butyloxycarbonyl-(2-[1-(*tert*-butyloxycarbonyl)-2-methyl-1*H*-indol-3-ylethyl]amino)methyl]phenyl)prop-2-enoate **(4)**

Di-*tert*-butyl dicarbonate (1.0 mL, 4.2 mmol, 3.0 eq) was added to a solution of **3** (0.48 g, 1.4 mmol, 1.0 eq) and *N,N*-dimethyl-4-aminopyridine (8.0 mg, 0.066 mmol, 0.05 eq) in tetrahydrofuran (14 mL) and stirred at rt for 18 h. The reaction was quenched with water (20 mL) and extracted with ethyl acetate (2 × 50 mL). The organic components were combined and washed with water (50 mL), brine (50 mL) then dried (MgSO_4_), filtered, and concentrated *in vacuo*. Purification using column chromatography, eluting with 0–60% ethyl acetate:petroleum ether, yielded the title compound (**4**) (0.62 g, 82%) as a colorless oil. *Rf* 0.71 (50% ethyl acetate:petroleum ether); ${\bar{\nu }}_{\max}$ (thin film)/cm^−1^ 2976, 1722, 1690, 1478, 1368, 1323, 1254, 1168, 1137; ^1^H NMR at 363 K (500 MHz, (D_6_-DMSO) δ_H_ 8.00 (1H, d, *J* 8.1), 7.66–7.57 (3H, m), 7.42 (1H, d, *J* 7.2), 7.25 (2H, d, *J* 7.8), 7.23–7.13 (2H, m), 6.53 (1H, d, *J* 16.1), 4.42 (2H, s), 3.74 (3H, s), 3.33 (2H, t, *J* 7.1), 2.84 (2H, t, *J* 7.1), 2.46 (3H, s), 1.64 (9H, s), 1.34 (9H, s); ^13^C NMR at 363 K (126 MHz, (D_6_-DMSO) δ_C_ 166.0, 154.5, 149.6, 143.5, 140.8, 134.9, 132.9, 132.6, 129.1, 127.8, 127.4, 122.7, 121.8, 117.3, 117.2, 114.8, 114.4, 83.1, 78.5, 50.8, 49.6, 46.1, 27.5, 27.4, 22.1, 12.7; HRMS *m/z* (ESI^+^) Found: 549.2953, C_32_H_41_N_2_O_6_ requires [M+H]^+^ 549.2959; LRMS (ESI^+^) 338 ([M+2Na+2MeCN]^2+^, 23%), 225 ([M+2Na+H+2MeCN]^3+^, 100%), 202 (26%), 123 (71%); HPLC Method C, Retention time - 15.0 min, 99%.

##### (*E*)-3-(4-[(*tert*-Butyloxycarbonyl-[2-(1-[*tert*-butyloxycarbonyl]-2-methyl-1*H*-indol-3-yl)ethyl]amino)methyl]phenyl)prop-2-enoic acid **(5)**

A solution of lithium hydroxide (2.05 g, 85.6 mmol, 10.0 eq) in water (57 mL) was slowly added to a stirred solution of **4** (4.70 g, 8.56 mmol, 1.0 eq) in tetrahydrofuran (57 mL) and methanol (57 mL) at 0°C. The reaction mixture was stirred at rt for 6 h then diluted with ethyl acetate (250 mL) and quenched with aqueous 1 M hydrochloric acid (200 mL). The reaction was extracted with ethyl acetate (3 × 200 mL) and the organic components were combined and washed with water (300 mL), brine (300 mL) then dried (MgSO_4_), filtered, and concentrated *in vacuo* to yield the title compound (**5**) (4.54 g, 99%) as a colorless fluffy, foamy solid. *R*_*f*_ 0.61 (100% ethyl acetate); mp 94–96°C (from THF); ${\bar{\nu }}_{\max}$ (thin film)/cm^−1^ 2976, 2930, 1726, 1687, 1410, 1366, 1258, 1136, 1116; ^1^H NMR at 363 K (500 MHz, (D_6_-DMSO) δ_H_ 8.00 (1H, d, *J* 8.0), 7.57 (2H, d, *J* 7.7), 7.55 (1H, d, *J* 15.9), 7.42 (1H, d, *J* 7.4), 7.24 (2H, d, *J* 7.7), 7.23–7.13 (2H, m), 6.44 (1H, d, *J* 15.9), 4.41 (2H, s), 3.33 (2H, t, *J* 7.2), 2.84 (2H, t, *J* 7.2), 2.46 (3H, s), 1.64 (9H, s), 1.34 (9H, s); ^13^C NMR at 363 K (126 MHz, (D_6_-DMSO) δ_C_ 166.8, 154.5, 149.6, 142.7, 140.4, 134.9, 132.94, 132.93, 129.1, 127.6, 127.4, 122.7, 121.8, 118.9, 117.2, 114.8, 114.4, 83.2, 78.5, 49.6, 46.1, 27.5, 27.4, 22.1, 12.8; HRMS *m/z* (ESI^-^) Found: 533.2648, C_31_H_37_N_2_O_6_ requires [M−H]^−^ 533.2657; LRMS (ESI^−^) 533 ([M−H]^−^, 100%); HPLC Method A, Retention time - 13.1 min, 99%.

##### (*E*)-*tert*-Butyl-3-(2-((4-(3-((benzyloxy)amino)-3-oxoprop-1-en-1-yl)benzyl)(*tert*-butoxycarbonyl)amino)ethyl)-2-methyl-1*H*-indole-1-carboxylate **(6)**

PyBOP (0.11 g, 0.21 mmol, 1.1 eq) was added to a solution of **5** (0.10 g, 0.19 mmol, 1.0 eq) and triethylamine (0.10 mL, 0.75 mmol, 4.0 eq) in dry tetrahydrofuran (1.9 mL). The reaction mixture was stirred for 15 min at rt before *O-*(benzyl)hydroxylamine hydrochloride (36 mg, 0.22 mmol, 1.2 eq) was added. Stirring was continued at rt for 18 h, the reaction mixture was diluted with ethyl acetate (20 mL) and quenched with aqueous 1 M solution of hydrochloric acid (10 mL). The reaction mixture was extracted with ethyl acetate (20 mL), the organic components were washed with a saturated solution of sodium hydrogen carbonate (40 mL), water (40 mL), brine (40 mL), dried (MgSO_4_), filtered, and concentrated *in vacuo*. Purification using column chromatography, eluting with 0–5% ethanol:chloroform, then a second purification using column chromatography, eluting with 0–50% ethyl acetate:petroleum ether, yielded the title compound (**6**) (87 mg, 73%) as a colorless solid. *R*_*f*_ 0.45 (50% ethyl acetate:petroleum ether); mp 71–74°C (from dichloromethane:hexane); ${\bar{\nu }}_{\max}$ (thin film)/cm^−1^ 3192, 2976, 1728, 1687, 1660, 1514, 1459, 1366, 1322, 1252, 1158, 1136, 1117, 1046; ^1^H NMR at 363 K (500 MHz, D_6_-DMSO ) δ_H_ 11.71 (1H, s), 8.82 (1H, d, *J* 7.8), 8.30 (2H, d, *J* 8.4), 8.30 (1H, d, *J* 15.8), 8.29–8.13 (6H, m), 8.06 (2H, d, *J* 8.0), 8.02 (1H, ddd, *J* 7.8, 7.3, 1.1), 7.98 (1H, ddd, *J* 7.5, 7.3, 1.3), 7.30 (1H, d, *J* 15.8), 5.71 (2H, s), 5.22 (2H, s), 4.15 (2H, t, *J* 7.2), 3.65 (2H, t, *J* 7.2), 3.27 (3H, s), 2.45 (9H, s), 2.16 (9H, s); ^13^C NMR at 363 K (126 MHz, D_6_-DMSO) δ_C_ 163.3,^∗^ 154.4, 149.5, 139.9, 138.9, 135.7, 134.9, 133.3, 132.9, 129.1, 128.2, 127.7, 127.6, 127.4, 127.1, 122.7, 121.7, 118.1, 117.1, 114.8, 114.4, 83.1, 78.5, 76.9, 49.5, 46.1, 27.5, 27.4, 22.1, 12.7; HRMS *m/z* (ESI^+^) Found: 640.33788, C_38_H_46_N_3_O_6_ requires [M+H]^+^ 640.33811; LRMS *m/z* (ESI^+^) 662 ([M+Na]^+^, 16%), 606 (34%), 562 (20%), 506 (38%), 461 (44%), 423 (38%), 405 (13%), 361 (100%), 317 (26%), 300 (25%), 282 (39%), 266 (10%); HPLC Method A, Retention time - 13.3 min, >99%. ^∗^Signal at 163.3 observed by ^1^H-^13^C HMBC correlation see supplemental information for details.

##### (*E*)-*tert*-Butyl-3-(2-((*tert*-butoxycarbonyl)(4-(3-(((4-nitrobenzyl)oxy)amino)-3-oxoprop-1-en-1-yl)benzyl)amino)ethyl)-2-methyl-1*H*-indole-1-carboxylate **(7)**

PyBOP (48 mg, 0.093 mmol, 1.1 eq) was added to a solution of **5** (45 mg, 0.084 mmol, 1.0 eq) and triethyl amine (35 μL, 0.25 mmol, 3.0 eq) in dry tetrahydrofuran (1.0 mL). The reaction mixture was stirred for 15 min at rt before *O*-(4-nitrobenzyl)-hydroxylamine (**S7**) (17 mg, 0.10 mmol, 1.2 eq) was added. Stirring was continued at rt for 18 h, the reaction mixture was diluted with ethyl acetate (20 mL) and quenched with an aqueous 1 M solution of hydrochloric acid (10 mL). The reaction mixture was extracted with ethyl acetate (20 mL), the organic components were washed with a saturated aqueous solution of sodium hydrogen carbonate (40 mL), water (40 mL), brine (40 mL), dried (MgSO_4_), filtered, and concentrated *in vacuo*. Purification using column chromatography, eluting with 0–10% ethanol:chloroform, yielded the title compound (**7**) (54 mg, 92%) as a pale-yellow solid. *R*_*f*_ ; mp 90–92°C (from DMSO:H_2_O); ${\bar{\nu }}_{\max}$ (thin film)/cm^−1^ 3199, 2978, 1728, 1688, 1523, 1460, 1346, 1323, 1159, 1137, 1117; ^1^H NMR at 363 K (500 MHz, (D_6_-DMSO) δ_H_ 11.04 (1H, br s), 8.22 (2H, d, *J* 8.3), 8.00 (1H, d, *J* 8.1), 7.72 (2H, d, *J* 8.3), 7.50 (2H, d, *J* 7.9), 7.50 (1H, d, *J* 15.9) 7.41 (1H, d, *J* 7.6), 7.24 (2H, d, *J* 7.9), 7.23–7.13 (2H, m), 6.46 (1H, d, *J* 15.9), 5.05 (2H, s), 4.41 (2H, s), 3.33 (2H, t, *J* 7.4), 2.84 (2H, t, *J* 7.4), 2.46 (3H, s), 1.64 (9H, s), 1.35 (9H, s); ^13^C NMR at 363 K (126 MHz, D_6_-DMSO) δ_C_ 163.5,^∗^ 154.4, 149.5, 147.1, 143.4, 140.0, 139.2, 134.9, 133.2, 132.9, 129.1, 128.9, 127.4, 127.2, 122.8, 122.7, 121.7, 117.8, 117.1, 114.8, 114.4, 83.1, 78.5, 75.6, 49.6,^∗^ 46.1, 27.5, 27.4, 22.1, 12.7; HRMS *m/z* (ESI^−^) Found: 683.3083, C_38_H_43_N_4_O_8_ requires [M−H]^−^ 683.3086; LRMS (ESI^−^) 683 ([M−H]^−^, 100%); HPLC Method B, Retention time - 13.3 min, 98%. ^∗^Signal at 163.5 observed by ^1^H-^13^C HMBC correlation, signal at 49.6 observed by ^1^H-^13^C HSQC correlation see supplemental information for details.

##### (*E*)-*tert*-Butyl-3-(2-((*tert*-butoxycarbonyl)(4-(3-(((5-nitrothiophen-2-yl)methoxy)amino)-3-oxoprop-1-en-1-yl)benzyl)amino)ethyl)-2-methyl-1*H*-indole-1-carboxylate **(8)**

PyBOP (0.15 g, 0.29 mmol, 1.1 eq) was added to a solution of **5** (0.14 g, 0.26 mmol, 1.0 eq) and triethylamine (0.11 mL, 0.79 mmol, 3.0 eq) in dry tetrahydrofuran (2.6 mL). The reaction mixture was stirred for 15 min at rt before *O*-(5-nitrothiophen-2-yl)methylene)hydroxylamine (**S10**) (55 mg, 0.32 mmol, 1.2 eq) was added. Stirring was continued at rt for 18 h, the reaction mixture was diluted with ethyl acetate (20 mL) and quenched with aqueous 1 M solution of hydrochloric acid (10 mL). The reaction mixture was extracted with ethyl acetate (20 mL), the organic components were washed with a saturated solution of sodium hydrogen carbonate (40 mL), water (40 mL), brine (40 mL), dried (MgSO_4_), filtered, and concentrated *in vacuo*. Purification using column chromatography, eluting with 2.5% ethanol:chloroform, yielded the title compound (**8**) (0.15 g, 84%) as a pale yellow solid. *R*_*f*_ 0.59 (5% ethanol:chloroform); mp 80–82°C (from hexane:dichloromethane); ${\bar{\nu }}_{\max}$ (thin film)/cm^−1^ 3205, 2977, 1728, 1684, 1460, 1336, 1158, 1138; ^1^H NMR Major rotamer at room temperature reported (400 MHz, CDCl_3_) δ_H_ 8.41 (1H, br s), 8.07 (1H, d, *J* 8.2), 7.82 (1H, d, *J* 4.1), 7.71 (1H, d, *J* 15.9), 7.48–6.97 (8H, m), 6.35 (1H, d, *J* 15.9), 5.12 (2H, s), 4.43 (2H, s), 3.30 (2H, s), 2.82 (2H, s), 2.47 (3H, s), 1.67 (9H, s), 1.46 (9H, s); ^13^C NMR spectroscopic data not collected due to instability of compound at prolonged high temperatures;. HRMS *m/z* (ESI^−^) Found: 689.26561, C_36_H_41_O_8_N_4_S requires [M−H]^−^ 689.26506; LRMS (ESI^−^) 689 ([M−H]^−^, 45%), 532 (7%), 432 (5%), 144 (100%); HPLC Method B, Retention time - 13.5 min, 96%.

##### (*E*)-*tert*-Butyl-3-(2-((*tert*-butoxycarbonyl)(4-(3-(((6-nitroquinolin-5-yl)methoxy)amino)-3-oxoprop-1-en-1-yl)benzyl)amino)ethyl)-2-methyl-1H-indole-1-carboxylate **(9)**

PyBOP (85 mg, 0.16 mmol, 1.1 eq) was added to a solution of **5** (79 mg, 0.15 mmol, 1.0 eq) and triethylamine (62 μL, 0.45 mmol, 3.0 eq) in dry tetrahydrofuran (1.5 mL). The reaction mixture was stirred for 15 min at rt before *O-*(6-nitroquinolin-5-yl)-hydroxylamine (**S14**) (39 mg, 0.18 mmol, 1.2 eq) was added. Stirring was continued at rt for 18 h, the reaction mixture was diluted with ethyl acetate (20 mL) and quenched with aqueous 1 M solution of hydrochloric acid (10 mL). The reaction mixture was extracted with ethyl acetate (20 mL), the organic components were washed with a saturated solution of sodium hydrogen carbonate (40 mL), water (40 mL), brine (40 mL), dried (MgSO_4_), filtered, and concentrated *in vacuo*. Purification using column chromatography, eluting with 0–10% ethanol:chloroform, then a second purification by column chromatography, eluting with 0–100% ethyl acetate:petroleum ether, yielded the title compound (**9**) (66 mg, 60%) as a colorless solid. *R*_*f*_ 0.44 (80% ethyl acetate:petroleum ether); mp 106–110°C (from dichloromethane:hexane); ${\bar{\nu }}_{\max}$ (thin film)/cm^−1^ 3189, 2976, 2930, 1731, 1687, 1634, 1531, 1459, 1366, 1323, 1259, 1160, 1138, 1117; ^1^H NMR at 363 K (500 MHz, (D_6_-DMSO) δ_H_ 11.21 (1H, s), 9.21 (1H, d, *J* 8.7), 9.11 (1H, dd, *J* 4.1, 1.6), 8.25 (1H, d, *J* 9.1), 8.15 (1H, d, *J* 9.1), 8.00 (1H, d, *J* 8.1), 7.79 (1H, dd, *J* 8.7, 4.1), 7.51 (1H, d, *J* 15.7), 7.48 (2H, d, *J* 7.8), 7.42 (1H, d, *J* 7.5), 7.25 (2H, d, *J* 7.8), 7.23–7.14 (2H, m), 6.42 (1H, d, *J* 15.7), 5.54 (2H, s), 4.41 (2H, s), 3.33 (2H, t, *J* 7.2), 2.84 (2H, t, *J* 7.2), 2.46 (3H, s), 1.64 (9H, s), 1.35 (9H, s); ^13^C NMR at 363 K (126 MHz, (D_6_-DMSO) δ_C_ 163.8,^∗^ 154.4, 152.6, 149.6, 148.1, 147.9, 140.1, 139.4, 134.9, 134.8, 133.2, 132.9, 131.7, 129.1, 127.5, 127.2, 127.0, 125.9, 122.8, 122.72, 122.68, 121.8, 117.5, 117.1, 114.8, 114.4, 83.1, 78.5, 67.7, 49.6,^∗^ 46.1, 27.5, 27.4, 22.1, 12.7; HRMS *m/z* (ESI^+^) Found: 736.33361, C_41_H_46_N_5_O_8_ requires [M+H]^+^ 736.33409; LRMS (ESI^−^) 770 ([M+Cl]^−^, 49%), 734 ([M−H]^−^, 81%), 354 (38%), 185 (100%), 141 (96%). HPLC Method A, Retention time - 14.0 min, 98%. ^∗^Signals at 163.8 and 49.6 observed by ^1^H-^13^C HMBC correlation see supplemental information for details.

##### (*E*)-*N*-(4-(3-((Benzyloxy)amino)-3-oxoprop-1-en-1-yl)benzyl)-2-(2-methyl-1*H*-indol-3-yl)ethan-1-aminium 2,2,2-trifluoroacetate (Bn-Pano, 10)

Trifluoroacetic acid (1.6 mL, 20% *v/v*) was added dropwise to a rapidly stirred solution of **6** (50 mg, 78 μmol, 1.0 eq) and triisopropylsilane (3.4 μL, 16 μmol, 0.2 eq) in dichloromethane (7.8 mL). The reaction mixture was stirred for 60 mins then diluted with toluene (1 mL) and dried by azeotroping with toluene (3 × 1 mL) *in vacuo*. Purification using column chromatography, eluting with 10% ethanol:chloroform, yielded the title compound (**10**) (34 mg, 79%) as a colorless solid. *Rf* 0.25 (10% ethanol: chloroform); Mp 102–104°C (Decomposed from methanol); ${\bar{\nu }}_{\max}$ (thin film)/cm^−1^; 3391, 3268, 3031, 2950, 2851, 162, 1624, 1461, 1341, 1202, 1046, 978; ^1^H NMR (500 MHz, CD_3_OD) δ_H_ 7.61 (1H, d, *J* 16.1), 7.60 (2H, d, *J* 7.7), 7.46 (2H, d, *J* 8.2), 7.41 (1H, d, *J* 8.1), 7.43–7.33 (4H, m), 7.25 (1H, d, *J* 7.8), 7.03 (1H, ddd, *J* 8.1, 7.1, 1.2), 6.96 (1H, ddd, *J* 7.8, 7.1, 1.1), 6.46 (1H, d, *J* 16.1), 4.93 (2H, s), 4.16 (2H, s), 3.18–3.12 (2H, m), 3.12–3.06 (2H, m), 2.39 (3H, s); ^13^C NMR (126 MHz, CD_3_OD) δ_C_ 165.8, 141.5, 137.2, 137.0, 136.9, 135.7, 133.8, 131.3, 130.3, 129.7, 129.53, 129.46, 129.3, 121.8, 119.8, 119.3, 118.0, 111.6, 106.2, 79.2, 52.1, 49.1,^∗^ 22.7, 11.3; HRMS *m/z* (ESI^+^) Found: 440.23323 C_28_H_30_N_3_O_2_ requires [M+H]^+^ 440.23325; LRMS (ESI^+^) 440 ([M+H]^+^, 63%), 423 (28%), 150 (100%); HPLC Method A, Retention time - 8.0 min, 99%. All batches used for biological testing were >95% purity. ^∗^Signal at 49.1 observed by ^1^H-^13^C HMBC correlation see supplemental information for details.

##### (*E*)-2-(2-Methyl-1*H*-indol-3-yl)-*N*-(4-(3-(((4-nitrobenzyl)oxy)amino)-3-oxoprop-1-en-1-yl)benzyl)ethan-1-aminium 2,2,2-trifluoroacetate (NB-Pano, 11)

Trifluoroacetic acid (0.16 mL, 20% *v/v*) was added dropwise to a rapidly stirred solution of **7** (22 mg, 0.32 mmol, 1.0 eq) and triisopropylsilane (1.3 μL, 6.4 μmol, 0.2 eq) in dichloromethane (0.8 mL). The reaction mixture was stirred for 75 mins then diluted with toluene (1 mL) and dried by azeotroping with toluene (3 × 1 mL) *in vacuo*. Purification using column chromatography, eluting with 2:5:80 water:isopropanol:ethyl acetate, to yield the title compound (**11**) (13 mg, 69%) as an off-white solid. *R*_*f*_ 0.55 (1:3:40 water:isopropanol:ethyl acetate), *R*_*f*_ 0.21 (10% ethanol:chloroform); mp 98–100°C (dec.; from ethanol); ${\bar{\nu }}_{\max}$ (thin film)/cm^−1^ 3399, 3293, 2922, 2852, 1669, 1629, 1520, 1462, 1345, 1202, 1014; ^1^H NMR (500 MHz, CD_3_OD) δ_H_ 8.25 (2H, d, *J* 8.7), 7.70 (2H, d, *J* 8.7), 7.57 (1H, d, *J* 15.8), 7.50 (2H, d, *J* 8.2), 7.38 (1H, ddd, *J* 7.9, 1.1, 1.0), 7.32 (2H, d, *J* 8.2), 7.23 (1H, ddd, *J* 8.1, 1.0, 1.0), 7.00 (1H, ddd, *J* 8.1, 7.0, 1.1), 6.93 (1H, ddd, *J* 7.9, 7.0, 1.0), 6.40 (1H, d, *J* 15.8), 5.06 (2H, s), 3.90 (2H, s), 3.01–2.91 (4H, m), 2.35 (3H, s); ^13^C NMR (126 MHz, CD_3_OD) δ_C_ 166.1, 149.2, 145.1, 141.0, 137.8, 137.2, 136.4, 133.6, 130.8, 130.5, 129.5, 129.2, 124.5, 121.6, 119.7, 119.3, 118.1, 111.5, 107.0, 77.3, 52.7, 49.4, 23.4, 11.3; HRMS *m/z* (ESI^+^) Found: 485.21835, C_28_H_29_N_4_O_4_ requires [M+H]^+^ 485.21833; LRMS (ESI^+^) 485 ([M+H]^+^, 100%), 468 (4%), 349 (4%), 304 (3%), 216 (4%), 130 (2%); HPLC Method B, Retention time - 8.4 min, 98–99% at 3 wavelengths, mean purity 98%. All batches used for biological testing were >95% purity.

##### (*E*)-2-(2-Methyl-1*H*-indol-3-yl)-*N*-(4-(3-(((5-nitrothiophen-2-yl)methoxy)amino)-3-oxoprop-1-en-1-yl)benzyl)ethan-1-aminium 2,2,2-trifluoroacetate (NT-Pano, 12)

Trifluoroacetic acid (0.25 mL, 20% *v/v*) was added dropwise to a rapidly stirred solution of (**8**) (9 mg, 0.01 mmol, 1.0 eq) and triisopropylsilane (0.5 μL, 3 μmol, 0.2 eq) in dichloromethane (1 mL). The reaction mixture was stirred for 65 mins then diluted with toluene (1 mL) and dried by azeotroping with toluene (3 × 1 mL) *in vacuo*. Purification using column chromatography, eluting with 1:2:10 H_2_O:isopropyl alcohol:ethyl acetate, yielded the title compound (**12**) (5 mg, 64%) as a pale-yellow solid. *R*_*f*_ 0.48 (1:2:10 H_2_O: isopropyl alcohol:ethyl acetate), 0.38 (20% ethanol:chloroform); mp 108–110°C (dec.; from H_2_O/isopropyl alcohol); ${\bar{\nu }}_{\max}$ (thin film)/cm^−1^ 3198, 2924, 1694, 1503, 1337, 1179, 1136; ^1^H NMR (500 MHz, CD_3_OD) δ_H_ 7.90 (1H, d, *J* 4.1), 7.60 (1H, d, *J* 15.8), 7.56 (2H, d, *J* 8.2), 7.41 (2H, d, *J* 8.2), 7.40 (1H, ddd, *J* 7.9, 1.2, 0.9), 7.24 (1H, ddd, *J* 8.1, 1.0, 0.9), 7.18 (1H, d, *J* 4.1), 7.01 (1H, ddd, *J* 8.1, 7.0, 1.2), 6.95 (1H, ddd, *J* 7.9, 7.0, 1.0), 6.47 (1H, d, *J* 15.8), 5.12 (2H, s), 4.05 (2H, s), 3.10–3.00 (4H, m), 2.37 (3H, s); ^13^C NMR (126 MHz, CD_3_OD) δ_C_ 166.2, 153.7, 147.9, 141.5, 137.6, 137.2, 136.4, 133.6, 130.9, 129.6, 129.5, 129.3, 128.9, 121.7, 119.7, 119.0, 118.1, 111.5, 106.9, 72.7, 52.6, 49.1,^∗^ 23.3, 11.3; HRMS *m/z* (ESI^+^) Found: 491.17457 C_26_H_27_N_4_O_4_S requires [M+H]^+^ 491.17475; LRMS (ESI^+^) 491 ([M+H]^+^, 58%), 349 (22%), 301 (41%), 107 (100%); HPLC Method B, Retention time - 8.1 min, 93–98%, mean purity 95%. All batches used for biological testing were >95% purity. ^∗^Signal at 49.1 observed by ^1^H-^13^C HMBC correlation see supplemental information for details.

##### (*E*)-2-(2-Methyl-1*H*-indol-3-yl)-*N*-(4-(3-(((6-nitroquinolin-5-yl)methoxy)amino)-3-oxoprop-1-en-1-yl)benzyl)ethan-1-aminium-2,2,2-trifluoroacetate (NQ-Pano, 13)

Trifluoroacetic acid (0.98 mL, 20% *v/v*) was added dropwise to a rapidly stirred solution of **9** (36 mg, 0.49 mmol, 1.0 eq) and triisopropylsilane (2.0 μL, 9.8 μmol, 0.2 eq) in dichloromethane (4.9 mL). The reaction mixture was stirred for 55 mins then diluted with toluene (1 mL) and dried by azeotroping with toluene (3 × 1 mL) *in vacuo*. Purification using column chromatography, eluting with 0–10% ethanol: chloroform, yielded the title compound (**13**) (31 mg, 83%) as a yellow solid. *R*_*f*_ 0.15 (10% ethanol:chloroform); mp 118–120°C (dec.; from methanol); ${\bar{\nu }}_{\max}$ (thin film)/cm^−1^; 3398, 3198, 3054, 2921, 2852, 1665, 1626, 1530, 1462, 1343, 1047; ^1^H NMR (500 MHz, CD_3_OD) δ_H_ 9.29 (1H, d, *J* 8.8), 9.07 (1H, d, *J* 3.8), 8.25 (1H, d, *J* 9.2), 8.14 (1H, d, *J* 9.2), 7.80 (1H, dd, *J* 8.8, 3.8), 7.58 (1H, d, *J* 15.8), 7.48 (2H, d, *J* 7.9), 7.39 (1H, ddd, *J* 7.9, 1.1, 0.9), 7.29 (2H, d, *J* 7.9), 7.24 (1H, ddd, *J* 8.1, 1.0, 0.9), 7.01 (1H, ddd, *J* 8.1, 7.0, 1.1), 6.93 (1H, ddd, *J* 7.9, 7.0, 1.0), 6.38 (1H, d, *J* 15.8), 5.62 (2H, s), 3.83 (2H, s), 2.95 (2H, t, *J* 7.1), 2.87 (2H, t, *J* 7.1), 2.36 (3H, s); ^13^C NMR (126 MHz, CD_3_OD) δ_C_ 166.7, 154.0, 150.5, 149.6, 142.1, 141.9, 137.5, 137.2, 135.2, 133.2, 132.7, 130.1, 129.8, 129.4, 129.1, 127.8, 125.0, 124.4, 121.4, 119.5, 118.3, 118.0, 111.3, 108.4, 69.4, 53.6, 50.1, 24.6, 11.4; HRMS *m/z* (ESI^+^) Found: 536.22894 C_31_H_30_N_5_O_4_ requires [M+H]^+^ 536.22923; LRMS (ESI^+^) 536 ([M+H]^+^, 7%), 332 (6%), 273 (6%), 158 (100%), 144 (35%), 115 (36%); HPLC Method B, Retention time - 7.7 min, 98–99%, mean purity 99%. All batches used for biological testing were >95% purity.

##### (*E*)-*tert*-Butyl-3-(2-((*tert*-butoxycarbonyl)(4-(3-(hydroxyamino)-3-oxoprop-1-en-1-yl)benzyl)amino)ethyl)-2-methyl-1*H*-indole-1-carboxylate **(14)**

1, 1’-Carbonyl diimidazole (59 mg, 0.36 mmol, 1.5 eq) was added to a solution **5** (0.13 g, 0.24 mmol, 1.0 eq) in dry tetrahydrofuran (0.8 mL) and stirred at rt for 4 h. Hydroxylamine hydrochloride (34 mg, 0.49 mmol, 2.0 eq) was added and stirring was continued for a further 19 h. The reaction was quenched by addition of a 1 M aqueous solution of hydrochloric acid (10 mL) then extracted with ethyl acetate (2 × 20 mL). The organic components were washed with brine (50 mL), dried (Na_2_SO_4_), filtered, and concentrated *in vacuo*. Purification by column chromatography (elution with 0–10% ethanol:chloroform) yielded the title compound (**14**) (98 mg, 74%) as a yellow solid, which was used in the next step without further purification. *Rf* 0.32 (10% ethanol:chloroform); mp 104–106°C (from ethanol); ${\bar{\nu }}_{\max}$ (thin film)/cm^−1^ 3204, 2976, 2930, 1727, 1687, 1460, 1366, 1323, 1251, 1159, 1137, 1117, 1048; ^1^H NMR at 363 K (500 MHz, D_6_-DMSO) δ_H_ 10.44 (1H, s), 8.21 (1H, s), 8.00 (1H, d, *J* 8.0), 7.49 (2H, d, *J* 8.1), 7.47–7.38 (2H, m), 7.24 (2H, d, *J* 8.1), 7.22–7.14 (2H, m), 6.51 (1H, d, *J* 15.5), 4.41 (2H, s), 3.33 (2H, t, *J* 7.2), 2.83 (2H, t, *J* 7.2), 2.46 (3H, s), 1.64 (9H, s), 1.35 (9H, s); ^13^C NMR∗ at 363 K (126 MHz, D_6_-DMSO) δ_C_ 154.5, 149.6, 139.6, 134.9, 133.6, 132.9, 129.1, 127.5, 127.0, 122.7, 121.8, 118.6, 117.1, 114.8, 114.4, 83.1, 78.7, 78.5, 49.6,^∗^ 46.0, 27.5, 27.4, 22.1, 12.7; HRMS *m/z* (ESI^+^) Found: 550.29100, C_31_H_40_N_3_O_6_ requires [M+H]^+^ 550.29116; LRMS (ESI^+^) 572 ([M+Na]^+^, 10%), 367 (18%), 277 (9%), 248 (18%), 225 (100%), 186 (57%); HPLC Method A, Retention time - 11.7 min, 99%. ^∗^Signal for hydroxamic acid carbonyl quaternary carbon not observed signal at 49.6 observed by ^1^H-^13^C HMBC correlation see supplemental information for details.

##### (*E*)-*tert*-Butyl-3-(2-((*tert*-butoxycarbonyl)(4-(3-(((1-methyl-2-nitro-1*H*-imidazol-5-yl)methoxy)amino)-3-oxoprop-1-en-1-yl)benzyl)amino)ethyl)-2-methyl-1*H*-indole-1-carboxylate

A 60% dispersion of sodium hydride in mineral oil (34 mg, 0.85 mmol, 1.5 eq) was added to a solution of **14** (0.31 g, 0.57 mmol, 1.0 eq) in *N,N-*dimethylformamide (1.5 mL) at −5°C and stirred for 10 min. (1-methyl-2-nitro-imidazol-5-yl)methyl chloride (**S17**) (0.11 g, 0.62 mmol, 1.1 eq) was added, then the reaction mixture was warmed to rt and stirred for 18 h. The reaction was diluted with ethyl acetate (50 mL), quenched with an aqueous 1 M solution of hydrochloric acid (5 mL) and washed with a 10% aqueous solution of lithium chloride (5 × 100 mL), brine (50 mL), dried (MgSO_4_), filtered, and concentrated *in vacuo*. Purification by column chromatography, eluting with 0–2.5% ethanol:chloroform, yielded the title compound (0.29 g, 86%) as a pale-yellow solid. *R*_*f*_ 0.68 (ethyl acetate); mp 140–142°C (from dichloromethane/hexane); ${\bar{\nu }}_{\max}$ (thin film)/cm^−1^ 3192, 2976, 1729, 1687, 1492, 1460, 1367, 1326, 1159, 1137; ^1^H NMR at 363 K (500 MHz, D_6_-DMSO) δ_H_ 11.05 (1H, s), 8.00 (1H, d, *J* 8.0), 7.50 (2H, d, *J* 8.1), 7.48 (1H, d, *J* 15.8), 7.42 (1H, d, *J* 7.5), 7.25 (2H, d, *J* 8.1), 7.25 (1H, s), 7.21 (1H, ddd, *J* 8.0, 7.3, 1.4), 7.16 (1H, ddd, *J* 7.5, 7.3, 1.2), 6.44 (1H, d, *J* 15.8), 5.02 (2H, s), 4.41 (2H, s), 4.08 (3H, s), 3.34 (2H, t, *J* 7.3), 2.84 (2H, t, *J* 7.3), 2.46 (3H, s), 1.64 (9H, s), 1.35 (9H, s); ^13^C NMR at 363 K (126 MHz, (D_6_-DMSO) δ_C_ 163.6,^∗^ 154.4, 149.5, 145.9,^∗^ 140.0, 139.4, 134.9, 133.1, 132.9, 132.4, 129.1, 129.0, 127.4, 127.2, 122.7, 121.8, 117.6, 117.1, 114.8, 114.4, 83.1, 78.5, 65.6, 49.6, 46.1, 33.6, 27.5, 27.4, 22.1, 12.7; HRMS *m/z* (ESI^+^) Found: 711.31117, C_36_H_44_N_6_O_8_Na requires [M+Na]^+^ 711.31128; LRMS (ESI^−^) 678 ([M−H]^−^, 100%), 409 (9%), 281 (9%), 255 (13%); HPLC Method B, Retention time - 12.6 min, 96%. ^∗^Signals at 163.6 and 145.9 observed by ^1^H-^13^C HMBC correlation see supplemental information for details.

##### (*E*)-2-(2-Methyl-1*H*-indol-3-yl)-*N*-(4-(3-(((1-methyl-2-nitro-1*H*-imidazol-5-yl)methoxy)amino)-3-oxoprop-1-en-1-yl)benzyl)ethan-1-aminium 2,2,2-trifluoroacetate (NI-Pano, CH-03, 1)

Trifluoroacetic acid (13.9 mL mL, 20% *v/v*) was added dropwise to a rapidly stirred solution of (*E*)-*tert*-butyl-3-(2-((*tert*-butoxycarbonyl)(4-(3-(((1-methyl-2-nitro-1*H*-imidazol-5-yl)methoxy)amino)-3-oxoprop-1-en-1-yl)benzyl)amino)ethyl)-2-methyl-1*H*-indole-1-carboxylate (410 mg, 0.696 mmol, 1.0 eq) and triisopropylsilane (30.6 μL, 0.139 mmol, 0.2 eq) in dichloromethane (70mL). The reaction mixture was stirred for 55 mins then diluted with toluene (1 mL) and dried by azeotroping with toluene (3 × 1 mL) *in vacuo*. Purification using column chromatography, eluting with 0–20% (70% isopropanol in water solution):ethyl acetate, yielded the title compound (**1**) (303 mg, 61%) as a pale yellow solid. Where necessary the compound was further purified by semi-preparative HPLC (see general experimental, retention time - 11.4 min). *Rf* 0.32 (10% ethanol: chloroform); mp 108–110°C (from methanol); ${\bar{\nu }}_{\max}$ (thin film)/cm^−1^ 3293, 2925, 2853, 1673, 1624, 1539, 1491, 1461, 1342, 1189, 1044; ^1^H NMR (500 MHz, CD_3_OD) δ_H_ 7.59 (2H, d, *J* 7.9), 7.56 (1H, d, *J* 15.9), 7.47 (2H, d, *J* 7.9), 7.39 (1H, d, *J* 7.6), 7.24 (1H, d, *J* 7.7), 7.19 (1H, s), 7.02 (1H, dd, *J* 7.7, 7.5), 6.95 (1H, dd, *J* 7.6, 7.5), 6.47 (1H, d, *J* 15.9), 5.03 (2H, s), 4.21 (2H, s), 4.13 (3H, s), 3.20 (2H, t, *J* 7.8), 3.09 (2H, t, *J* 7.8), 2.37 (3H, s); ^13^C NMR (126 MHz, CD_3_OD) δ_C_ 166.1, 163.1 (q, ^2^*J*_CF_ 34.2), 147.7, 141.7, 137.2, 137.0, 134.7, 134.0 (2×C),^∗^ 131.5, 130.4, 129.5, 129.2, 121.8, 119.9, 119.1, 118.2 (q, ^1^*J*_CF_ 294.2), 118.0, 111.6, 105.8, 67.4, 51.9, 48.9,^∗^ 35.1, 22.3, 11.3; HRMS *m/z* (ESI^+^) Found: 489.22418 C_26_H_29_N_6_O_4_ requires [M+H]^+^ 489.22448; LRMS (ESI^+^) 489 ([M+H]^+^, 100%), 349 (9%); HPLC Method B, Retention time - 7.3 min, 98–99%, mean purity 98%. HPLC Methods D and E were used where necessary. All batches used for biological testing were >95% purity. All batches used for animal studies were >99.9% purity. ^∗^The signals at 48.9 and pair at 134.0 were observed by ^1^H-^13^C HMBC correlation, see supplemental information for details.

##### (*E*)-*N*-(4-(2-Carboxyvinyl)benzyl)-2-(2-methyl-1*H*-indol-3-yl)ethan-1-aminium 2,2,2-trifluoroacetate (Pano acid, S18)

Trifluoroacetic acid (0.14 mL, 20% *v/v*) was added dropwise to a rapidly stirred solution of **5** (15 mg, 0.028 mmol, 1.0 eq) and triisopropylsilane (1.2 μL, 5.6 μmol, 0.2 eq) in dichloromethane (0.7 mL). The reaction mixture was stirred for 65 mins then diluted with toluene (1 mL) and dried by azeotroping with toluene (3 × 1 mL) *in vacuo*. Purification using column chromatography, eluting with 1:2:6 H_2_O:isopropyl alcohol:ethyl acetate, then 100% ethanol, yielded the title compound (**S18**) (7 mg, 78%) as a colorless solid. R_*f*_ 0.37 (1:2:6 H_2_O:isopropyl alcohol:ethyl acetate); mp 95–97°C (from methanol); ${\bar{\nu }}_{\max}$ (thin film)/cm^−1^ 3398, 3032, 2829, 1672, 1462, 1426, 1198, 1139; ^1^H NMR (500 MHz, CD_3_OD) δ_H_ 7.66 (2H, d, *J* 8.3), 7.64 (1H, d, *J* 16.0), 7.50 (2H, d, *J* 8.3), 7.41 (1H, ddd, *J* 8.0, 1.1, 1.0), 7.26 (1H, ddd, *J* 8.1, 1.1, 1.0), 7.03 (1H, ddd, *J* 8.1, 7.1, 1.1), 6.97 (1H, ddd, *J* 8.0, 7.1, 1.1), 6.54 (1H, d, *J* 16.0), 4.24 (2H, s), 3.26–3.19 (2H, m), 3.15–3.07 (2H, m), 2.39 (3H, s); ^13^C NMR (126 MHz, CD_3_OD) δ_C_ 170.8, 163.1 (q, ^2^*J*_CF_ 35.0), 144.1, 137.3, 137.2, 134.3, 134.0, 131.5, 129.7, 129.2, 122.0, 121.8, 119.9, 118.3 (q, ^1^*J*_CF_ 293.2) 117.9, 111.6, 105.6, 51.8, 48.8^∗^, 22.2, 11.3; HRMS *m/z* (ESI^+^) Found: 335.17539 C_21_H_23_N_2_O_2_ requires [M+H]^+^ 335.17540; LRMS (ESI^−^) 369 ([M+Cl]^−^, 100%), 333 ([M−H]^−^, 36%); HPLC Method B, Retention time – 6.9 min, 97%. ^∗^Overlapped by solvent residual signal, see supplemental information for expansion.

##### 4-((1-Methyl-2-nitro-1*H*-imidazol-5-yl)methoxy)benzaldehyde

5-(Chloromethyl)-1-methyl-2-nitro-1*H*-imidazole (0.085 g, 0.485 mmol, 1.00 eq) was added to a stirring solution of 4-hydroxybenzaldehyde (0.065 g, 0.534 mmol, 1.10 eq) and potassium carbonate (0.073 g, 0.534 mmol, 1.10 eq) in dry DMF (2 mL). The solution was stirred at 60°C for 2 h then cooled to rt. The reaction mixture was diluted with EtOAc (50 mL) and washed with brine (5 × 30 mL), then dried (MgSO_4_), filtered, and concentrated *in vacuo*. The residue was dissolved in EtOAc (10 mL), adsorbed onto Celite© and purified using column chromatography (elution with 50% EtOAc/petroleum ether) to yield the title compound (0.073 g, 57%) as a pale-yellow solid; R_*f*_ 0.16 (1:1 EtOAc/petroleum ether); mp 159-161°C (from EtOAc) [lit. (Jin et al., 2017) 181-183°C]; ${\bar{\nu }}_{\max}$ (thin film)/cm^−1^ 2026, 1862, 1527; ^1^H NMR (500 MHz, CDCl_3_) δ_H_ 9.96 (1H, s), 7.93 (2H, d, *J* 4.6), 7.30 (1H, s), 7.12 (2H, d, *J* 1.9), 5.18 (2H, s), 4.11 (3H, s); ^13^C NMR (126 MHz, CDCl_3_) δ_C_ 190.7, 162.3, 132.3, 131.7, 131.3, 129.4, 115.1, 59.8, 34.7; HRMS *m/z* (ESI^+^) Found (M+H)^+^ 262.0824 C_12_H_11_N_3_O_2_ requires (M+H)^+^ 262.0822; LRMS *m/z* (ESI^+^) 284.0 (M+Na)^+^, 100 %]; HPLC (Dionex Acclaim® 120 C18 column [5 μm, 12 Å, 150 mm × 4.6 mm]; 95:5 H_2_O: MeCN → 5:95 H_2_O: MeCN: H_2_O with 0.1 % TFA modifier 10 min; 5 min hold; 1.5 mL/min] Retention time - 7.9 min, 100 %. The spectroscopic data are consistent with literature (Jin et al., 2017).

##### (*E*)-2-(2-(4-((1-Methyl-2-nitro-1*H*-imidazol-5-yl)methoxy)styryl)-4*H*-chromen-4-ylidene)malononitrile

Piperidine (0.089 mL, 0.872 mmol, 4.00 eq) was added to a stirring solution of 4-((1-methyl-2-nitro-1*H*-imidazol-5-yl)methoxy)benzaldehyde (0.060 g, 0.229 mmol, 1.05 eq) and 2-(2-methyl-4*H*-chromen-4-ylidene)malononitrile (0.045 g, 0.218 mmol, 1.00 eq) in dry EtOH (5 mL). The reaction mixture was stirred under reflux for 2 h then cooled to rt. The resulting precipitate was filtered and dried *in vacuo* to give the title compound (0.078 g, 75%) as a brown solid; R_*f*_ 0.28 (dichloromethane); mp 205-207°C (from EtOH) [lit. (Jin et al., 2017) >300°C]; ${\bar{\nu }}_{\max}$ (thin film)/cm^−1^ 2214, 1598; ^1^H NMR (500 MHz, CDCl_3_) δ_H_ 8.95 (1H, d, *J* 8.4), 7.79 (1H, d, *J* 6.7), 7.67–7.58 (4H, m), 7.50 (1H, d, *J* 8.3), 7.29 (1H, s), 7.07 (2H, d, *J* 8.7), 6.89 (1H, s), 6.76 (1H, d, *J* 16.0), 5.16 (2H, s), 4.12 (3H, s); ^13^C NMR (126 MHz, CDCl_3_) δ_C_ 159.3, 157.7, 153.0, 152.5, 138.0, 134.8, 132.0, 129.9, 129.4, 129.1, 126.2, 126.0, 118.7, 118.0, 117.6, 116.9, 106.8, 62.8, 59.8, 34.7; HRMS *m/z* Found (M-H)^−^ 450.1201, C_25_H_17_N_5_O_3_ requires (M-H)^−^ 450.1208; HPLC (Dionex Acclaim® 120 C18 column [5 μm, 12 Å, 150 mm × 4.6 mm]; 95:5 H_2_O: MeCN → 5:95 H_2_O: MeCN: H_2_O with 0.1 % TFA modifier 10 min; 5 min hold; 1.5 mL/min] Retention time - 11.9 min, 100 %. The spectroscopic data are consistent with literature (Jin et al., 2017).

### Quantification and statistical analysis

Statistical analysis was performed using GraphPad Prism 8 software (GraphPad Software Inc.). For the colony survival assay and spheroid growth analysis the 2-way ANOVA test was used. The log-rank test was used to compare mouse survival curves. P values of less than 0.05 were considered as significant (∗p < 0.05; ∗∗p < 0.01; ∗∗∗p < 0.001; ∗∗∗∗p < 0.0001). Unless otherwise indicated, all data represent the mean ± standard deviation from three independent experiments.

## Supplemental information

Document S1. Figures S1–S6, Scheme S1–S3, and Data S1 and S2

Document S2. Article plus supplemental information

## Footnotes

Supplemental information can be found online at https://doi.org/10.1016/j.chembiol.2021.04.004.

## References

[bib1] Arts J., King P., Marien A., Floren W., Belien A., Janssen L., Pilatte I., Roux B., Decrane L., Gilissen R. (2009). JNJ-26481585, a novel “second-generation” oral histone deacetylase inhibitor, shows broad-spectrum preclinical antitumoral activity. Clin. Cancer Res..

[bib2] Baran N., Konopleva M. (2017). Molecular pathways: hypoxia-activated prodrugs in cancer therapy. Clin. Cancer Res..

[bib3] Bergman J.A., Woan K., Perez-Villarroel P., Villagra A., Sotomayor E.M., Kozikowski A.P. (2012). Selective histone deacetylase 6 inhibitors bearing substituted urea linkers inhibit melanoma cell growth. J. Med. Chem..

[bib4] Bradner J.E., West N., Grachan M.L., Greenberg E.F., Haggarty S.J., Warnow T., Mazitschek R. (2010). Chemical phylogenetics of histone deacetylases. Nat. Chem. Biol..

[bib5] Brady O.L., Klein L. (1927). CXXVIII.—the isomerism of the oximes. Part XXIX. Isomeric p-nitrobenzyl and methyl ethers of some aldoximes. J. Chem. Soc..

[bib6] Bristow R.G., Hill R.P. (2008). Hypoxia and metabolism. Hypoxia, DNA repair and genetic instability. Nat. Rev. Cancer.

[bib7] Butler K.V., Kalin J., Brochier C., Vistoli G., Langley B., Kozikowski A.P. (2010). Rational design and simple chemistry yield a superior, neuroprotective HDAC6 inhibitor, tubastatin A. J. Am. Chem. Soc..

[bib8] Calder E.D.D., Skwarska A., Sneddon D., Folkes L.K., Mistry I.N., Conway S.J., Hammond E.M. (2020). Hypoxia-activated pro-drugs of the KDAC inhibitor vorinostat (SAHA). Tetrahedron.

[bib9] Cao L.L., Yue Z., Liu L., Pei L., Yin Y., Qin L., Zhao J., Liu H., Wang H., Jia M. (2017). The expression of histone deacetylase HDAC1 correlates with the progression and prognosis of gastrointestinal malignancy. Oncotarget.

[bib10] Cazares-Korner C., Pires I.M., Swallow I.D., Grayer S.C., O’Connor L.J., Olcina M.M., Christlieb M., Conway S.J., Hammond E.M. (2013). CH-01 is a hypoxia-activated prodrug that sensitizes cells to hypoxia/reoxygenation through inhibition of Chk1 and Aurora A. ACS Chem. Biol..

[bib11] Chen C., Pore N., Behrooz A., Ismail-Beigi F., Maity A. (2001). Regulation of glut1 mRNA by hypoxia-inducible factor-1. Interaction between H-ras and hypoxia. J. Biol. Chem..

[bib12] Chen S., Zhang P., Chen H., Zhang P., Yu Y., Gan Z. (2018). An improved and efficient synthesis of panobinostat. J. Chem. Res..

[bib13] Chouaib S., Noman M.Z., Kosmatopoulos K., Curran M.A. (2017). Hypoxic stress: obstacles and opportunities for innovative immunotherapy of cancer. Oncogene.

[bib14] Clive S., Woo M.M., Nydam T., Kelly L., Squier M., Kagan M. (2012). Characterizing the disposition, metabolism, and excretion of an orally active pan-deacetylase inhibitor, panobinostat, via trace radiolabeled 14C material in advanced cancer patients. Cancer Chemother. Pharmacol..

[bib15] Collins S.L., Saha J., Bouchez L.C., Hammond E.M., Conway S.J. (2018). Hypoxia-activated, small-molecule-induced gene expression. ACS Chem. Biol..

[bib16] Conway, S., O’Connor, L., Hammond, E., 2016. Fluorogenic protecting group. US20160264558A1.

[bib17] Darvin P., Toor S.M., Sasidharan Nair V., Elkord E. (2018). Immune checkpoint inhibitors: recent progress and potential biomarkers. Exp. Mol. Med..

[bib18] Feng W., Zhang B., Cai D., Zou X. (2014). Therapeutic potential of histone deacetylase inhibitors in pancreatic cancer. Cancer Lett..

[bib19] Fraga M.F., Ballestar E., Villar-Garea A., Boix-Chornet M., Espada J., Schotta G., Bonaldi T., Haydon C., Ropero S., Petrie K. (2005). Loss of acetylation at Lys16 and trimethylation at Lys20 of histone H4 is a common hallmark of human cancer. Nat. Genet..

[bib20] Fritz J.M., Lenardo M.J. (2019). Development of immune checkpoint therapy for cancer. J. Exp. Med..

[bib21] Fulmer G.R., Miller A.J.M., Sherden N.H., Gottlieb H.E., Nudelman A., Stoltz B.M., Bercaw J.E., Goldberg K.I. (2010). NMR chemical shifts of trace impurities: common laboratory solvents, organics, and gases in deuterated solvents relevant to the organometallic chemist. Organometallics.

[bib22] Gao S., Zang J., Gao Q., Liang X., Ding Q., Li X., Xu W., Chou C.J., Zhang Y. (2017). Design, synthesis and anti-tumor activity study of novel histone deacetylase inhibitors containing isatin-based caps and o-phenylenediamine-based zinc binding groups. Bioorg. Med. Chem..

[bib23] Gatti L., Zunino F. (2005). Overview of tumor cell chemoresistance mechanisms. Methods Mol. Med..

[bib24] Gottesman M.M. (2002). Mechanisms of cancer drug resistance. Annu. Rev. Med..

[bib25] Graham K., Unger E. (2018). Overcoming tumor hypoxia as a barrier to radiotherapy, chemotherapy and immunotherapy in cancer treatment. Int. J. Nanomedicine.

[bib26] Grandberg I.I. (1974). Indolylalkylamines from arylhydrazines and γ- or δ-halocarbonyl compounds (review). Chem. Heterocycl Compd..

[bib27] Grandberg I.I., Zuyanova T.I. (1971). Indoles. Chem. Heterocycl Compd..

[bib28] Hai Y., Christianson D.W. (2016). Histone deacetylase 6 structure and molecular basis of catalysis and inhibition. Nat. Chem. Biol..

[bib29] Hajipour A.R., Karami K., Tavakoli G. (2010). Heck coupling reaction using monomeric ortho-palladated complex of 4-methoxy- benzoylmethylenetriphenylphosphorane under microwave irradiation. Appl. Organomet. Chem..

[bib30] Hammond E.M., Asselin M.C., Forster D., O’Connor J.P., Senra J.M., Williams K.J. (2014). The meaning, measurement and modification of hypoxia in the laboratory and the clinic. Clin. Oncol. R Coll. Radiol..

[bib31] Harris A.L. (2002). Hypoxia--a key regulatory factor in tumour growth. Nat. Rev. Cancer.

[bib32] Hennika T., Hu G., Olaciregui N.G., Barton K.L., Ehteda A., Chitranjan A., Chang C., Gifford A.J., Tsoli M., Ziegler D.S. (2017). Pre-clinical study of panobinostat in xenograft and genetically engineered murine diffuse intrinsic pontine glioma models. PLoS One.

[bib33] Ho Y.-H., Wang K.-J., Hung P.-Y., Cheng Y.-S., Liu J.-R., Fung S.-T., Liang P.-H., Chern J.-W., Yu C.-W. (2018). A highly HDAC6-selective inhibitor acts as a fluorescent probe. Org. Biomol. Chem..

[bib34] Hu J., Handisides D.R., Van Valckenborgh E., De Raeve H., Menu E., Vande Broek I., Liu Q., Sun J.D., Van Camp B., Hart C.P. (2010). Targeting the multiple myeloma hypoxic niche with TH-302, a hypoxia-activated prodrug. Blood.

[bib35] Huang F.-I., Wu Y.-W., Sung T.-Y., Liou J.-P., Lin M.-H., Pan S.-L., Yang C.-R. (2019). MPT0G413, A novel HDAC6-selective inhibitor, and bortezomib synergistically exert anti-tumor activity in multiple myeloma cells. Front. Oncol..

[bib36] Hunter F.W., Wouters B.G., Wilson W.R. (2016). Hypoxia-activated prodrugs: paths forward in the era of personalised medicine. Br. J. Cancer.

[bib37] Jayaprakash P., Ai M., Liu A., Budhani P., Bartkowiak T., Sheng J., Ager C., Nicholas C., Jaiswal A.R., Sun Y. (2018). Targeted hypoxia reduction restores T cell infiltration and sensitizes prostate cancer to immunotherapy. J. Clin. Invest..

[bib38] Jin C., Zhang Q., Lu W. (2017). Selective turn-on near-infrared fluorescence probe for hypoxic tumor cell imaging. RSC Adv..

[bib39] Kazanets A., Shorstova T., Hilmi K., Marques M., Witcher M. (2016). Epigenetic silencing of tumor suppressor genes: paradigms, puzzles, and potential. Biochim. Biophys. Acta.

[bib40] Kim M.S., Kwon H.J., Lee Y.M., Baek J.H., Jang J.E., Lee S.W., Moon E.J., Kim H.S., Lee S.K., Chung H.Y. (2001). Histone deacetylases induce angiogenesis by negative regulation of tumor suppressor genes. Nat. Med..

[bib41] Kim S.H., Jeong J.W., Park J.A., Lee J.W., Seo J.H., Jung B.K., Bae M.K., Kim K.W. (2007). Regulation of the HIF-1alpha stability by histone deacetylases. Oncol. Rep..

[bib42] Kong X., Lin Z., Liang D., Fath D., Sang N., Caro J. (2006). Histone deacetylase inhibitors induce VHL and ubiquitin-independent proteasomal degradation of hypoxia-inducible factor 1alpha. Mol. Cell Biol..

[bib43] Lee J.-H., Mahendran A., Yao Y., Ngo L., Venta-Perez G., Choy M.L., Kim N., Ham W.-S., Breslow R., Marks P.A. (2013). Development of a histone deacetylase 6 inhibitor and its biological effects. Proc. Natl. Acad. Sci. U S A.

[bib44] Leszczynska K.B., Dobrynin G., Leslie R.E., Ient J., Boumelha A.J., Senra J.M., Hawkins M.A., Maughan T., Mukherjee S., Hammond E.M. (2016). Preclinical testing of an ATR inhibitor demonstrates improved response to standard therapies for esophageal cancer. Radiother. Oncol..

[bib45] Li Y., Seto E. (2016). HDACs and HDAC inhibitors in cancer development and therapy. Cold Spring Harb Perspect. Med..

[bib46] Meel M.H., Schaper S.A., Kaspers G.J.L., Hulleman E. (2018). Signaling pathways and mesenchymal transition in pediatric high-grade glioma. Cell Mol. Life Sci..

[bib47] Minchinton A.I., Tannock I.F. (2006). Drug penetration in solid tumours. Nat. Rev. Cancer.

[bib48] Mistry I.N., Thomas M., Calder E.D.D., Conway S.J., Hammond E.M. (2017). Clinical advances of hypoxia-activated prodrugs in combination with radiation therapy. Int. J. Radiat. Oncol. Biol. Phys..

[bib49] O’Connor L.J., Cazares-Korner C., Saha J., Evans C.N., Stratford M.R., Hammond E.M., Conway S.J. (2016). Design, synthesis and evaluation of molecularly targeted hypoxia-activated prodrugs. Nat. Protoc..

[bib50] O’Connor L.J., Cazares-Körner C., Saha J., Evans C.N.G., Stratford M.R.L., Hammond E.M., Conway S.J. (2015). Efficient synthesis of 2-nitroimidazole derivatives and the bioreductive clinical candidate Evofosfamide (TH-302). Org. Chem. Front..

[bib51] Pangborn A.B., Giardello M.A., Grubbs R.H., Rosen R.K., Timmers F.J. (1996). Safe and convenient procedure for solvent purification. Organometallics.

[bib52] Parveen I., Naughton D.P., Whish W.J., Threadgill M.D. (1999). 2-nitroimidazol-5-ylmethyl as a potential bioreductively activated prodrug system: reductively triggered release of the PARP inhibitor 5-bromoisoquinolinone. Bioorg. Med. Chem. Lett..

[bib53] Phillips R.M. (2016). Targeting the hypoxic fraction of tumours using hypoxia-activated prodrugs. Cancer Chemother. Pharmacol..

[bib54] Portwood S., Lal D., Hsu Y.C., Vargas R., Johnson M.K., Wetzler M., Hart C.P., Wang E.S. (2013). Activity of the hypoxia-activated prodrug, TH-302, in preclinical human acute myeloid leukemia models. Clin. Cancer Res..

[bib55] Righi M., Topi F., Bartolucci S., Bedini A., Piersanti G., Spadoni G. (2012). Synthesis of tryptamine derivatives via a direct, one-pot reductive alkylation of indoles. J. Org. Chem..

[bib56] Rudakova E.V., Boltneva N.P., Makhaeva G.F. (2011). Comparative analysis of esterase activities of human, mouse, and rat blood. Bull. Exp. Biol. Med..

[bib57] San-Miguel J.F., Richardson P.G., Gunther A., Sezer O., Siegel D., Blade J., LeBlanc R., Sutherland H., Sopala M., Mishra K.K. (2013). Phase Ib study of panobinostat and bortezomib in relapsed or relapsed and refractory multiple myeloma. J. Clin. Oncol..

[bib58] Sapountzis I., Dube H., Lewis R., Gommermann N., Knochel P. (2005). Synthesis of functionalized nitroarylmagnesium halides via an Iodine−Magnesium exchange. J. Org. Chem..

[bib59] Sharma A., Arambula J.F., Koo S., Kumar R., Singh H., Sessler J.L., Kim J.S. (2019). Hypoxia-targeted drug delivery. Chem. Soc. Rev..

[bib60] Skultetyova L., Ustinova K., Kutil Z., Novakova Z., Pavlicek J., Mikesova J., Trapl D., Baranova P., Havlinova B., Hubalek M. (2017). Human histone deacetylase 6 shows strong preference for tubulin dimers over assembled microtubules. Sci. Rep..

[bib61] Slade J., Parker D., Girgis M., Wu R., Joseph S., Repič O. (2007). Optimization and scale-up of the Grandberg synthesis of 2-methyltryptamine. Org. Process. Res. Dev..

[bib62] Spiegelberg L., Houben R., Niemans R., de Ruysscher D., Yaromina A., Theys J., Guise C.P., Smaill J.B., Patterson A.V., Lambin P. (2019). Hypoxia-activated prodrugs and (lack of) clinical progress: the need for hypoxia-based biomarker patient selection in phase III clinical trials. Clin. Transl Radiat. Oncol..

[bib63] Spiegelberg L., van Hoof S.J., Biemans R., Lieuwes N.G., Marcus D., Niemans R., Theys J., Yaromina A., Lambin P., Verhaegen F. (2019). Evofosfamide sensitizes esophageal carcinomas to radiation without increasing normal tissue toxicity. Radiother. Oncol..

[bib64] Sudo T., Mimori K., Nishida N., Kogo R., Iwaya T., Tanaka F., Shibata K., Fujita H., Shirouzu K., Mori M. (2011). Histone deacetylase 1 expression in gastric cancer. Oncol. Rep..

[bib65] Sohda T., Taketomi S., Oda T. (1998).

[bib66] Tannock I.F., Lee C.M., Tunggal J.K., Cowan D.S., Egorin M.J. (2002). Limited penetration of anticancer drugs through tumor tissue: a potential cause of resistance of solid tumors to chemotherapy. Clin. Cancer Res..

[bib67] Tredan O., Galmarini C.M., Patel K., Tannock I.F. (2007). Drug resistance and the solid tumor microenvironment. J. Natl. Cancer Inst..

[bib68] Wagner F.F., Olson D.E., Gale J.P., Kaya T., Weïwer M., Aidoud N., Thomas M., Davoine E.L., Lemercier B.C., Zhang Y.-L. (2013). Potent and selective inhibition of histone deacetylase 6 (HDAC6) does not require a surface-binding motif. J. Med. Chem..

[bib69] Wang M.-Z., Xu H., Liu T.-W., Feng Q., Yu S.-J., Wang S.-H., Li Z.-M. (2011). Design, synthesis and antifungal activities of novel pyrrole alkaloid analogs. Eur. J. Med. Chem..

[bib70] Wang S.-H., Wang S.-F., Xuan W., Zeng Z.-H., Jin J.-Y., Ma J., Tian G.R. (2008). Nitro as a novel zinc-binding group in the inhibition of carboxypeptidase A. Bioorg. Med. Chem..

[bib71] Weinberg D.N., Allis C.D., Lu C. (2017). Oncogenic mechanisms of histone H3 mutations. Cold Spring Harb Perspect. Med..

[bib72] Wiles C., Watts P., Haswell S.J. (2006). Clean and selective oxidation of aromatic alcohols using silica-supported Jones’ reagent in a pressure-driven flow reactor. Tetrahedron Lett..

[bib73] Winn B.A., Shi Z., Carlson G.J., Wang Y., Nguyen B.L., Kelly E.M., Ross R.D., Hamel E., Chaplin D.J., Trawick M.L. (2017). Bioreductively activatable prodrug conjugates of phenstatin designed to target tumor hypoxia. Bioorg. Med. Chem. Lett..

[bib74] Witter D.J., Harrington P., Wilson K.J., Chenard M., Fleming J.C., Haines B., Kral A.M., Secrist J.P., Miller T.A. (2008). Optimization of biaryl selective HDAC1&2 inhibitors (SHI-1:2). Bioorg. Med. Chem. Lett..

[bib75] Xu, Y., 2017. Synthesis method of panobinostat. CN106674079A.

[bib76] Zeng Y., Ma J., Zhan Y., Xu X., Zeng Q., Liang J., Chen X. (2018). Hypoxia-activated prodrugs and redox-responsive nanocarriers. Int. J. Nanomedicine.

[bib77] Zhang J., Zhong Q. (2014). Histone deacetylase inhibitors and cell death. Cell Mol. Life Sci..

[bib78] Zhang Z., Yamashita H., Toyama T., Sugiura H., Ando Y., Mita K., Hamaguchi M., Hara Y., Kobayashi S., Iwase H. (2005). Quantitation of HDAC1 mRNA expression in invasive carcinoma of the breast∗. Breast Cancer Res. Treat..

