# Supplementary material for: Development and pre-clinical testing of a novel hypoxia-activated KDAC inhibitor
Source: Cell Chem Biol. 2021 Sep 16;28(9):1258–1270.e13. doi: 10.1016/j.chembiol.2021.04.004 (PMC8460716; doi:10.1016/j.chembiol.2021.04.004)

Cell Chemical Biology, Volume *28*

Supplemental information

Development and pre-clinical testing

# of a novel hypoxia-activated KDAC inhibitor

Anna Skwarska, Ewen D.D. Calder, Deborah Sneddon, Hannah Bolland, Maria L. Odyniec, Ishna N. Mistry, Jennifer Martin, Lisa K. Folkes, Stuart J. Conway, and Ester M. Hammond

Development and pre-clinical testing of a novel hypoxia-activated KDAC inhibitor

Anna Skwarska[$2], Ewen D. D. Calder[$1], Deborah Sneddon[1], Hannah Bolland[2], Maria L. Odyniec[1], Ishna N. Mistry[2], Jennifer Martin[2], Lisa K. Folkes[2], Stuart J. Conway*[1] and Ester M. Hammond*[2]

[1]Department of Chemistry, Chemistry Research Laboratory, University of Oxford, Mansfield Road, Oxford, OX1 3TA, UK.
[2]Oxford Institute for Radiation Oncology, Department of Oncology, University of Oxford, Old Road Campus Research Building, Oxford, OX3 7DQ, UK.

[$]equal contributions

*corresponding authors

Contents

Supplementary Figures S2
Supplementary Schemes S8
Data S1 NMR Spectra S10
Data S2 HPLC Traces S70

SUPPLEMENTARY FIGURES

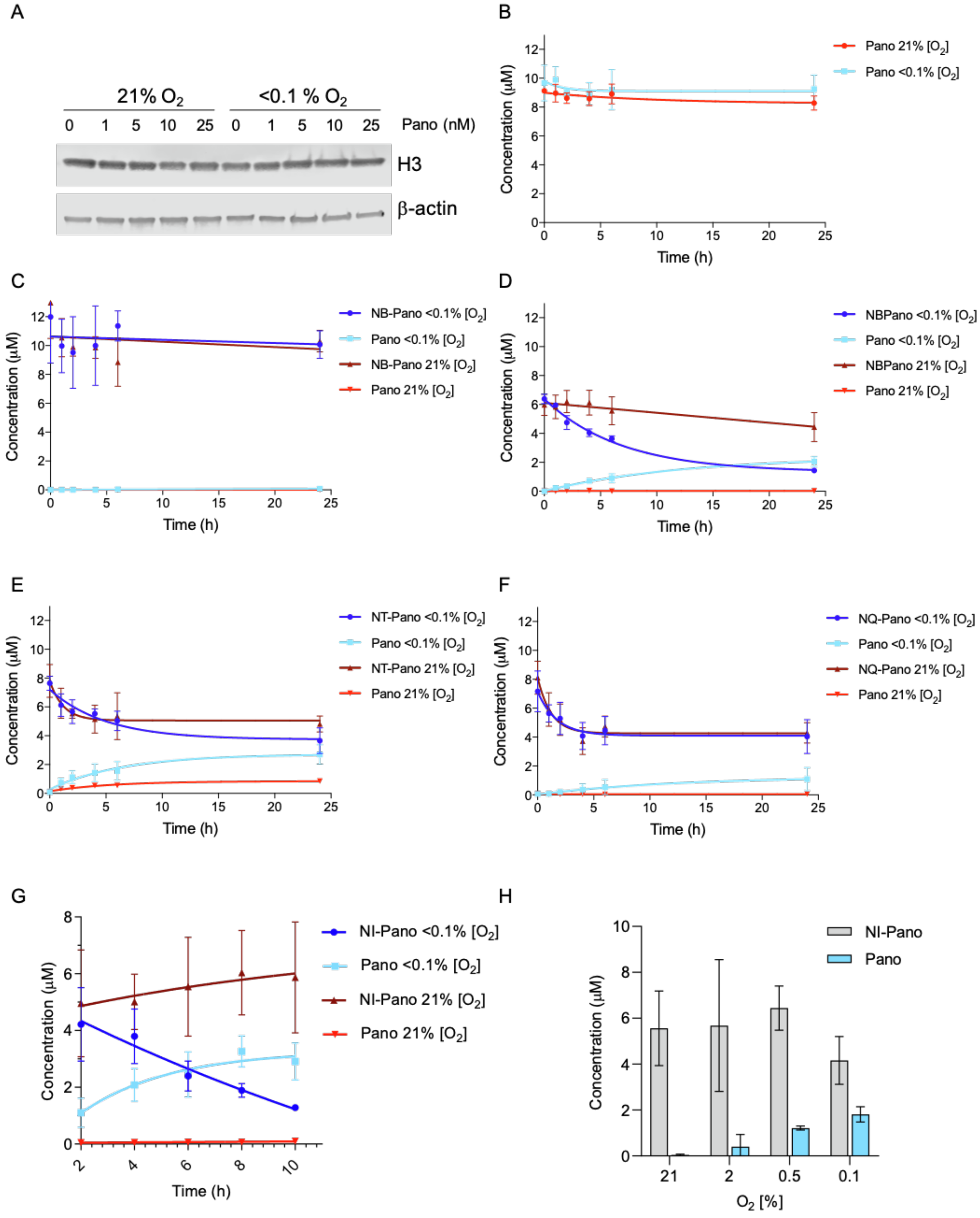

Figure S1. Oxygen dependent reduction and fragmentation of HAPs of panobinostat, related to figure 2. **A.** H3 and β-actin blots corresponding to the H3K18Ac shown in figure 2A. **B**. panobinostat (10 µM), **C**. NB-Pano (10 µM), **D**. NBPano at 92 pmol CYP004, **E.** NT-Pano (10 µM) and **F.** NQ-Pano (10 µM) were incubated with 9.2 pmol/mL of bactosomal NADPH-CYP reductase (CYP004) in normoxic (21% O_2) or hypoxic (<0.1% O_2) conditions for 0-24 h and analyzed using LCMS. **G.** OE21 cells were treated with NI-Pano (10 µM) in either normoxia or hypoxia (<0.1% O_2) for the indicated times and the reduction of NI-Pano to Pano was determined by LCMS. **H.** OE21 cells were treated with NI-Pano (10 µM) for 6 h at different oxygen concentrations and analyzed by LCMS. Data are mean ± SD, *n*=3, except **B** where *n*=2.

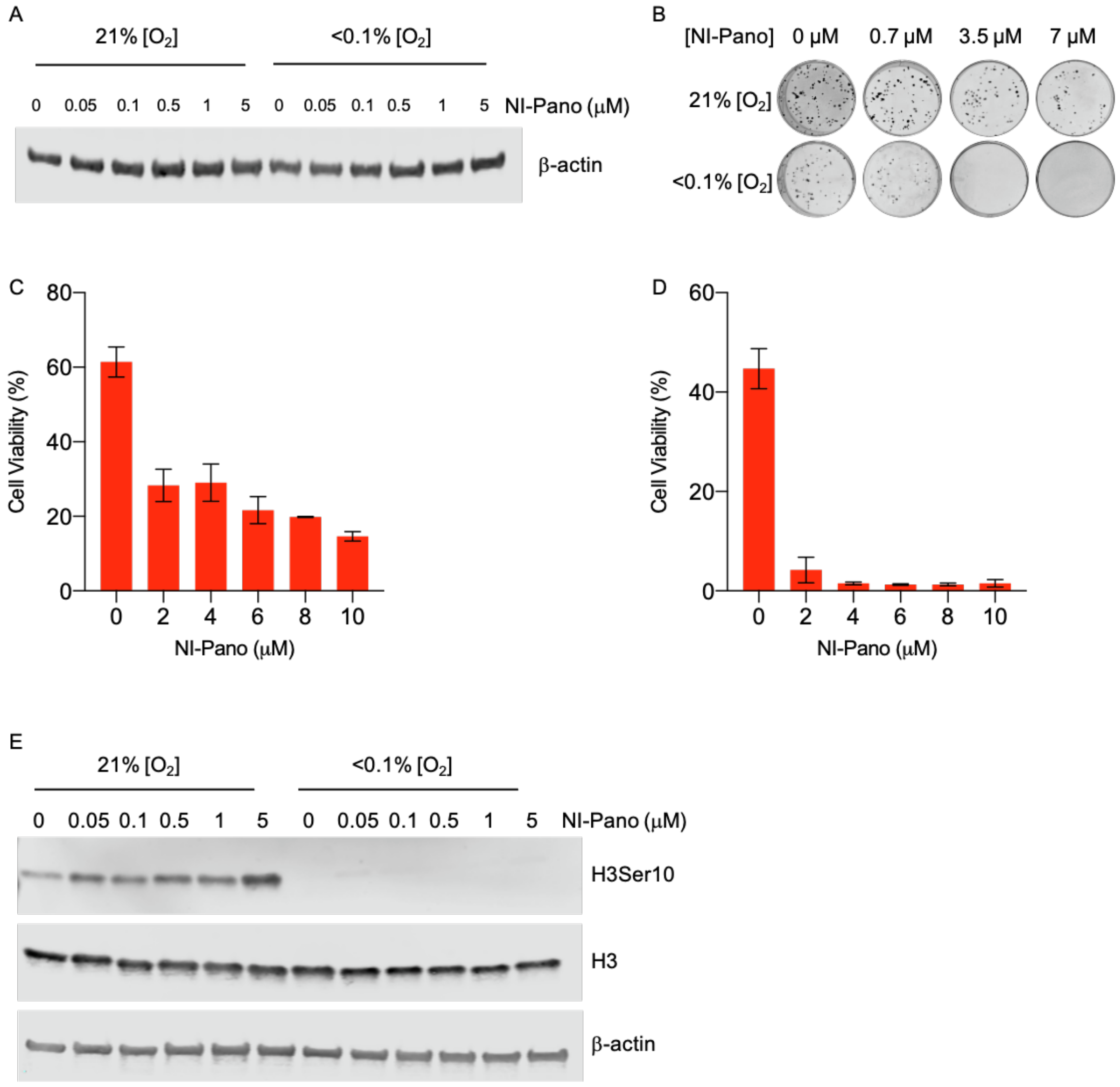

Figure S2. NI-pano decreases cancer cell survival in hypoxia, related to figure 3. A. Additional loading control for western blots shown in Fig. 2A. **B.** HCT116 cells were treated with NI-pano (0-7 μM) for 24 h at the oxygen concentration shown. NI-Pano was removed and cells allowed to form colonies in normoxic conditions. Representative images from figure 3D are shown. **C, D.** OE21 cells were treated with 0, 2, 4, 6, 8 or 10 μM of NI-Pano for 24 or 48 hours and exposed to <0.1% O_2. Percentage cell viability was assessed using MTT assay. **E.** OE21 cells were treated with indicated concentrations of NI-Pano and exposed to normoxia or hypoxia (<0.1% O_2) for 24 hours. Samples were probed for the phosphorylation of H3Ser10, total H3 and β-actin.

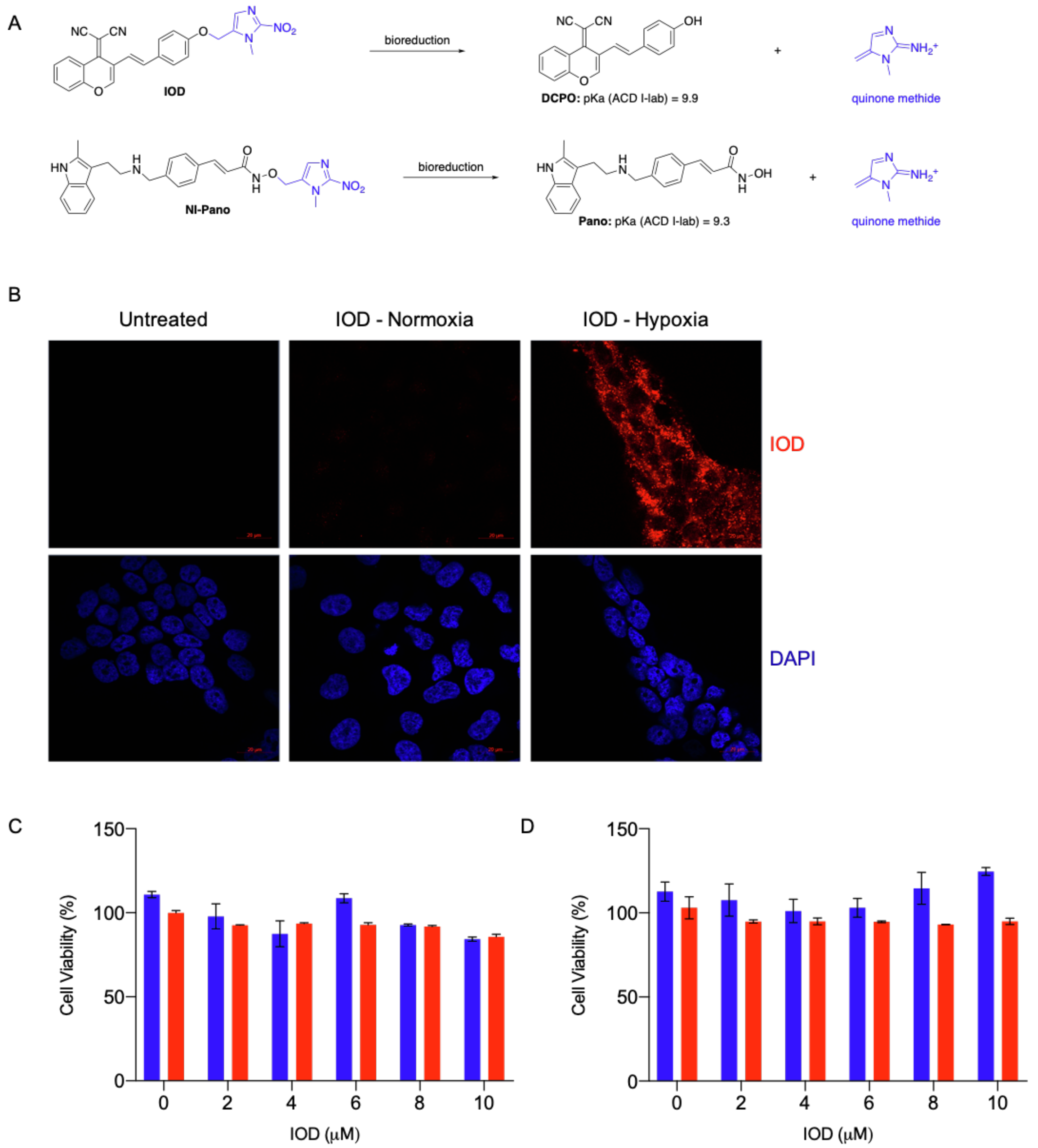

Figure S3. IOD treatment is not toxic, related to figure 2. A. A scheme showing the bioreductions of IOD and NI-Pano, which both result in the production of the same quinone-methide-like by-product. **B.** OE21 cells were treated with 10 μM of IOD and exposed to <0.1% O_2 for 16 hours. DAPI (DNA binding) was used to visualize the nucleus. Cells were fixed and imaged on a Zeiss LSM 780 confocal microscope. Images were taken at 63x magnification. Scale bar represents 20 μm. **C**. OE21 cells were treated with indicated concentrations of IOD for 24 hours and exposed to either 21% O_2 (blue) or <0.1% O_2 (red). Cell viability was measured using an MTT assay. 21% O_2 and <0.1% O_2 data are normalized to the respective 0 μM controls. **D**. OE21 cells were treated with indicated concentrations of IOD for 72 hours and exposed to either 21% O_2 or <0.1% O_2. Cell viability was measured using an MTT assay. 21% O_2 and <0.1% O_2 data are normalized to the respective 0 μM controls.

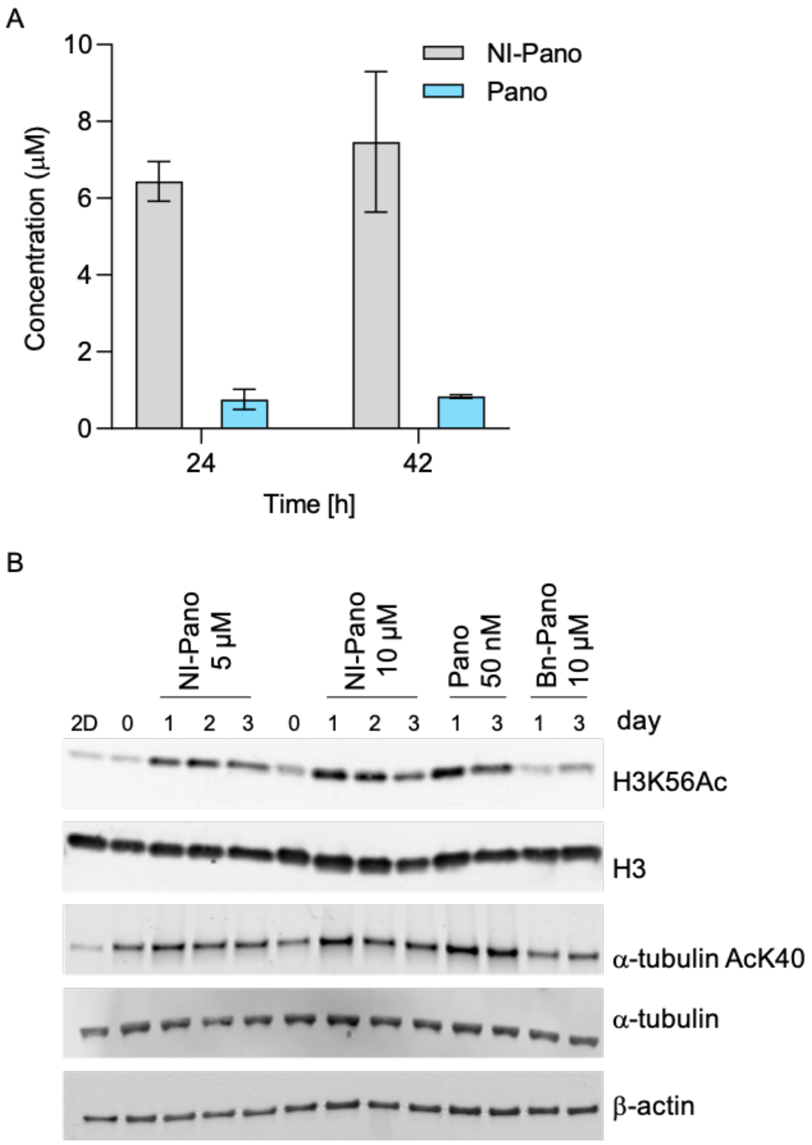

Figure S4. NI-Pano is reduced in cancer cell spheroids, related to figure 4. A. HCT116 cell spheroids were treated with NI-Pano (10 μM) for the times indicated and the reduction of NI-Pano to Pano was determined by HPLC. At least 24 spheroids were analyzed per treatment condition. Data are mean ± SD, *n*=3. **B.** Western blotting was carried out on the spheroid samples described in Fig. 4D using the antibodies indicated.

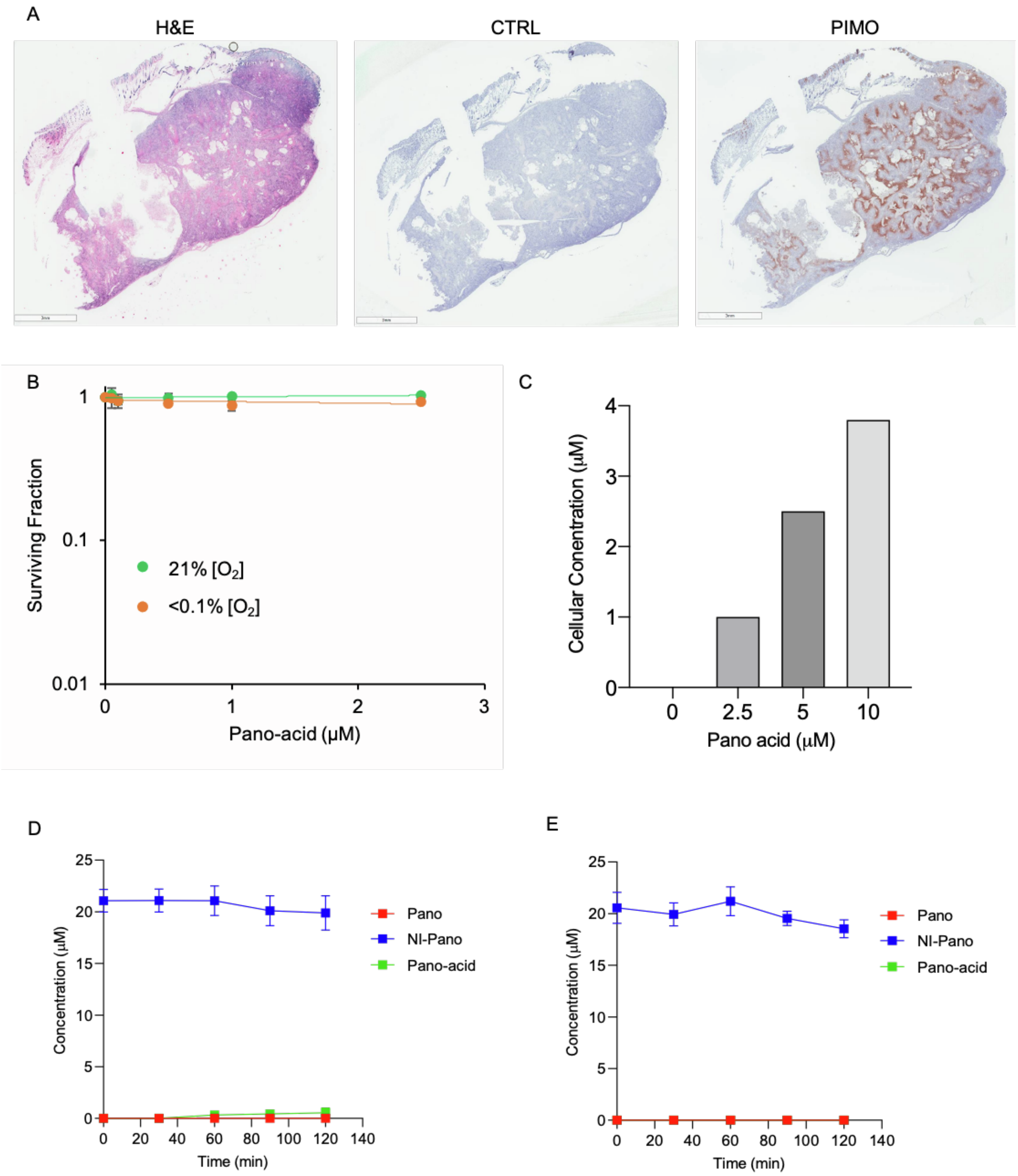

Figure S5. ***In vivo*** **testing of NI-Pano, related to figure 5. A.** OE21 xenograft tumors were stained with anti-pimonidazole antibody (PIMO) to visualize hypoxic regions and counterstained with hematoxylin and eosin (H&E). Anti-mouse secondary antibody alone was used as a negative control (CTRL). **B.** OE21 cells were treated with Pano-acid (0-2.5 µM) for 24 h at the oxygen concentrations shown. The Pano-acid was removed (media change) and cells allowed to form colonies in normoxic conditions. **C.** OE21 cells were treated with indicated doses of Pano-acid for 6 hours under normoxic conditions. Cells were collected and resulting supernatant analyzed by HPLC **D.** Plasma from healthy rats or **E.** human plasma from healthy donors was incubated with NI-Pano (20 µM) for indicated times and analyzed by LCMS. Data are mean ± SD, *n*=3.

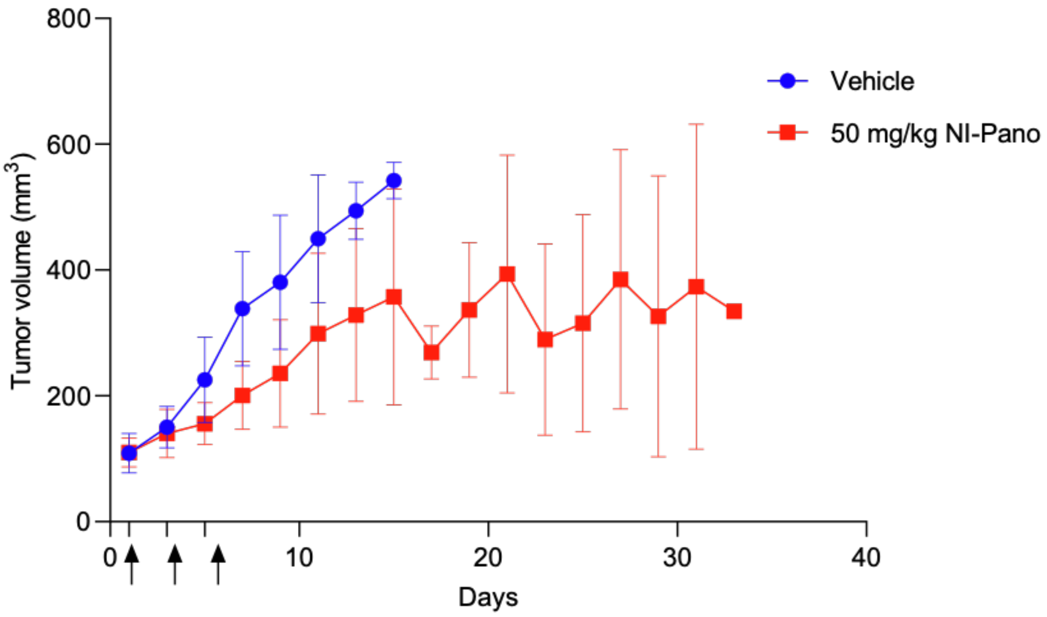

Figure S6. NI-Pano inhibits growth of OE21 xenografts, related to figure 5. Tumor volume over time in CD-1 nude mice administered with three doses of either vehicle (*n*=6) or 50 mg/kg NI-Pano (*n*=7). Arrows indicate days of treatment (days 1, 3 and 5). Results are mean ± SD.

SUPPLEMENTARY SCHEMES

Scheme S1. General Synthesis of Panobinostat (2), related to figure 1

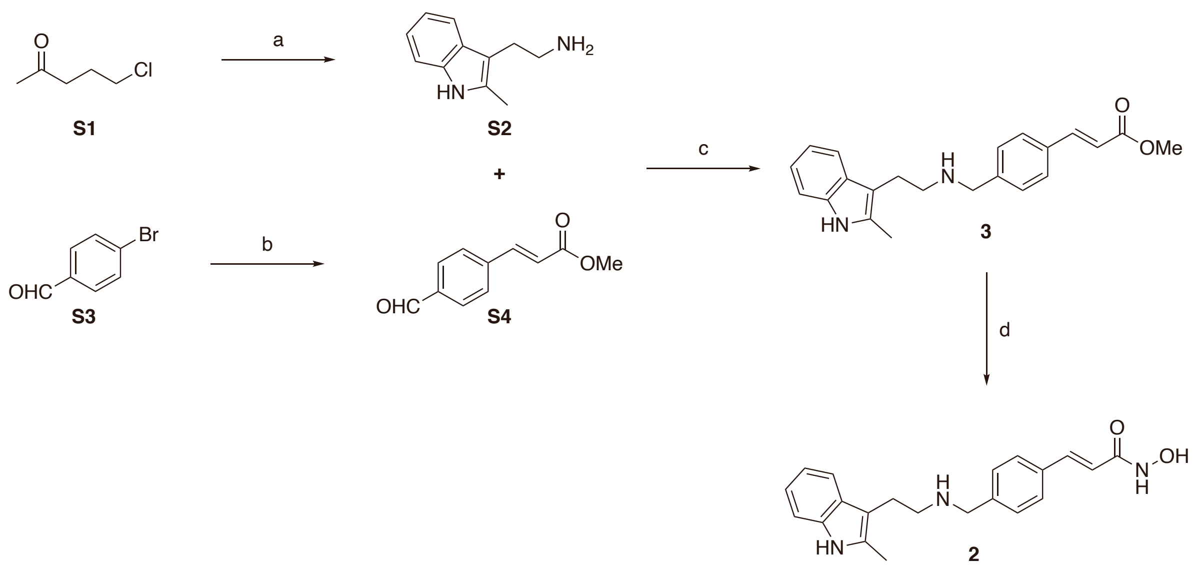

Reagents and conditions: (a) $PhNHNH_2$, EtOH, 80 °C, 18 h, 79-89%, n=4; (b) Methyl acrylate, $Pd(OAc)_2$, KOAc, DMF, 110 C, 24 h, 94-99%, n=5; (c) $ClCH_2CH_2Cl$, AcOH, 3 Å molecular sieves, rt, 1 h then $NaBH(OAc)_3$, rt, 18 h, 52-86%, n=6; (d) KOH, $HONH_2{\cdot}HCl$, MeOH, rt, 20 h, 63%.

Scheme S2: Synthesis the bioreductive group precursors S7, S10, S14, S17, related to scheme 1.

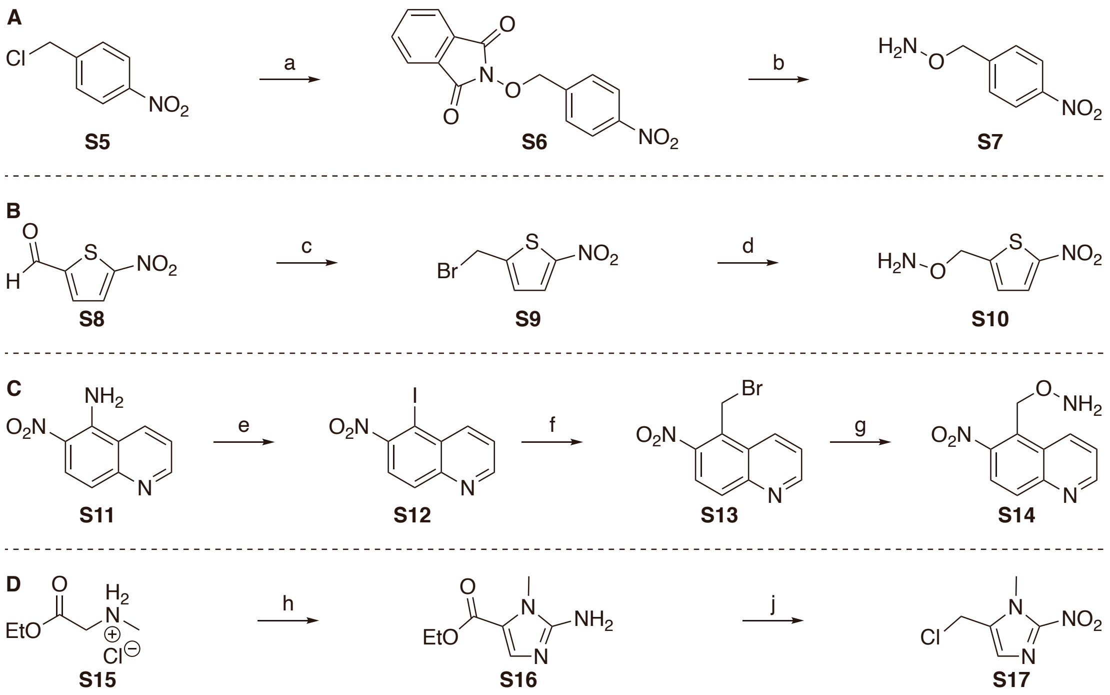

Reagents and conditions: (**A**) (a) *N*-Hydroxyphthalimide, DIPEA, DMF, 0 °C to rt, 2 h, 76–84%, n=2; (b) $N_2H_4 \cdot H_2O$, CH_2Cl_2, rt, 2 h, 72–95%, n=4. (**B**) (c) (i) $NaBH_4$, MeOH, 0 °C to rt, 2 h, 79–99%, n=4; (ii) PBr_3, CH_2Cl_2, 0 °C to rt, 5 h, 24–48%, n=4; (d) (i) *N*-Hydroxyphthalimide, DIPEA, DMF, 0 °C to rt, 2 h, 47–63%, n=2, (ii) $N_2H_4 \cdot H_2O$, CH_2Cl_2, rt 2 h, 46%, n=1. (**C**) (e) CuI, $NaNO_2$, HI (57% aq.), DMSO, 60 °C, 0.5 h, 31–60%, n=2; (f) (i) PhMgCl, THF, −40 °C, 10 min then $(CH_2O)_n$, −40 °C to 40 °C, 18 h, 42–86%, n=4; (ii) HBr (48% aq.), 75 °C, 18 h, 77–99%, n=2; (g) (i) *N*-Hydroxyphthalimide, DIPEA, DMF, 0 °C to rt, 2 h, 88%, n=1; (ii) $N_2H_4 \cdot H_2O$, CH_2Cl_2, rt 2 h, 92%, n=1. (**D**) (h) (i) NaH, EtOCHO, THF, EtOH, 0 °C to rt, 18 h; (ii) HCl (37% aq.), EtOH, rt, 2 h; (iii) $NCNH_2$, EtOH, H_2O, 100 °C, 2 h, 41–79% (over 3 steps), n=5; (j) (i) $NaNO_2$, AcOH, H_2O, 0 °C to rt, 4 h, 57–81%, n=5; (ii) $NaBH_4$, EtOH, THF, 0 °C, 3 h, 70–80%, n=5; (iii) CH_3SO_2Cl, pyridine, 0 °C to rt, 3 h, 54–67%, n=5.

Scheme S3. Synthesis of Pano-acid (S18), related to figure 5

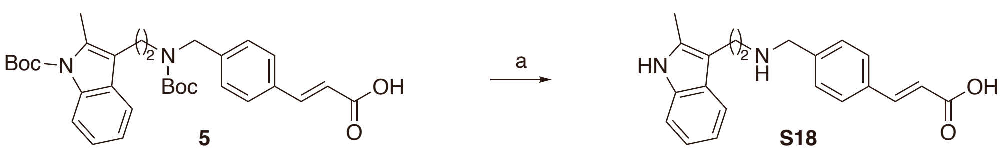

Reagents and conditions: (a) TFA, TIPS-H, CH_2Cl_2, rt, 65 min, 78%.

*Methyl (E)-3-(4-{[2-(2-methyl-1H-indol-3-yl)ethylamino]methyl}phenyl)prop-2-enoate (**3**)*

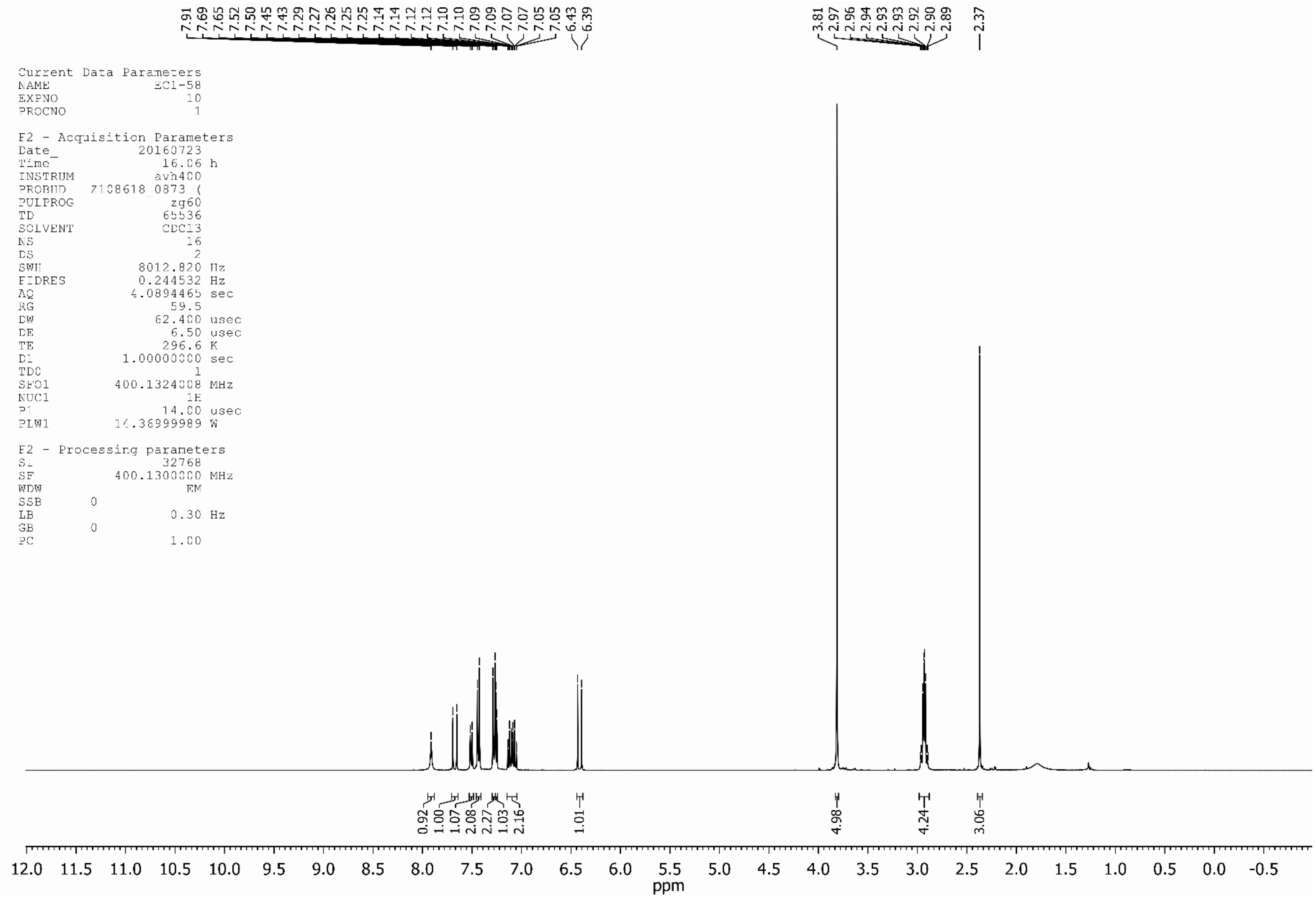

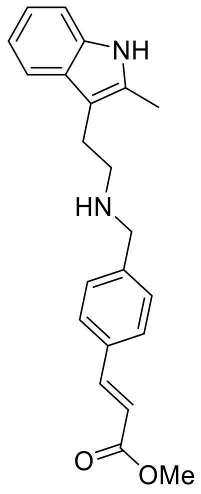

*Methyl (E)-3-(4-{[2-(2-methyl-1H-indol-3-yl)ethylamino]methyl}phenyl)prop-2-enoate (**3**)*

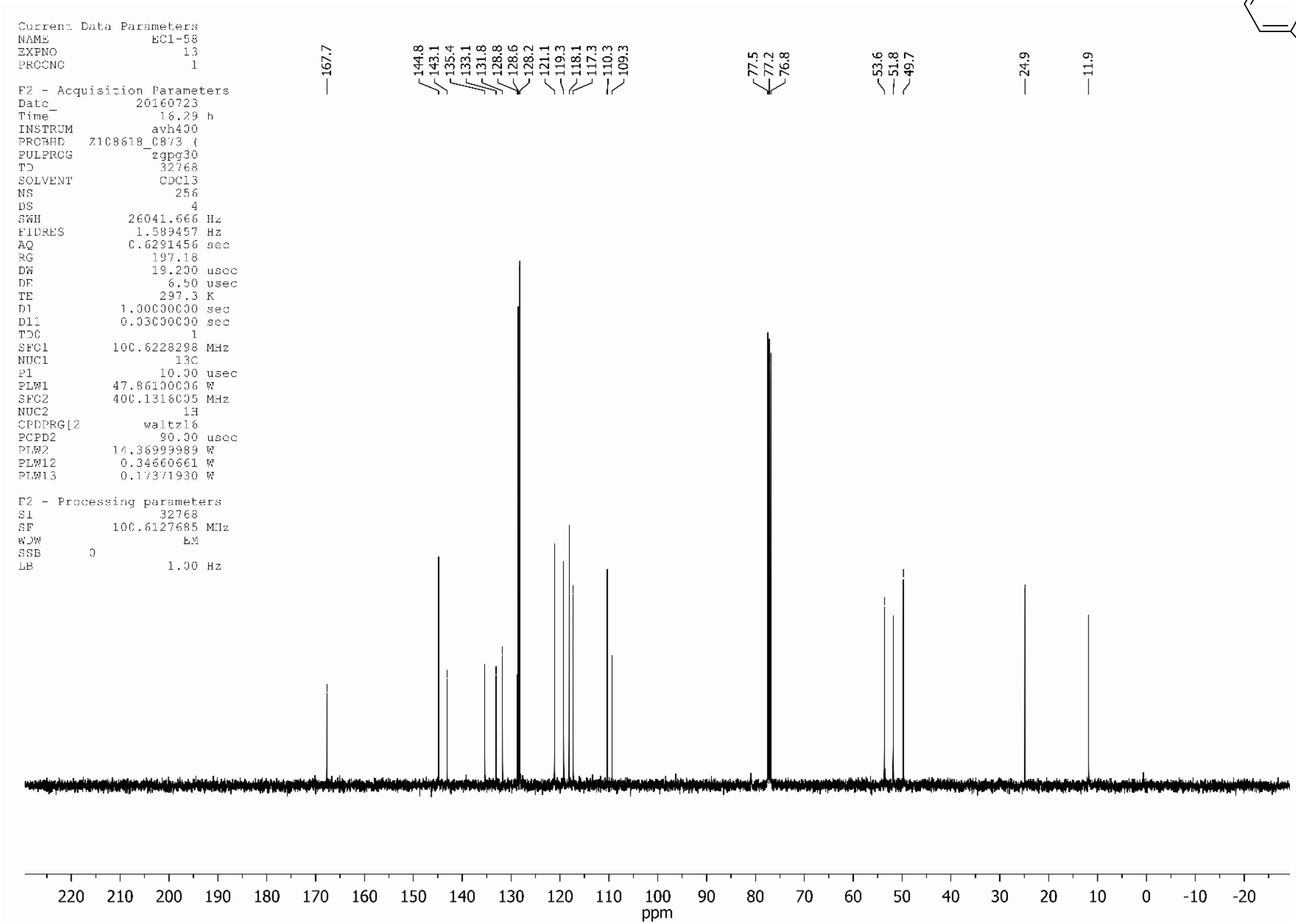

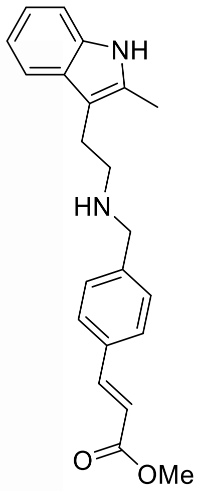

Panobinostat (Pano, **2**)

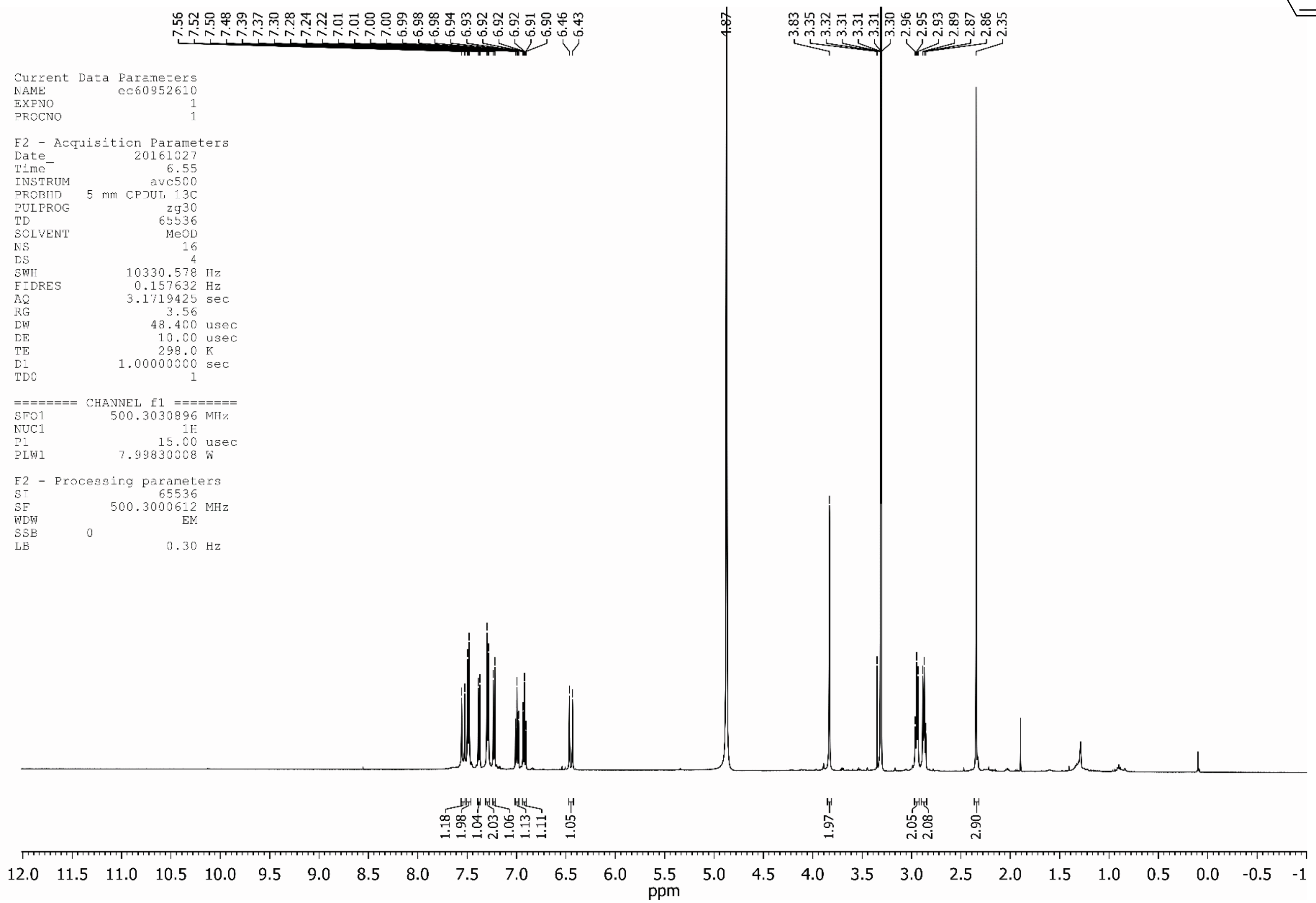

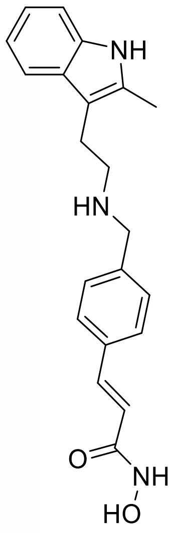

Panobinostat (Pano, **2**)

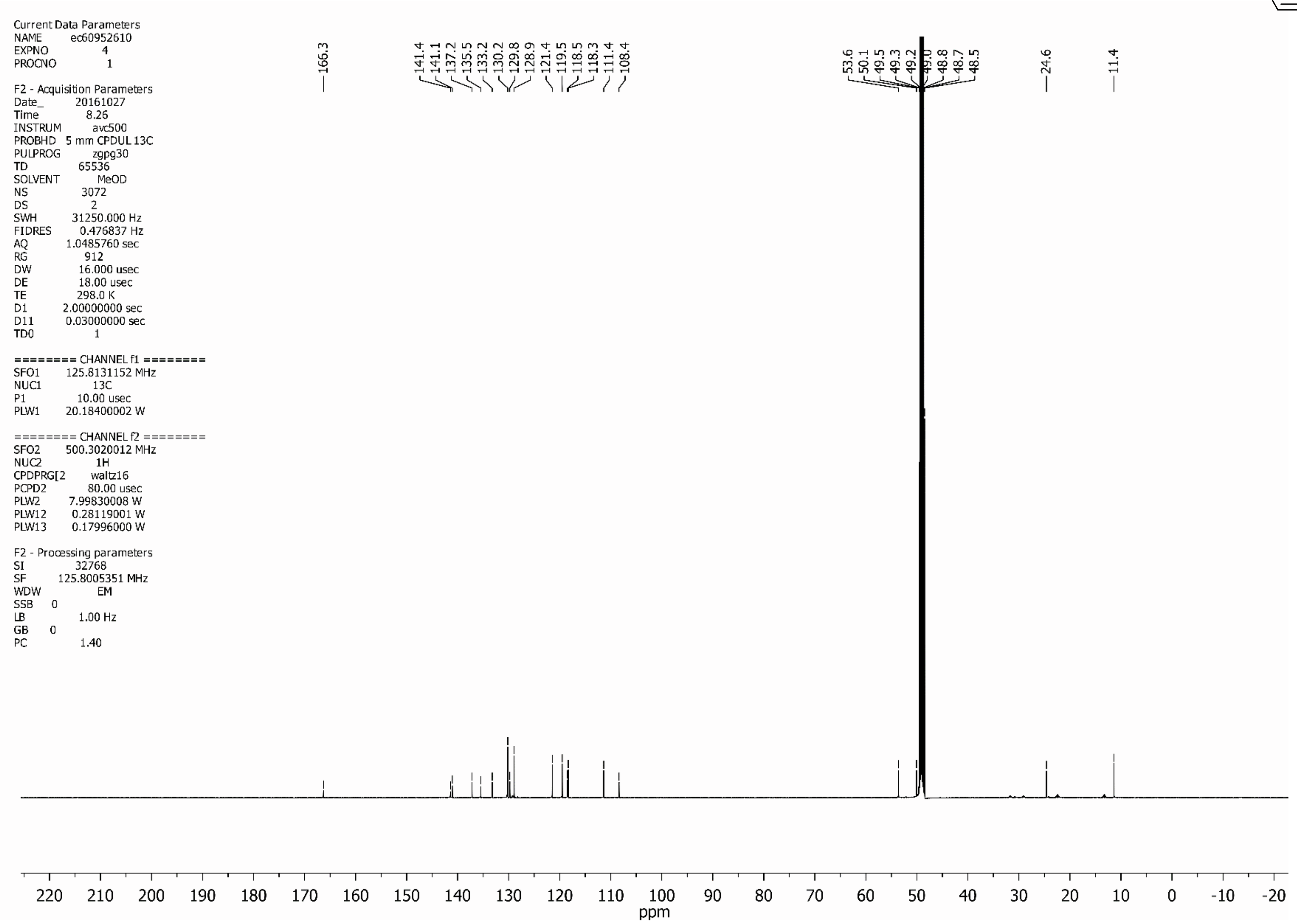

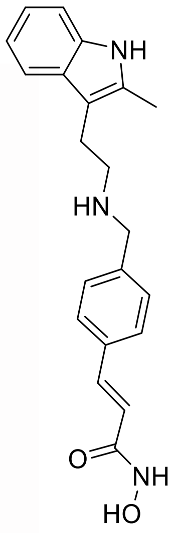

*(5-Nitrothiophen-2-yl)methyl bromide (**S9**)*

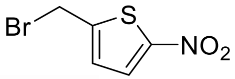

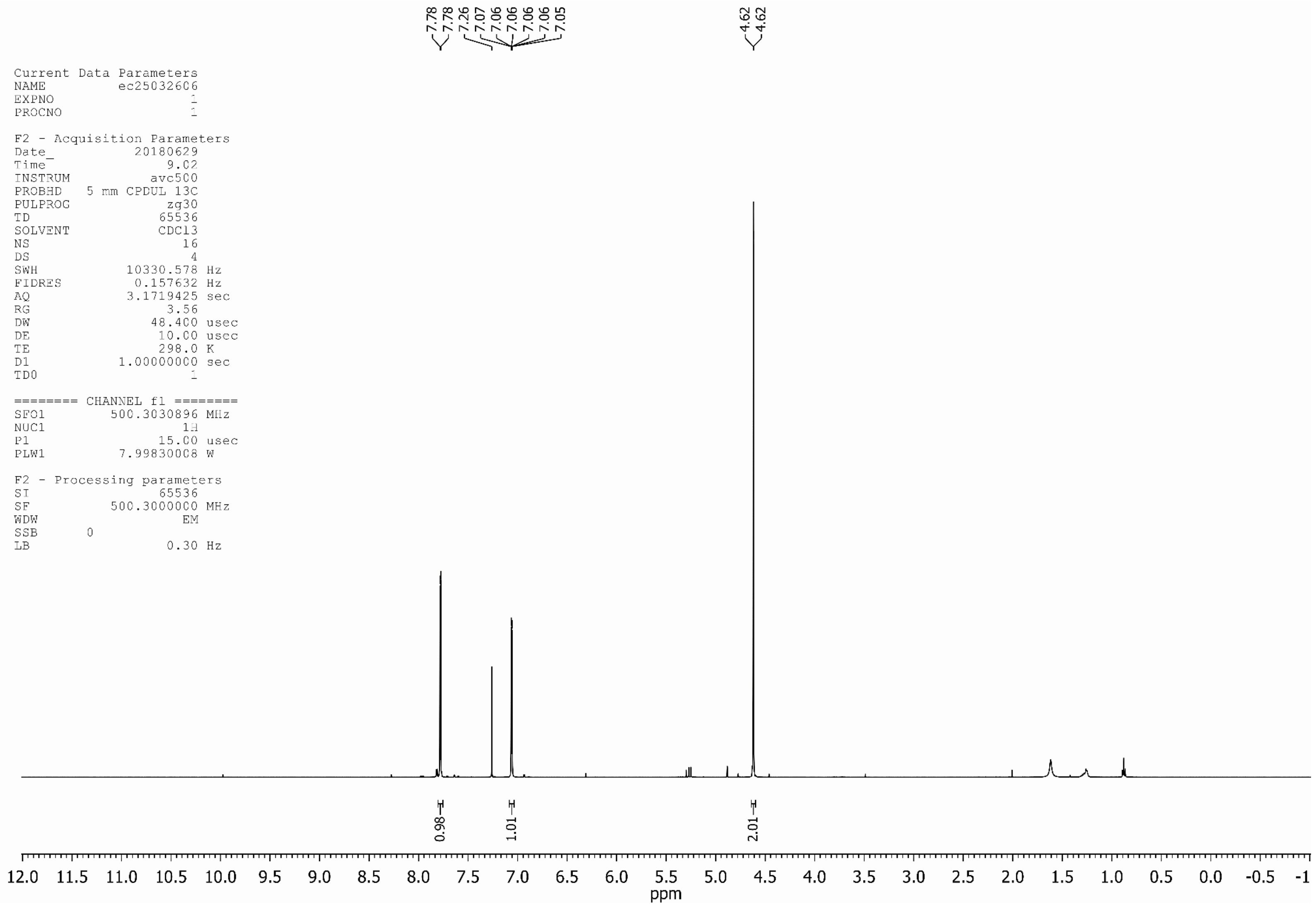

(5-Nitrothiophen-2-yl)methyl bromide (**S9**)

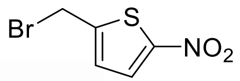

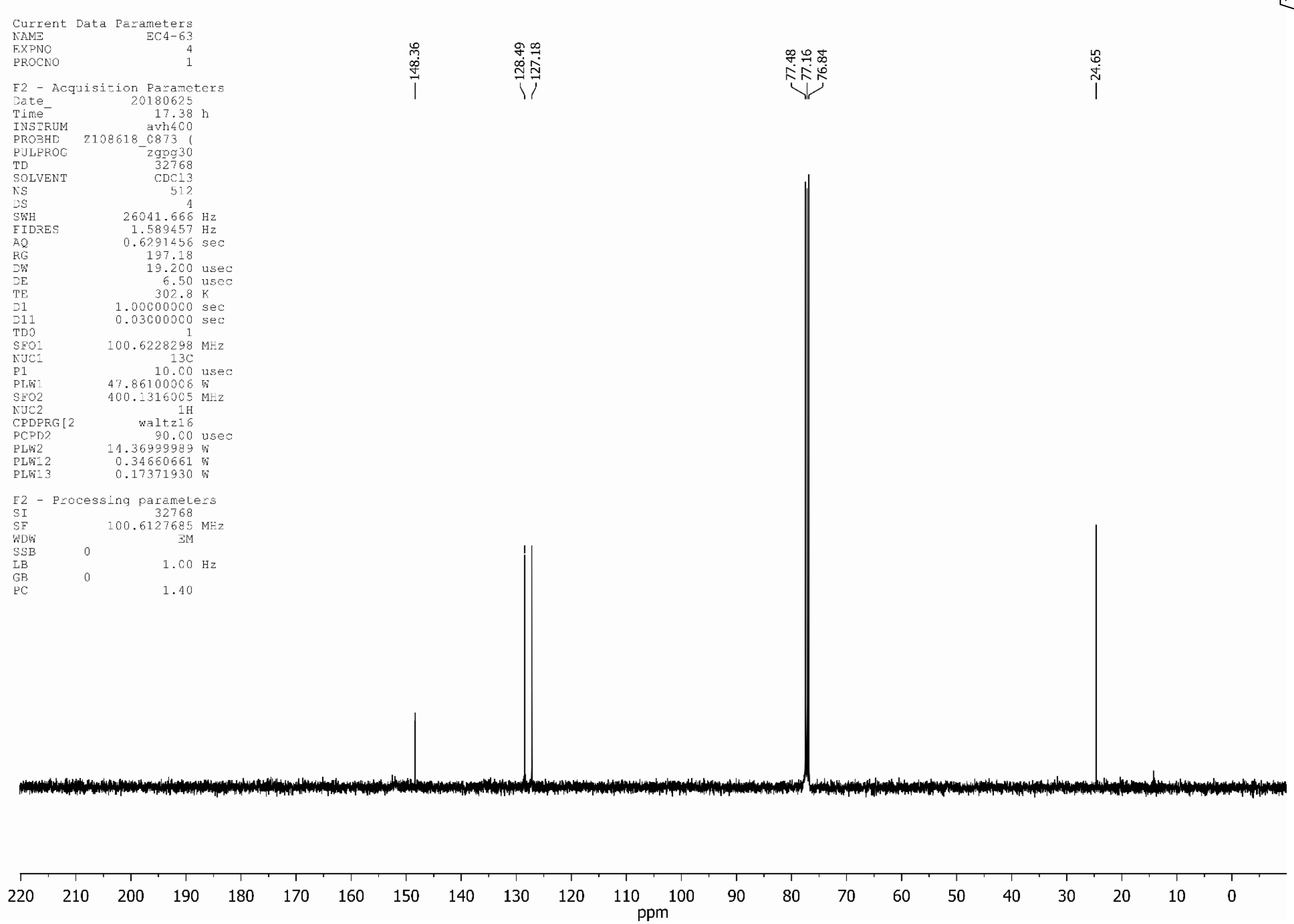

*(5-Nitrothiophen-2-yl)methyl bromide (**S9**)*

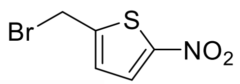

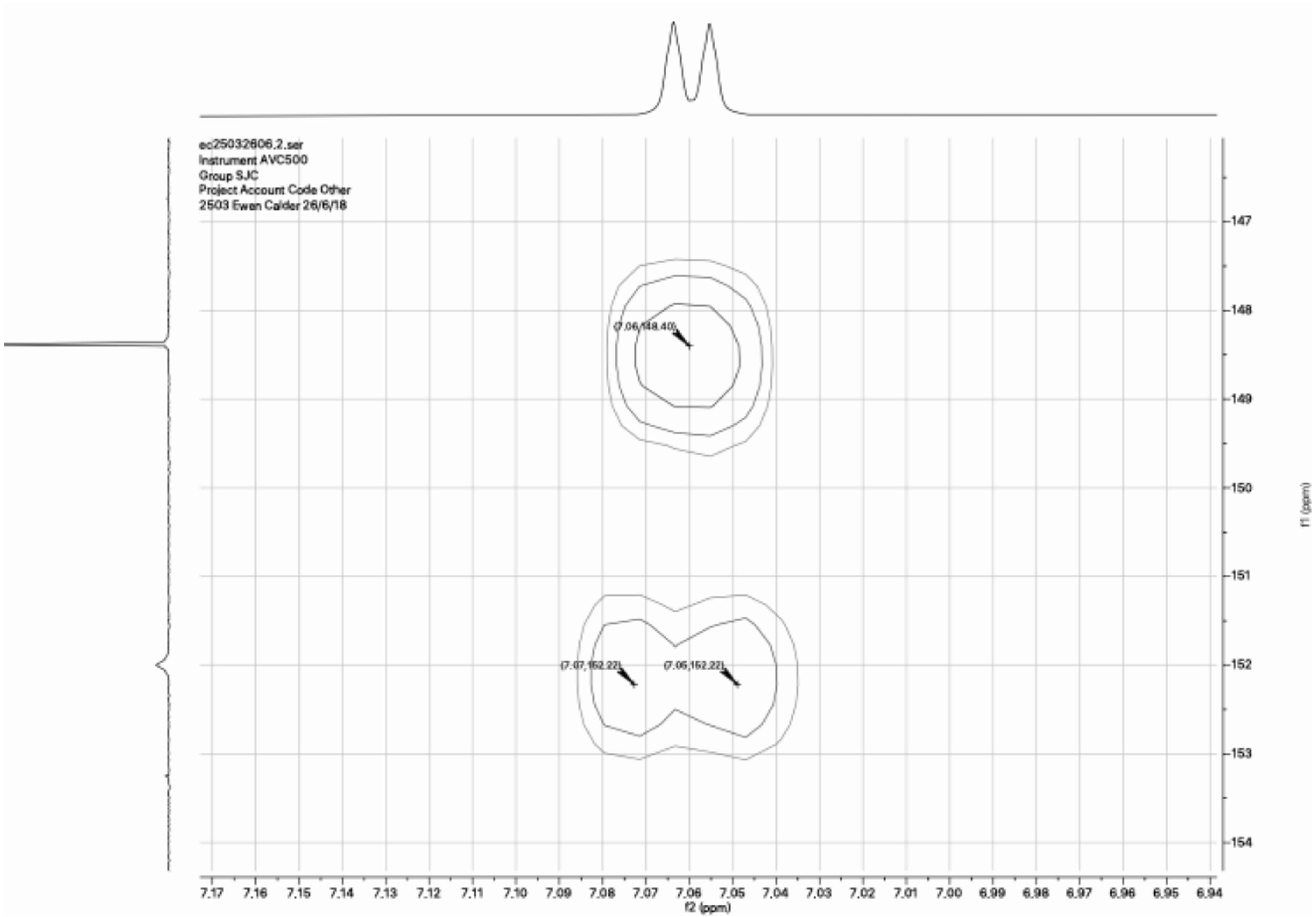

N-Phthalimido-*O*-(5-nitrothiophen-2-yl)-hydroxylamine

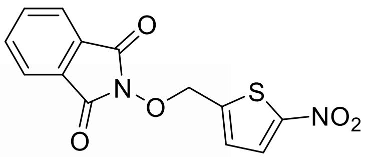

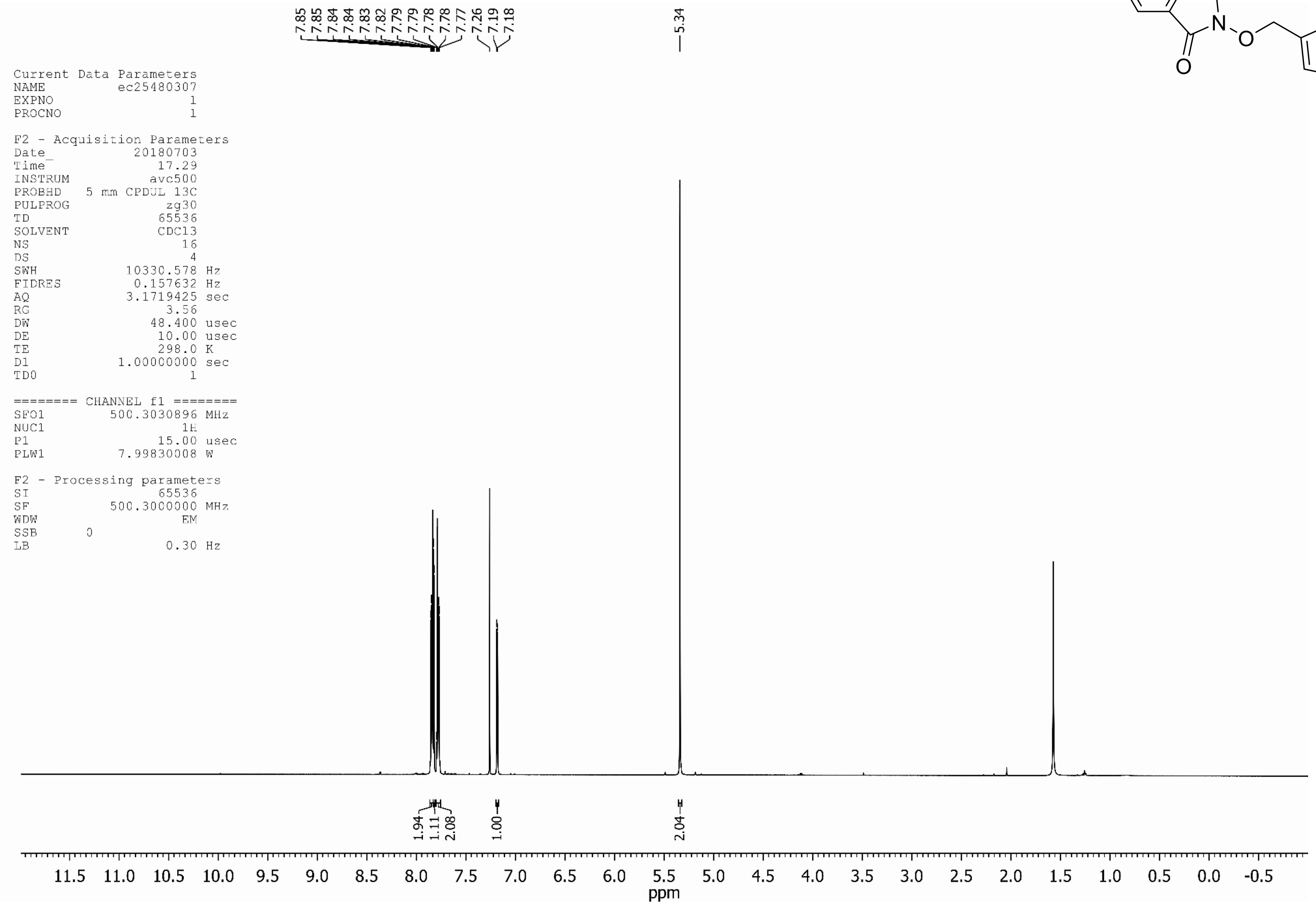

N-Phthalimido-*O*-(5-nitrothiophen-2-yl)-hydroxylamine

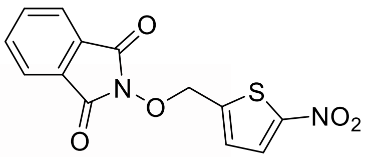

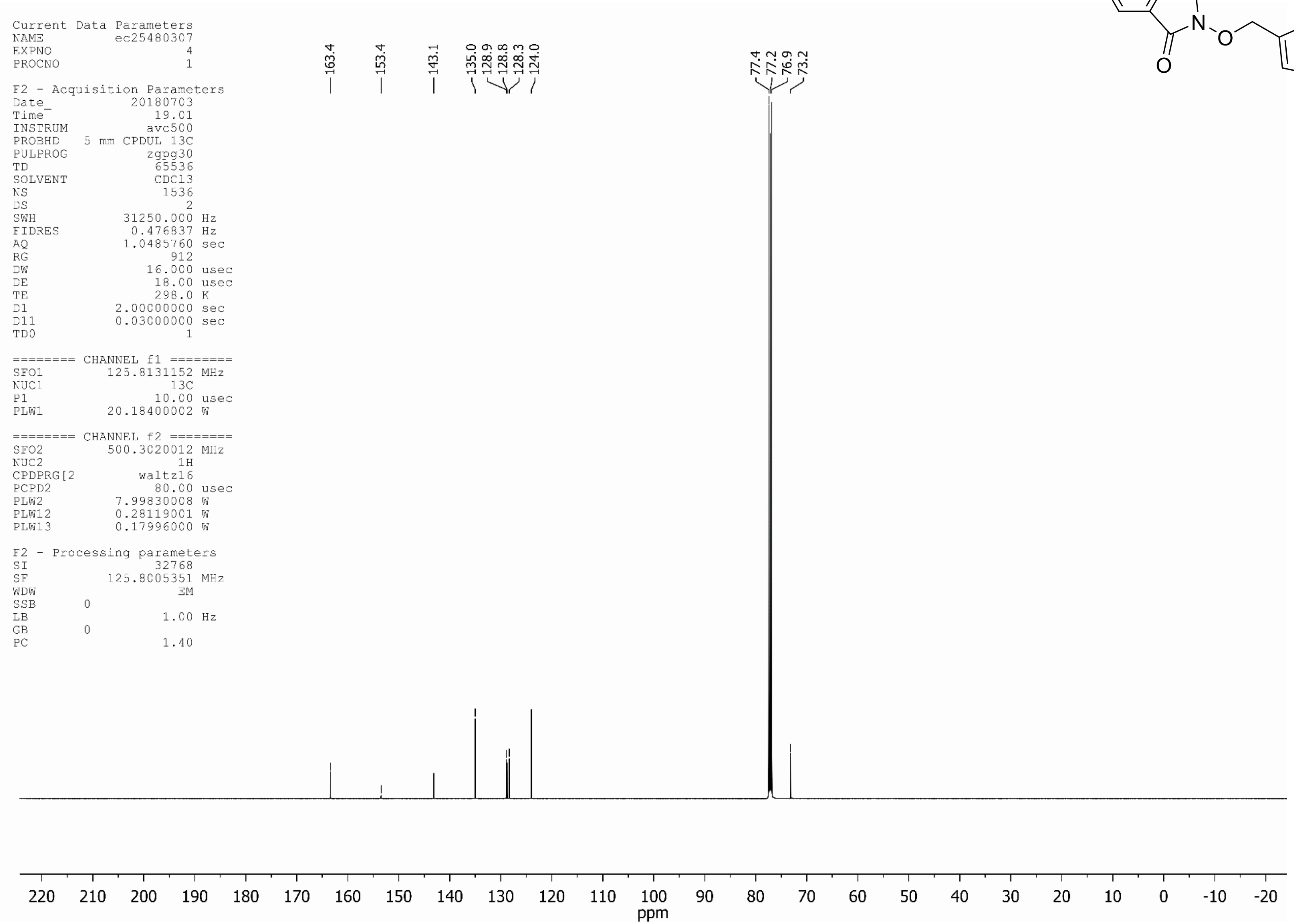

O-(5-Nitrothiophen-2-yl)-hydroxylamine (**S10**)

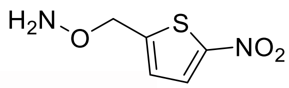

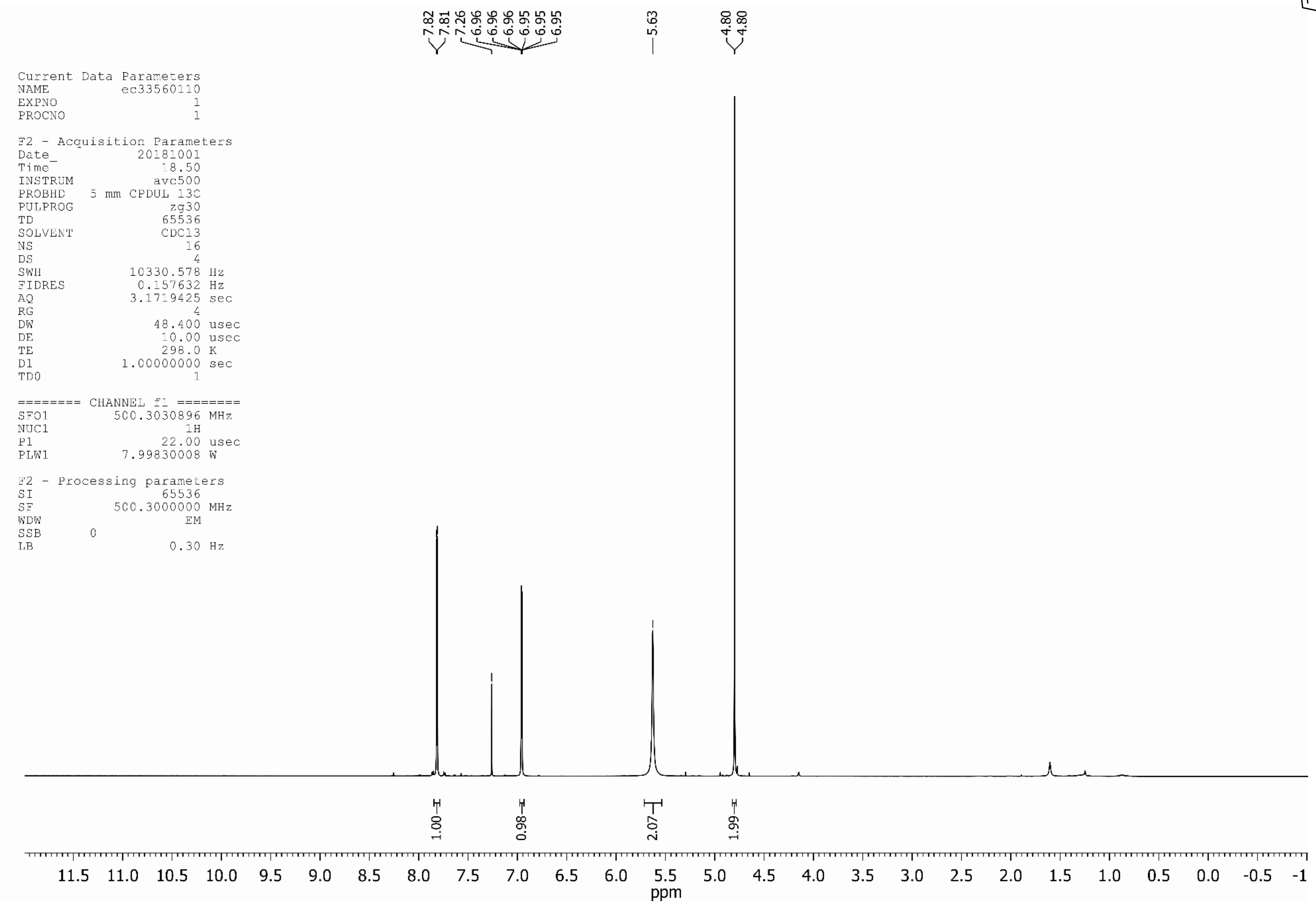

O-(5-Nitrothiophen-2-yl)-hydroxylamine (**S10**)

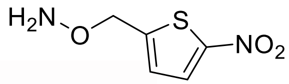

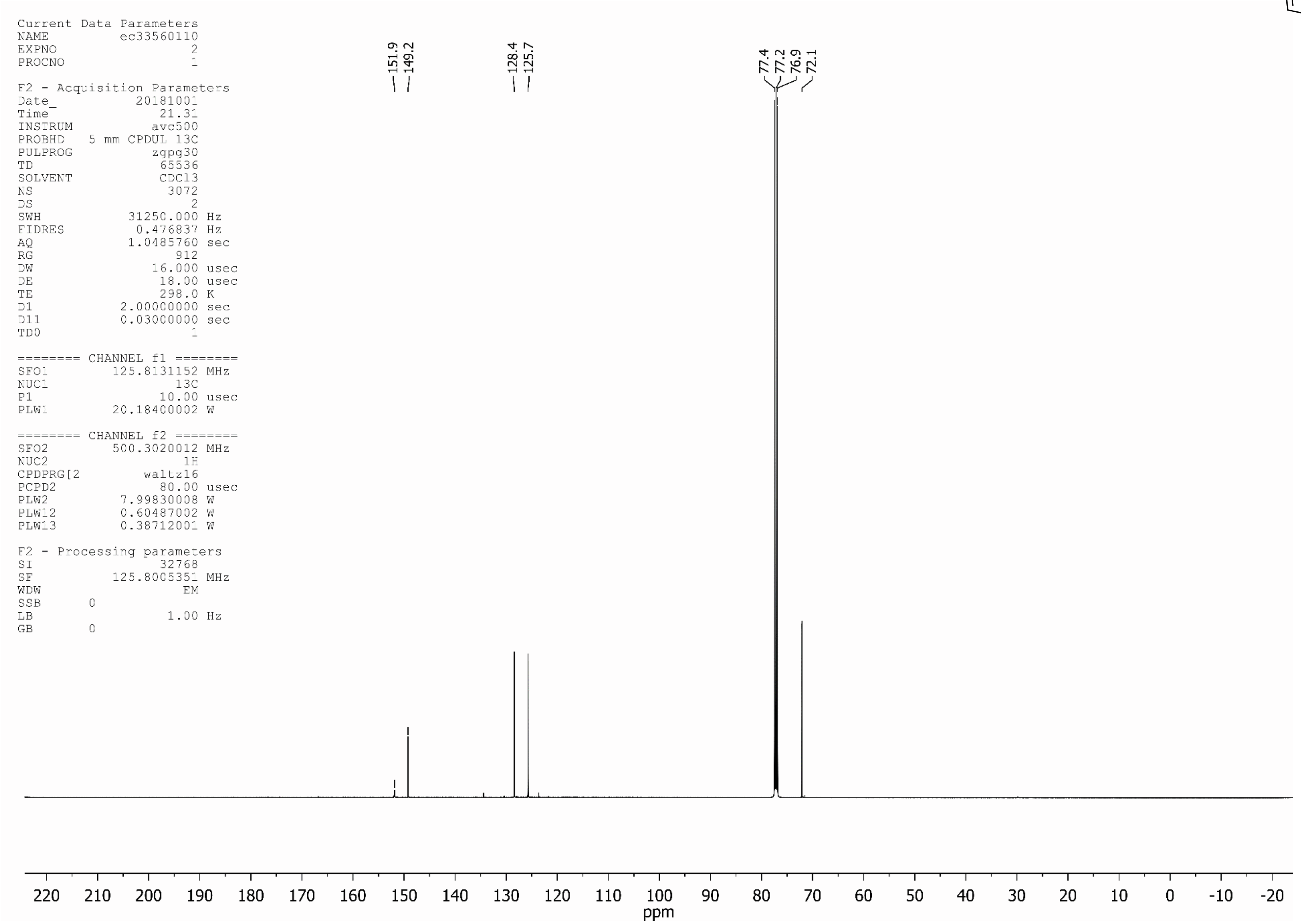

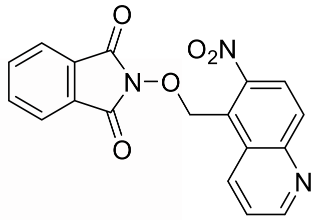

N-Phthalimido-*O*-(6-nitroquinolin-5-yl)-hydroxylamine

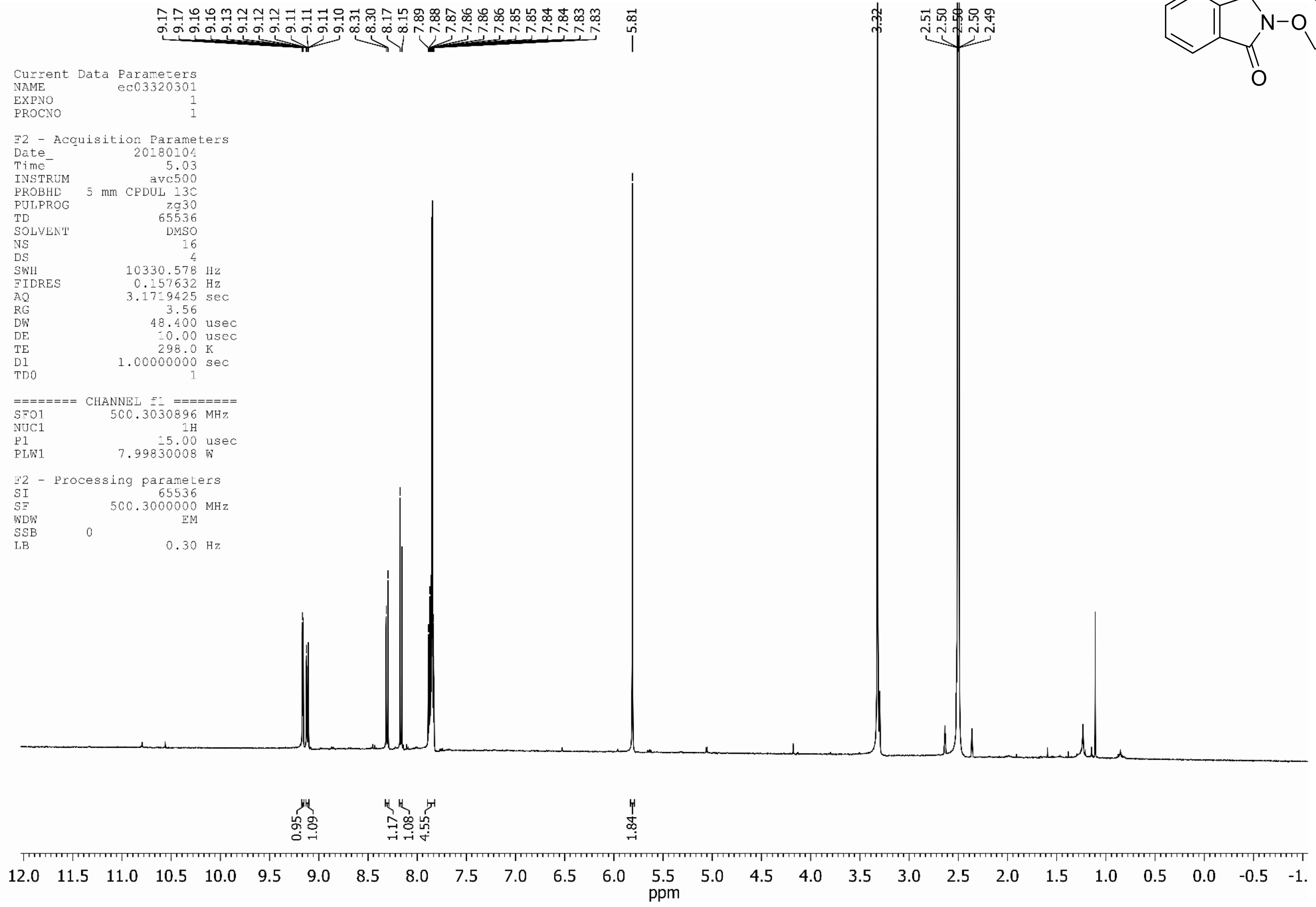

N-Phthalimido-O-(6-nitroquinolin-5-yl)-hydroxylamine

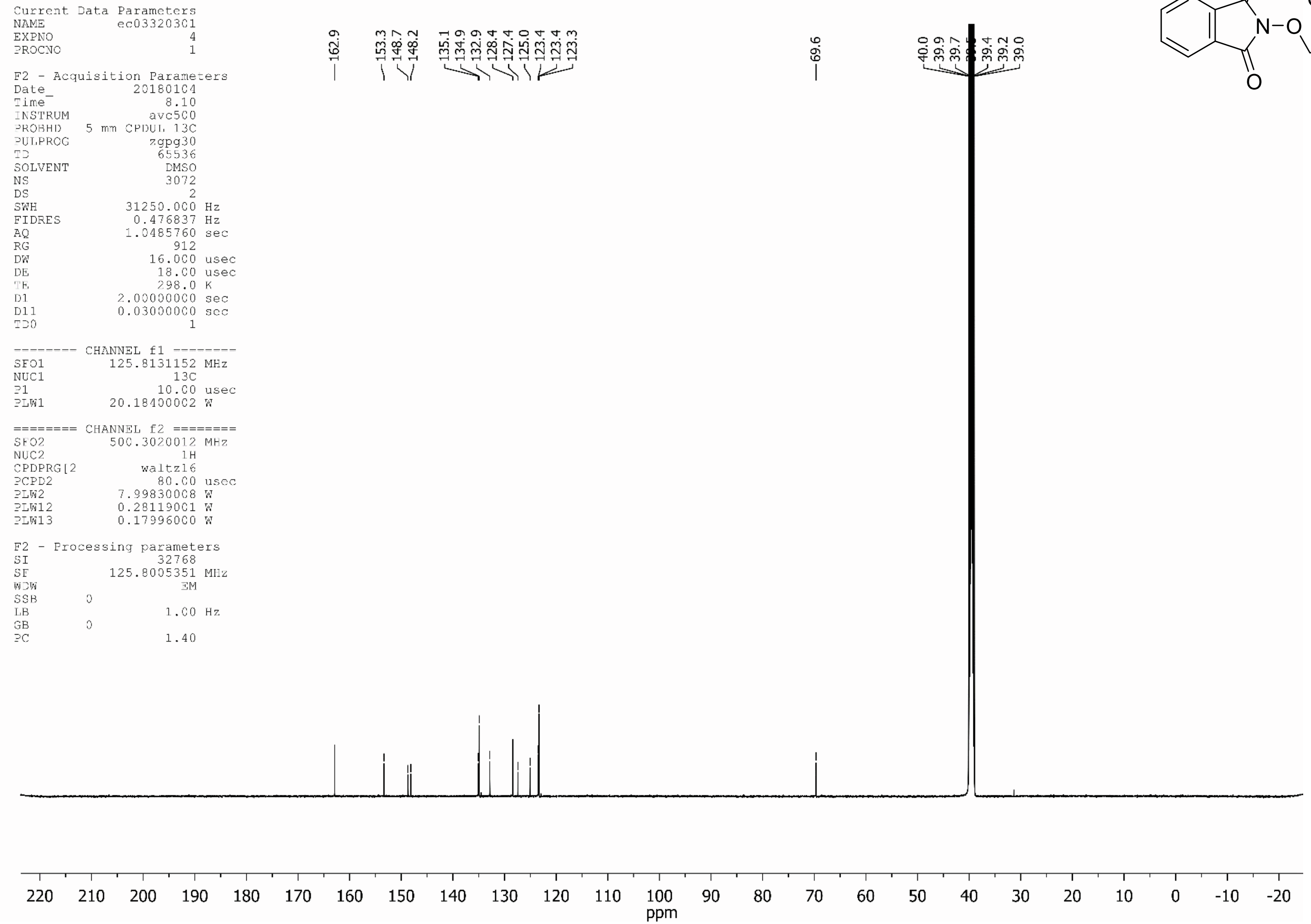

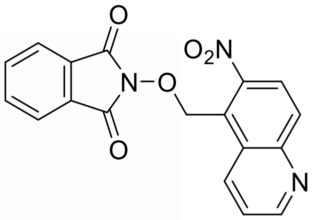

*O-(6-Nitroquinolin-5-yl)-hydroxylamine (**S14**)*

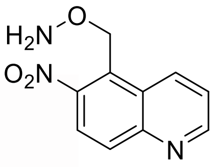

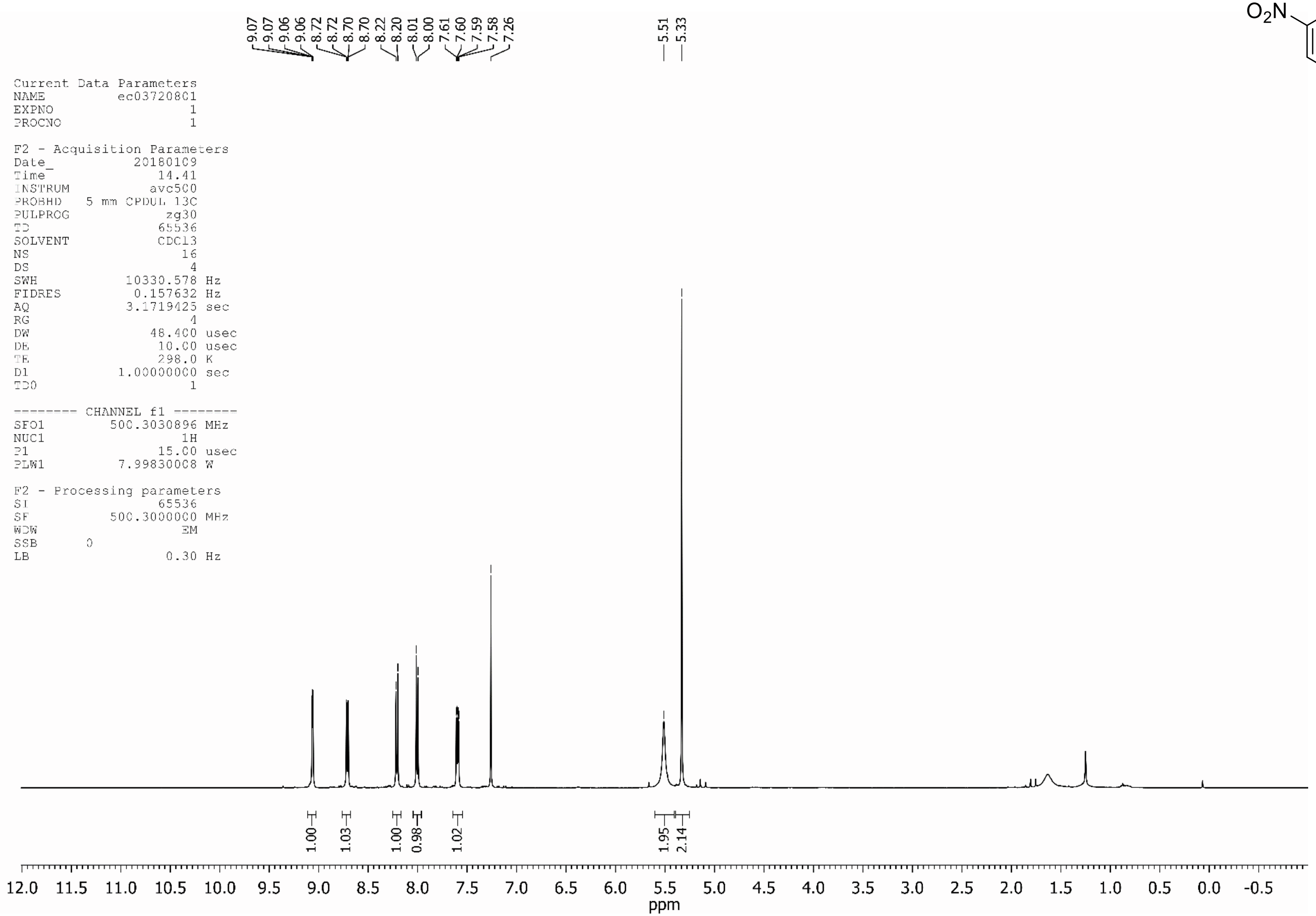

O-(6-Nitroquinolin-5-yl)-hydroxylamine (**S14**)

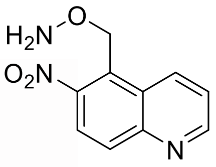

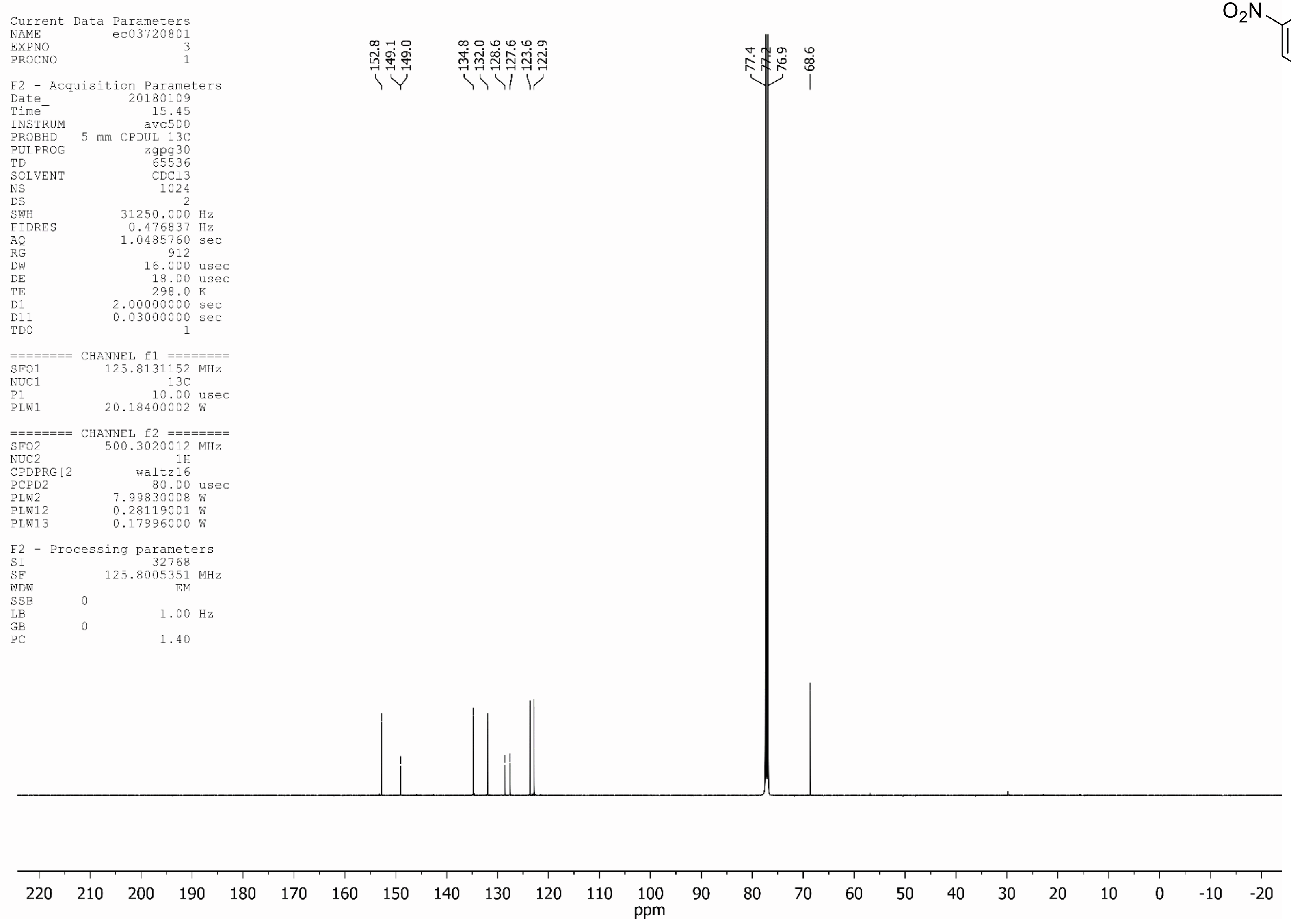

Methyl (*E*)-3-(4-{[*tert*-butyloxycarbonyl-(2-{1-[*tert*-butyloxycarbonyl]-2-methyl-*1H*-indol-3-yl}ethyl)amino]methyl}phenyl)prop-2-enoate (**4**)

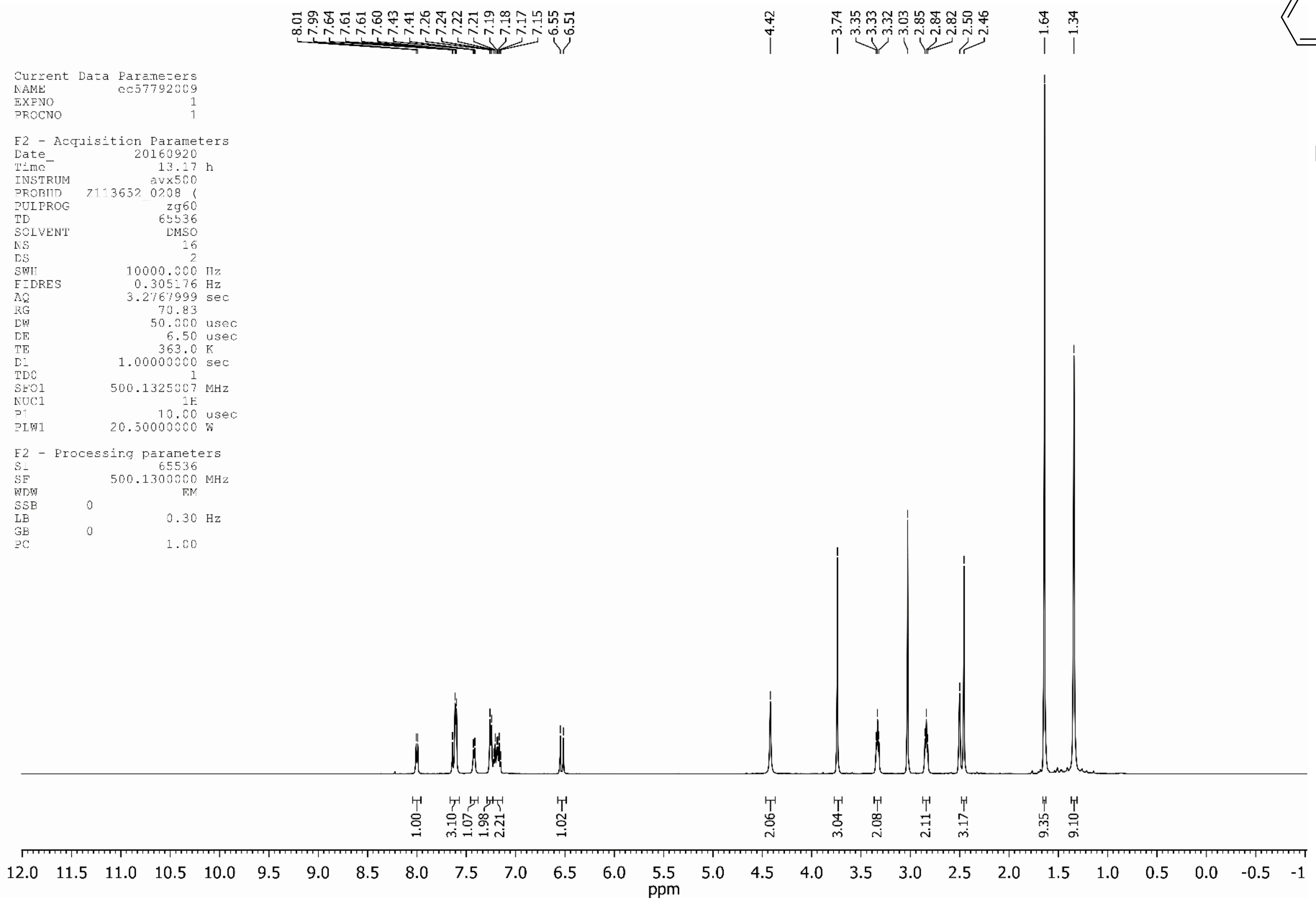

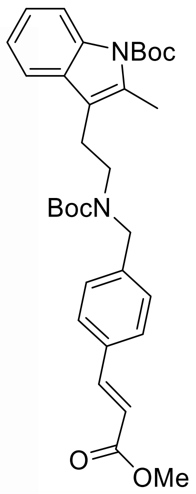

Methyl (*E*)-3-(4-{[*tert*-butyloxycarbonyl-(2-{1-[*tert*-butyloxycarbonyl]-2-methyl-*1H*-indol-3-yl}ethyl)amino]methyl}phenyl)prop-2-enoate (**4**)

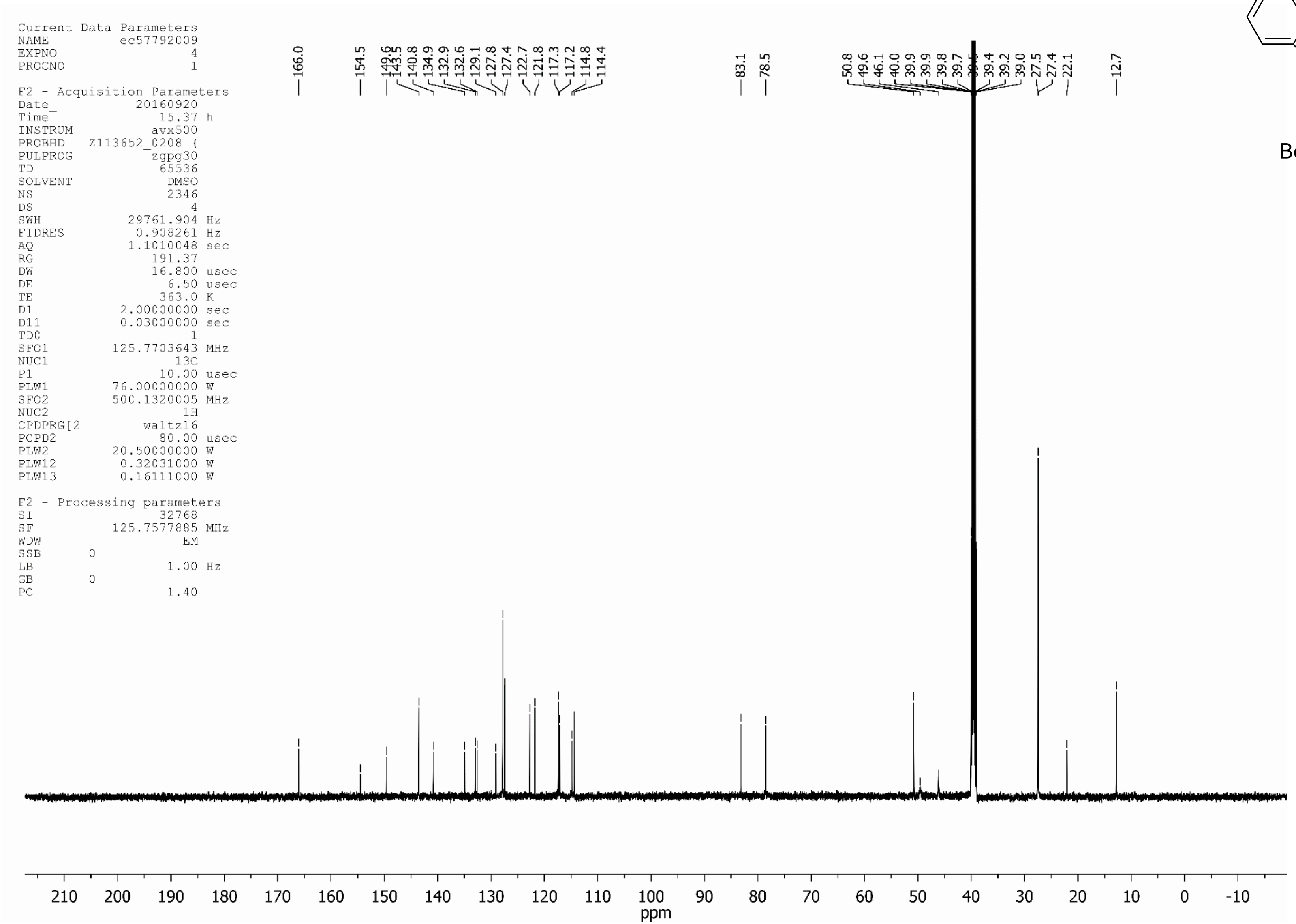

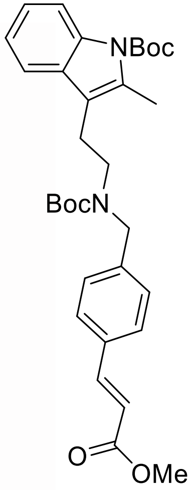

(*E*)-3-(4-{[*tert*-Butyloxycarbonyl-(2-{1-[*tert*-butyloxycarbonyl]-2-methyl-*1H*-indol-3-yl}ethyl)amino]methyl}phenyl)prop-2-enoic acid (**5**)

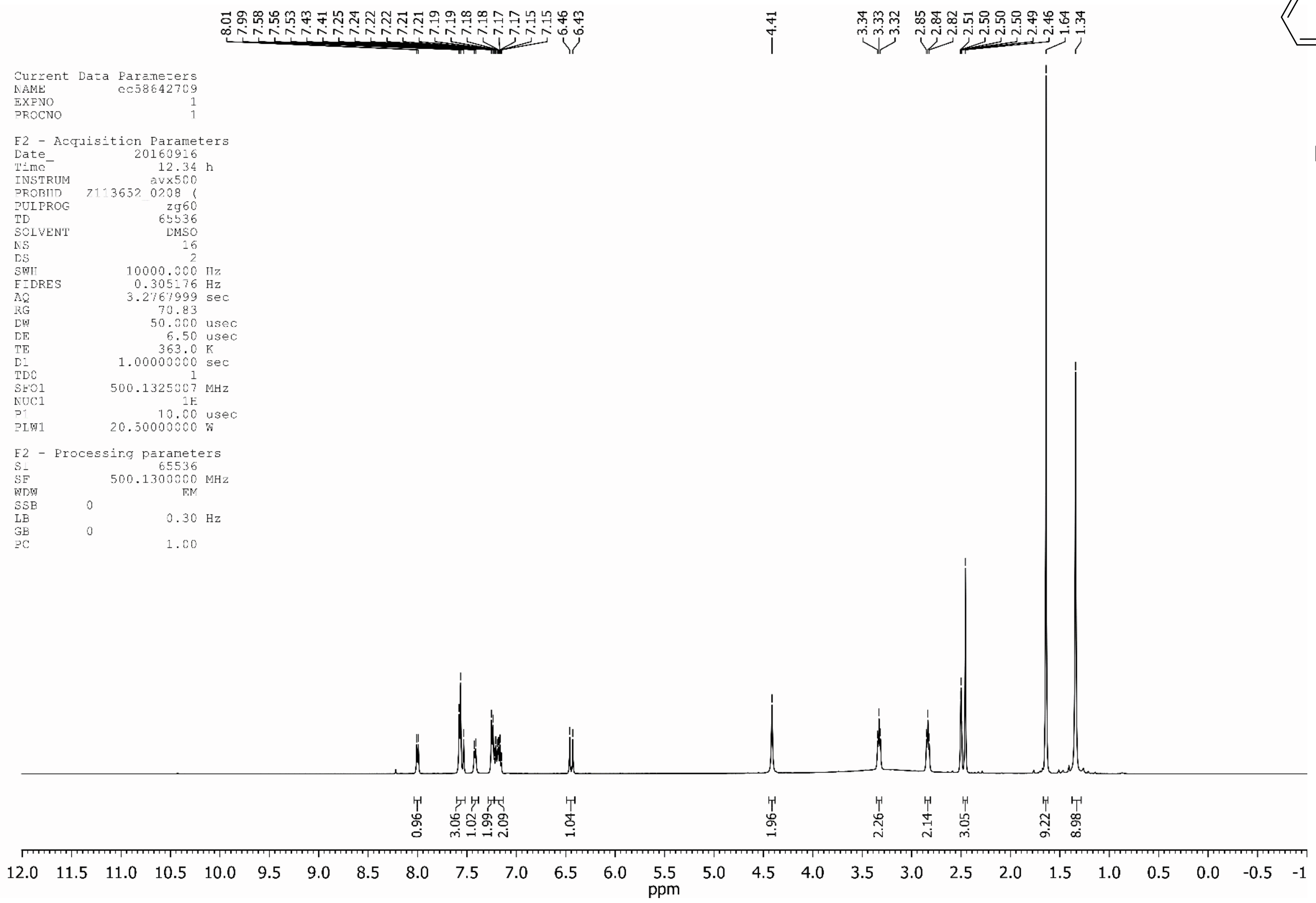

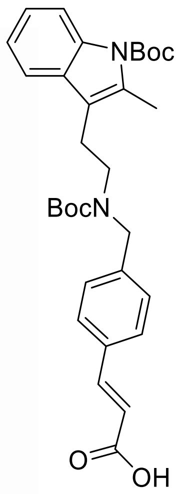

(*E*)-3-(4-{[*tert*-Butyloxycarbonyl-(2-{1-[*tert*-butyloxycarbonyl]-2-methyl-*1H*-indol-3-yl}ethyl)amino]methyl}phenyl)prop-2-enoic acid (**5**)

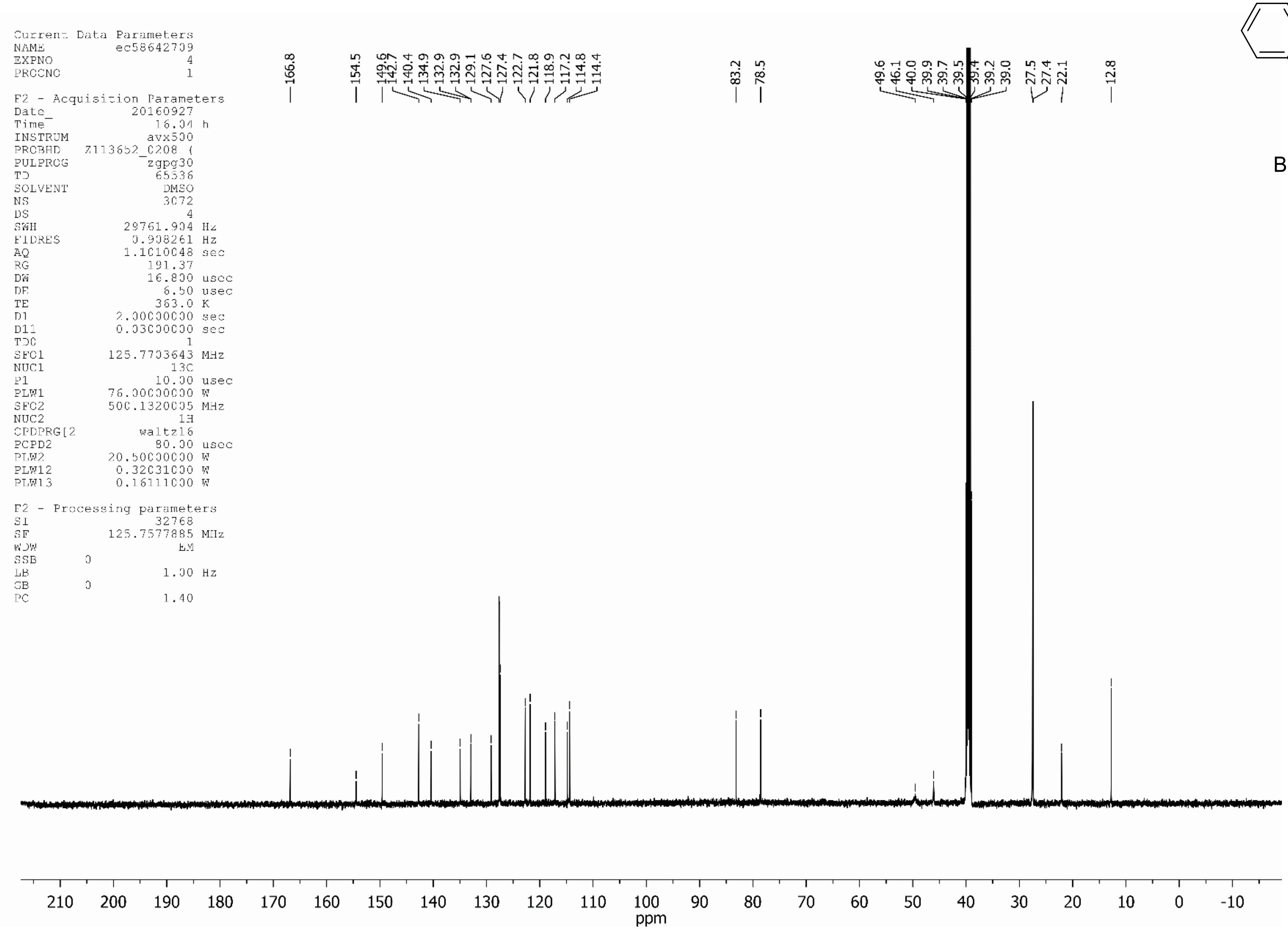

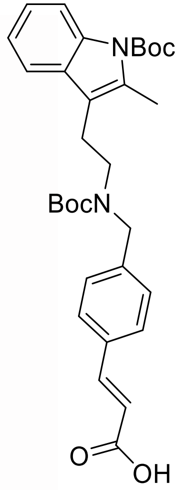

N,N-Di-Boc-*O*-benzyl-Panobinostat (**6**)

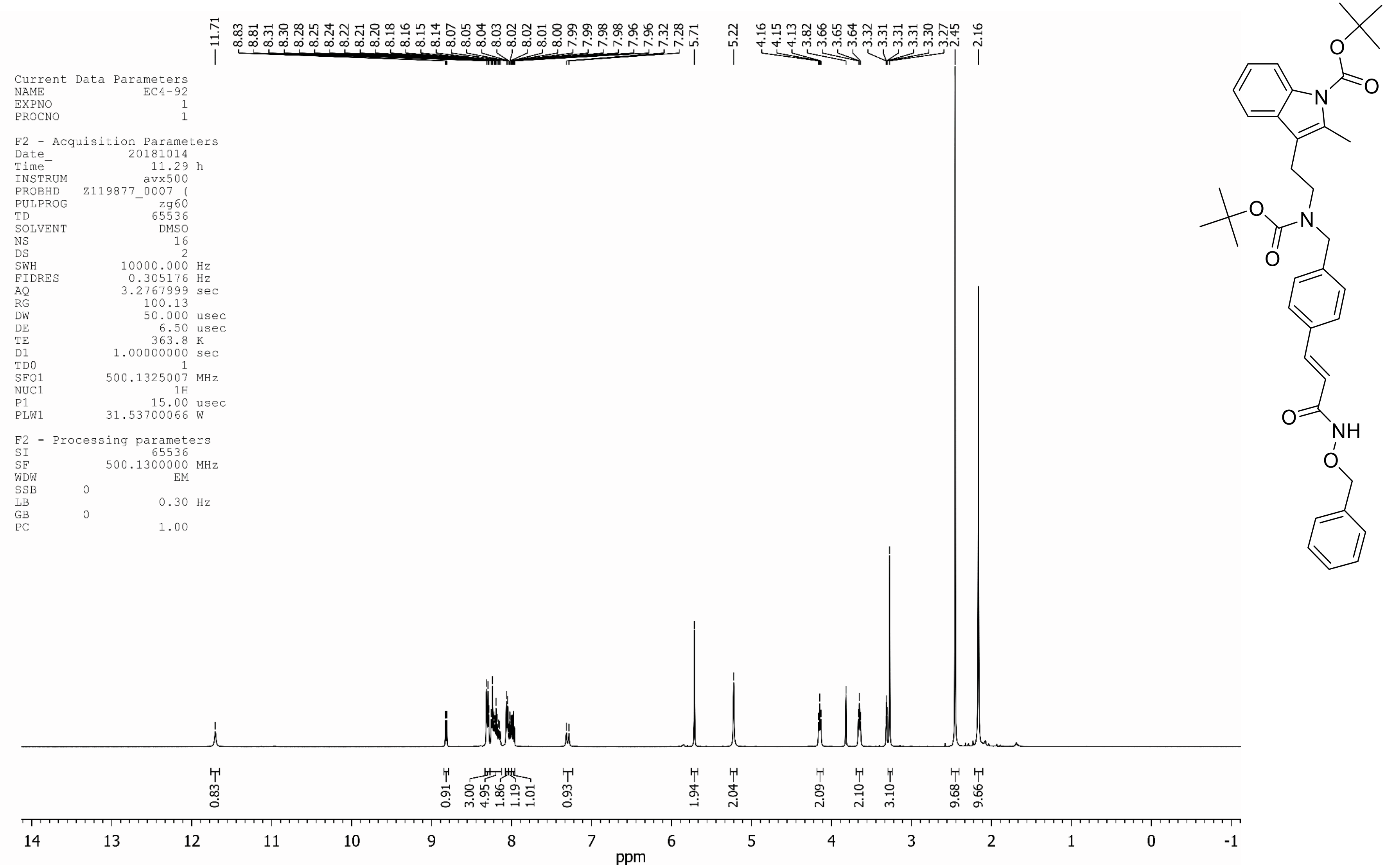

N,N-DiBoc-*O*-benzyl-Panobinostat (**6**)

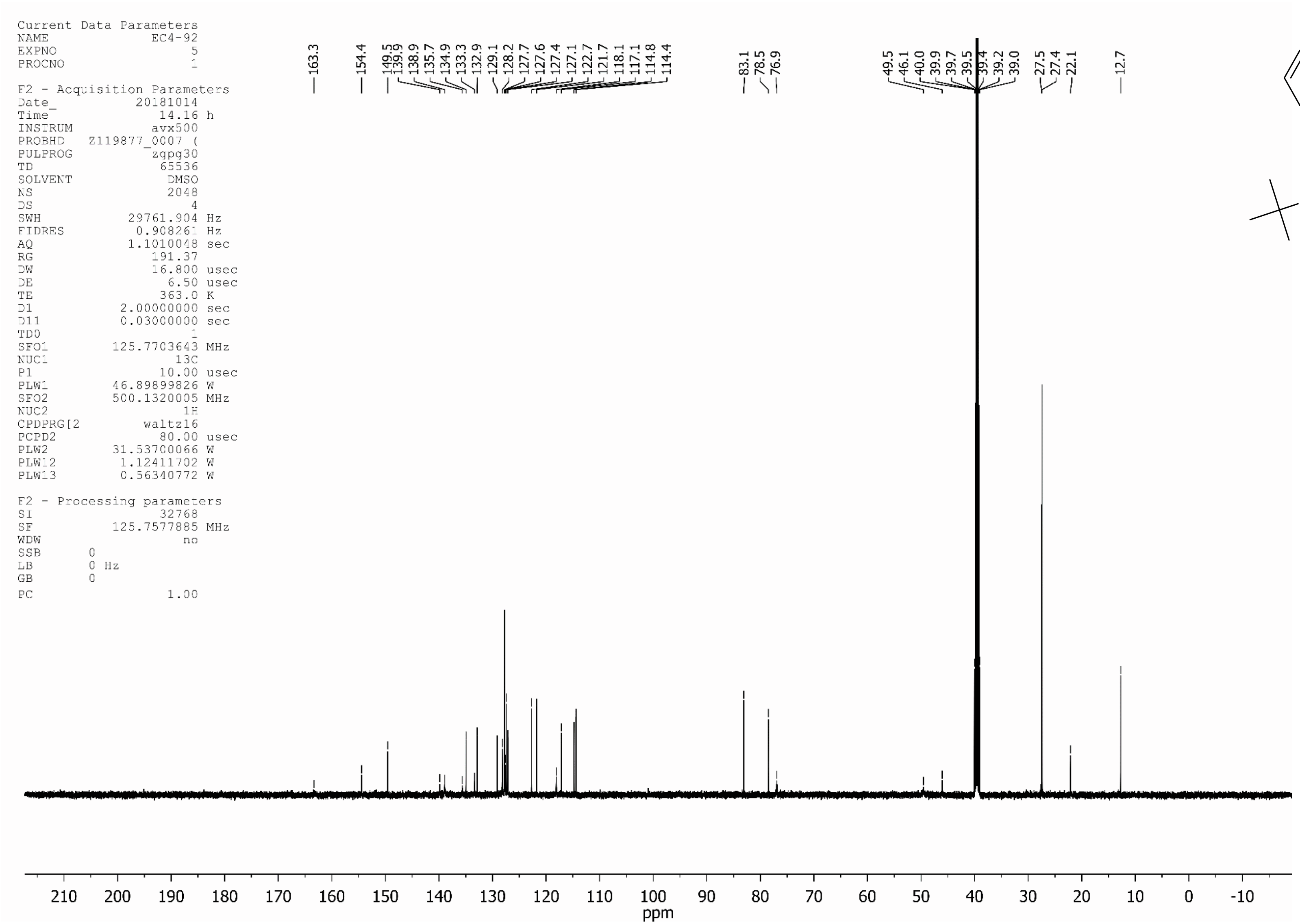

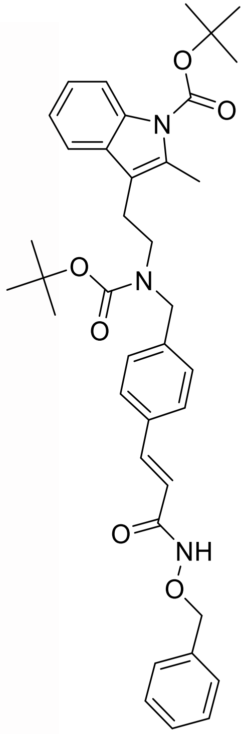

N,N-Di-Boc-*O*-benzyl-Panobinostat (**6**)

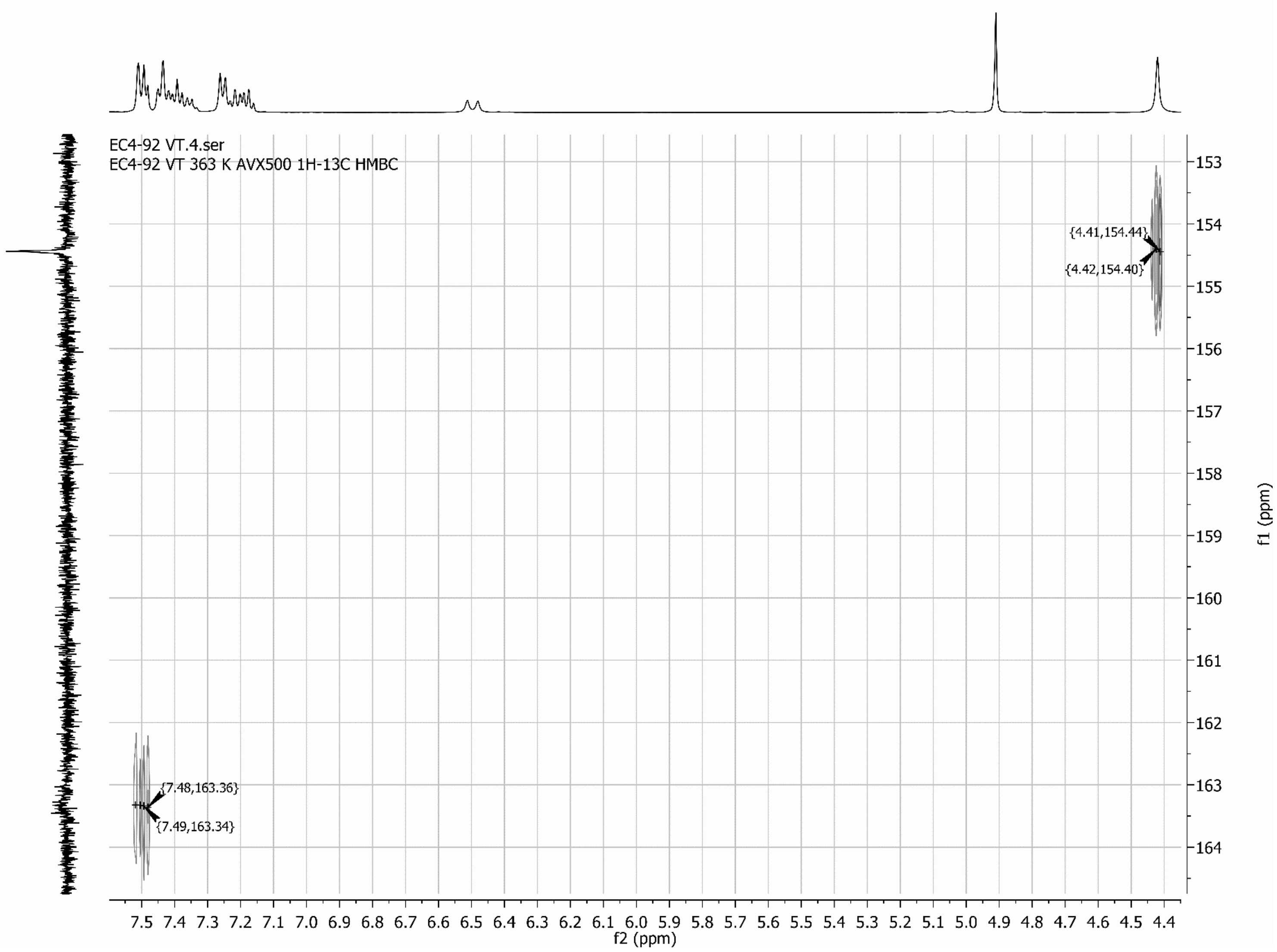

N,N-Di-Boc-*O*-(4-nitrobenzyl)-Panobinostat (**7**)

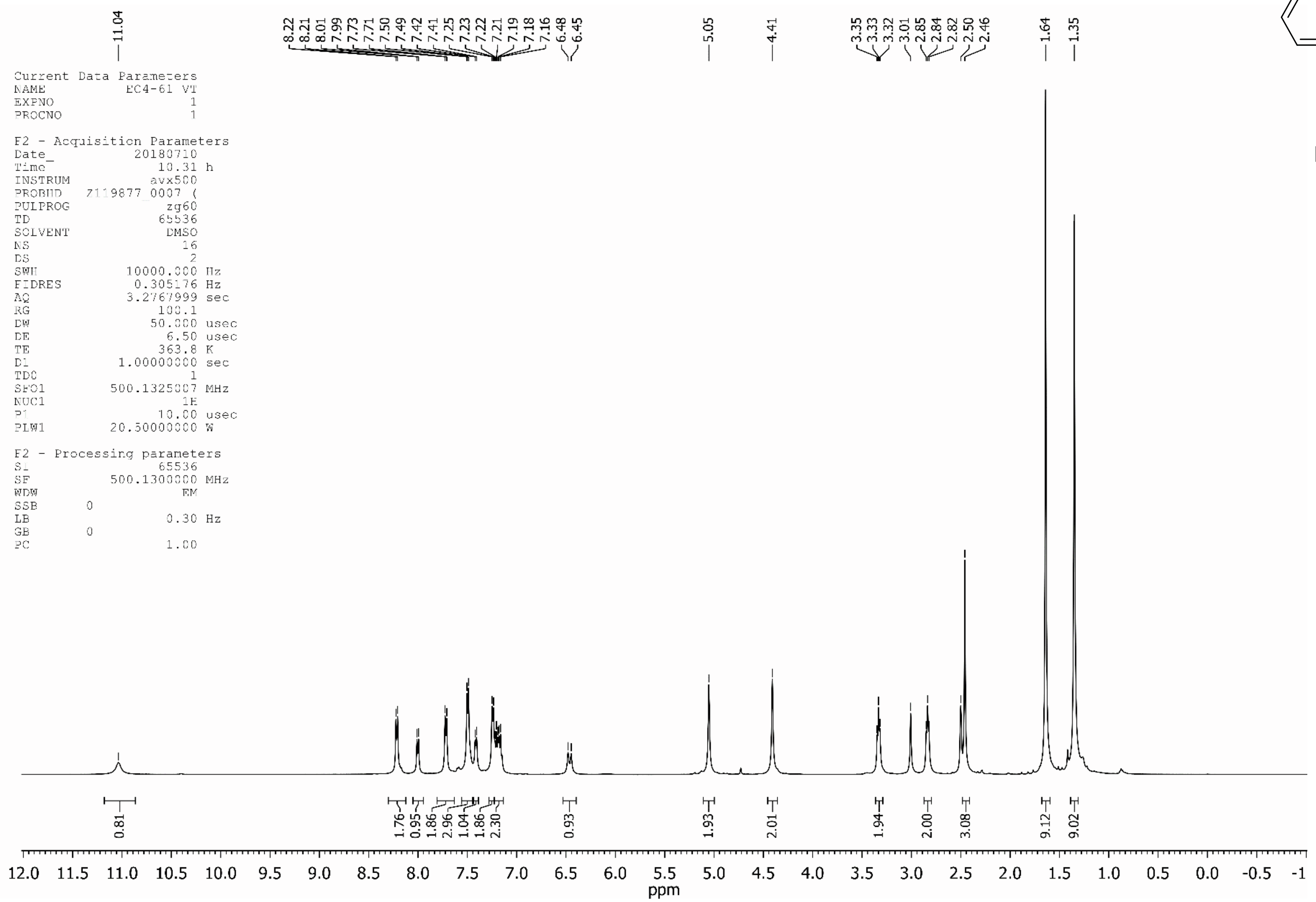

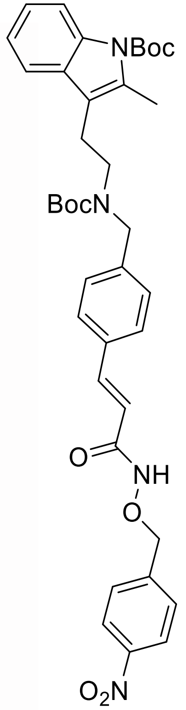

N,N-Di-Boc-*O*-(4-nitrobenzyl)-Panobinostat (**7**)

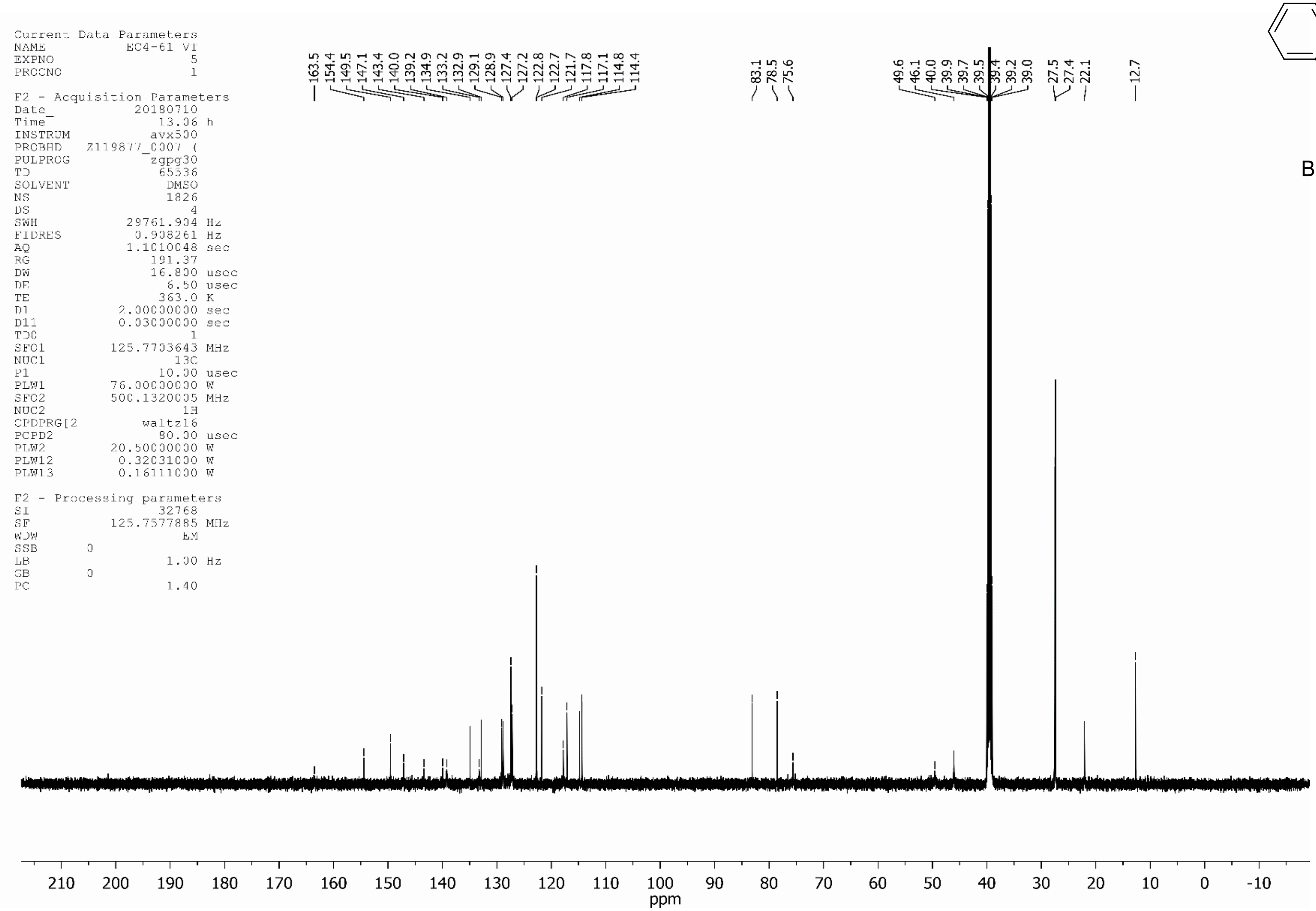

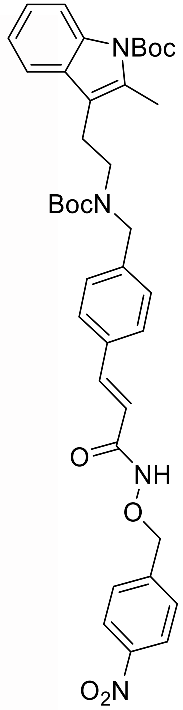

*N,N-Di-Boc-O-(4-nitrobenzyl)-Panobinostat (**7**)*

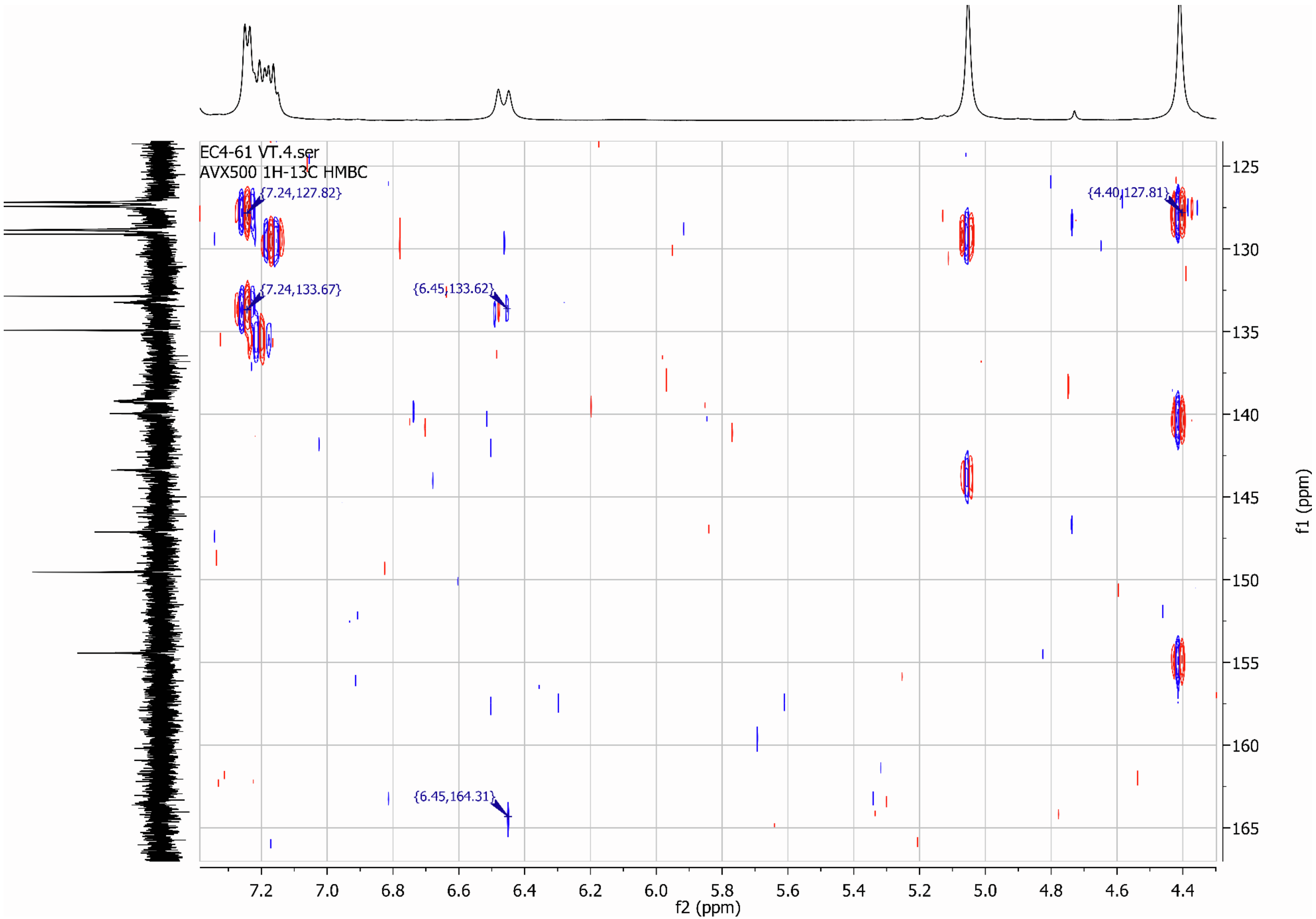

N,N-Di-Boc-*O*-(4-nitrobenzyl)-Panobinostat (**7**)

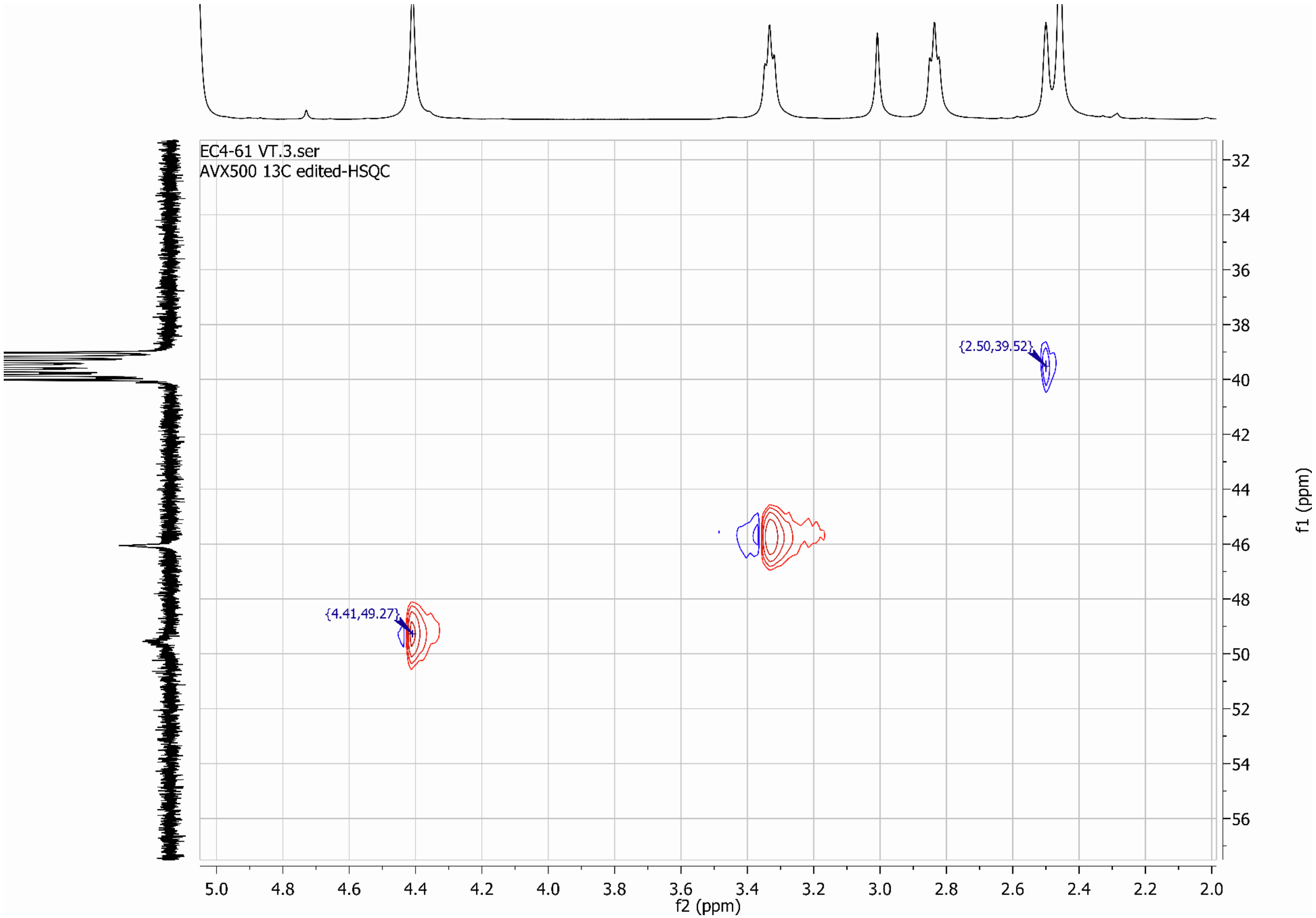

N,N-Di-Boc-*O*-(2-nitrothiophen-5-yl)methyl)-Panobinostat (**8**)

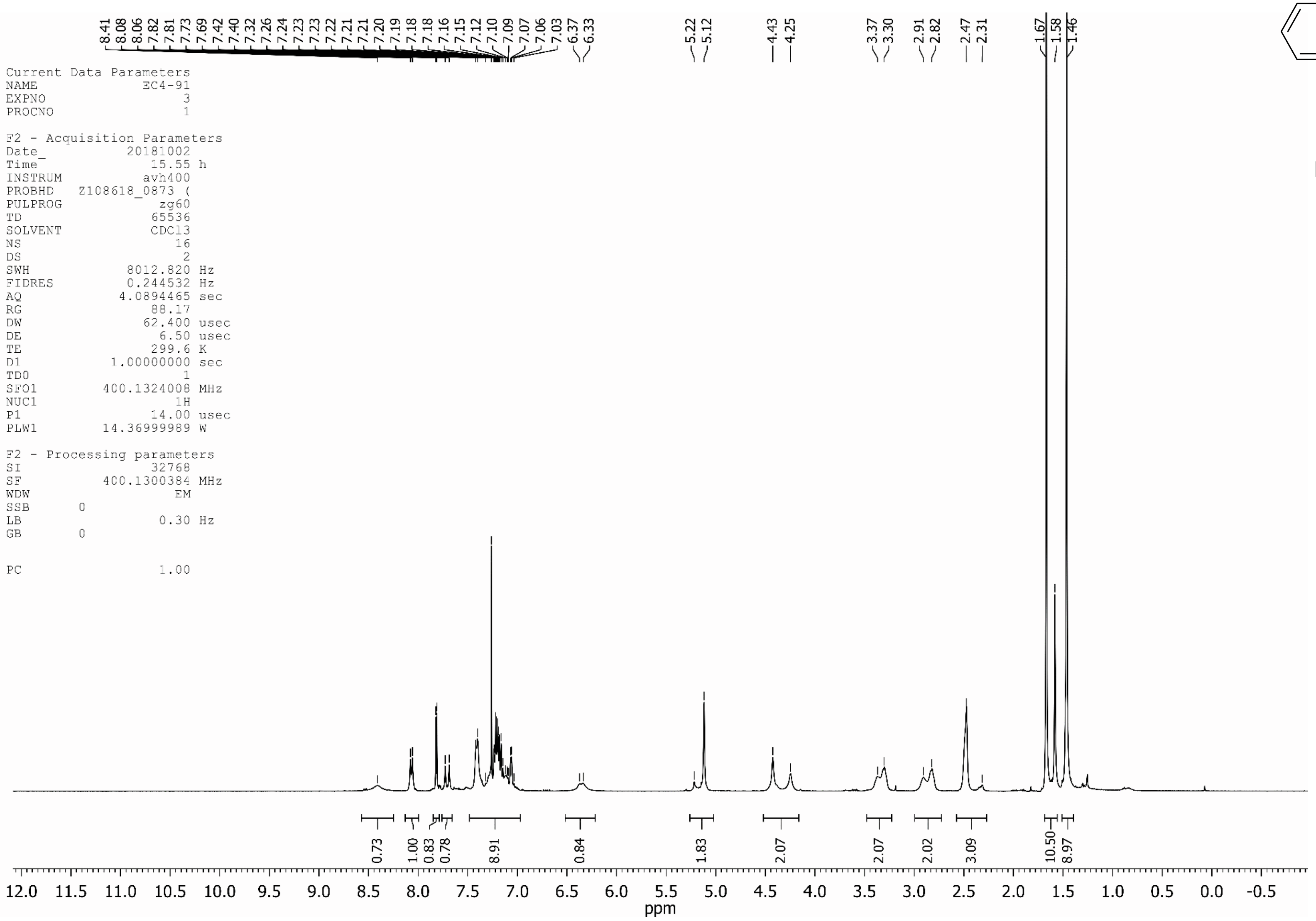

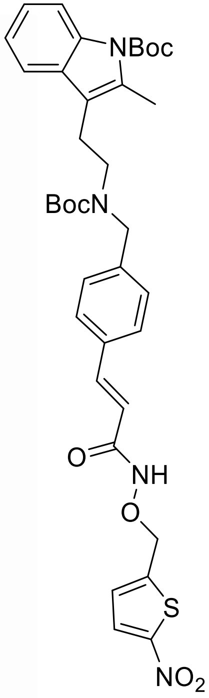

N,N-Di-Boc-*O*-(6-nitroquinolin-5-yl)methyl)-Panobinostat (**9**)

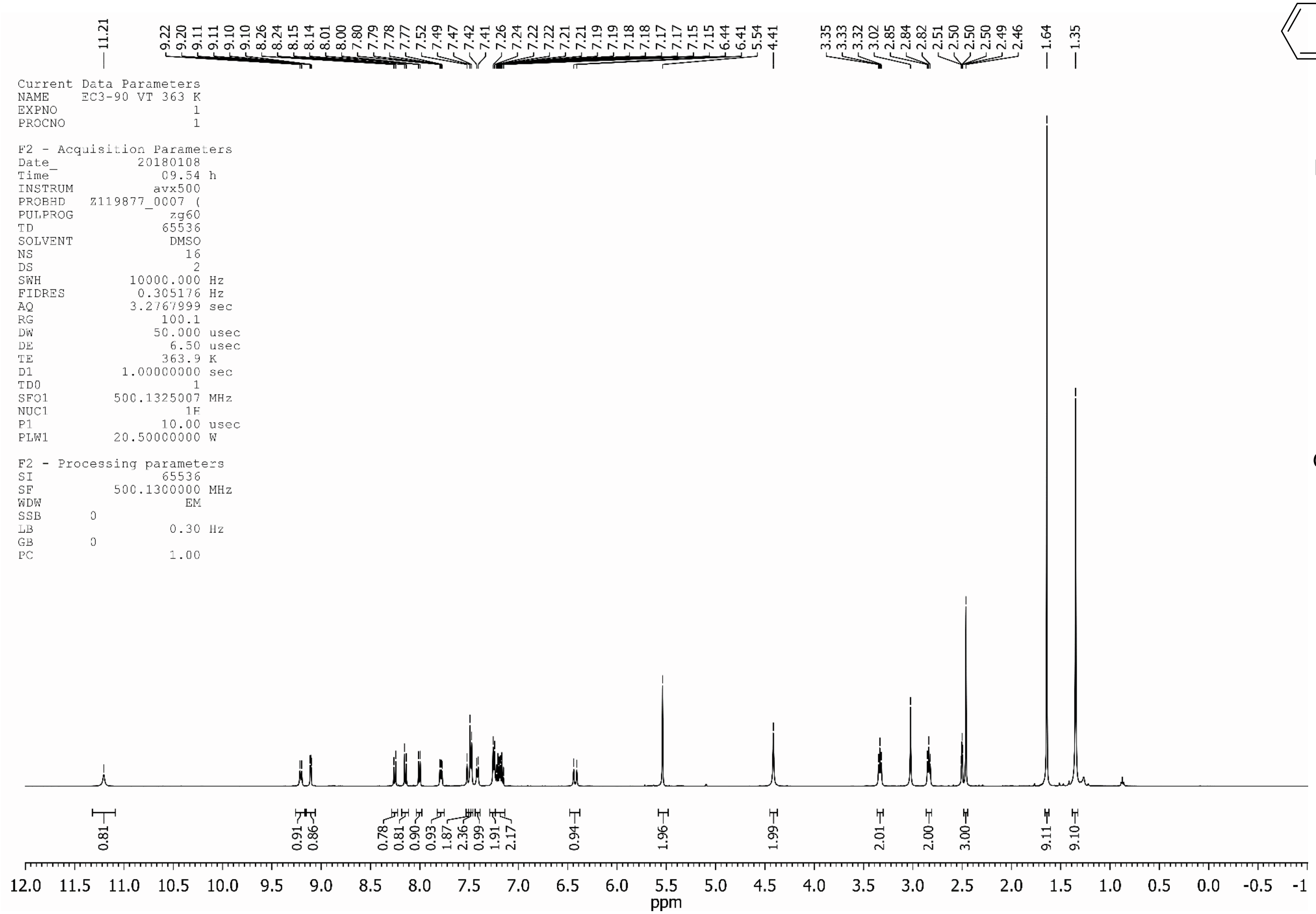

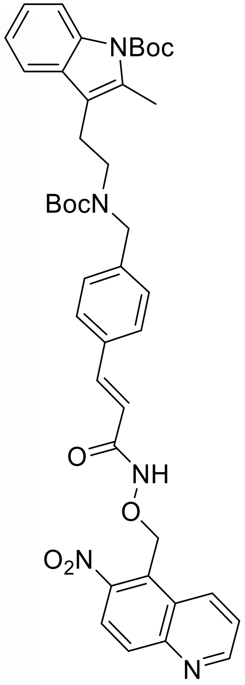

N,N-Di-Boc-*O*-((6-nitroquinolin-5-yl)methyl)-Panobinostat (**9**)

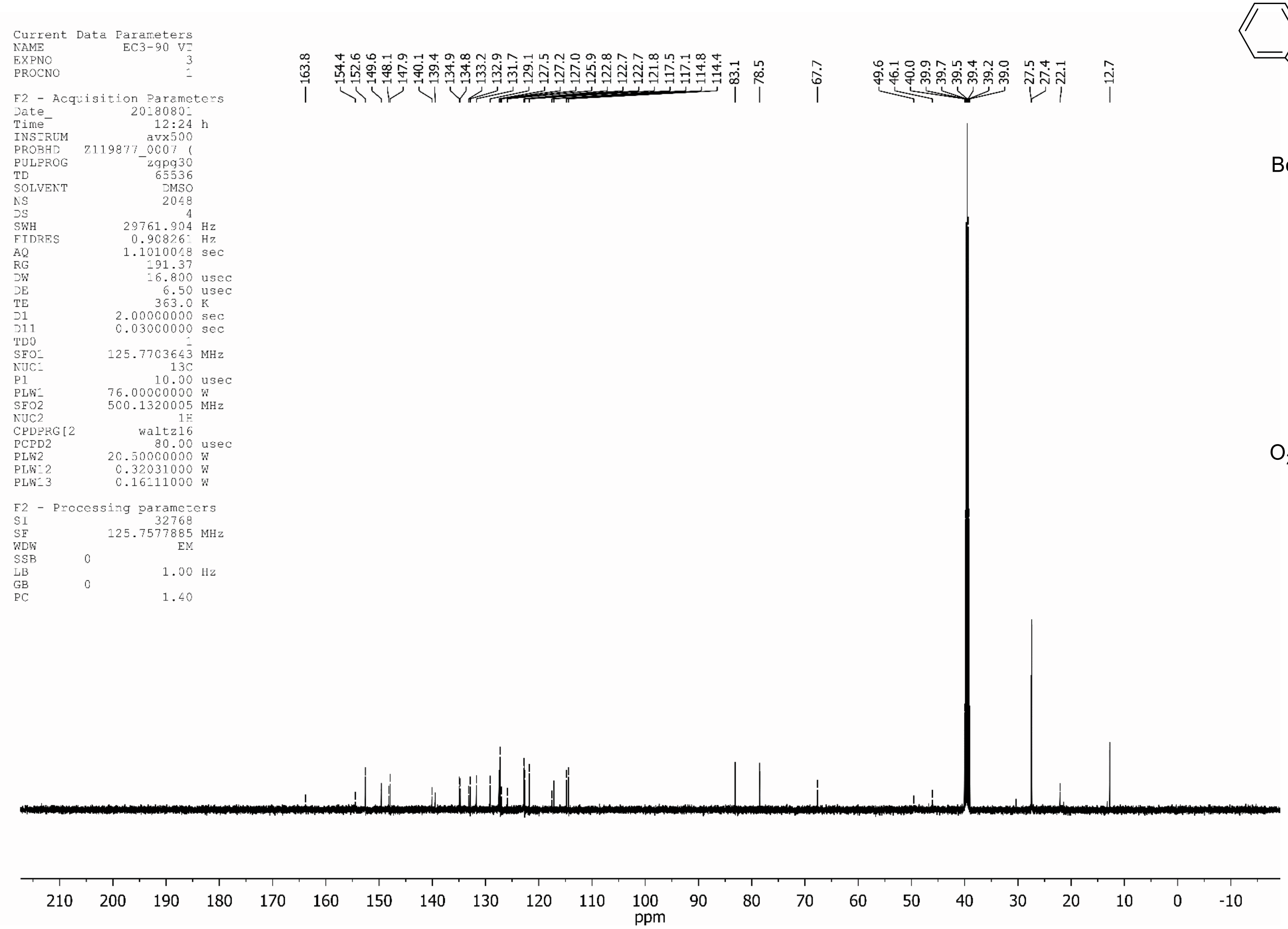

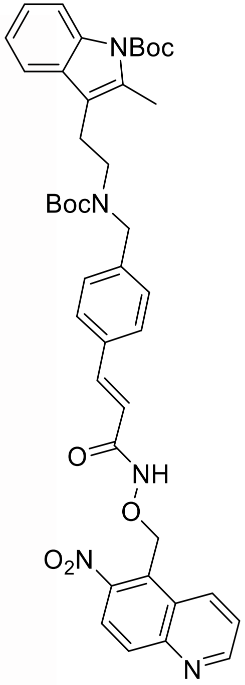

N,N-Di-Boc-*O*-(6-nitroquinolin-5-yl)methyl)-Panobinostat (**9**)

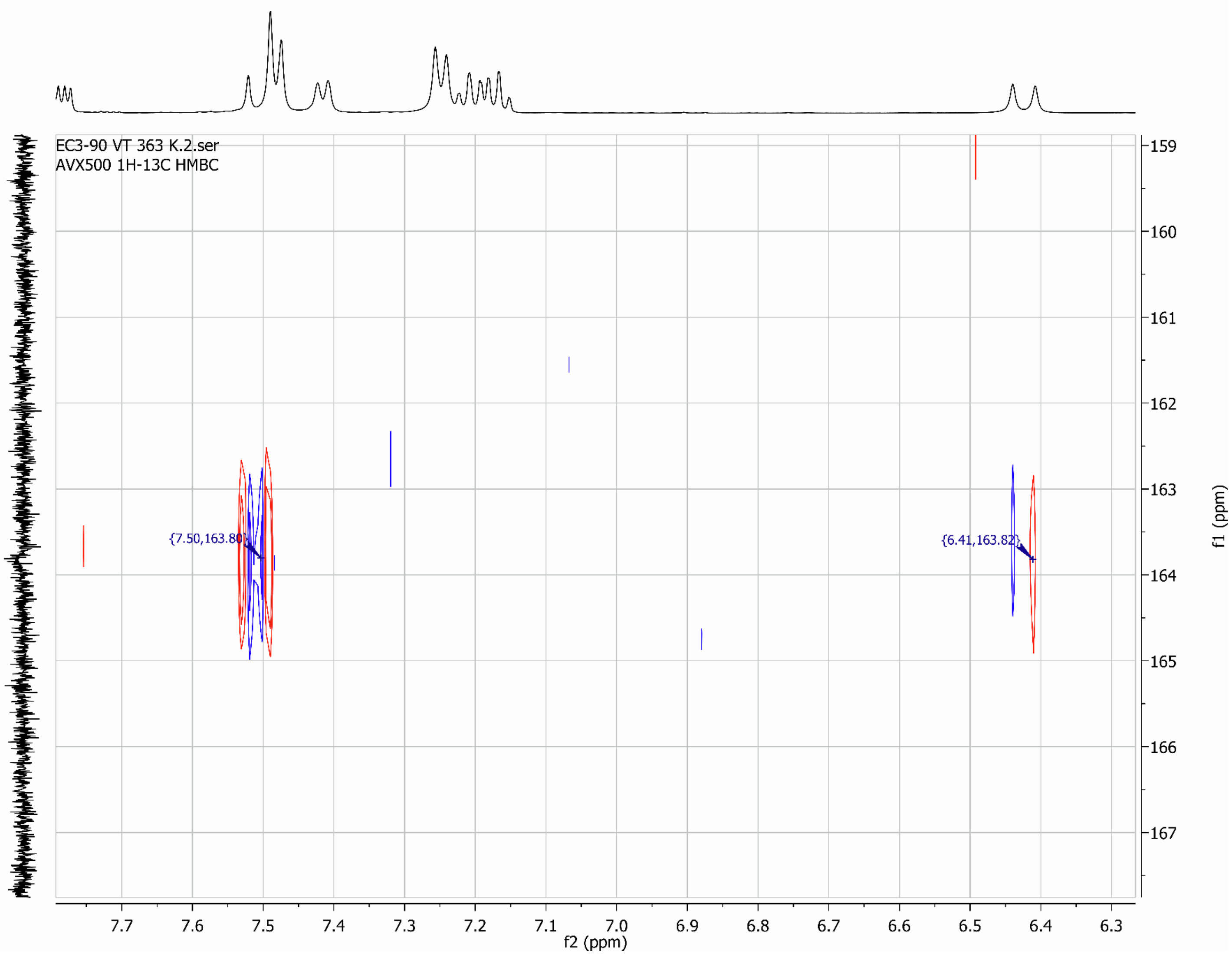

N,N-Di-Boc-*O*-((6-nitroquinolin-5-yl)methyl)-Panobinostat (**9**)

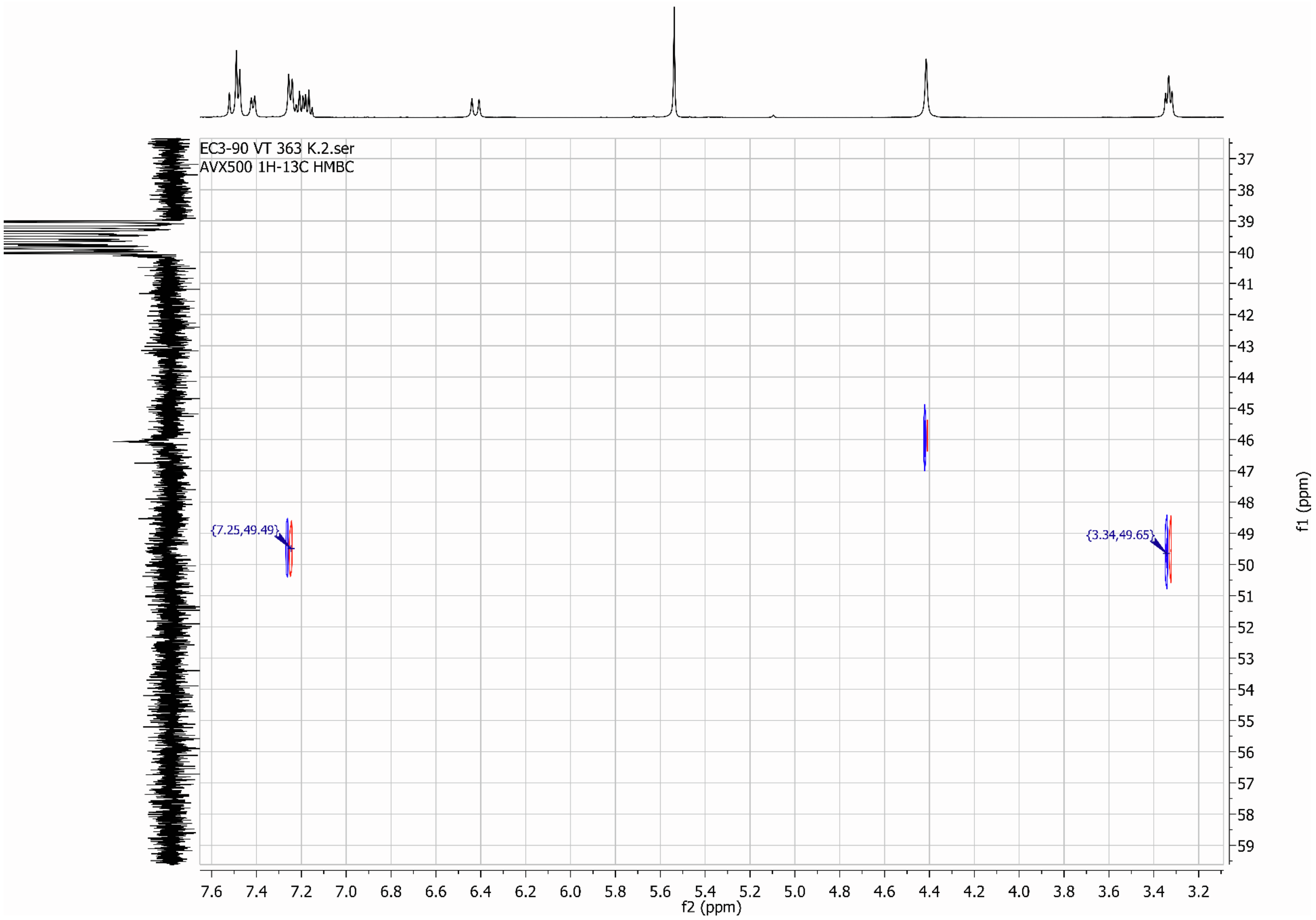

N,N-Di-Boc-O-(1-methyl-2-nitroimidazol-5-yl)methyl)-panobinostat

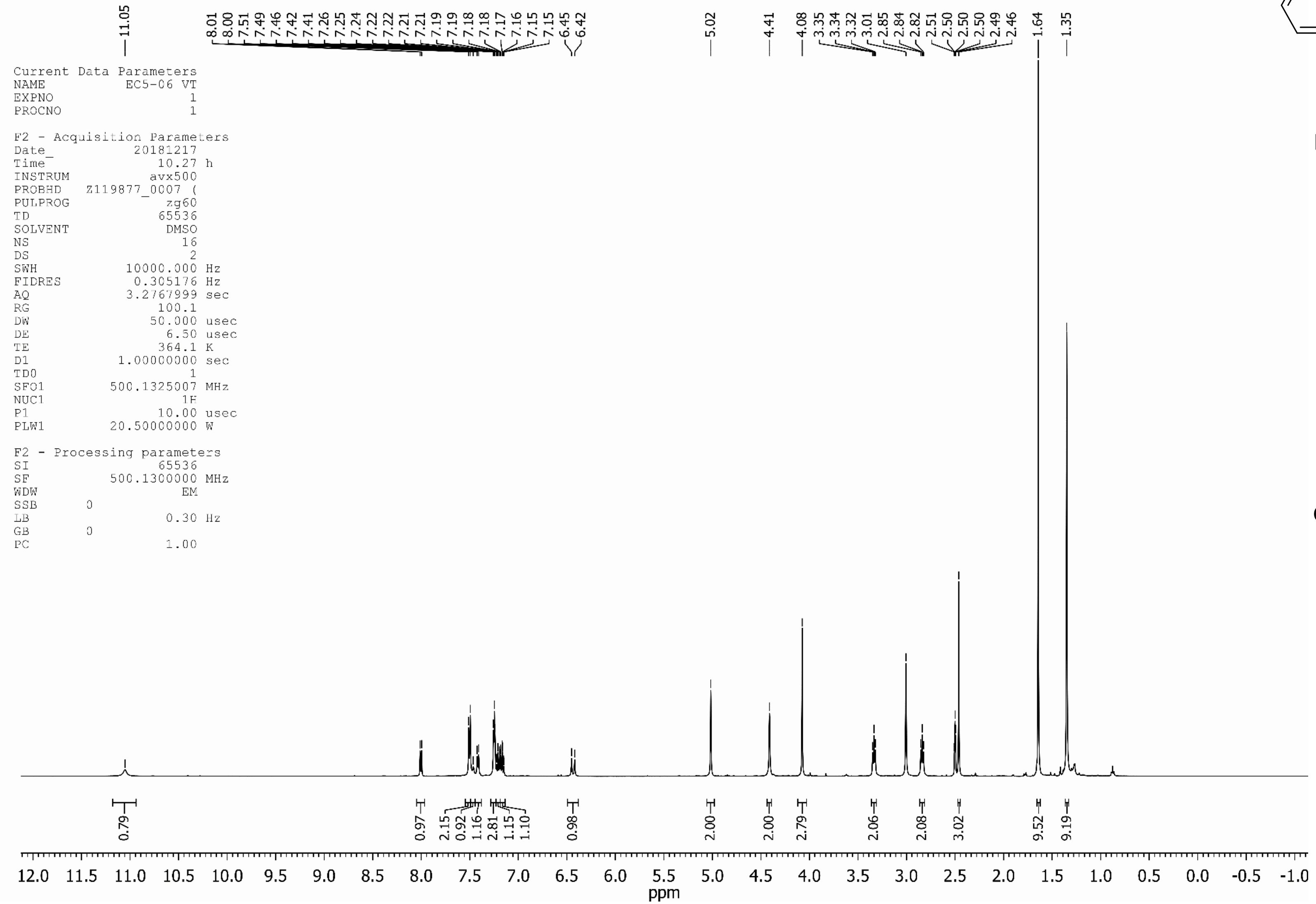

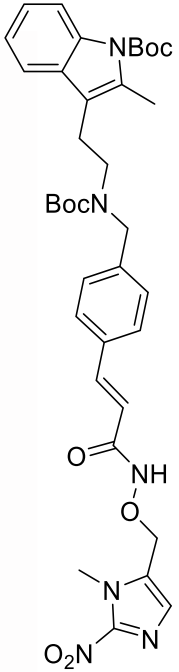

N,N-Di-Boc-*O*-((1-methyl-2-nitroimidazol-5-yl)methyl)-panobinostat

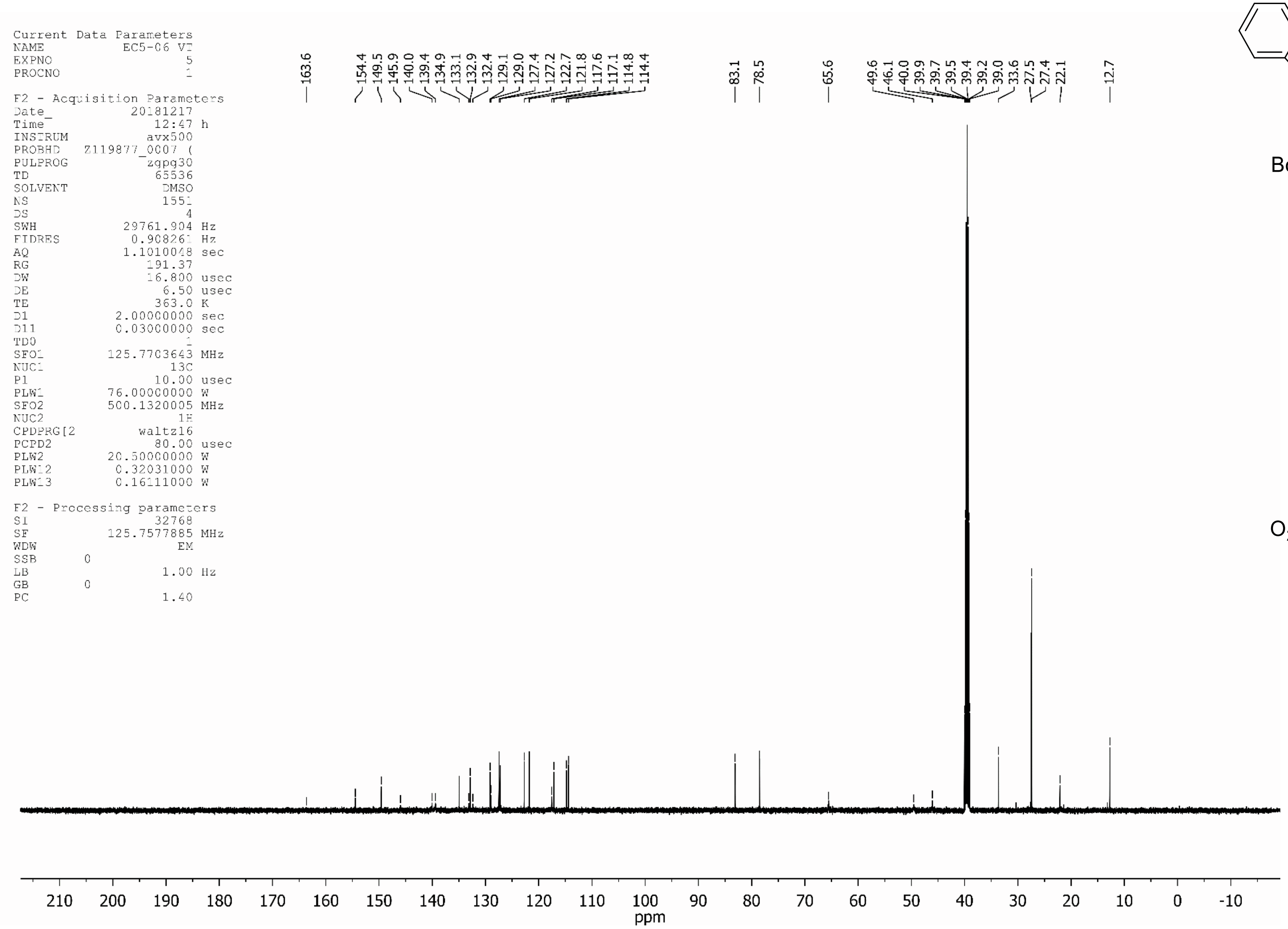

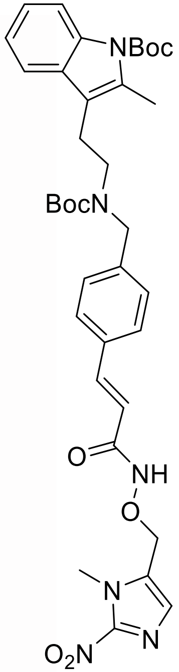

N,N-Di-Boc-*O*-((1-methyl-2-nitroimidazol-5-yl)methyl)-panobinostat

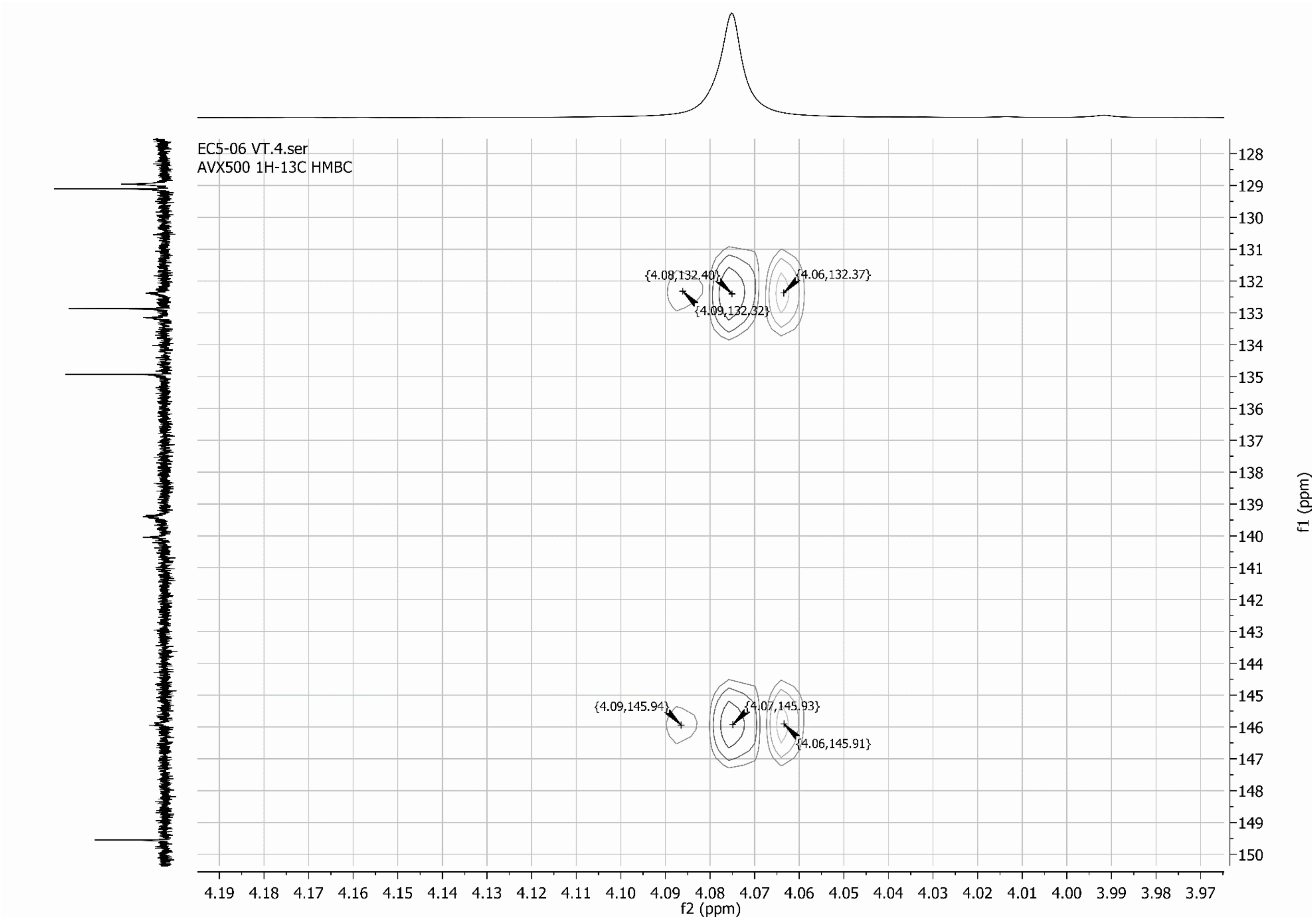

N,N-Di-Boc-*O*-((1-methyl-2-nitroimidazol-5-yl)methyl)-panobinostat

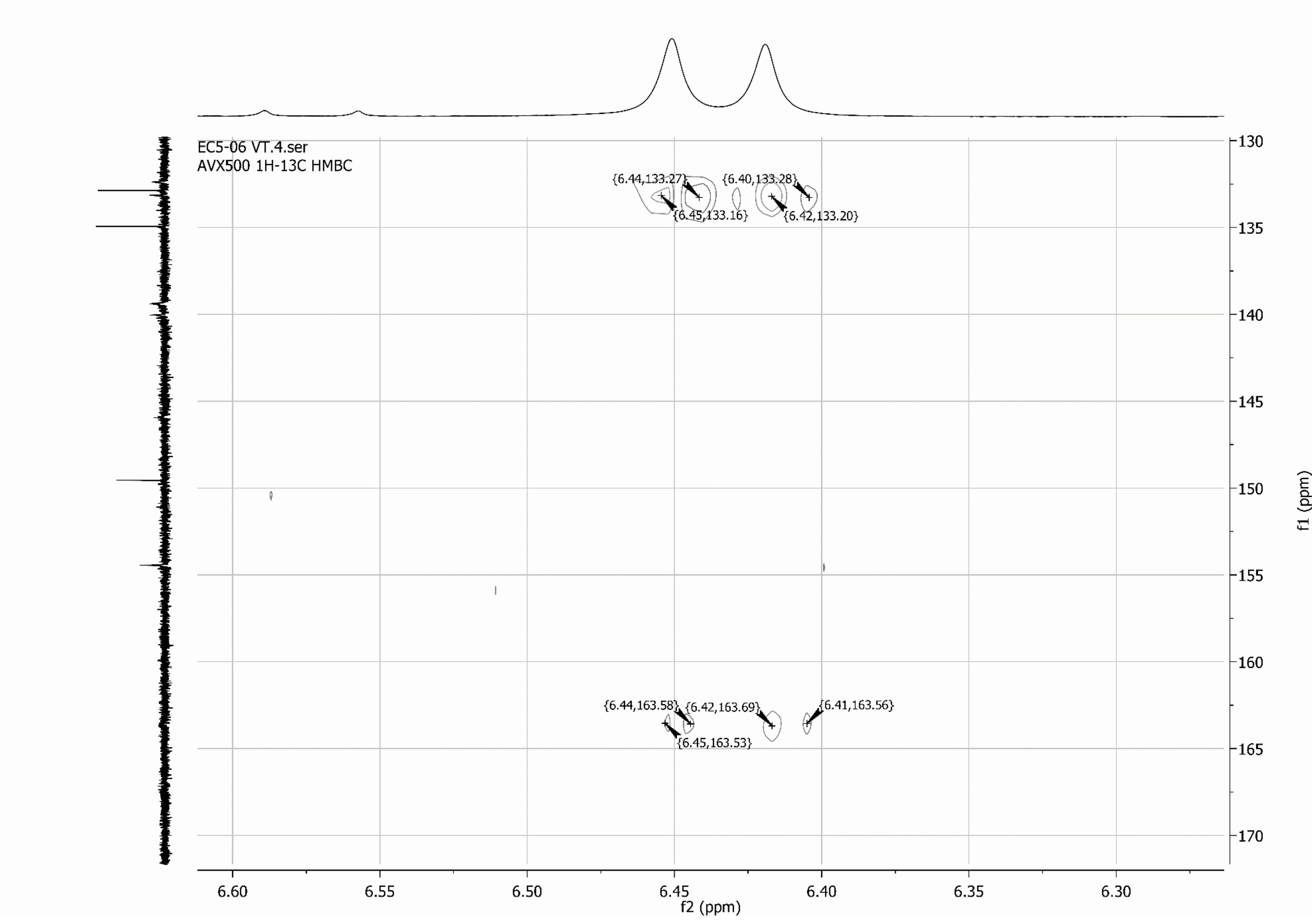

O-Benzyl-Panobinostat (**Bn-Pano**, **10**)

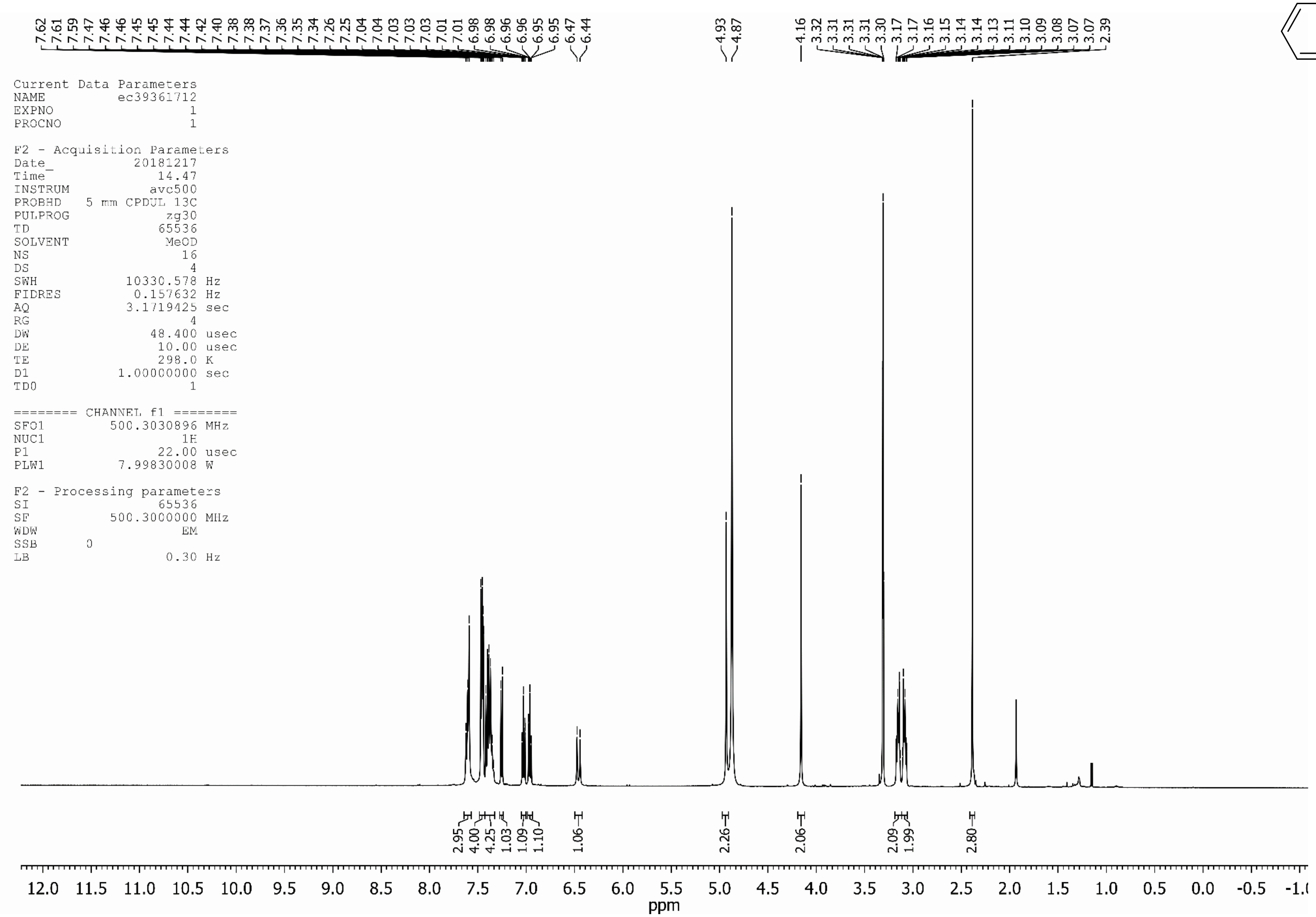

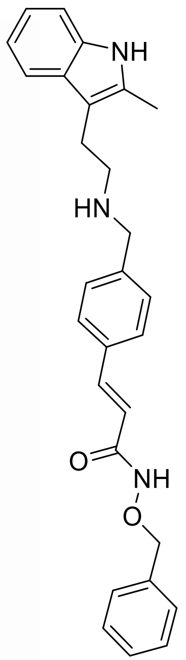

O-Benzyl-Panobinostat (**Bn-Pano, 10**)

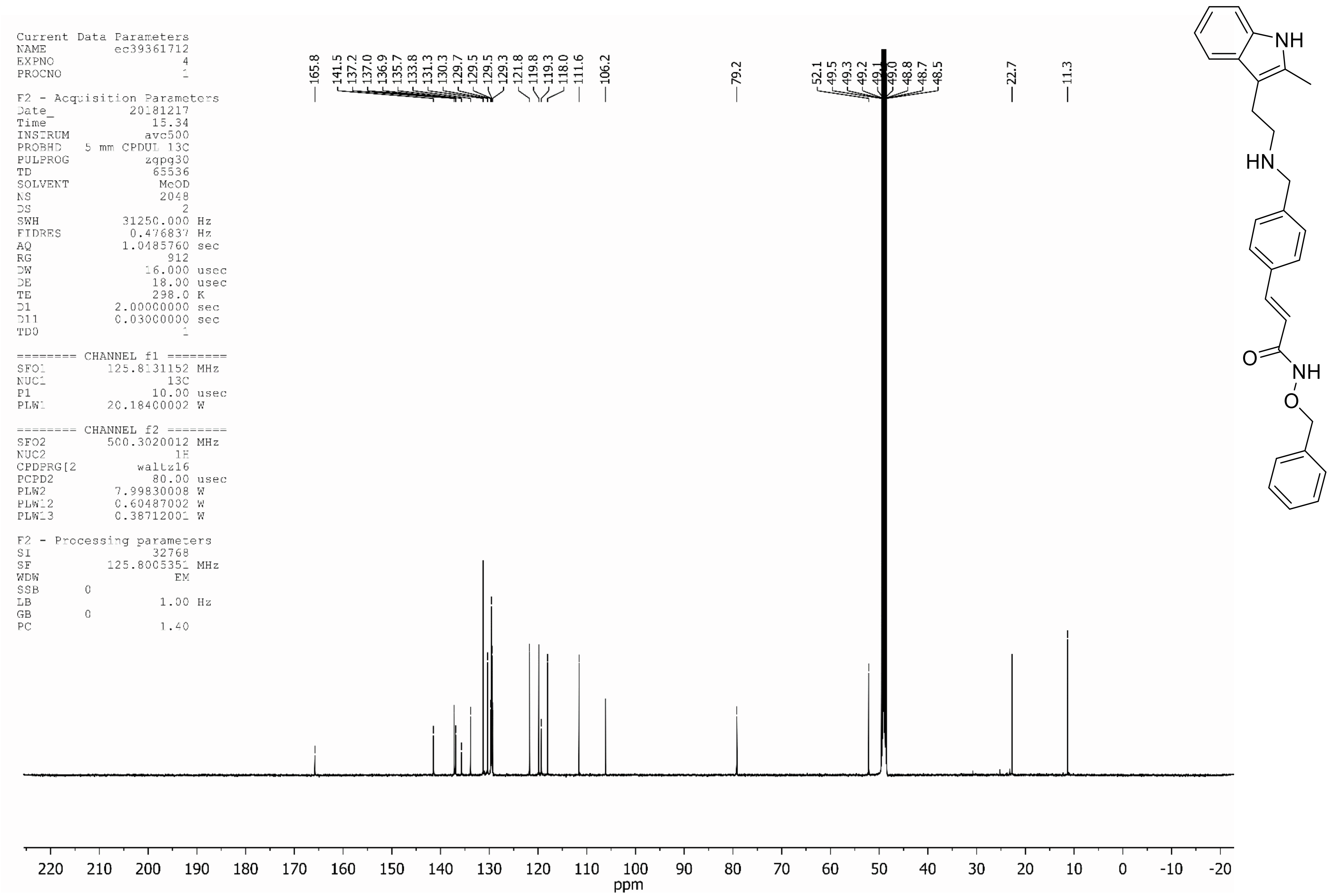

O-Benzyl-Panobinostat (**Bn-Pano, 10**)

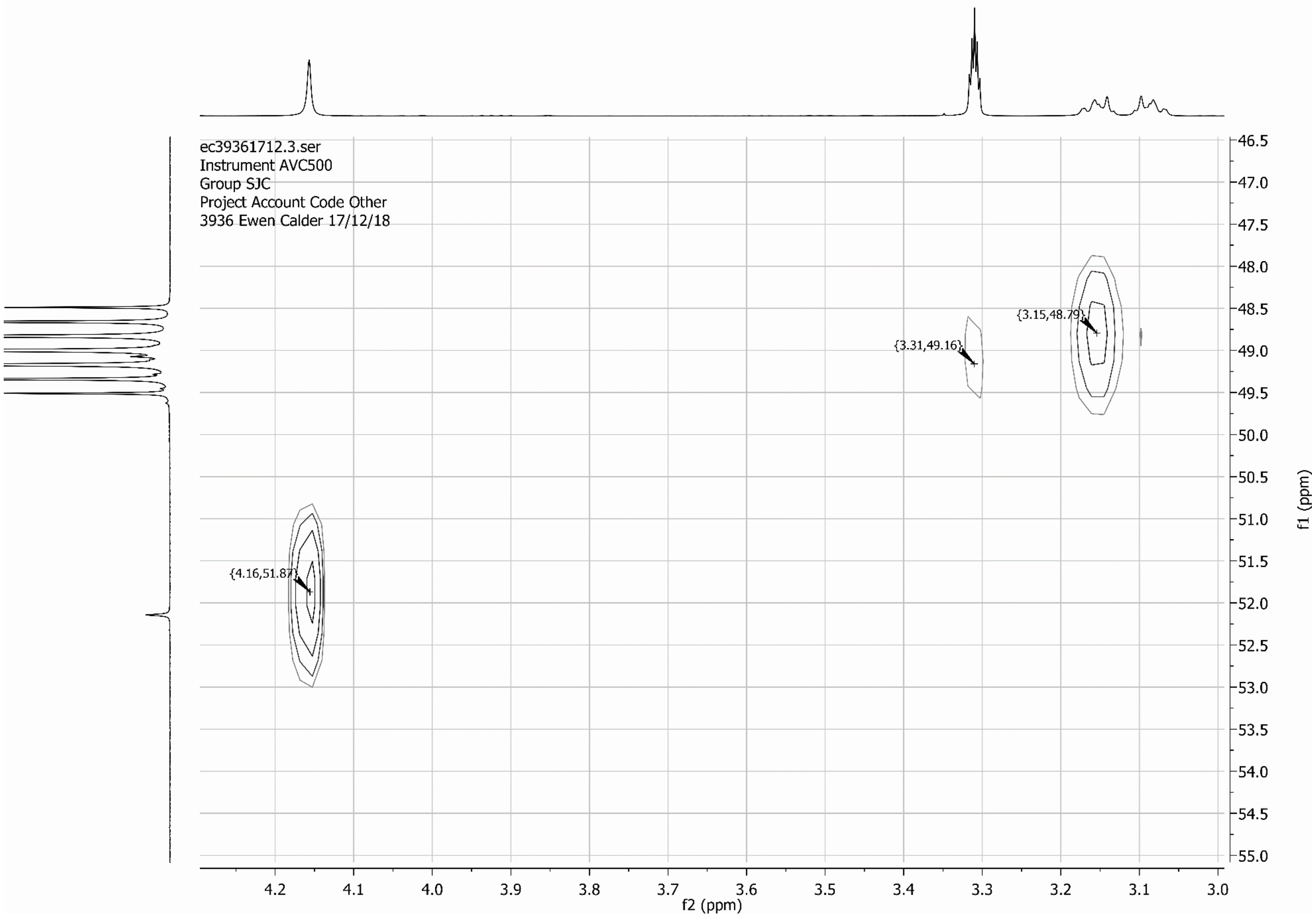

O-(4-Nitrobenzyl)-panobinostat trifluoroacetate (NB-Pano, **11**)

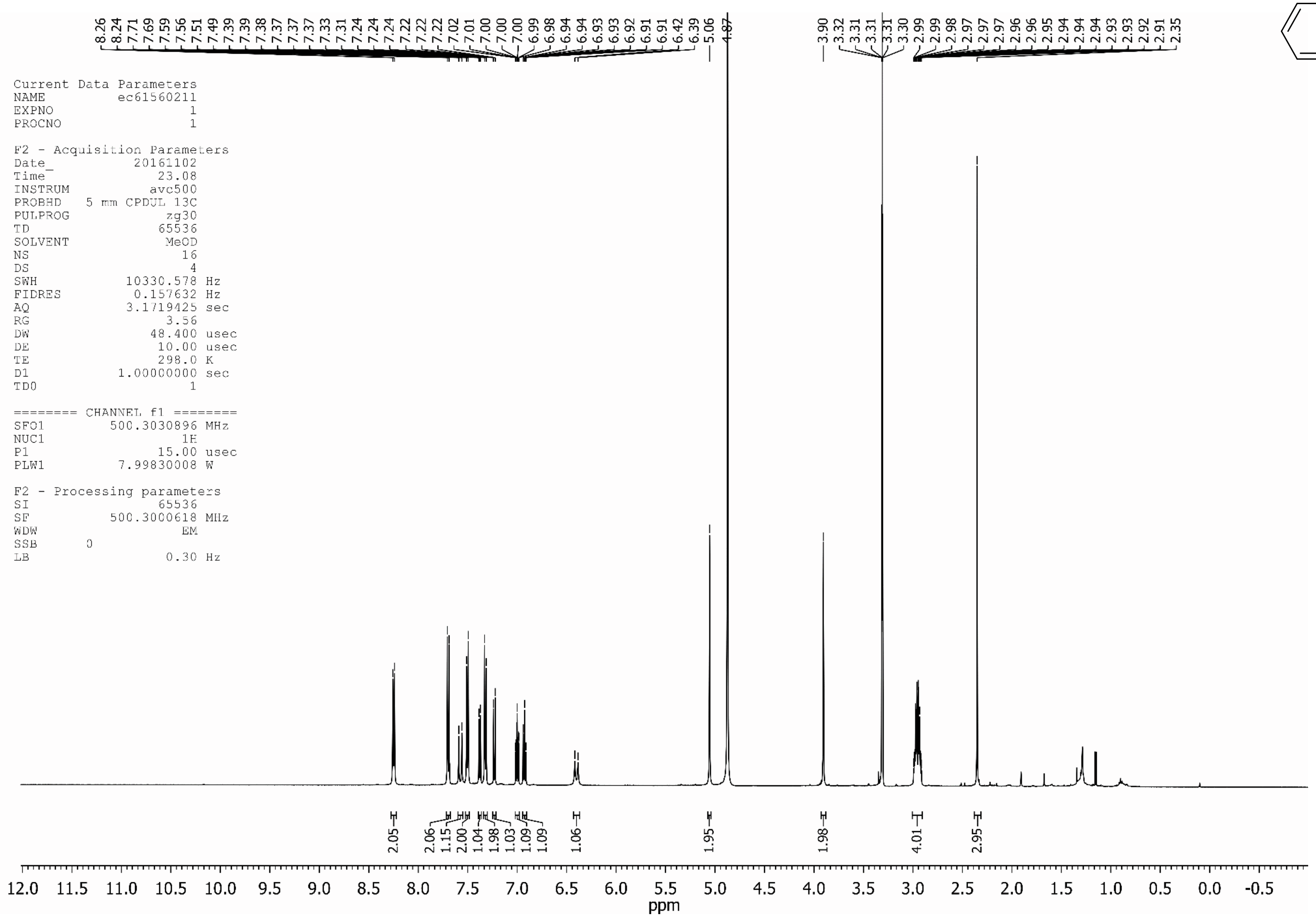

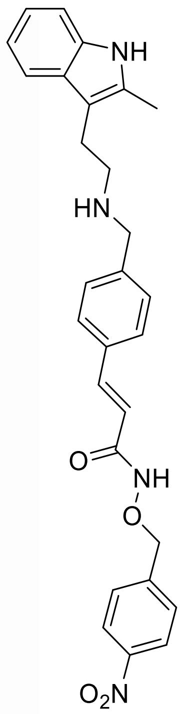

O-(4-Nitrobenzyl)-panobinostat trifluoroacetate (NB-Pano, **11**)

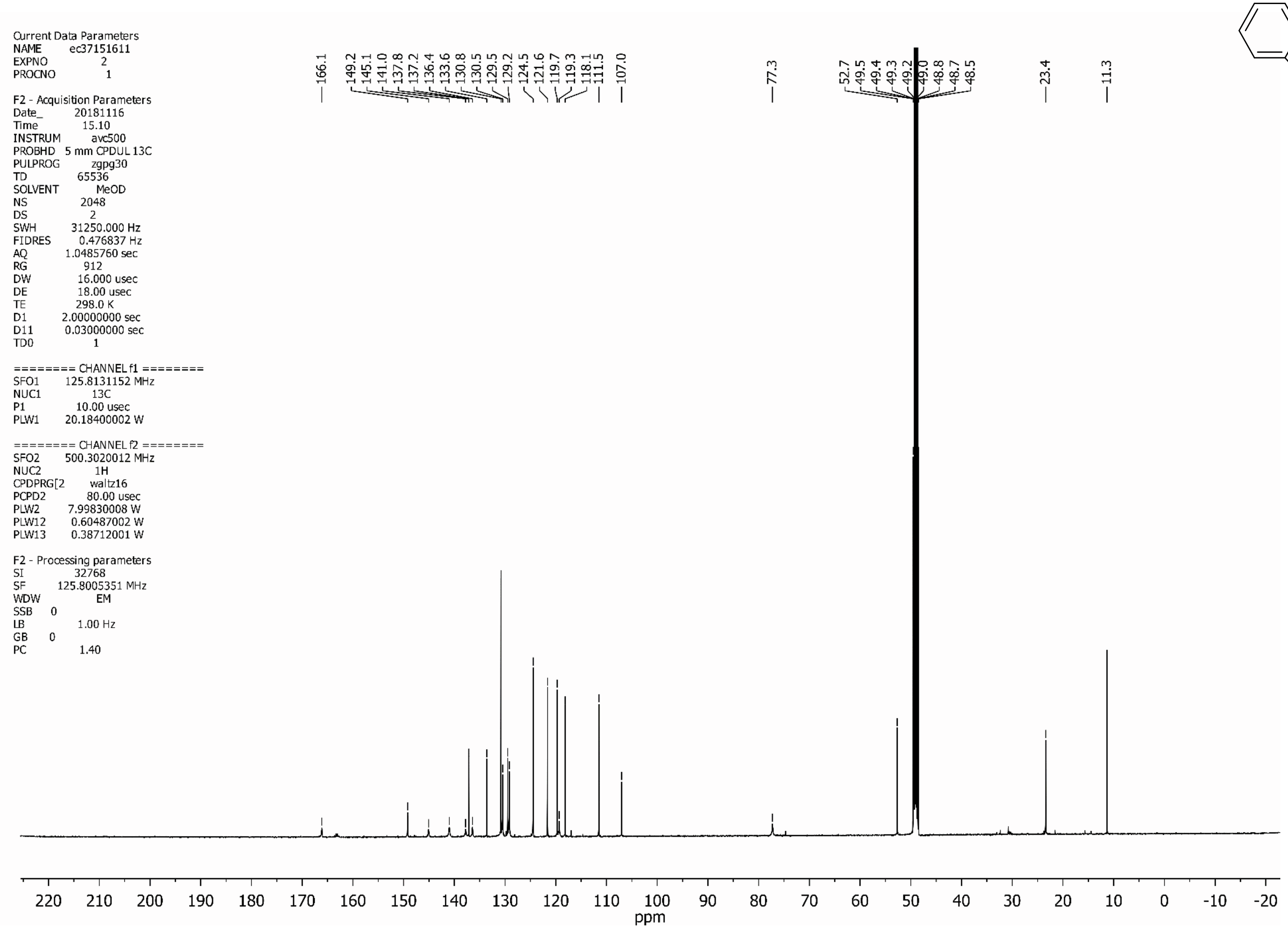

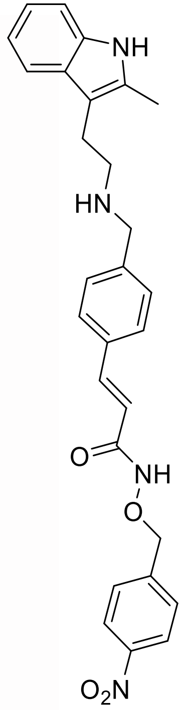

O-(5-Nitrothiophen-2-yl)methyl))-panobinostat trifluoroacetate (NT-Pano, **12**)

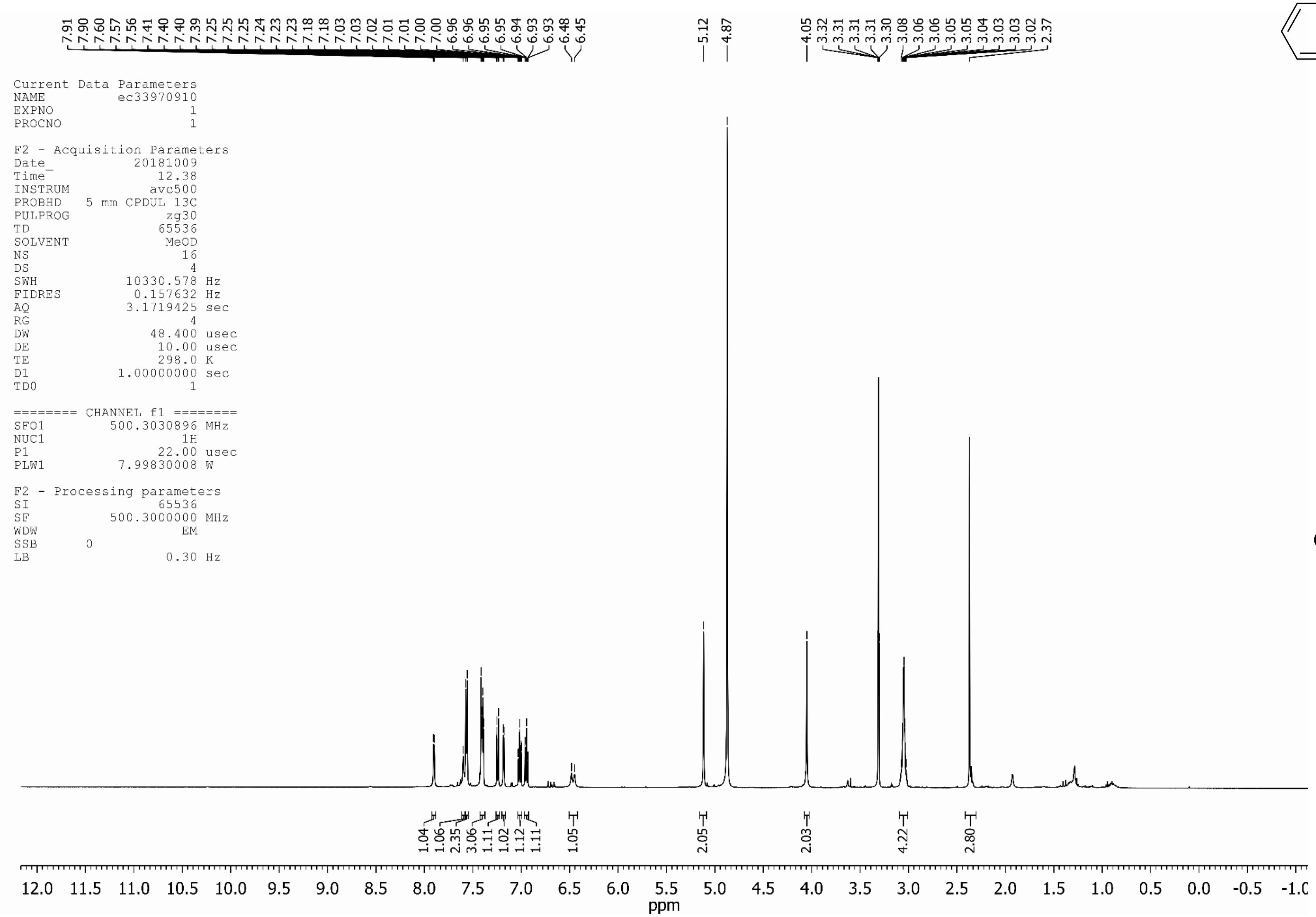

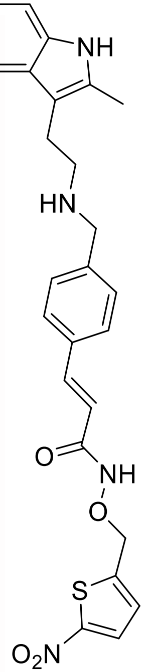

O-(5-Nitrothiophen-2-yl)methyl))-panobinostat trifluoroacetate (NT-Pano, **12**)

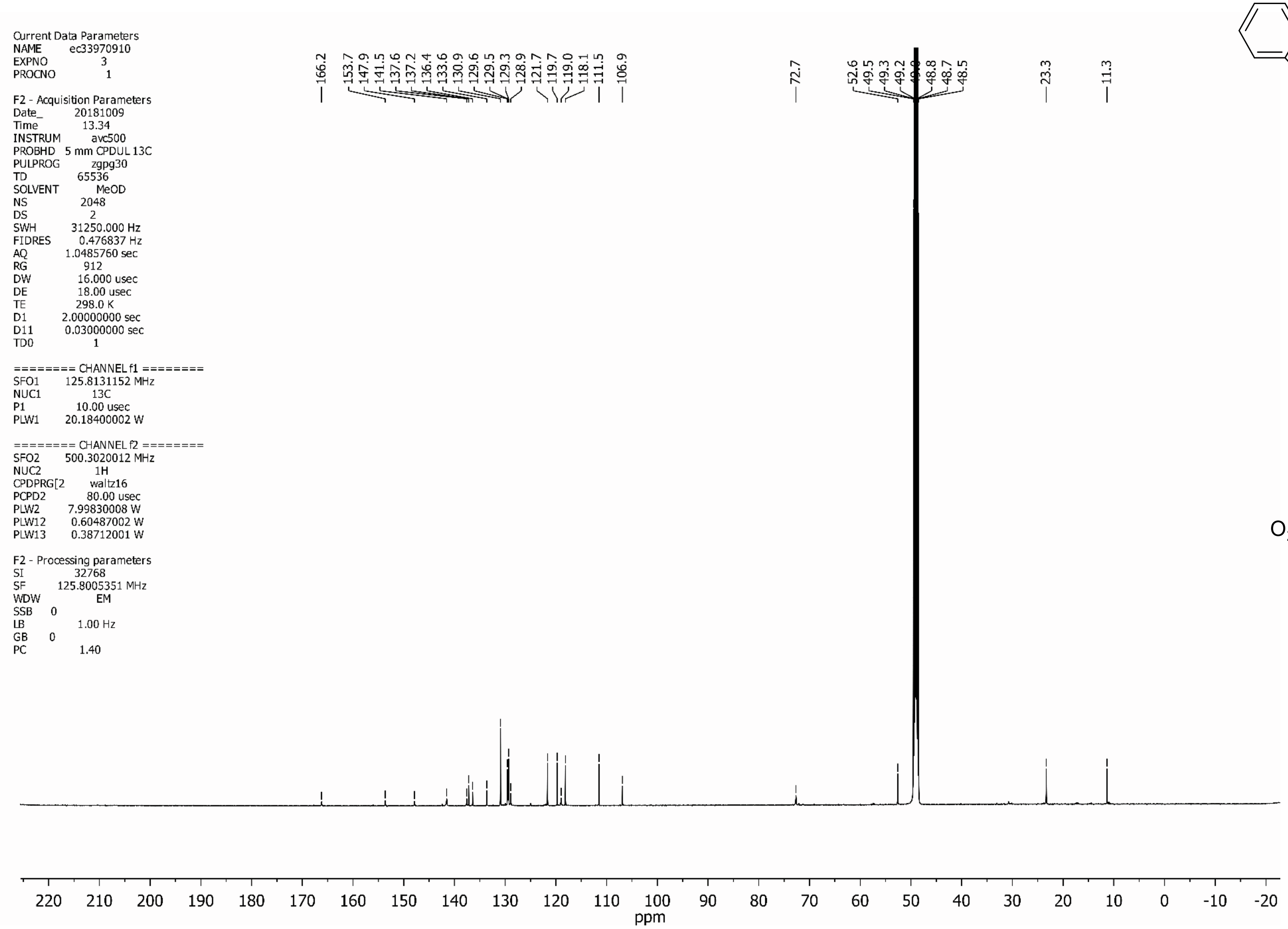

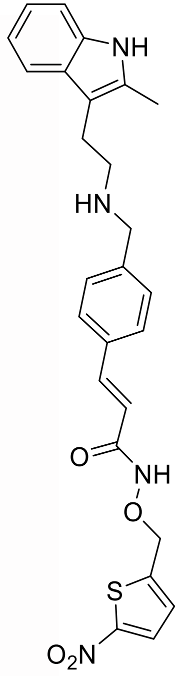

O-(5-Nitrothiophen-2-yl)methyl))-panobinostat trifluoroacetate (NT-Pano, **12**)

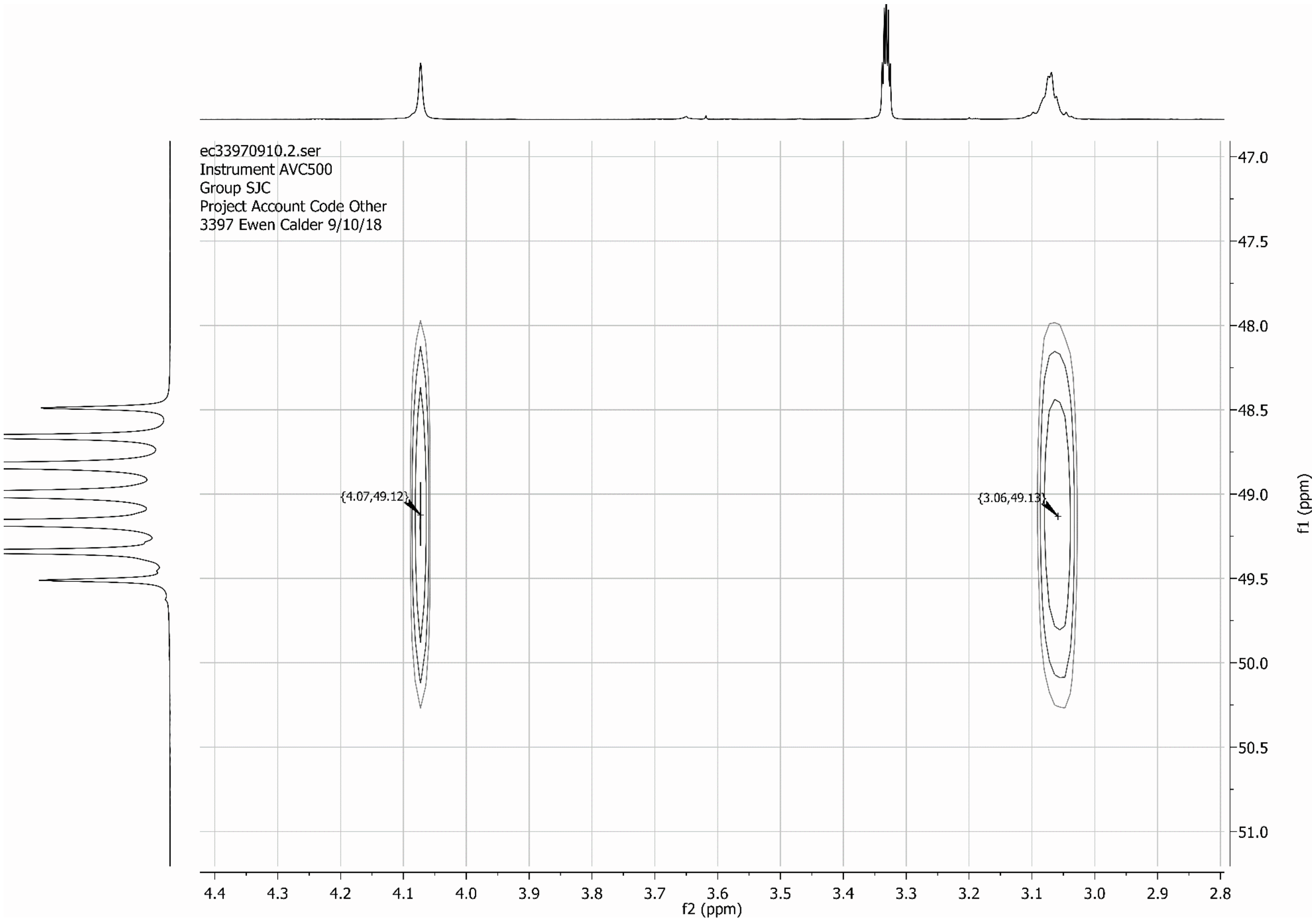

O-(6-Nitroquinolin-5-yl)methyl))-panobinostat di-trifluoroacetate (NQ-Pano, **13**)

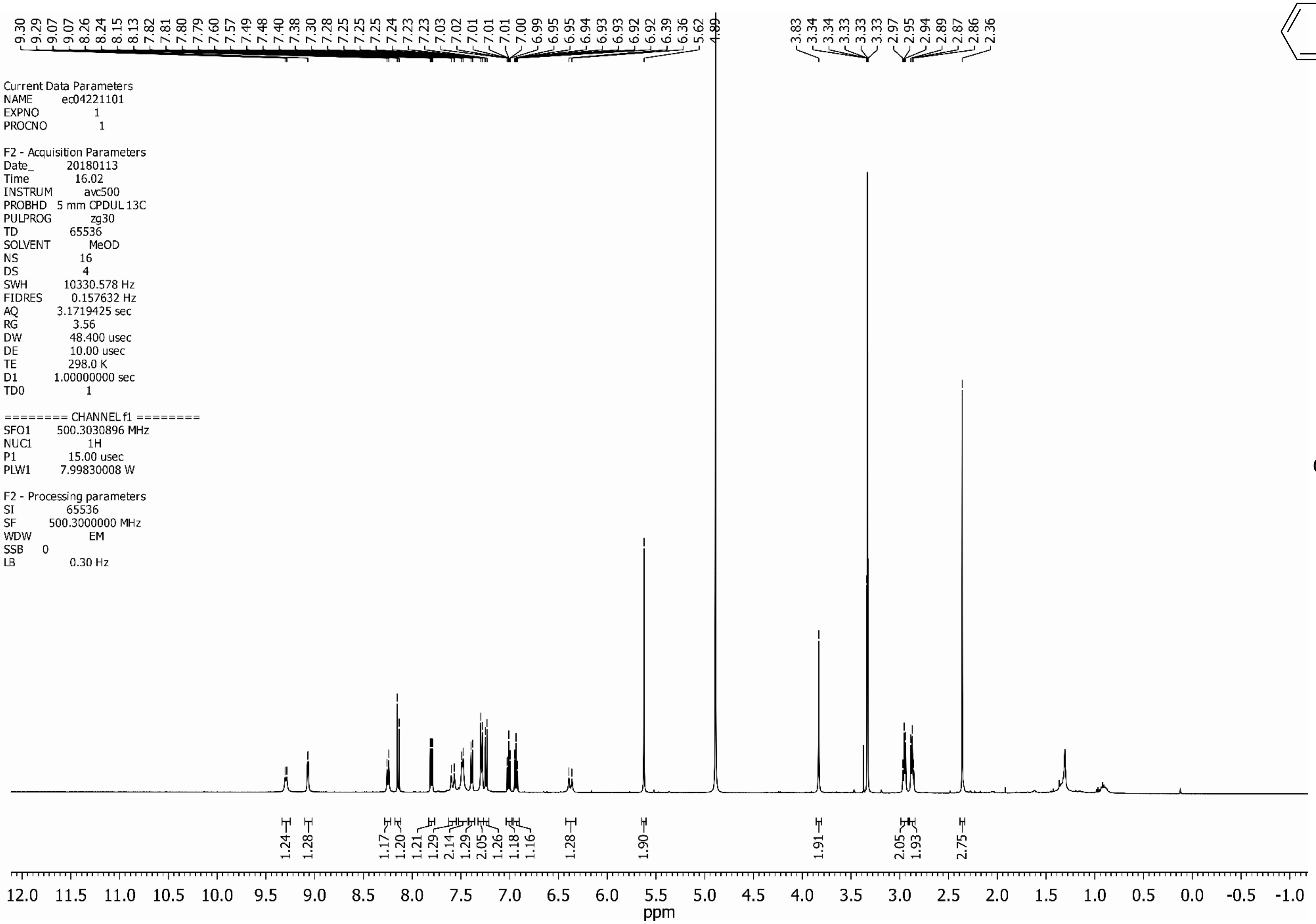

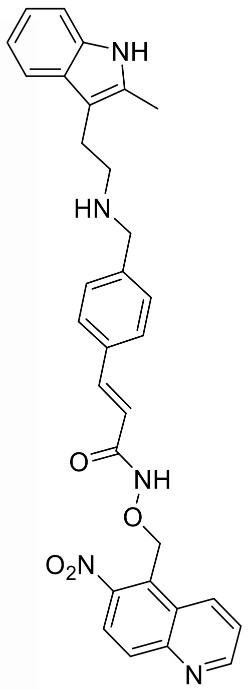

O-(6-Nitroquinolin-5-yl)methyl))-panobinostat di-trifluoroacetate (NQ-Pano, **13**)

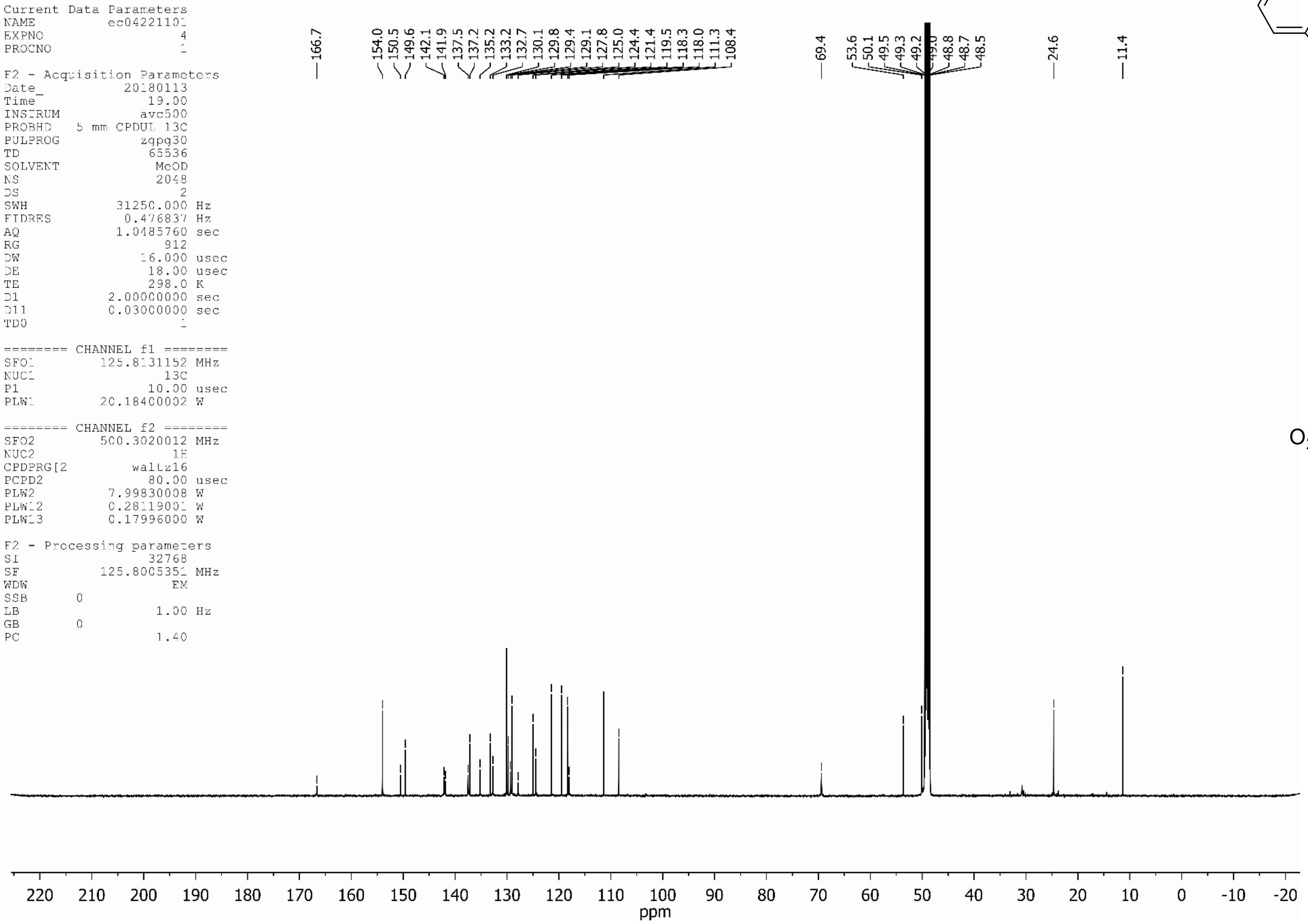

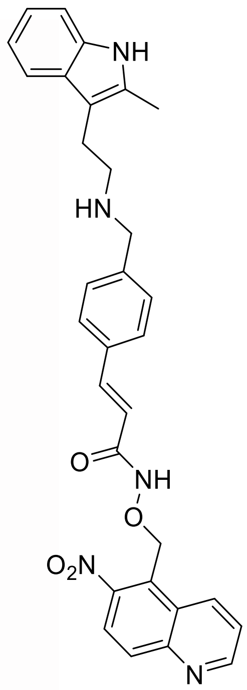

N,N-Di-Boc-Panobinostat (***14***)

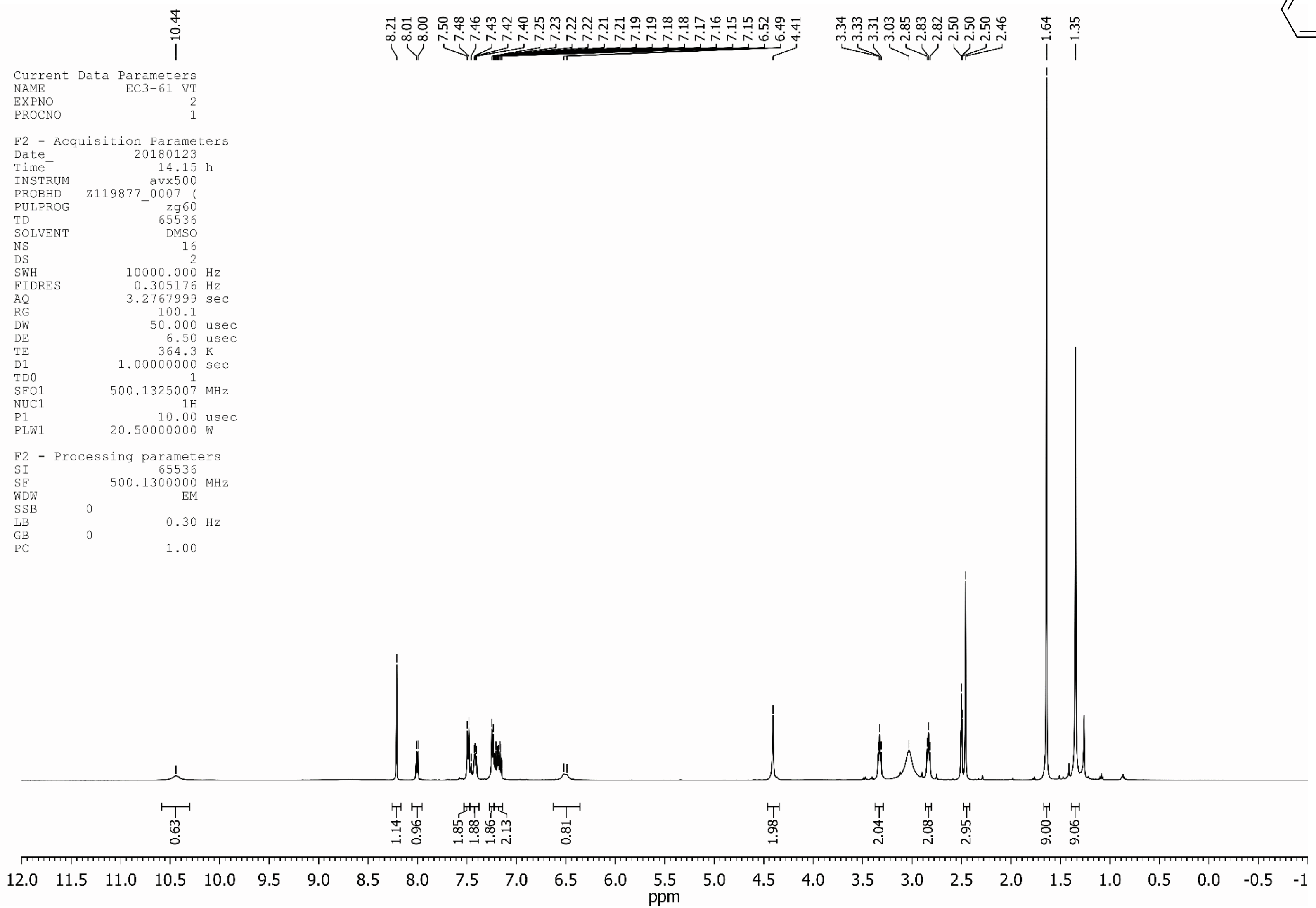

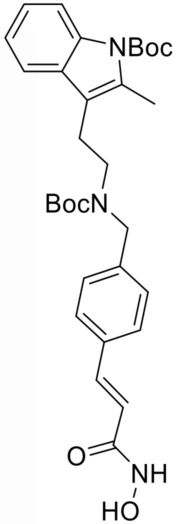

N,N-Di-Boc-Panobinostat (**14**)

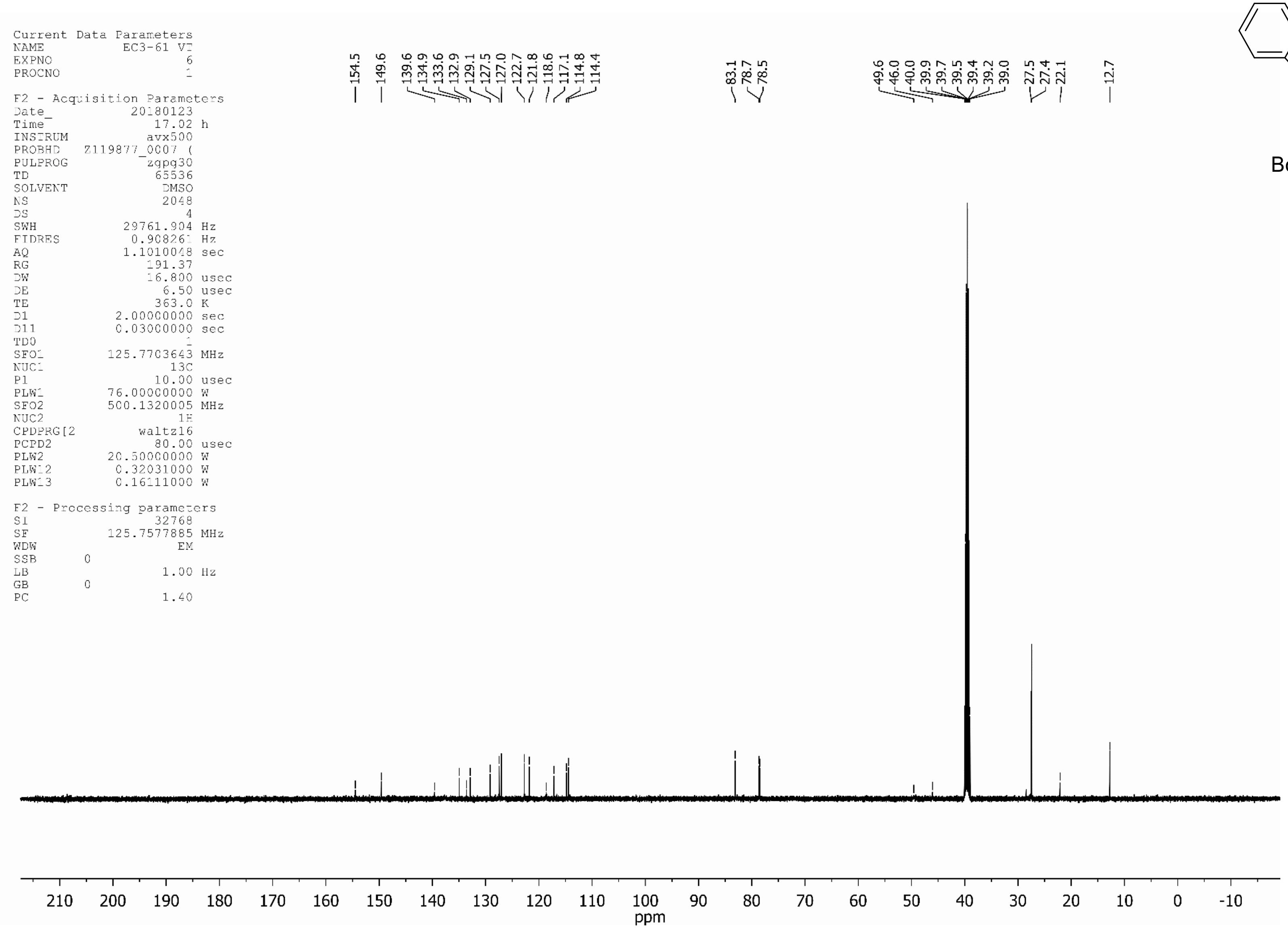

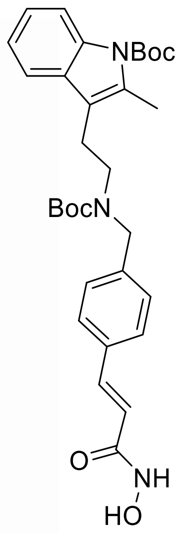

N,N-Di-Boc-Panobinostat (**14**)

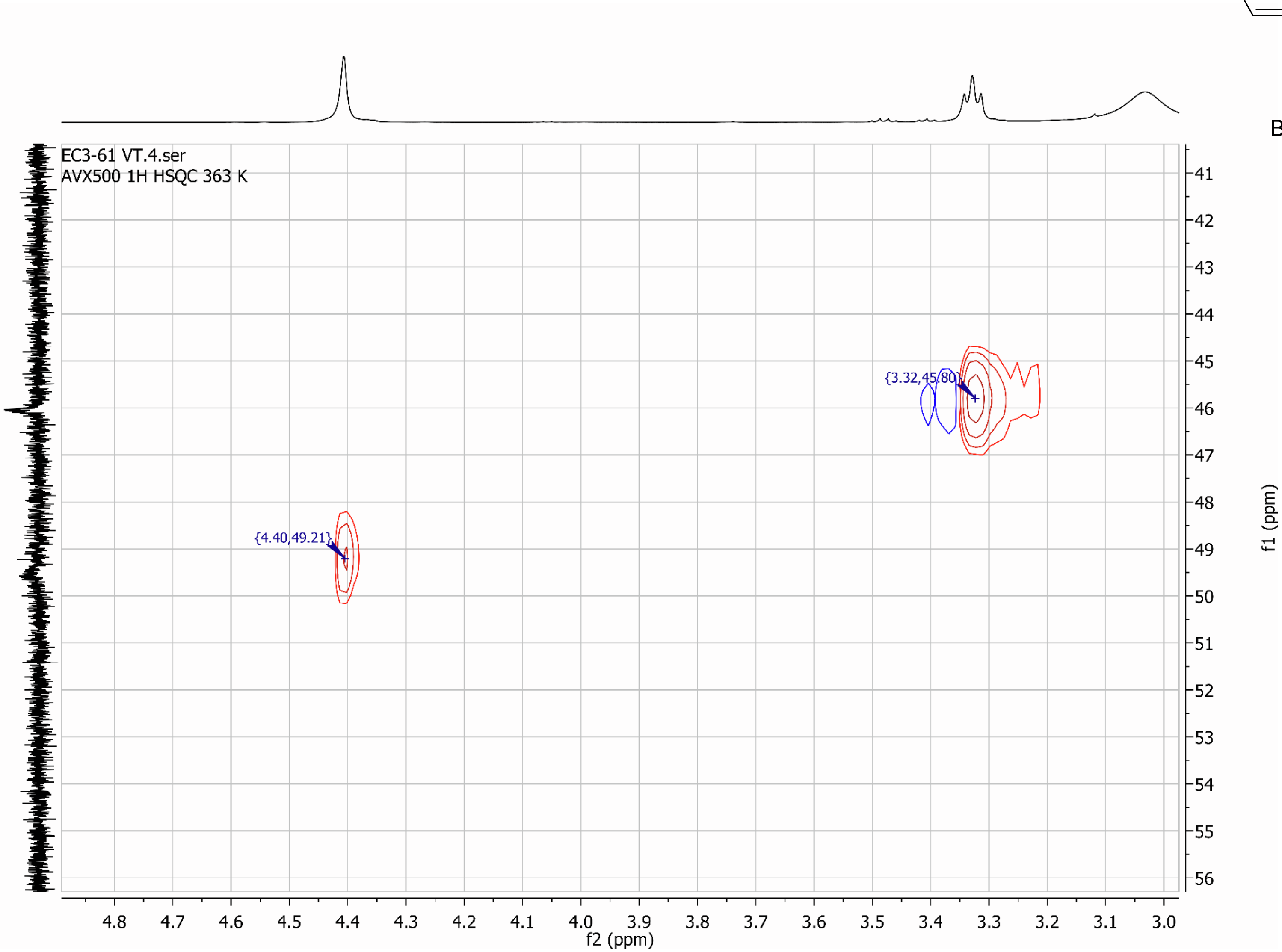

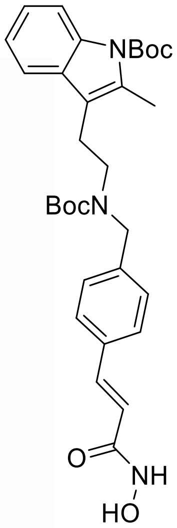

O-((1-Methyl-2-nitroimidazol-5-yl)methyl)-Panobinostat di-trifluoroacetate (CH-03, NI-Pano, **1**)

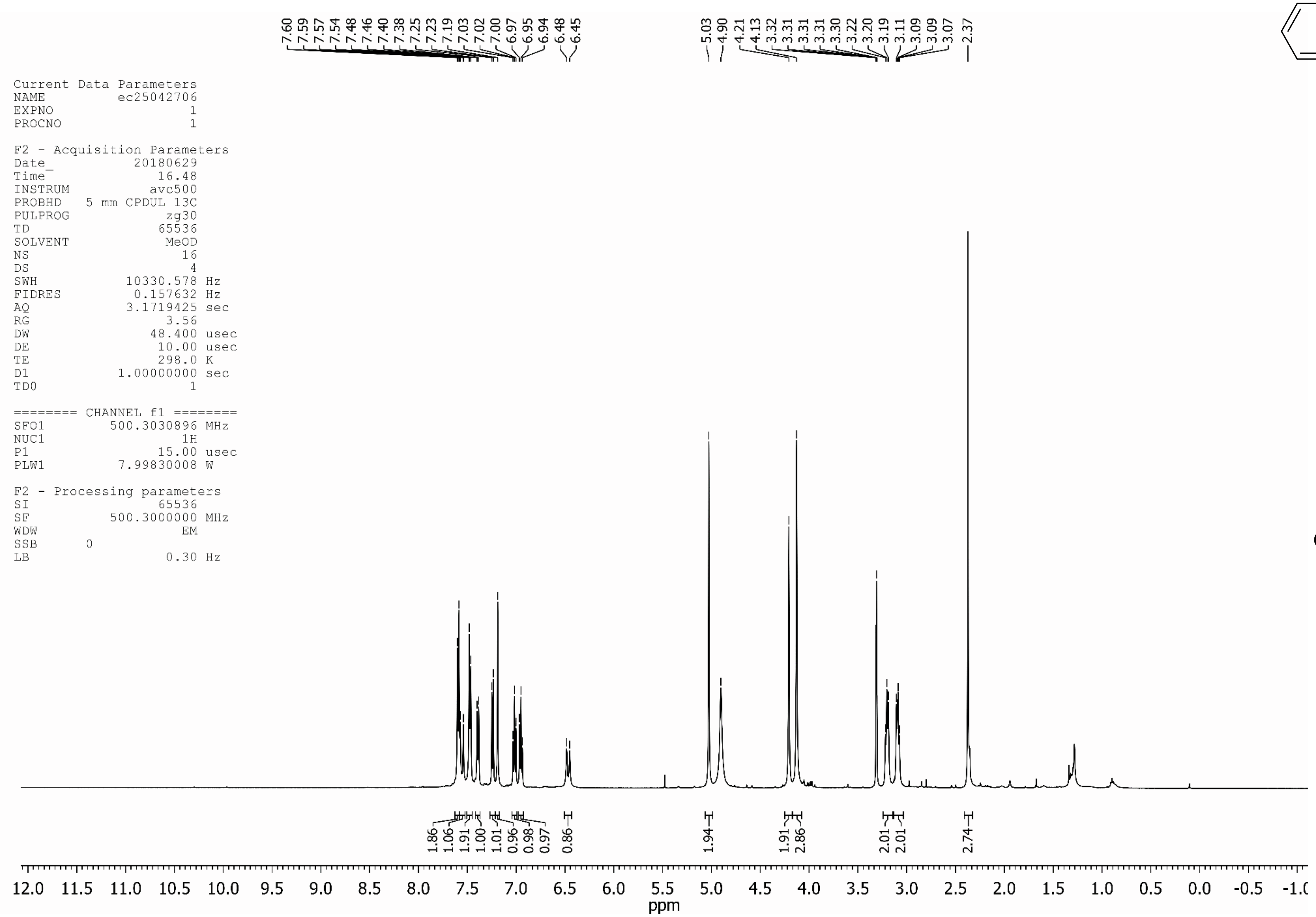

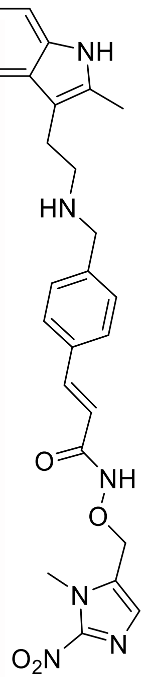

O-((1-Methyl-2-nitroimidazol-5-yl)methyl)-Panobinostat di-trifluoroacetate (CH-03, NI-Pano, **1**)

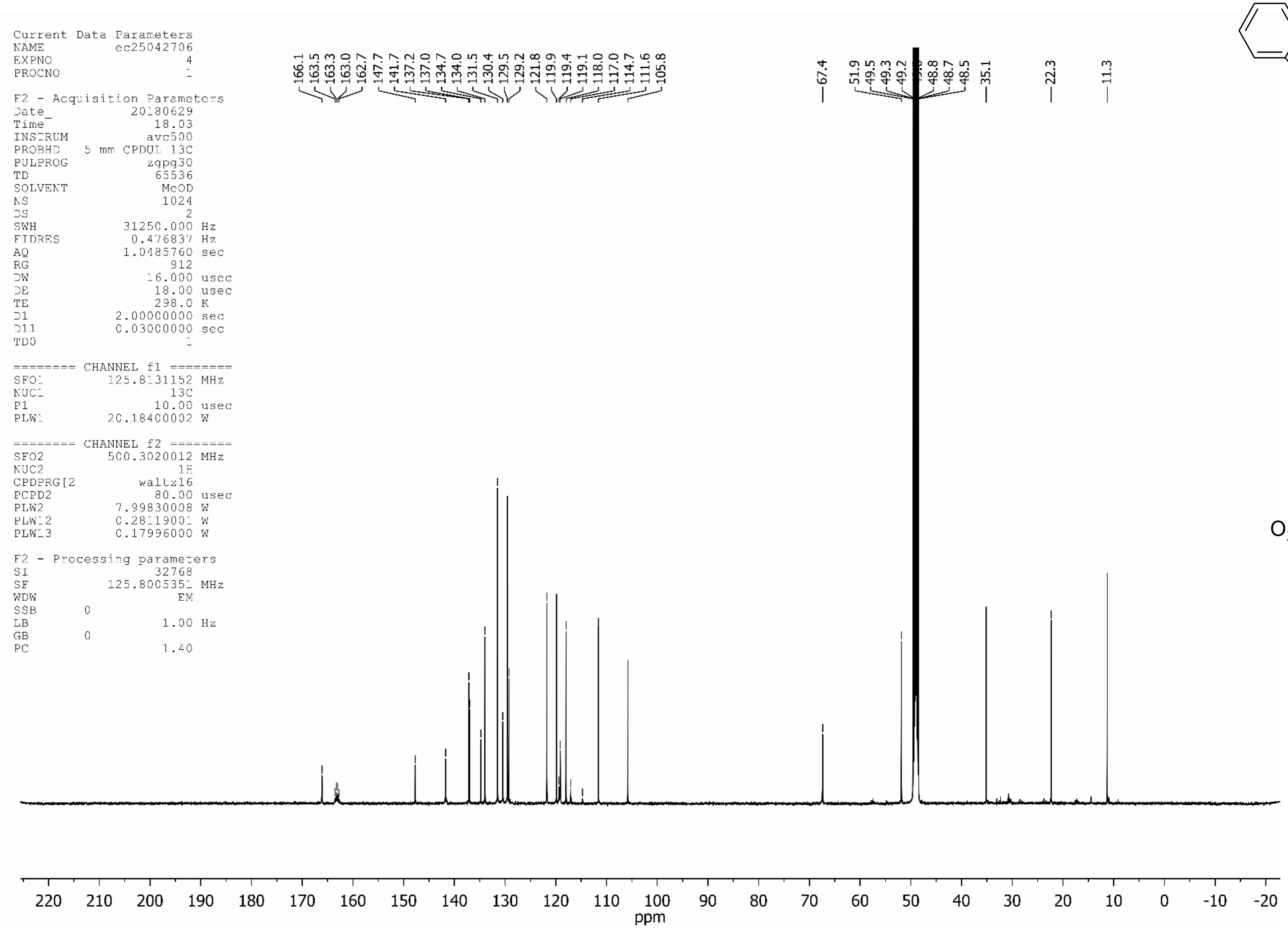

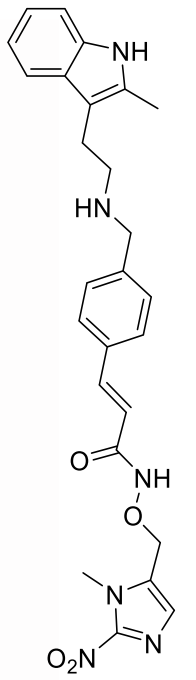

O-((1-Methyl-2-nitroimidazol-5-yl)methyl)-Panobinostat di-trifluoroacetate (CH-03, NI-Pano, **1**)

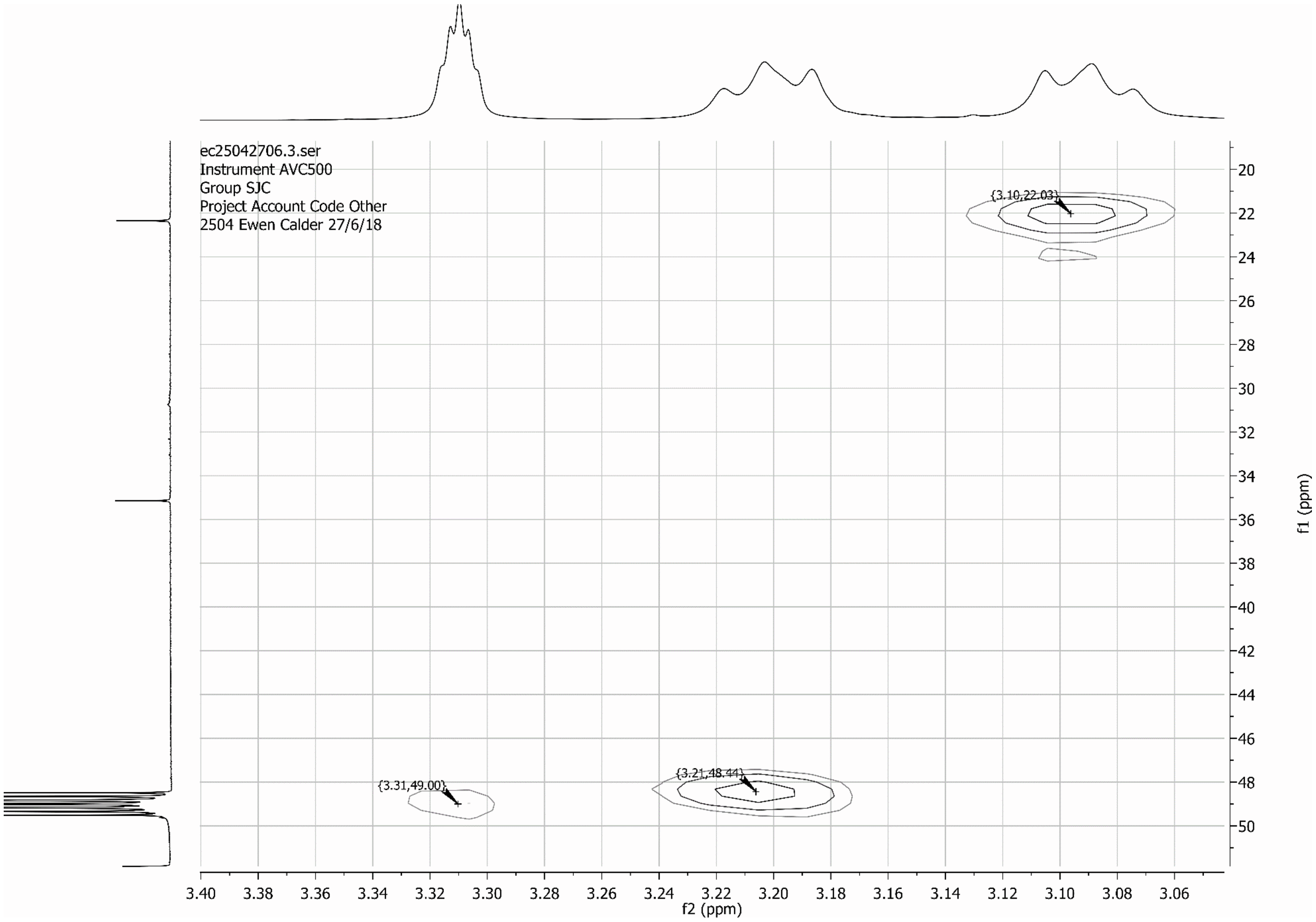

O-((1-Methyl-2-nitroimidazol-5-yl)methyl)-Panobinostat di-trifluoroacetate (CH-03, NI-Pano, **1**)

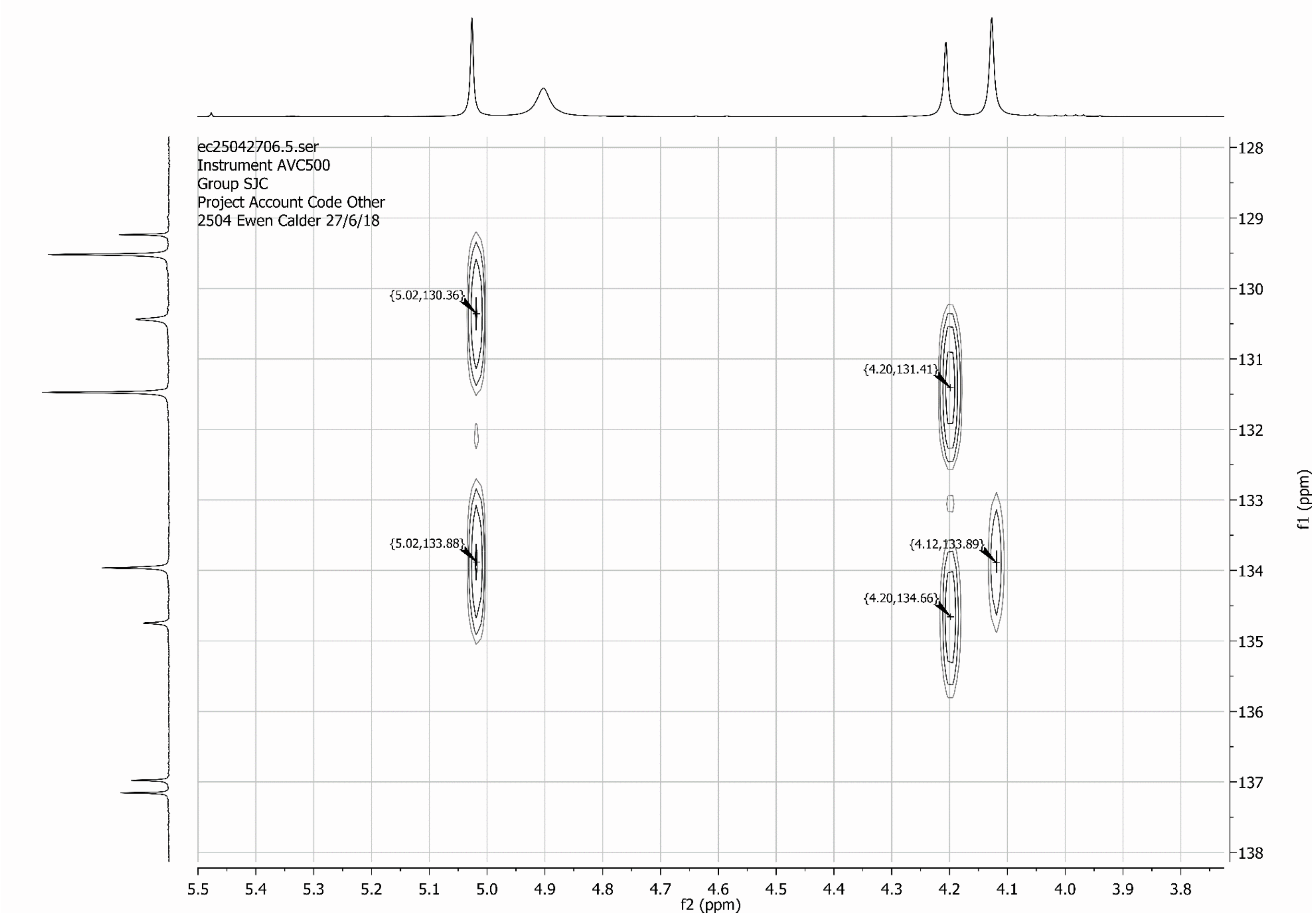

O-((1-Methyl-2-nitroimidazol-5-yl)methyl)-Panobinostat di-trifluoroacetate (CH-03, NI-Pano, **1**)

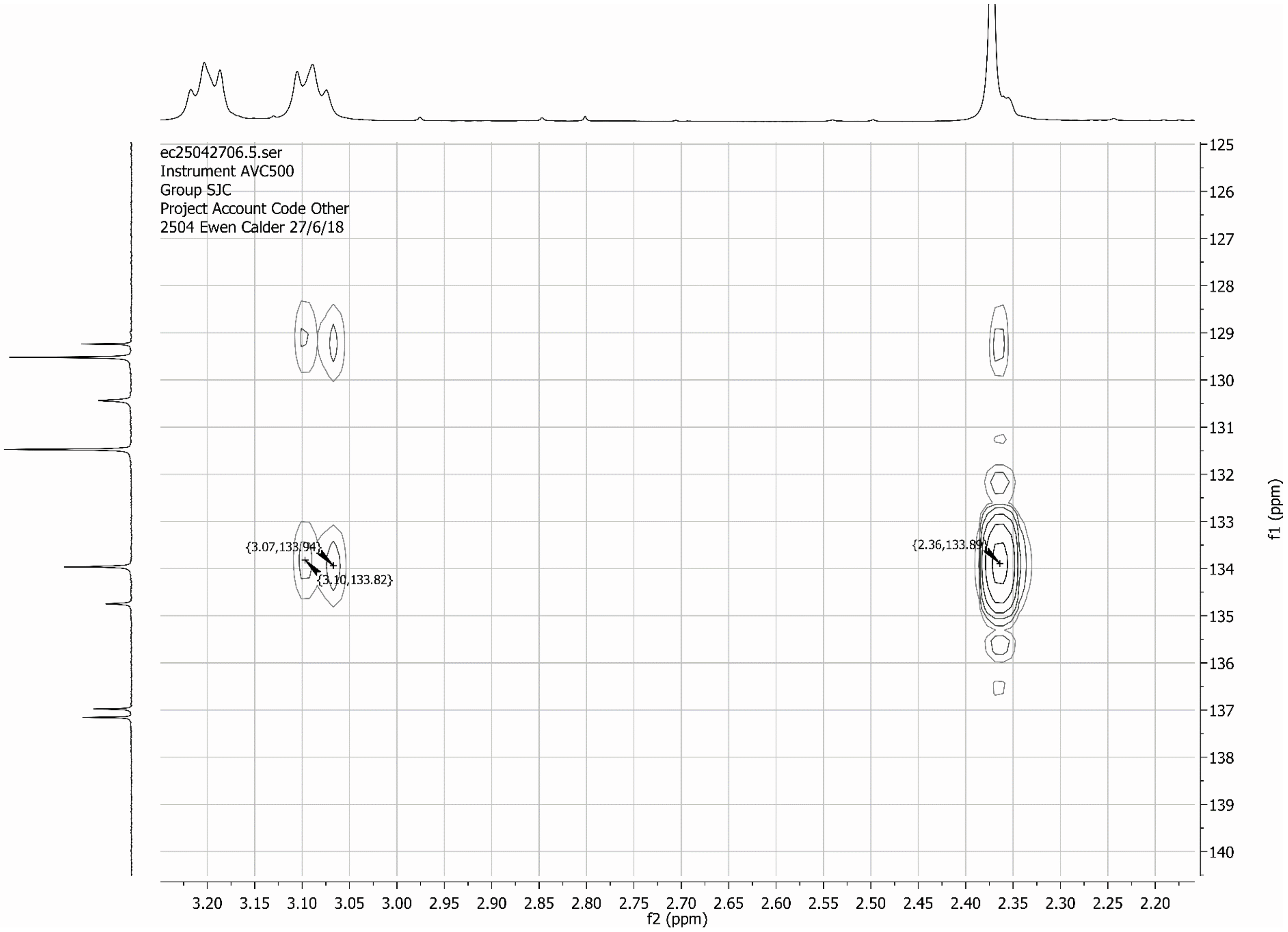

(*E*)-3-(4-{[2-(2-methyl-1H-indol-3-yl)ethylamino]methyl}phenyl)prop-2-enoic acid trifluoroacetate (**S18**)

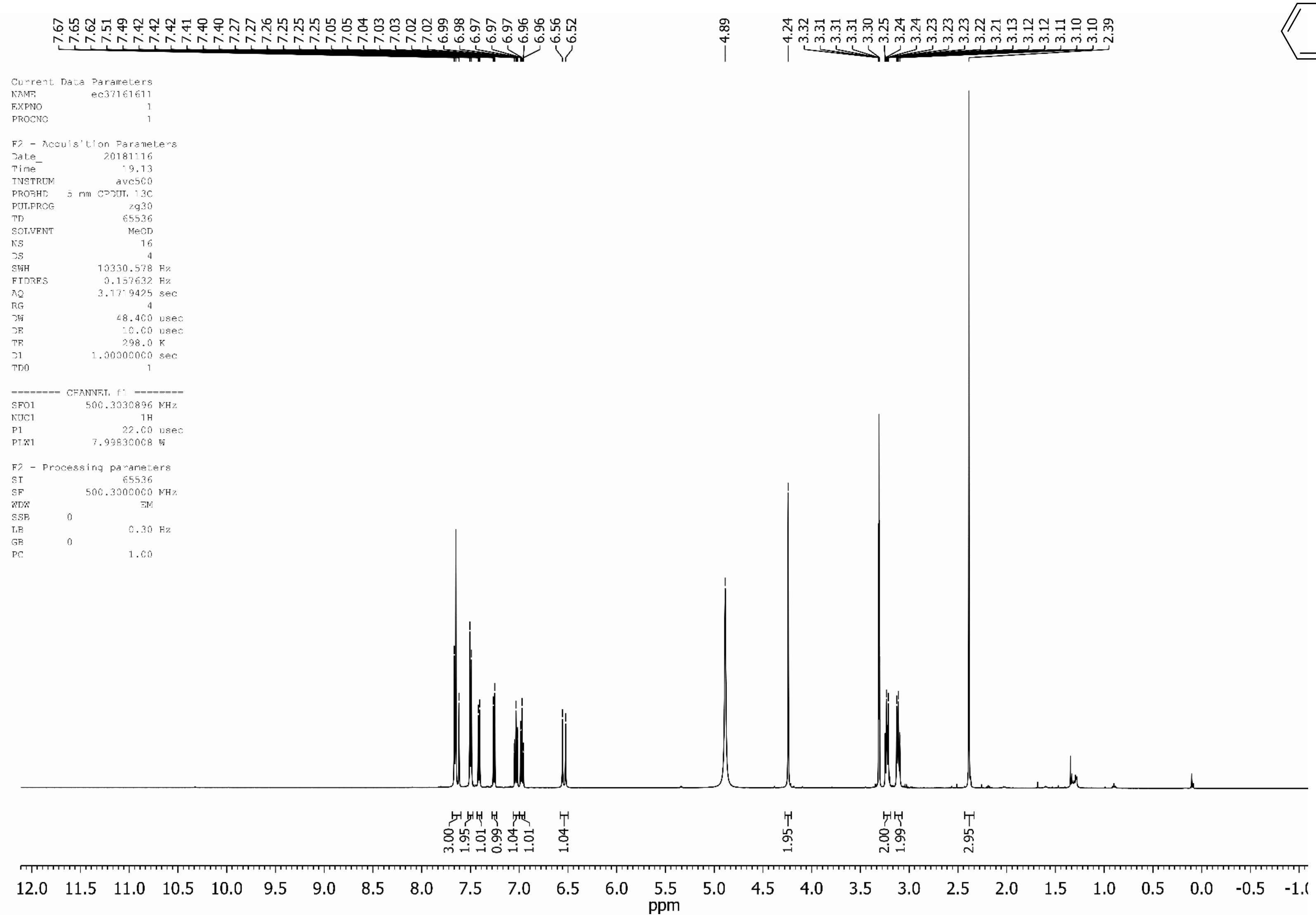

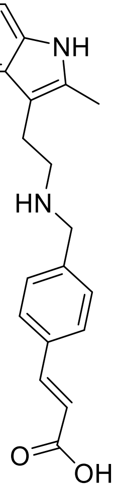

(*E*)-3-(4-{[2-(2-methyl-1H-indol-3-yl)ethylamino]methyl}phenyl)prop-2-enoic acid trifluoroacetate (**S18**)

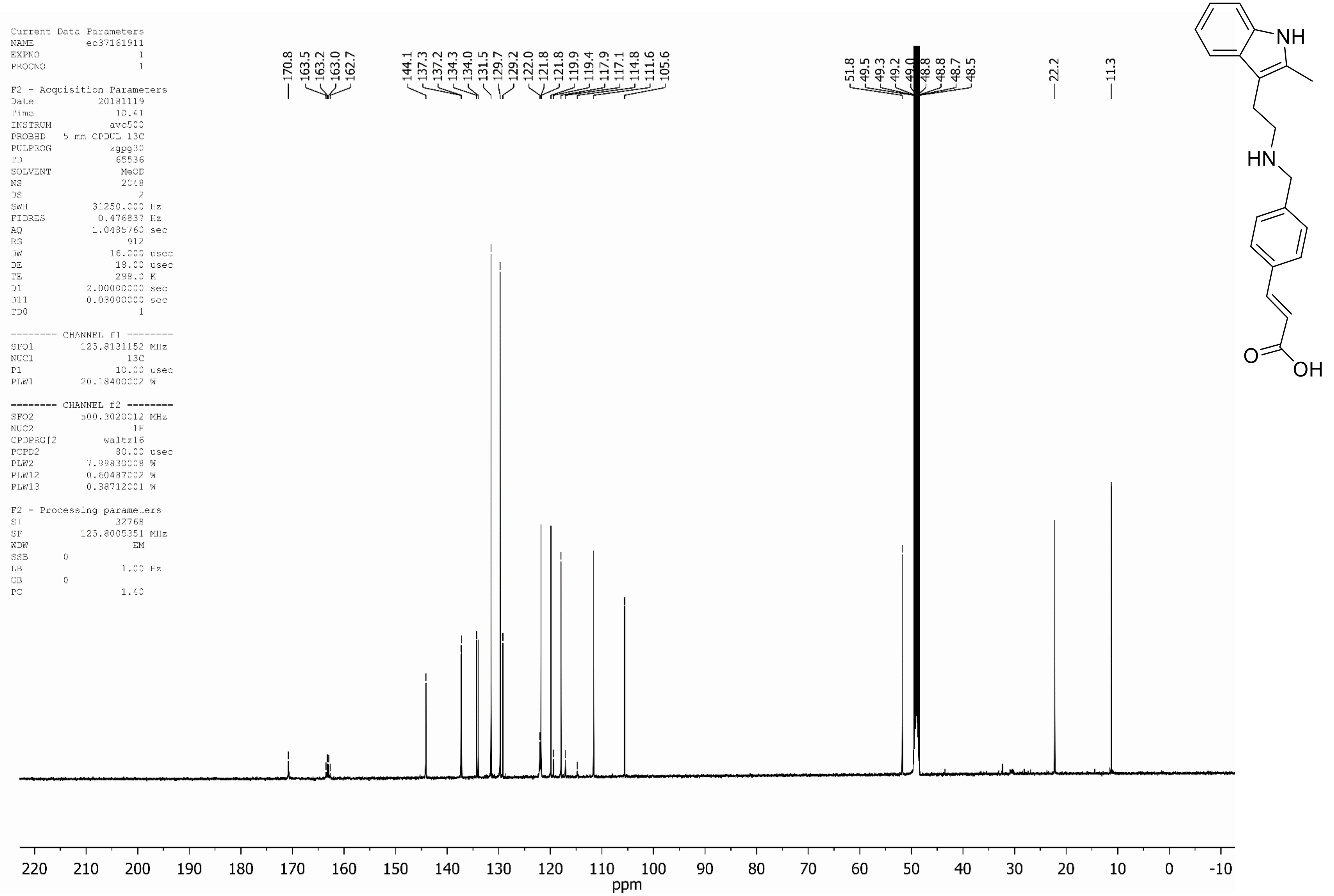

(*E*)-3-(4-{[2-(2-methyl-1*H*-indol-3-yl)ethylamino]methyl}phenyl)prop-2-enoic acid trifluoroacetate (**S18**)

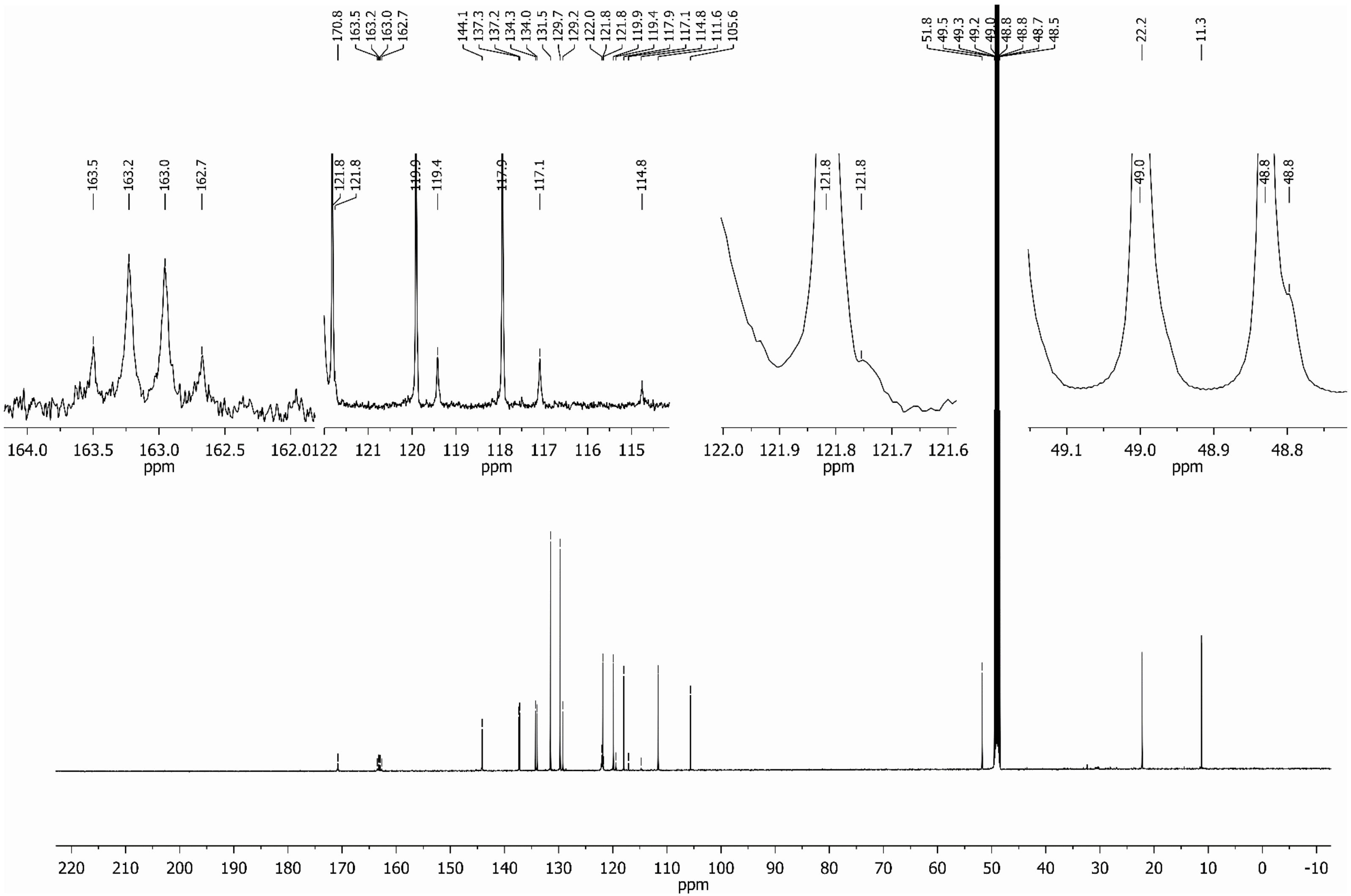

4-((1-Methyl-2-nitro-1*H*-imidazol-5-yl)methoxy)benzaldehyde

Current Data Parameters
NAME mo604701501 MLOx120
EXPNO 1
PROCNO 1

F2 - Acquisition Parameters
Date_ 20210117
Time 9.11
INSTRUM avc500
PROBHD 5 mm CPDUL 13C
PULPROG zg30
TD 65536
SOLVENT CDCl3
NS 16
DS 4
SWH 10330.578 Hz
FIDRES 0.157632 Hz
AQ 3.1719425 sec
RG 4
DW 48.400 usec
DE 10.00 usec
TE 298.0 K
D1 1.00000000 sec
TD0 1

======== CHANNEL f1 ========
SFO1 500.3030896 MHz
NUC1 1H
P1 22.00 usec
PLW1 7.99830008 W

F2 - Processing parameters
SI 65536
SF 500.3000131 MHz
WDW EM
SSB 0
LB 0.30 Hz
GB 0
PC 1.00

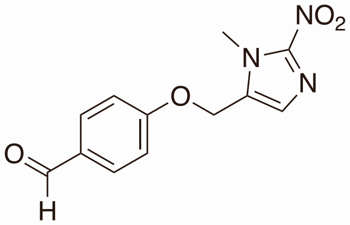

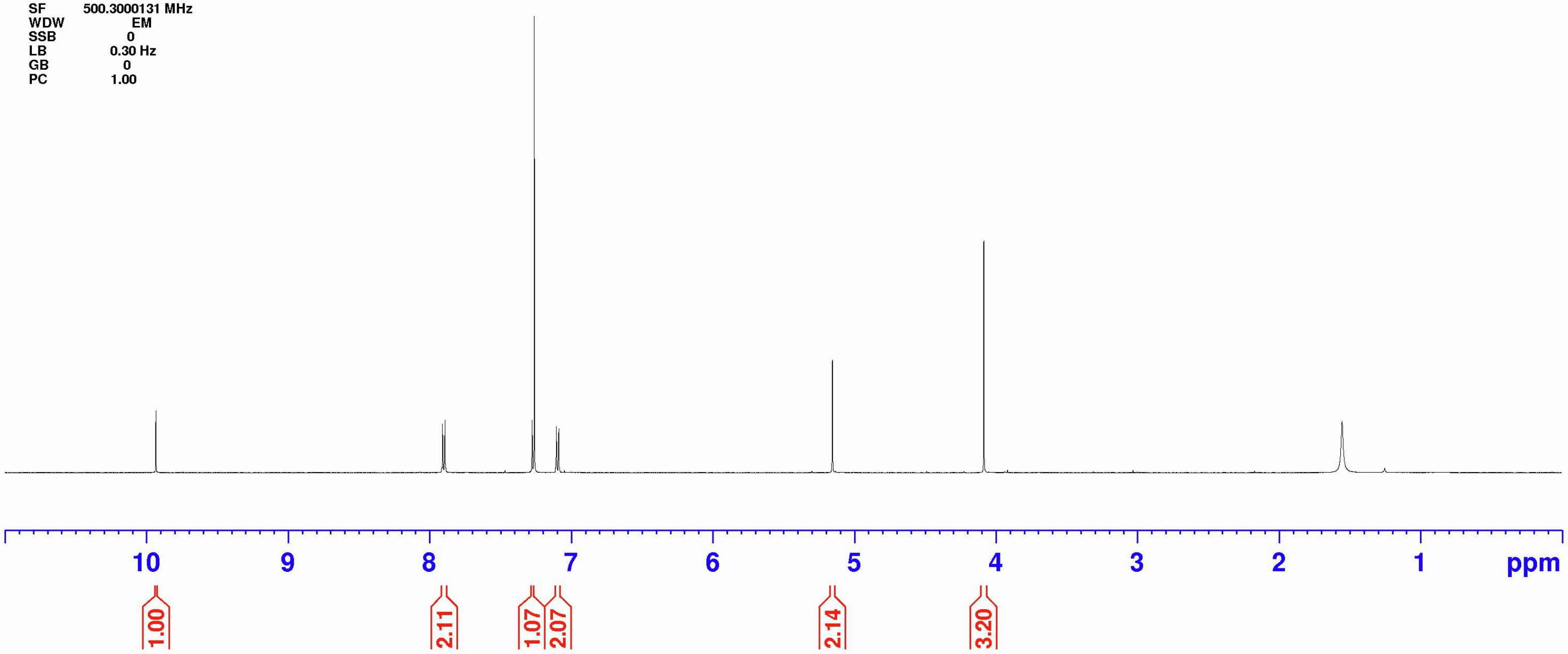

4-((1-Methyl-2-nitro-1*H*-imidazol-5-yl)methoxy)benzaldehyde

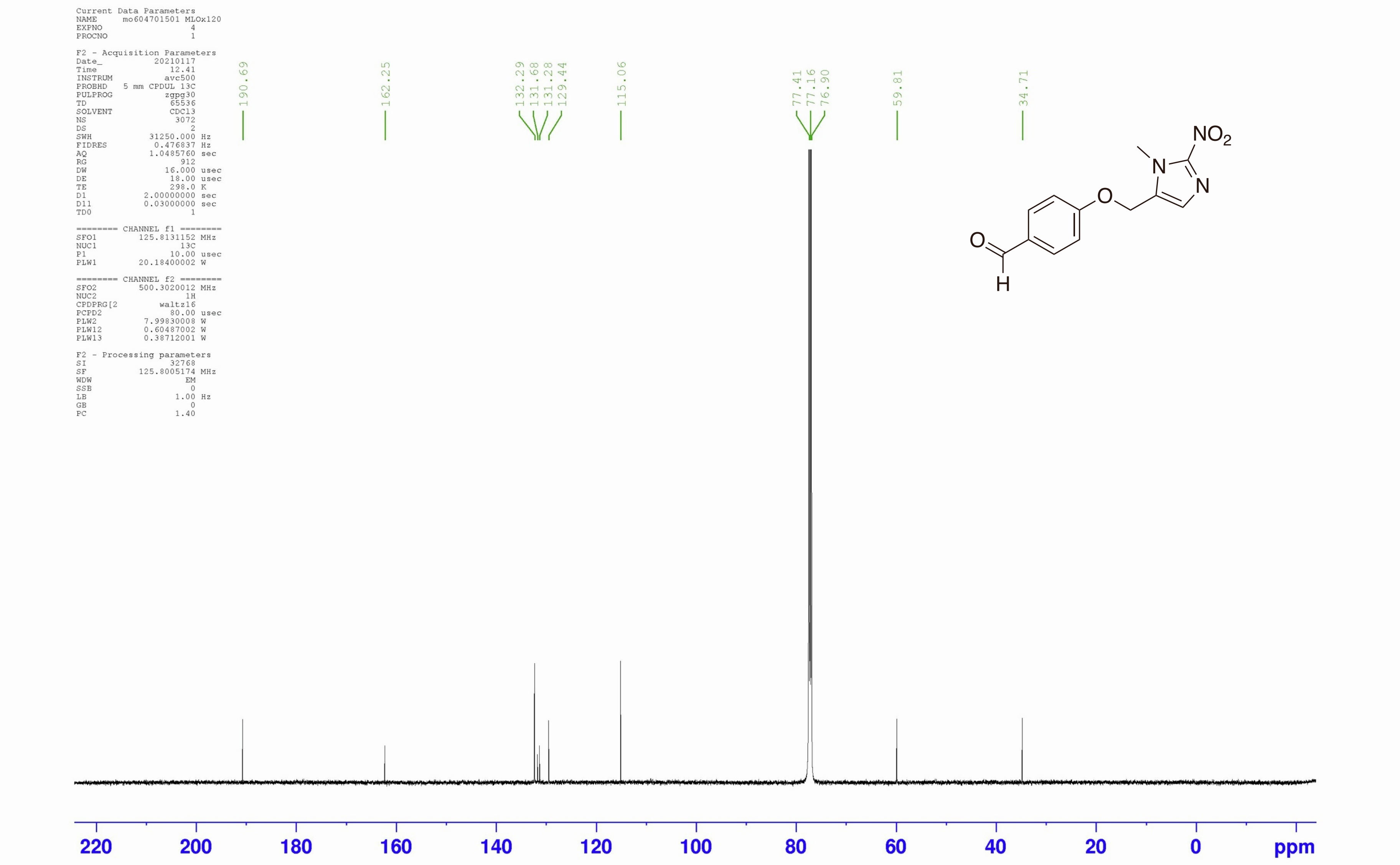

(*E*)-2-(2-(4-((1-Methyl-2-nitro-1*H*-imidazol-5-yl)methoxy)styryl)-4*H*-chromen-4-ylidene)malononitrile

Current Data Parameters
NAME mo605021801 - MLOx121
EXPNO 1
PROCNO 1

F2 - Acquisition Parameters
Date_ 20210119
Time 22.51
INSTRUM avc500
PROBHD 5 mm CPDUL 13C
PULPROG zg30
TD 65536
SOLVENT CDCl3
NS 16
DS 4
SWH 10330.578 Hz
FIDRES 0.157632 Hz
AQ 3.1719425 sec
RG 4.5
DW 48.400 usec
DE 10.00 usec
TE 298.0 K
D1 1.00000000 sec
TD0 1

======== CHANNEL f1 ========
SFO1 500.3030896 MHz
NUC1 1H
P1 22.00 usec
PLW1 7.99830008 W

F2 - Processing parameters
SI 65536
SF 500.3000129 MHz
WDW EM
SSB 0
LB 0.30 Hz
GB 0
PC 1.00

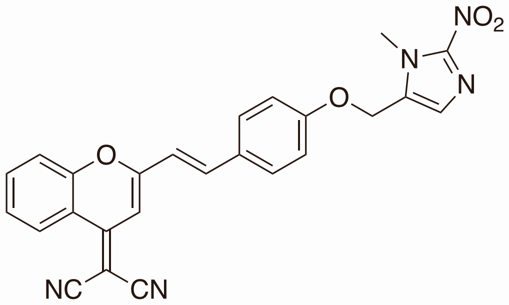

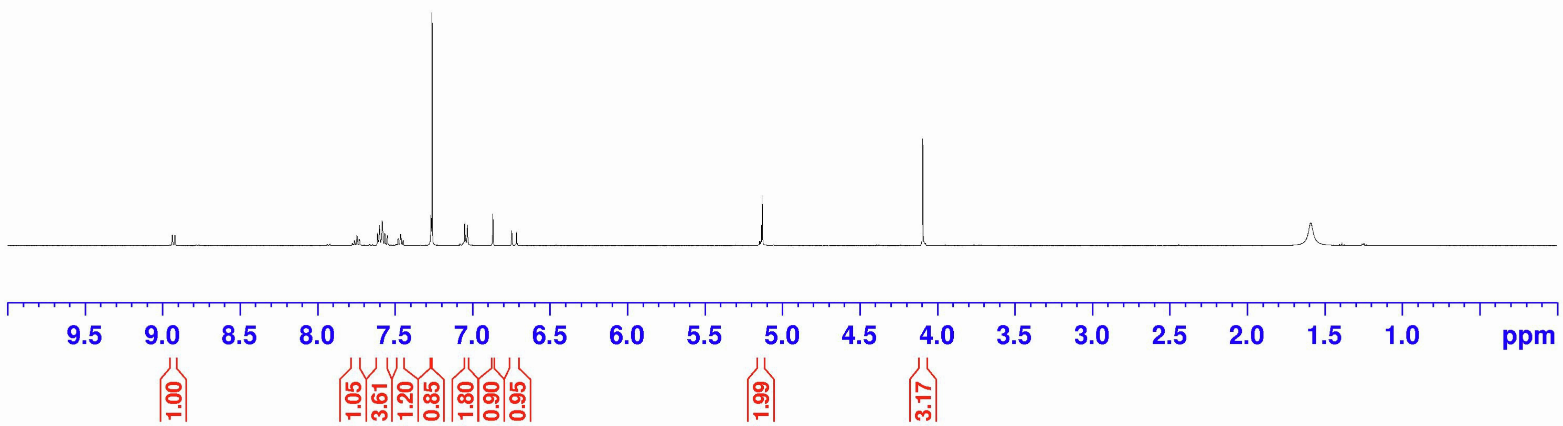

(*E*)-2-(2-(4-((1-Methyl-2-nitro-1*H*-imidazol-5-yl)methoxy)styryl)-4*H*-chromen-4-ylidene)malononitrile

```
Current Data Parameters
NAME      mo605021801 - MLOx121
EXPNO                    4
PROCNO                   1

F2 - Acquisition Parameters
Date_             20210120
Time                  2.59
INSTRUM             avc500
PROBHD   5 mm CPDUL 13C
PULPROG            zgpg30
TD                   65536
SOLVENT              CDCl3
NS                    3072
DS                       2
SWH          31250.000 Hz
FIDRES        0.476837 Hz
AQ           1.0485760 sec
RG                     912
DW              16.000 usec
DE               18.00 usec
TE               298.0 K
D1          2.00000000 sec
D11         0.03000000 sec
TD0                      1

======== CHANNEL f1 ========
SFO1       125.8131152 MHz
NUC1                   13C
P1               10.00 usec
PLW1       20.18400002 W

======== CHANNEL f2 ========
SFO2       500.3020012 MHz
NUC2                    1H
CPDPRG[2           waltz16
PCPD2            80.00 usec
PLW2        7.99830008 W
PLW12       0.60487002 W
PLW13       0.38712001 W

F2 - Processing parameters
SI                   32768
SF         125.8005182 MHz
WDW                     EM
SSB                      0
LB                1.00 Hz
GB                       0
PC                    1.40
```

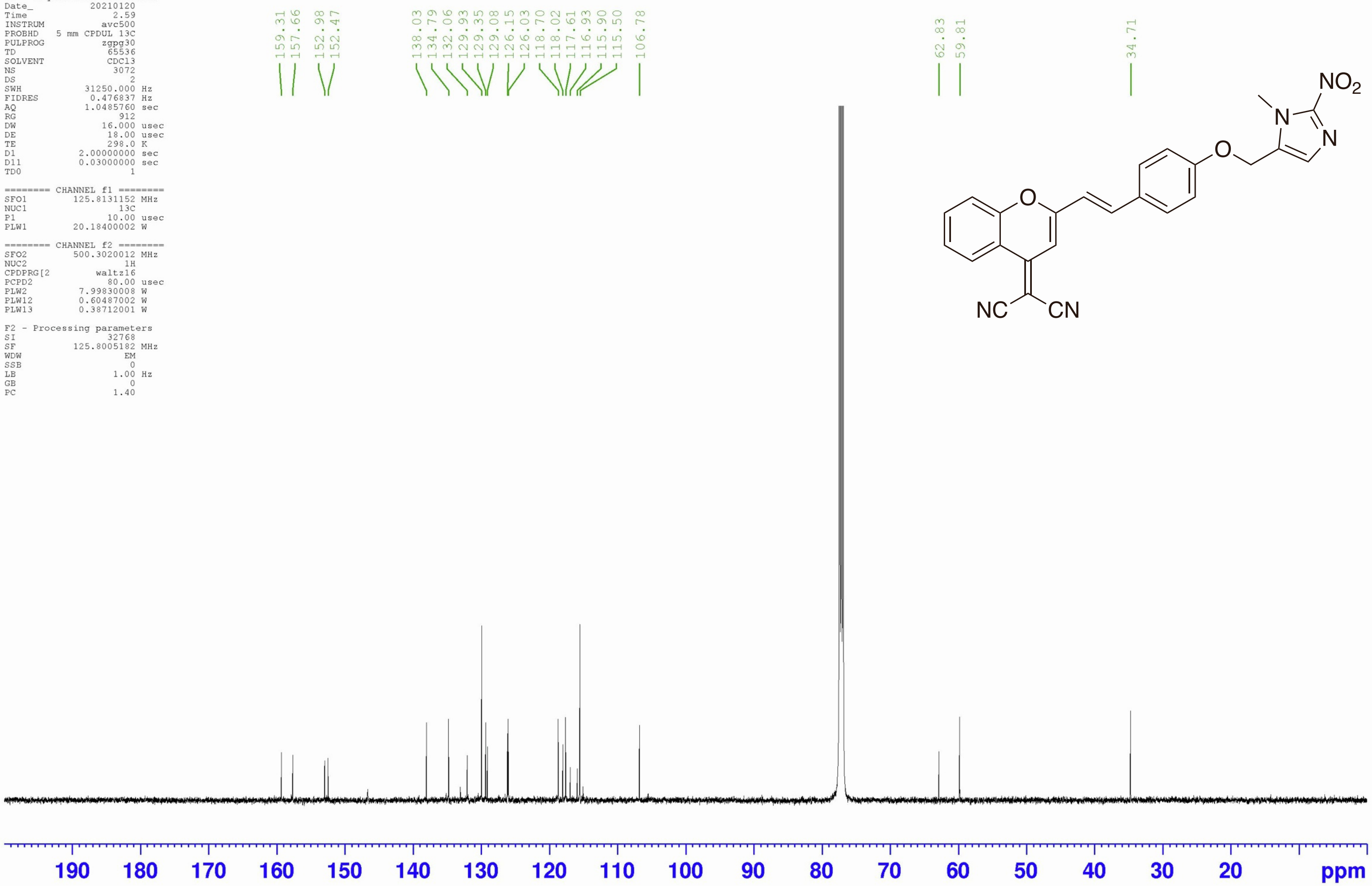

Methyl (*E*)-3-(4-{[2-(2-methyl-*1H*-indol-3-yl)ethylamino]methyl}phenyl)prop-2-enoate (**3**)

EC2-10 **Purity short run @254 nm** **2/1/2017 8:37 pm**

Sample Name	EC2-10
Vial Number	6
Injection Volume	5
Acquisition Date/Time	1/31/2017 6:02 pm
Acquisition Method	Purity short run @254 nm
Processing Method	Purity short run @254 nm

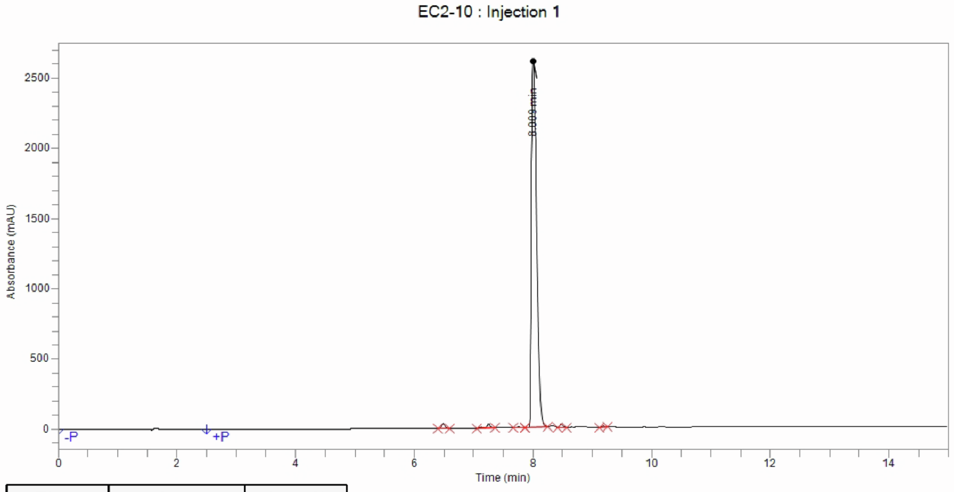

Time	Area	Area %
6.492	142,182.4	0.86
7.131	27,766.8	0.17
7.257	110,631.7	0.67
7.753	39,724.4	0.24
8.009	16,026,271.6	97.35
8.475	88,047.3	0.53
9.188	28,156.0	0.17
**Total**	16,462,780.3	100.00

2/1/2017 8:37 pm **Flexar HPLC 2**

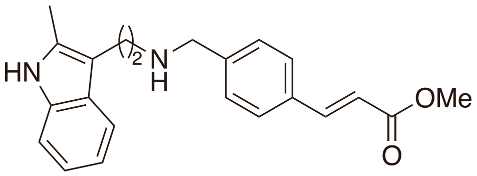

Panobinostat (**2**)

EC4-14 Pano **2/26/2018**

Acquisition Method	Purity short run @254 nm
Acquisition Date/Time	2/26/2018 6:56 pm
Injection Volume	10
Sample Name	EC4-14 Pano
Sample Description	

EC4-14 Pano : Injection 1

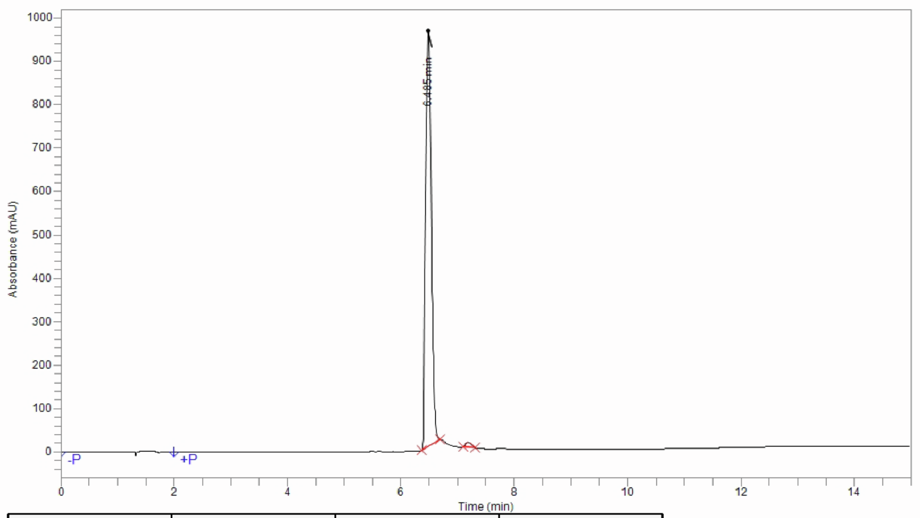

Time	Height	Area	Area %
6.485	958,967.0	6,619,001.0	98.81
7.183	11,494.8	79,439.7	1.19
**Total**		6,698,440.7	100.00

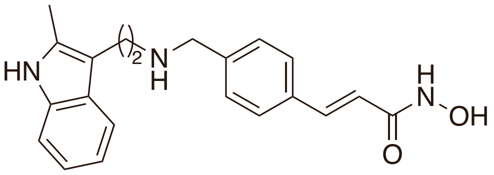

(5-Nitrothiophen-2-yl)methyl bromide (**S9**)

EC4-63 9/11/2018

Acquisition Method	Purity short run @254 nm
Acquisition Date/Time	6/25/2018 4:14 pm
Injection Volume	10
Sample Name	EC4-63
Sample Description	
Batch Description	

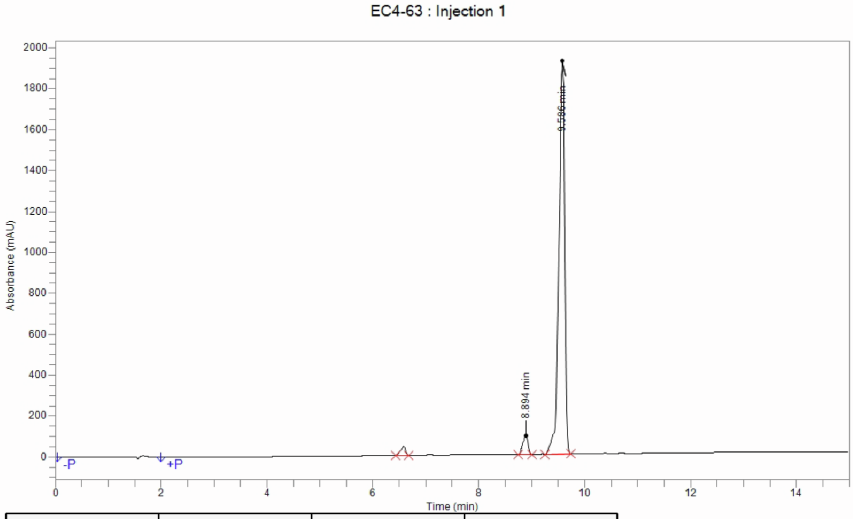

Time	Height	Area	Area %
6.585	43,937.1	298,598.4	1.88
8.894	91,542.3	599,907.0	3.78
9.586	1,923,605.5	14,976,498.3	94.34
**Total**		15,875,003.6	100.00

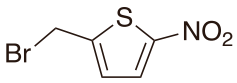

N-Phthalimido-*O*-(5-nitrothiophen-2-yl)-hydroxylamine

EC4-68 **9/11/2018**

Acquisition Method	Purity short run @254 nm
Acquisition Date/Time	7/3/2018 5:41 pm
Injection Volume	10
Sample Name	EC4-68
Sample Description	
Batch Description	

EC4-68 : Injection 1

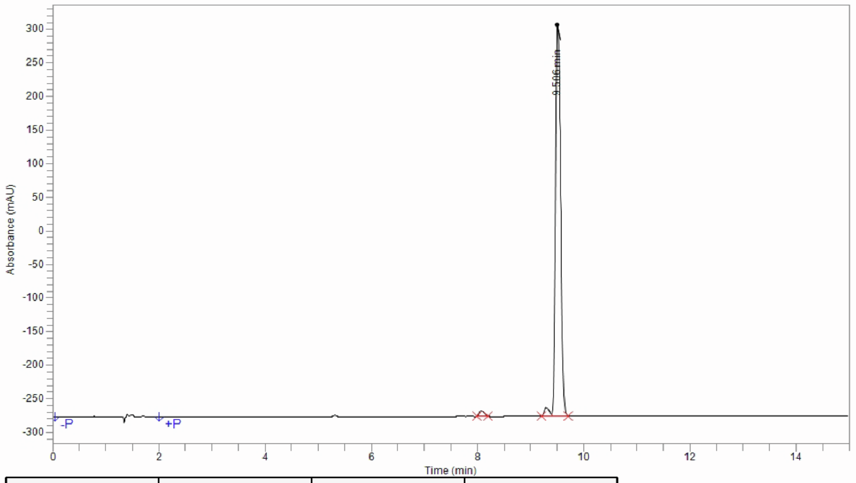

Time	Height	Area	Area %
8.071	8,336.6	51,781.2	1.29
9.292	12,498.6	81,327.4	2.03
9.506	582,893.5	3,865,476.7	96.67
**Total**		3,998,585.4	100.00

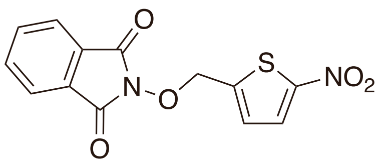

O-(5-Nitrothiophen-2-yl)-hydroxylamine (**S10**)

Acquisition Method	Purity short run @254 nm
Acquisition Date/Time	10/1/2018 2:41 pm
Injection Volume	10
Sample Name	EC4-90
Sample Description	
Batch Description	

EC4-90 : Injection 1

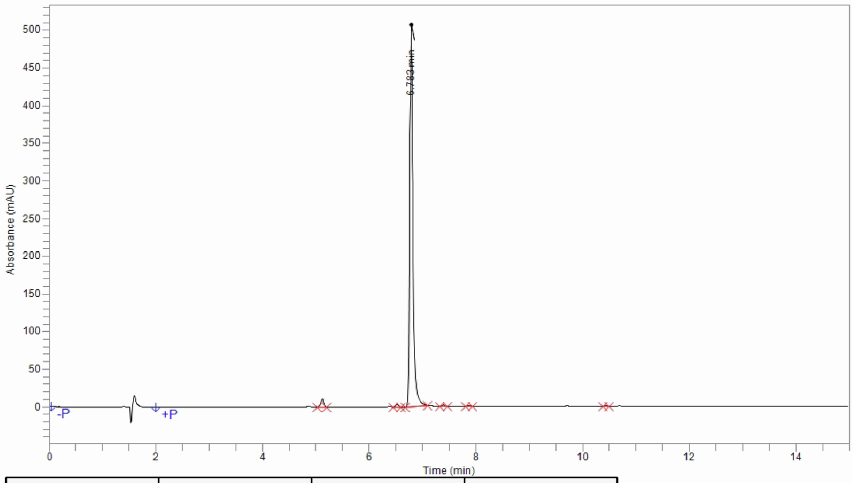

Time	Height	Area	Area %
5.119	11,409.7	44,269.3	1.93
6.518	3,648.0	13,869.0	0.61
6.783	507,580.5	2,210,708.1	96.60
7.387	2,336.8	8,811.9	0.39
7.870	1,697.5	6,964.7	0.30
10.436	1,216.3	3,794.2	0.17
**Total**		2,288,417.3	100.00

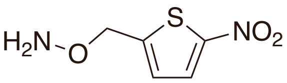

N-Phthalimido-*O*-(6-nitroquinolin-5-yl)-hydroxylamine

EC3-88 **9/12/2018**

Acquisition Method	Purity short run @254 nm
Acquisition Date/Time	1/8/2018 1:50 pm
Injection Volume	20
Sample Name	EC3-88
Sample Description	
Batch Description	

EC3-88 : Injection 1

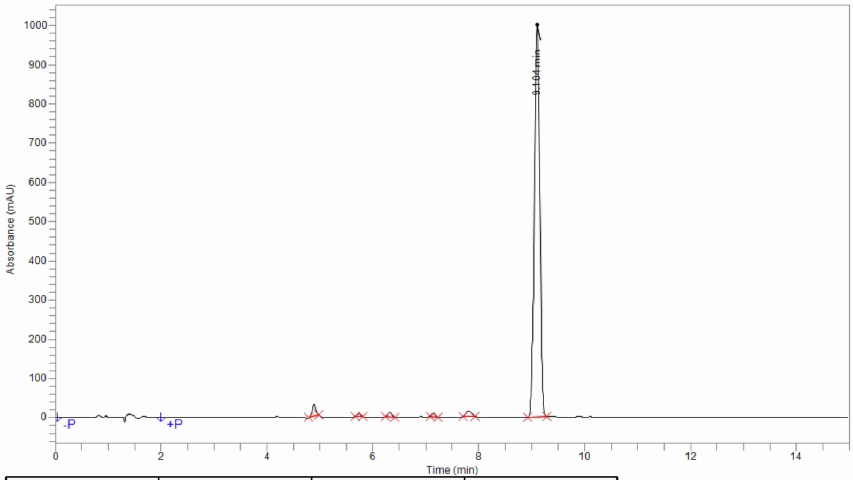

Time	Height	Area	Area %
4.891	29,133.4	145,138.3	1.98
5.735	6,556.7	31,610.1	0.43
6.319	11,319.6	57,009.5	0.78
7.138	6,535.2	32,462.8	0.44
7.807	13,451.0	94,303.7	1.28
9.104	1,001,339.3	6,982,876.3	95.09
**Total**		7,343,400.6	100.00

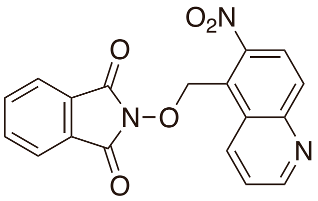

O-(6-Nitroquinolin-5-yl)-hydroxylamine (**S14**)

EC3-89 9/12/2018

Acquisition Method	Purity short run @254 nm
Acquisition Date/Time	1/8/2018 2:25 pm
Injection Volume	20
Sample Name	EC3-89
Sample Description	
Batch Description	

EC3-89 : Injection 1

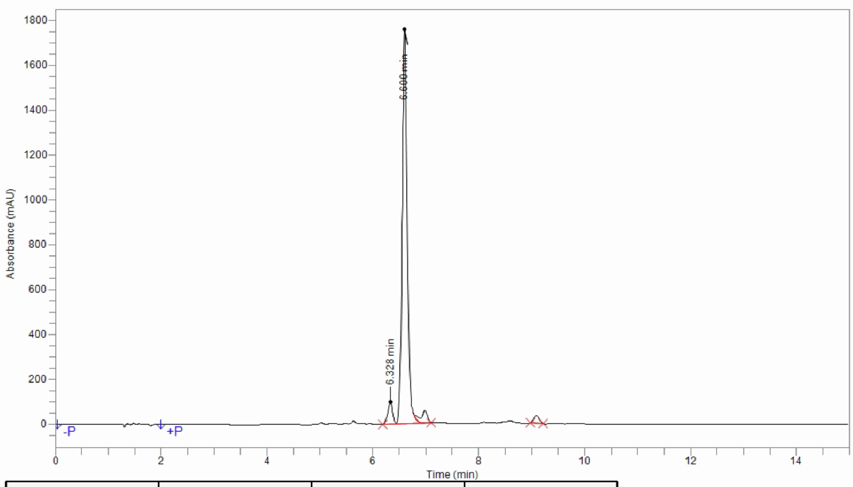

Time	Height	Area	Area %
6.328	99,800.0	620,898.6	4.75
6.600	1,756,176.4	11,754,905.5	89.95
6.986	53,118.9	455,530.4	3.49
9.092	34,047.3	236,249.0	1.81
**Total**		13,067,583.6	100.00

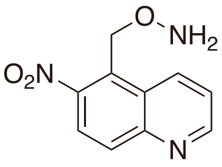

Methyl (*E*)-3-(4-{[*tert*-butyloxycarbonyl-(2-{1-[*tert*-butyloxycarbonyl]-2-methyl-*1H*-indol-3-yl}ethyl)amino]methyl}phenyl)prop-2-enoate (**4**)

EC2-38 **Purity short run @254 nm** **2/1/2017 1:48 pm**

Sample Name EC2-38
Vial Number 1
Injection Volume 10
Acquisition Date/Time 2/1/2017 9:45 am
Acquisition Method Purity short run @254 nm
Processing Method Purity short run @254 nm

EC2-38 : Injection 1

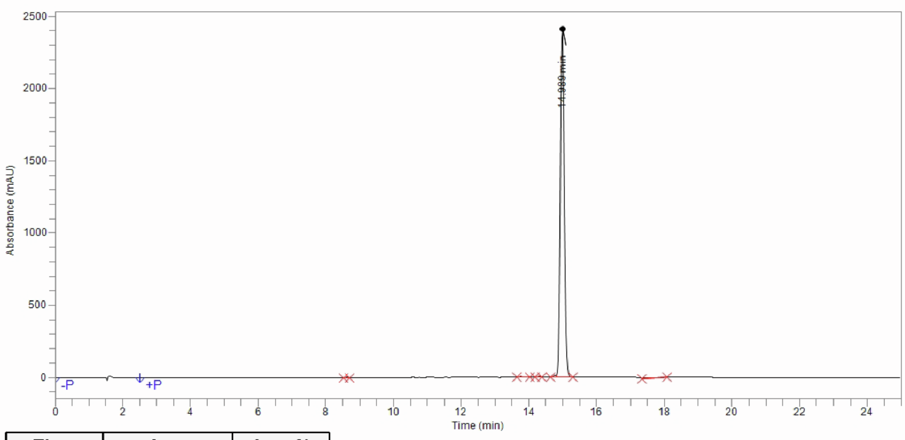

Time	Area	Area %
8.624	8,997.7	0.05
13.759	34,580.5	0.18
13.941	13,625.3	0.07
14.296	33,348.9	0.17
14.789	82,617.3	0.43
14.989	18,982,727.6	98.24
18.018	167,802.4	0.87
**Total**	19,323,699.6	100.00

2/1/2017 1:48 pm **Flexar HPLC 2**

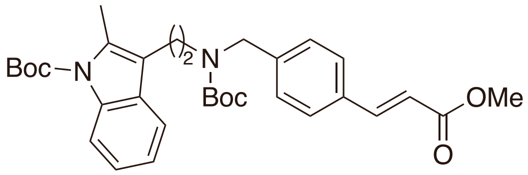

(*E*)-3-(4-{[*tert*-Butyloxycarbonyl-(2-{1-[*tert*-butyloxycarbonyl]-2-methyl-*1H*-indol-3-yl}ethyl)amino]methyl}phenyl)prop-2-enoic acid (**5**)

EC2-39 **EC Purity short run @254 nm longer** **2/2/2017 5:15 pm**

Sample Name	EC2-39
Vial Number	7
Injection Volume	5
Acquisition Date/Time	2/2/2017 4:55 pm
Acquisition Method	EC Purity short run @254 nm longer
Processing Method	EC Purity short run @254 nm longer

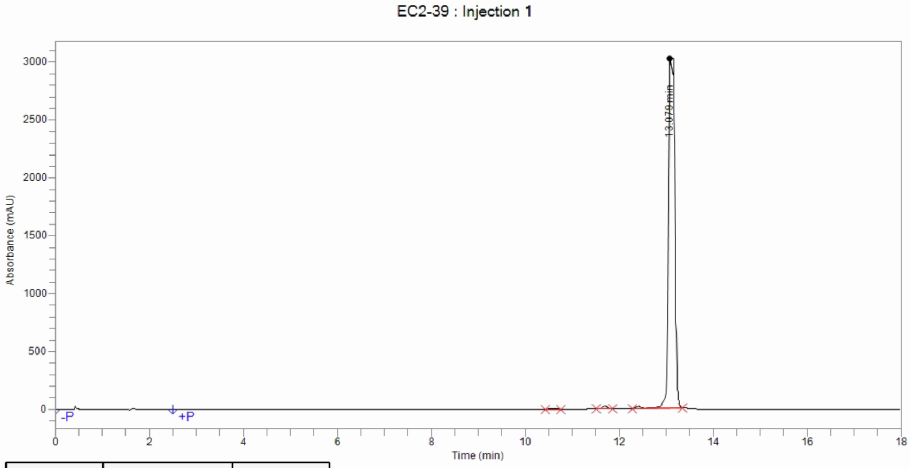

Time	Area	Area %
10.601	49,007.8	0.17
11.713	182,154.1	0.63
12.427	140,338.8	0.48
13.079	28,606,702.2	98.72
**Total**	28,978,202.8	100.00

2/2/2017 5:15 pm **Flexar HPLC 2**

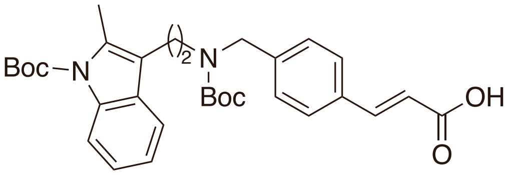

N,N-Di-Boc-O-benzyl-panobinostat (**6**)

EC4-92 10/10/2018

Acquisition Method Purity short run @254 nm
Acquisition Date/Time 10/10/2018 12:27 pm
Injection Volume 10
Sample Name EC4-92
Sample Description
Batch Description

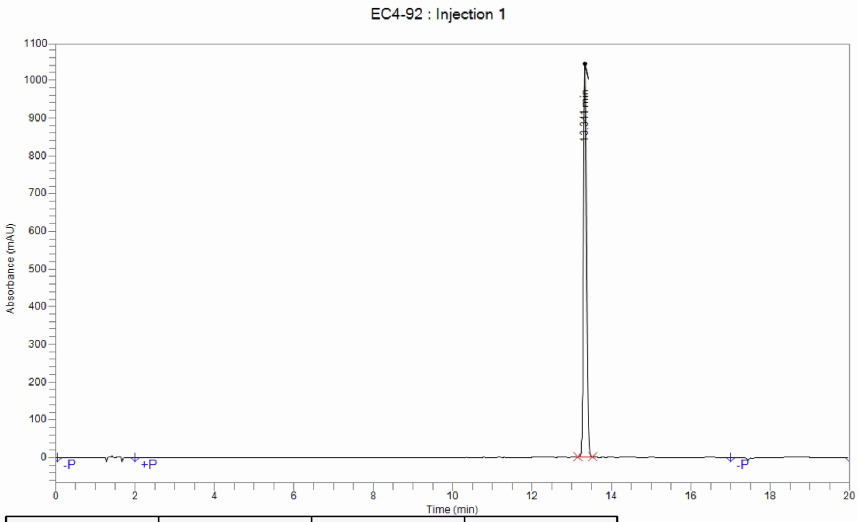

Time	Height	Area	Area %
13.341	1,044,426.9	5,146,725.1	100.00
**Total**		5,146,725.1	100.00

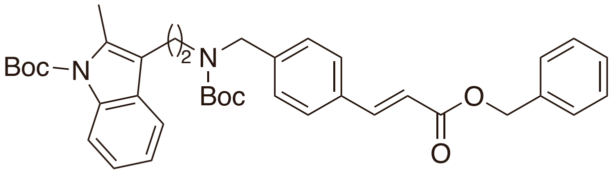

N,N-Di-Boc-*O*-(4-nitrobenzyl)-panobinostat (**7**)

EC4-61 **9/19/2018**

Acquisition Method	Purity short run @254 nm
Acquisition Date/Time	6/22/2018 4:51 pm
Injection Volume	10
Sample Name	EC4-61
Sample Description	
Batch Description	

EC4-61 : Injection 1

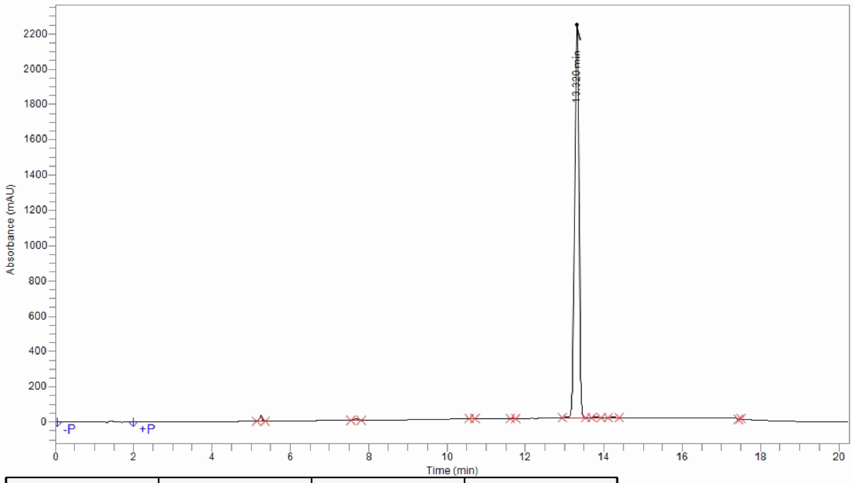

Time	Height	Area	Area %
5.258	33,504.5	167,128.0	0.96
7.687	10,754.0	76,820.7	0.44
10.632	2,487.5	12,000.8	0.07
11.671	2,152.6	9,633.4	0.06
13.320	2,229,257.0	17,107,225.8	98.04
13.802	3,814.4	30,947.5	0.18
14.244	4,433.2	38,467.9	0.22
17.455	2,460.5	6,740.1	0.04
**Total**		17,448,964.1	100.00

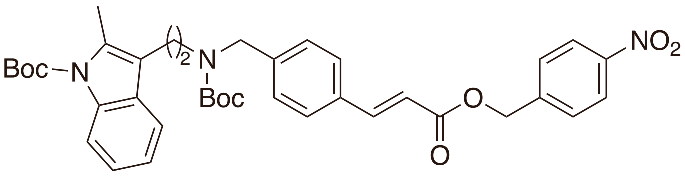

N,N-Di-Boc-O-(2-nitrothiophen-5-yl)methyl)-panobinostat (**8**)

EC2-35 **EC Purity short run @254 nm longer** **2/1/2017 6:23 pm**

Sample Name	EC2-35
Vial Number	12
Injection Volume	10
Acquisition Date/Time	2/1/2017 5:38 pm
Acquisition Method	EC Purity short run @254 nm longer
Processing Method	EC Purity short run @254 nm longer

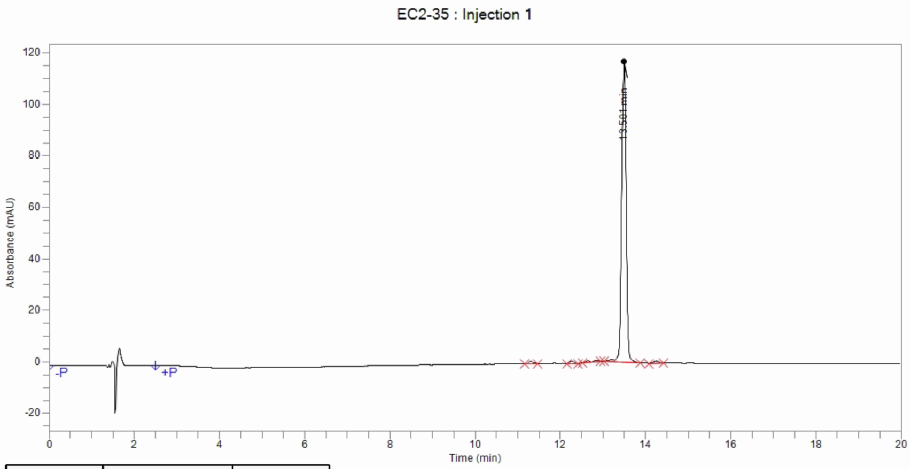

Time	Area	Area %
11.309	7,739.2	0.88
12.284	5,856.3	0.67
12.649	5,843.2	0.66
12.853	3,870.5	0.44
13.185	6,107.3	0.69
13.501	840,590.3	95.64
14.270	8,871.3	1.01
**Total**	878,878.1	100.00

2/1/2017 6:23 pm **Flexar HPLC 2**

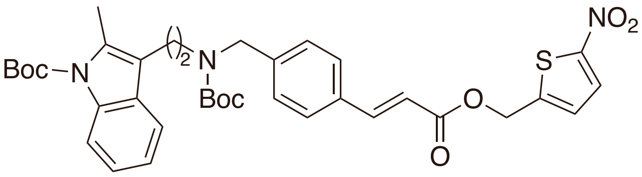

N,N-Di-Boc-*O*-((6-nitroquinolin-5-yl)methyl)-panobinostat (**9**)

EC3-90 **9/20/2018**

Acquisition Method	Purity short run @254 nm
Acquisition Date/Time	1/8/2018 3:01 pm
Injection Volume	10
Sample Name	EC3-90
Sample Description	
Batch Description	

EC3-90 : Injection 1

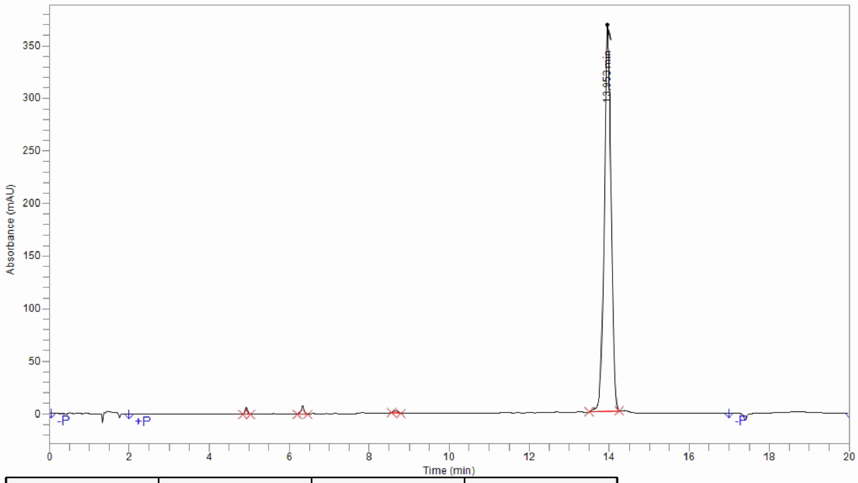

Time	Height	Area	Area %
4.927	6,738.4	31,633.7	0.70
6.332	7,877.7	48,338.7	1.07
8.658	2,341.0	16,158.8	0.36
13.953	368,327.9	4,423,922.2	97.87
**Total**		4,520,053.3	100.00

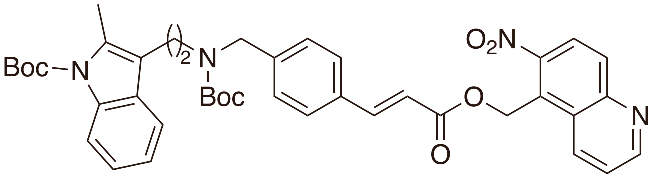

N,N-Di-Boc-*O*-((1-methyl-2-nitroimidazol-5-yl)methyl)-panobinostat

EC5-06 12/11/2018

Acquisition Method	Purity short run @254 nm with long hold
Acquisition Date/Time	12/11/2018 2:40 pm
Injection Volume	10
Sample Name	EC5-06
Sample Description	
Batch Description	

EC5-06 : Injection 1

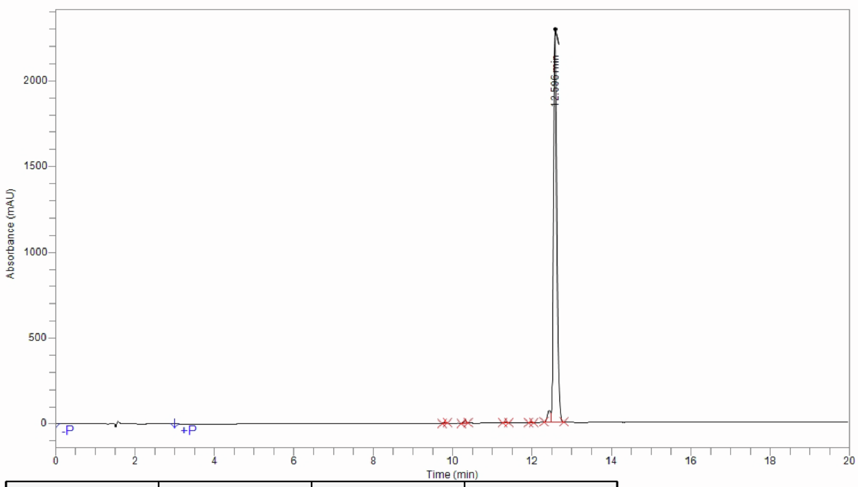

Time	Height	Area	Area %
9.809	5,860.4	23,457.5	0.18
10.319	16,539.6	69,084.1	0.54
11.351	5,998.6	25,916.7	0.20
11.990	3,952.6	15,955.0	0.12
12.440	68,462.0	327,459.7	2.54
12.596	2,293,450.6	12,416,674.7	96.41
**Total**		12,878,547.7	100.00

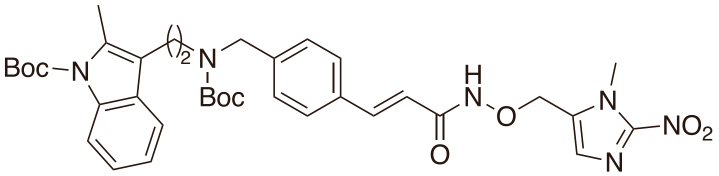

O-Benzyl-panobinostat (Bn-Pano, **10**)

EC5-03 **11/15/2018**

Acquisition Method	Purity short run @254 nm
Acquisition Date/Time	11/15/2018 10:41 am
Injection Volume	10
Sample Name	EC5-03
Sample Description	
Batch Description	

EC5-03 : Injection 1

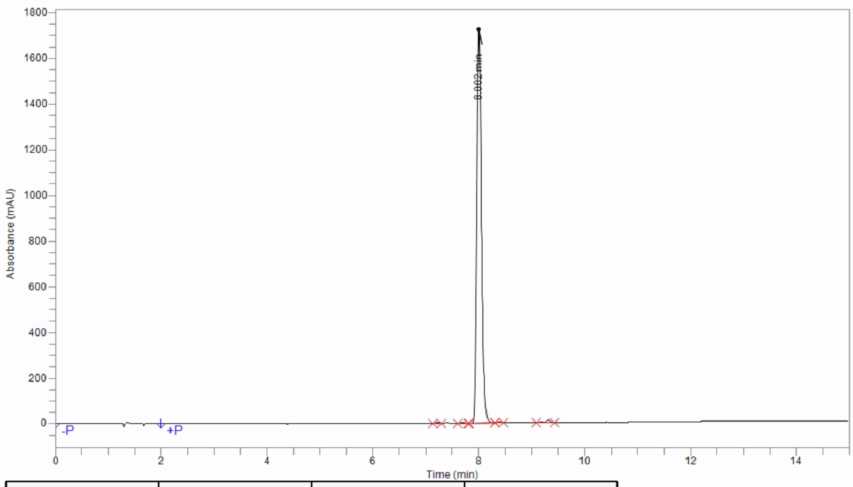

Time	Height	Area	Area %
7.223	3,653.0	16,559.7	0.16
7.696	3,057.3	14,995.5	0.14
8.002	1,727,141.3	10,237,501.0	98.80
8.393	4,153.3	17,709.5	0.17
9.185	3,472.0	16,907.9	0.16
9.317	11,731.4	58,166.0	0.56
**Total**		10,361,839.6	100.00

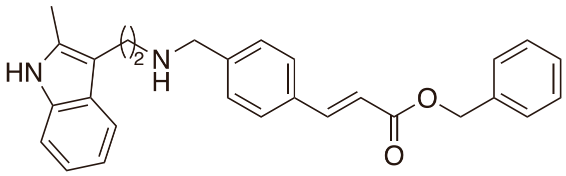

O-(4-Nitrobenzyl)-panobinostat trifluoroacetate (NB-Pano, **11**)

EC2-18 **Purity short run @254 nm** **11/4/2016 3:54 pm**

Sample Name	EC2-18
Vial Number	3
Injection Volume	20
Acquisition Date/Time	11/4/2016 1:24 pm
Acquisition Method	Purity short run @254 nm
Processing Method	Purity short run @254 nm

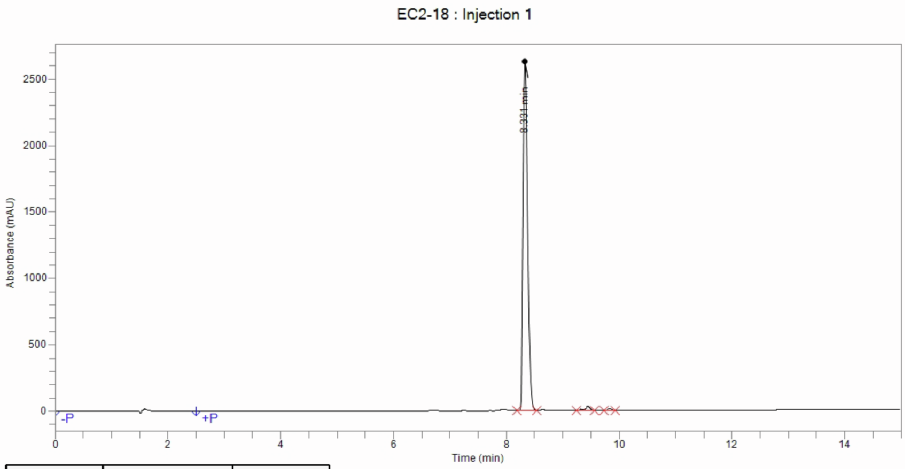

Time	Area	Area %
8.331	14,908,280.8	98.07
9.321	49,133.2	0.32
9.444	168,073.8	1.11
9.829	76,090.2	0.50
**Total**	15,201,577.9	100.00

11/4/2016 3:54 pm **Flexar HPLC 2**

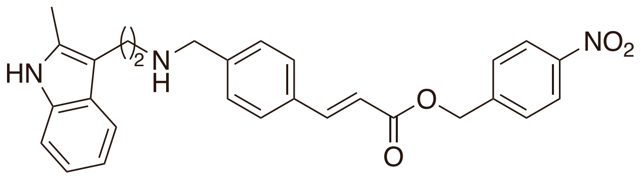

O-(5-Nitrothiophen-2-yl)methyl))-panobinostat trifluoroacetate (NT-Pano, **12**)

EC2-37 **Purity short run @254 nm** **1/11/2017 2:40 pm**

Sample Name	EC2-37
Vial Number	1
Injection Volume	10
Acquisition Date/Time	1/11/2017 2:22 pm
Acquisition Method	Purity short run @254 nm
Processing Method	Purity short run @254 nm

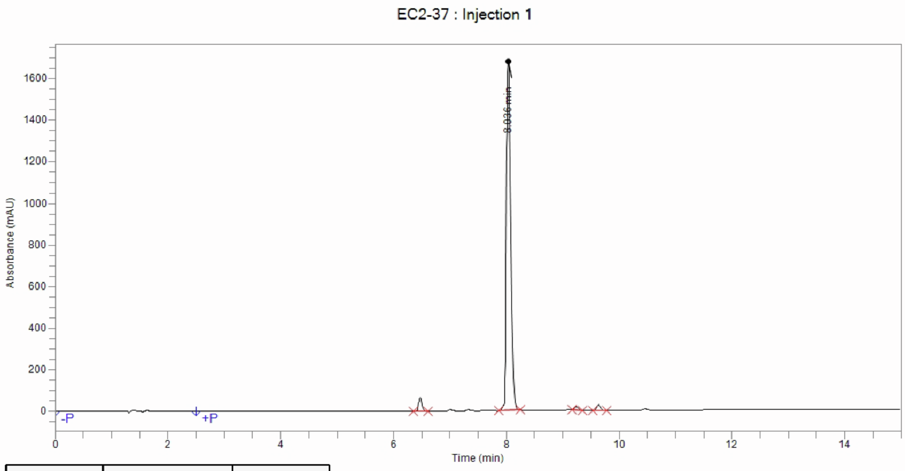

Time	Area	Area %
6.474	267,632.6	2.77
8.036	9,169,783.6	95.06
9.237	76,595.7	0.79
9.635	132,733.0	1.38
**Total**	9,646,744.8	100.00

1/11/2017 2:40 pm **Flexar HPLC 2**

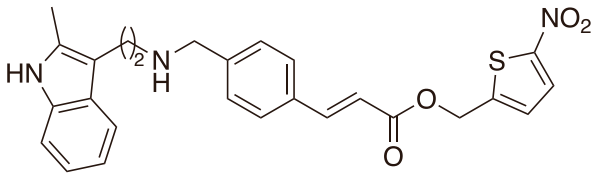

O-(6-Nitroquinolin-5-yl)methyl))-panobinostat di-trifluoroacetate (NQ-Pano, **13**)

EC3-91 1/4/2019

Acquisition Method	Purity short run @254 nm
Acquisition Date/Time	1/10/2018 12:56 pm
Injection Volume	10
Sample Name	EC3-91
Sample Description	
Batch Description	EC

EC3-91 : Injection 1

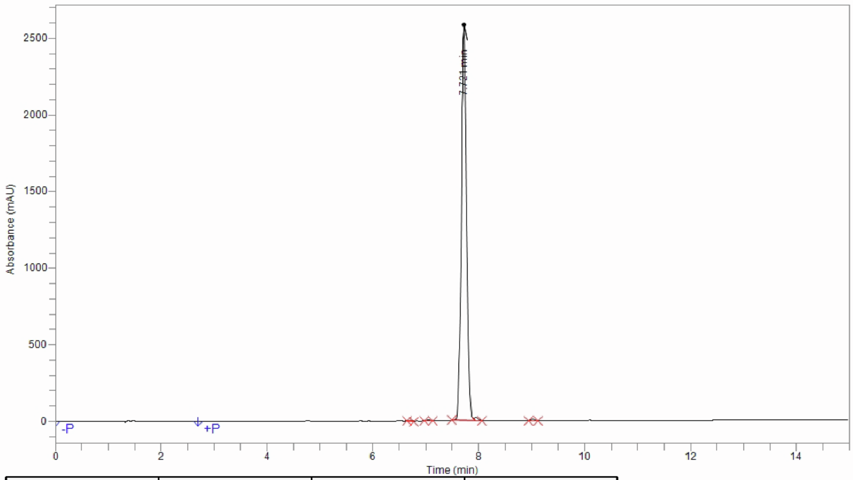

Time	Height	Area	Area %
6.703	5,278.4	22,946.9	0.13
7.053	5,771.8	27,979.5	0.16
7.721	2,585,241.8	17,486,575.7	98.82
7.952	20,853.4	105,450.1	0.60
9.023	10,176.2	52,548.6	0.30
**Total**		17,695,500.7	100.00

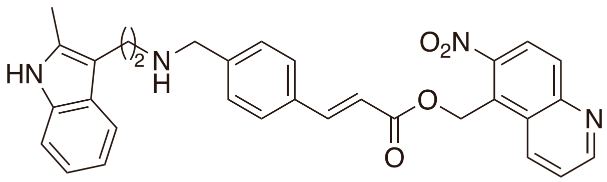

N,N-Di-Boc-panobinostat (**14**)

Acquisition Method	Purity short run @254 nm
Acquisition Date/Time	1/4/2019 11:13 am
Injection Volume	10
Sample Name	EC4-42 +TFA
Sample Description	
Batch Description	

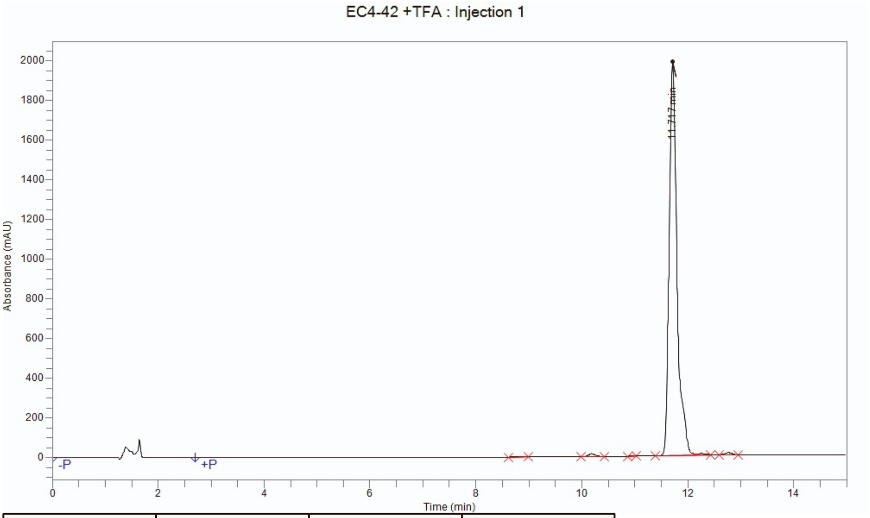

Time	Height	Area	Area %
8.752	3,563.8	31,672.7	0.15
10.185	14,619.4	115,771.7	0.57
11.006	193.5	1,350.5	0.01
11.717	1,986,041.2	20,146,471.9	98.52
12.264	5,418.6	54,861.0	0.27
12.770	11,253.5	97,984.8	0.48
**Total**		20,448,112.5	100.00

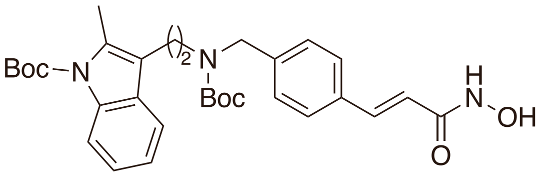

O-((1-Methyl-2-nitroimidazol-5-yl)methyl)-Panobinostat di-trifluoroacetate (CH-03, NI-Pano, **1**)

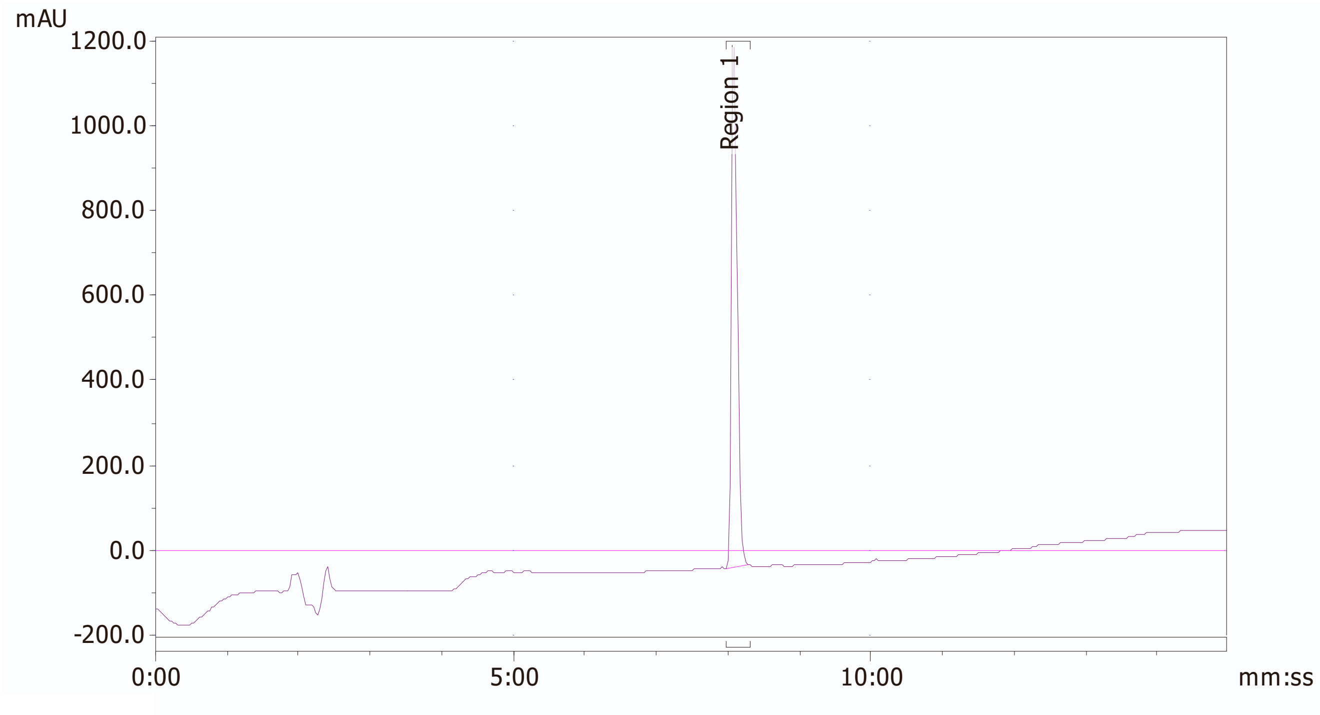

<u>Regions:</u> <u>DA-C@220nm</u> Detector:

Name	Start (mm:ss)	End (mm:ss)	Retention (mm:ss)	Area (mAU·s)	%ROI (%)	%Total (%)
Region 1	7:58	8:18	8:04	6361.4	100.00	N/A
1 Peak				6361.4	100.00	N/A

Total Area: -33335.6 mAU
Average Background: N/A mAU

<u>Method:</u> <u>Xbridge_nonRadio_default</u>

Instrument: N/A Serial no FR1A/0217/389

Run Length: 15m
Dwell: 1s

Cell Volume: 10 µL
Cell Type: Solid
Eluate Flow: 1.00 mL/min
Residence Time: 0.6s
Injection Volume: 10 µL

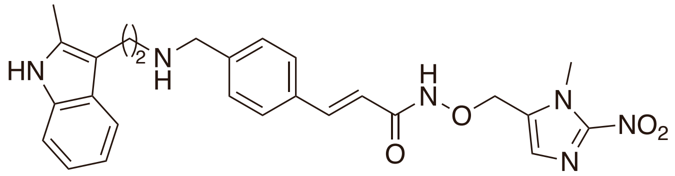

O-((1-Methyl-2-nitroimidazol-5-yl)methyl)-Panobinostat di-trifluoroacetate (CH-03, NI-Pano, **1**)

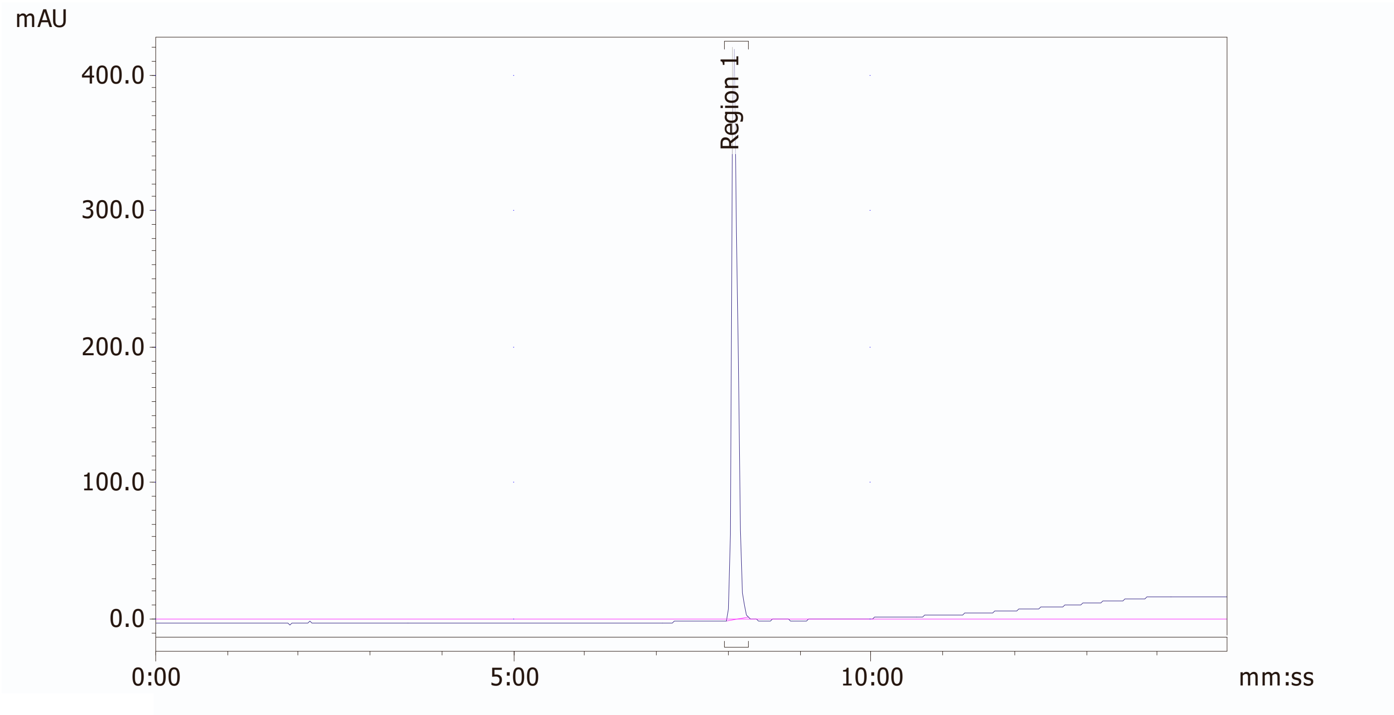

Regions: DA-B@254nm Detector:

Name	Start (mm:ss)	End (mm:ss)	Retention (mm:ss)	Area (mAU·s)	%ROI (%)	%Total (%)
Region 1	7:57	8:16	8:04	2147.8	100.00	62.27
1 Peak				2147.8	100.00	62.27

Total Area: 3448.9 mAU
Average Background: N/A mAU

Method: Xbridge_nonRadio_default

Instrument: N/A Serial no FR1A/0217/389

Run Length: 15m
Dwell: 1s

Cell Volume: 10 µL
Cell Type: Solid
Eluate Flow: 1.00 mL/min
Residence Time: 0.6s
Injection Volume: 10 µL

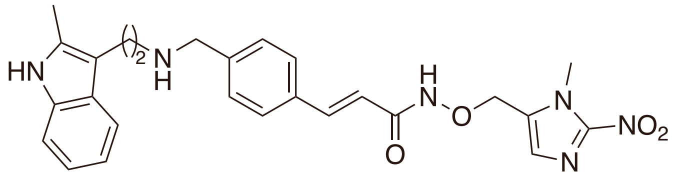

O-((1-Methyl-2-nitroimidazol-5-yl)methyl)-Panobinostat di-trifluoroacetate (CH-03, NI-Pano, **1**)

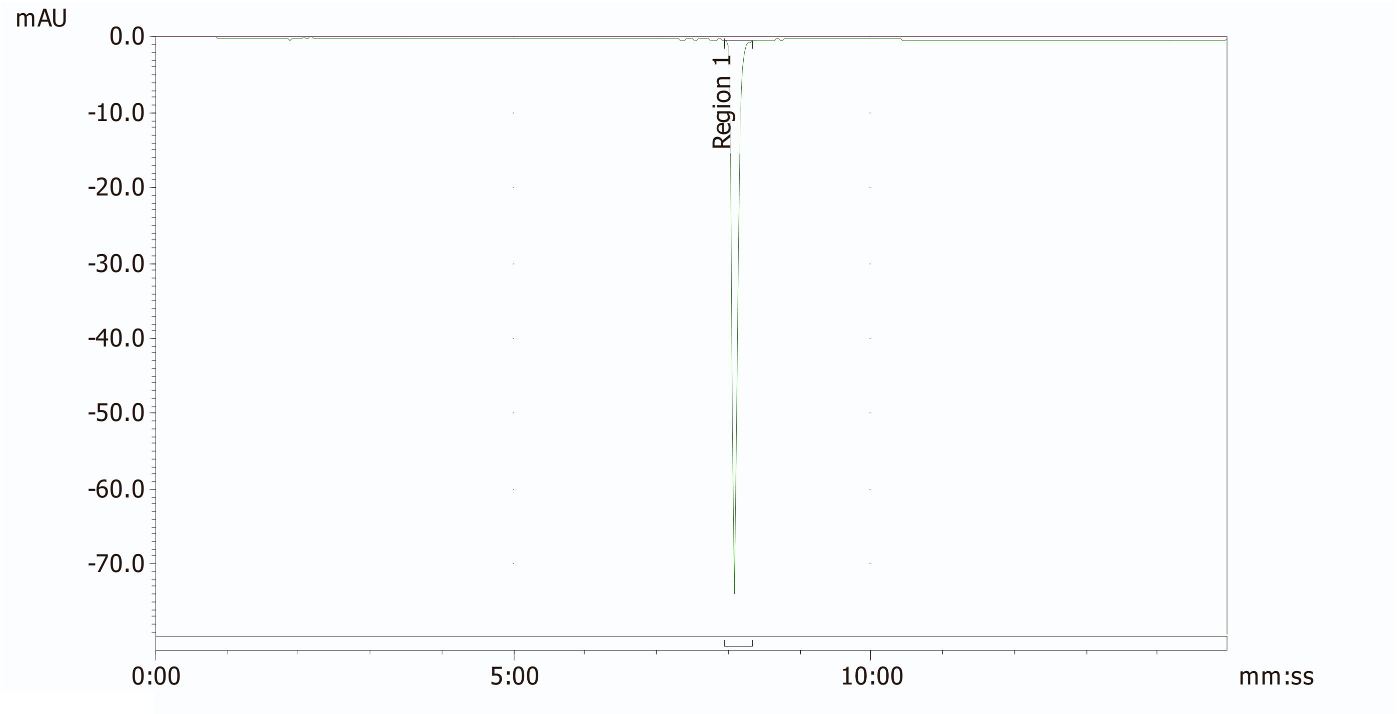

Regions: DA-D@365nm Detector:

Name	Start (mm:ss)	End (mm:ss)	Retention (mm:ss)	Area (mAU·s)	%ROI (%)	%Total (%)
Region 1	7:57	8:20	7:57	-400.8		N/A
1 Peak				-400.8	100.00	N/A

Total Area: -635.4 mAU
Average Background: N/A mAU

Method: Xbridge_nonRadio_default

Instrument: N/A Serial no FR1A/0217/389

Run Length: 15m
Dwell: 1s

Cell Volume: 10 µL
Cell Type: Solid
Eluate Flow: 1.00 mL/min
Residence Time: 0.6s
Injection Volume: 10 µL

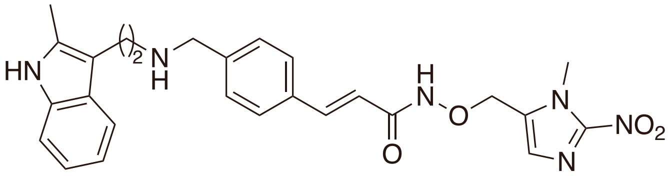

(*E*)-3-(4-{[2-(2-Methyl-1*H*-indol-3-yl)ethylamino]methyl}phenyl)prop-2-enoic acid trifluoroacetate (**S18**)

EC5-05 **11/15/2018**

Acquisition Method	Purity short run @254 nm
Acquisition Date/Time	11/13/2018 5:31 pm
Injection Volume	10
Sample Name	EC5-05
Sample Description	
Batch Description	

EC5-05 : Injection 1

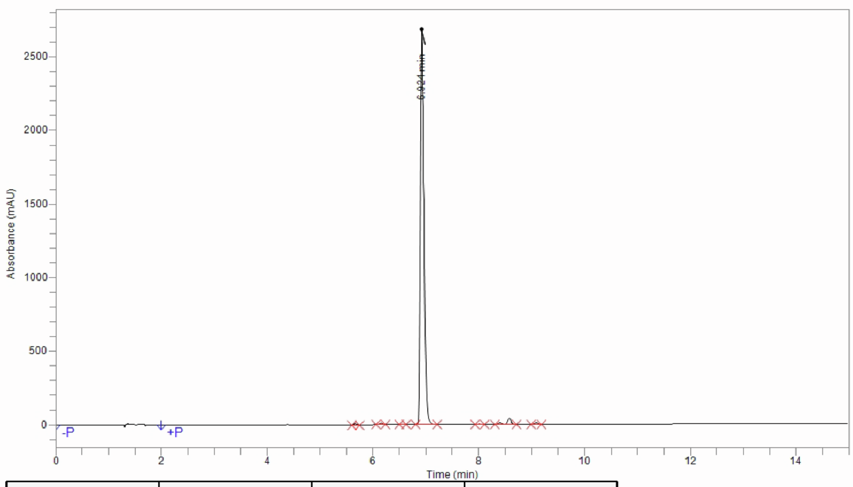

Time	Height	Area	Area %
5.666	7,378.7	24,641.2	0.18
6.151	12,629.0	48,704.8	0.35
6.560	4,172.3	14,854.3	0.11
6.924	2,689,301.6	13,536,942.6	96.97
8.011	4,110.7	19,689.0	0.14
8.397	12,240.3	59,987.8	0.43
8.578	41,434.1	197,179.3	1.41
9.088	12,533.8	57,532.1	0.41
**Total**		13,959,531.1	100.00

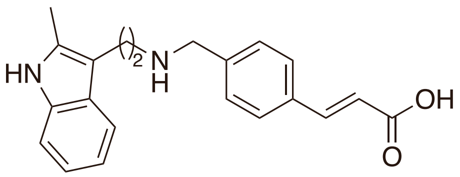

(*E*)-2-(2-(4-((1-Methyl-2-nitro-1*H*-imidazol-5-yl)methoxy)styryl)-4*H*-chromen-4-ylidene)malononitrile

Acquisition Method	Purity short run @254 nm
Acquisition Date/Time	1/15/2021 12:40 pm
Injection Volume	10
Sample Name	MLOx120
Sample Description	
Batch Description	

MLOx120 : Injection 1

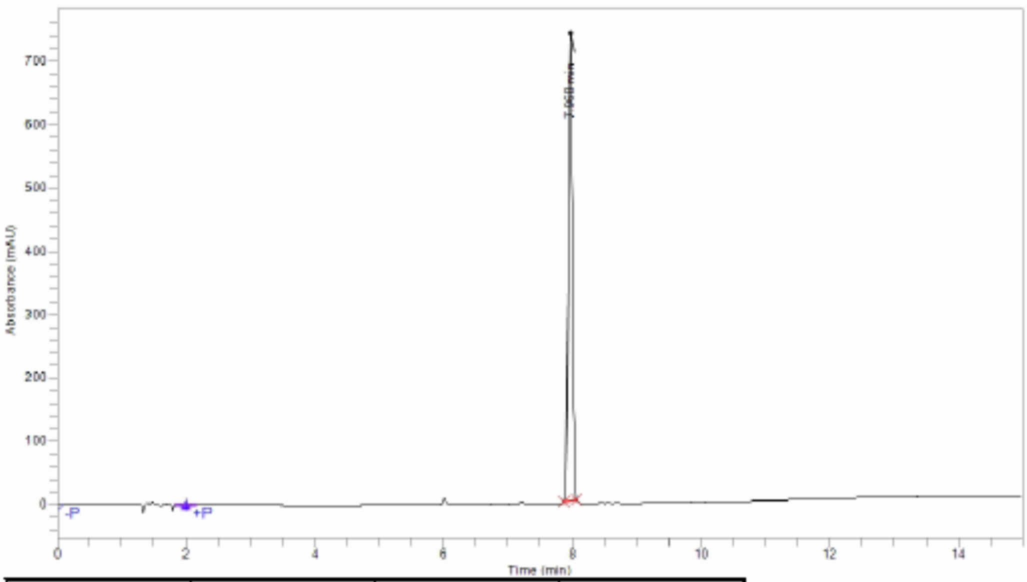

Time	Height	Area	Area %
7.968	738,461.0	3,213,778.3	100.00
Total		3,213,778.3	100.00

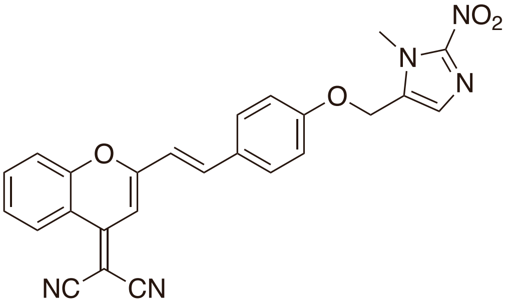

Supplement: Document S1. Figures S1–S6, Scheme S1–S3, and Data S1 and S2 [file mmc1.pdf]
